# Effects of metabolic traits, lifestyle factors, and pharmacological interventions on liver fat: mendelian randomisation study

**DOI:** 10.1136/bmjmed-2022-000277

**Published:** 2022-12-20

**Authors:** Shuai Yuan, Jie Chen, Marijana Vujkovic, Kyong-Mi Chang, Xue Li, Susanna C Larsson, Dipender Gill

**Affiliations:** 1 Unit of Cardiovascular and Nutritional Epidemiology, Institute of Environmental Medicine, Karolinska Institutet, Stockholm, Sweden; 2 Centre for Global Health, Zhejiang University School of Medicine, Hangzhou, China; 3 Department of Gastroenterology, The Third Xiangya Hospital, Central South University, Changsha, China; 4 Department of Big Data in Health Science, School of Public Health and The Second Affiliated Hospital, Zhejiang University School of Medicine, Hangzhou, Zhejiang, China; 5 Corporal Michael J Crescenz VA Medical Center, Philadelphia, PA, USA; 6 Department of Medicine, University of Pennsylvania, Perelman School of Medicine, Philadelphia, PA, USA; 7 Unit of Medical Epidemiology, Department of Surgical Sciences, Uppsala University, Uppsala, Sweden; 8 Department of Epidemiology and Biostatistics, School of Public Health, Imperial College London, London, UK; 9 Chief Scientific Advisor Office, Research and Early Development, Novo Nordisk, Copenhagen, Denmark; 10 Medical Research Council Biostatistics Unit, Cambridge Institute of Public Health, Cambridge, UK

## Abstract

**Objective:**

To investigate the effects of metabolic traits, lifestyle factors, and drug interventions on liver fat using the mendelian randomisation paradigm.

**Design:**

Mendelian randomisation study.

**Setting:**

Publicly available summary level data from genome-wide association studies.

**Participants:**

Genome-wide association studies of 32 974 to 1 407 282 individuals who were predominantly of European descent.

**Exposures:**

Genetic variants predicting nine metabolic traits, six lifestyle factors, four lipid lowering drug targets, three antihypertensive drug targets, and genetic association estimates formagnetic resonance imaging measured liver fat.

**Main outcome measures:**

Mendelian randomisation analysis was used to investigate the effects of these exposures on liver fat, incorporating sensitivity analyses that relaxed the requisite modelling assumptions.

**Results:**

Genetically predicted liability to obesity, type 2 diabetes, elevated blood pressure, elevated triglyceride levels, cigarette smoking, and sedentary time watching television were associated with higher levels of liver fat. Genetically predicted lipid lowering drug effects were not associated with liver fat; however, β blocker and calcium channel blocker antihypertensive drug effects were associated with lower levels of liver fat.

**Conclusion:**

These analyses provide evidence of a causal effect of various metabolic traits, lifestyle factors, and drug targets on liver fat. The findings complement existing epidemiological associations, further provide mechanistic insight, and potentially supports a role for drug interventions in reducing the burden of hepatic steatosis and related disease. Further clinical study is now warranted to investigate the relevance of these genetic analyses for patient care.

**WHAT IS ALREADY KNOWN ON THIS TOPIC**

- Fat accumulation in the liver contributes to the development of chronic liver disease and is implicated in adverse cardiometabolic outcomes
- Several metabolic, lifestyle, and pharmacological factors have been implicated in the accumulation of liver fat, however, evidence for their causal effects is limited

**WHAT THIS STUDY ADDS**

- Genetic evidence supports causal effects of increased adiposity, type 2 diabetes (including raised fasting insulin levels), systolic blood pressure, smoking, alcohol consumption, and sedentary time watching television on increasing liver fat
- Genetic evidence supports protective effects on liver fat of higher low density lipoprotein cholesterol and high density lipoprotein cholesterol levels, but detrimental effects of higher triglyceride levels
- No strong genetic evidence to support effects of lipid lowering drug targets on liver fat, but some evidence supports blood pressure lowering through β blocker and calcium channel blocker antihypertensive drugs might reduce liver fat

**HOW THIS STUDY MIGHT AFFECT RESEARCH, PRACTICE, OR POLICY**

- The findings complement existing epidemiological associations, further provide mechanistic insight, and support a potential role for drug interventions in reducing the burden of hepatic steatosis and related disease
- Further clinical study is now warranted to investigate the relevance of these genetic analyses for patient care

## Introduction

The accumulation of fat in the liver contributes to the development of chronic liver disease and is implicated in various adverse cardiometabolic outcomes.^1 2^ Hepatic steatosis, which is defined by a liver fat content of more than 5.5%, has an estimated prevalence of 25% globally, with rates rapidly increasing as the burden of diabetes and obesity also rises.^3^ Several metabolic, lifestyle, and pharmacological factors have been associated in the accumulation of liver fat. The most commonly encountered comorbidities are obesity, type 2 diabetes mellitus, hyperlipidaemia, and hypertension.^4^ In terms of lifestyle factors, alcohol consumption, cigarette smoking, physical inactivity, and caffeine consumption have most closely been linked with development of liver fat.^5^ Consensus guidance advocates alcohol cessation, weight loss, and specific drug treatments for reducing liver fat and the associated risk of hepatic steatosis.^6^ However, broader evidence of the causal effects of metabolic traits, lifestyle factors, and drug treatments on liver fat is limited. Furthermore, conventional epidemiological studies investigating these areas are limited in their ability to draw causal inferences because of the potential for spurious associations arising due to environmental confounding or reverse causation.

Use of mendelian randomisation leverages randomly allocated genetic variants as instrumental variables for investigating the effect of modifying an exposure on the risk of an outcome.^7^ The random distribution of genetic variants means that their associations with disease risk are not subject to confounding from environmental factors. Furthermore, the allocation of genetic variants at conception means that their associations with disease outcomes are unlikely to be attributable to reverse causation. In this study, we use two sample summary data mendelian randomisation to investigate the effects of metabolic traits, lifestyle factors, and lipid lowering and antihypertensive drug interventions on liver fat. The independent roles were explored by use of multivariable mendelian randomisation analysis for traits with genetic correlations. By identifying modifiable causal risk factors for hepatic steatosis, we offer insight into potential therapeutic strategies for lowering the burden of liver disease and related adverse cardiometabolic outcomes.

## Methods

### Study design

**Figure 1:**
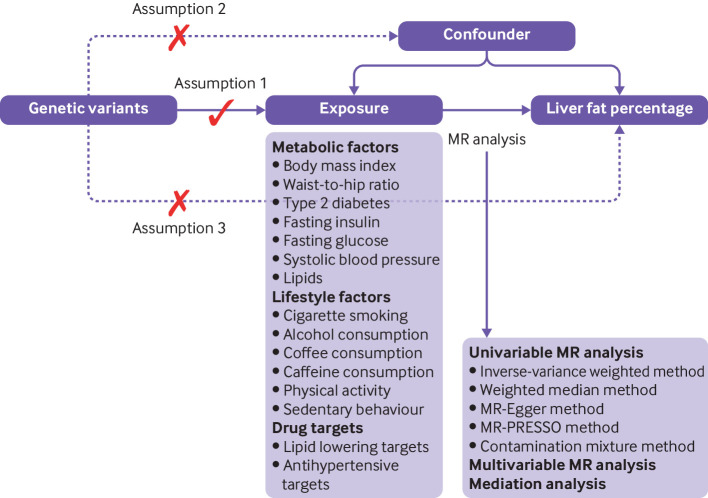
Directed acyclic graph and key assumptions of mendelian randomisation (MR) design. For details of assumptions, please refer to the study design part in the manuscript. MR-PRESSO=Mendelian Randomization Pleiotropy Residual Sum and Outlier

This study is a two sample, mendelian randomisation study based on publicly available summary level data for nine metabolic traits, six lifestyle factors, four lipid lowering drug targets, three antihypertensive drug targets, and liver fat measured by magnetic resonance imaging. Figure 1 shows the study design and three key assumptions of mendelian randomisation analysis: the genetic variants used as instrumental variables should be strongly associated with the exposure; the genetic variants for the exposure should not be associated with any confounders in the association between the exposure and outcome (independence assumption); and the genetic variants affect the outcome merely through their effects on the exposure, but not via other alternative pathways (exclusion restriction assumption). All studies that we used had been approved by corresponding ethical review committees.

### Genetic instrument selection

We selected single nucleotide polymorphisms associated at genome-wide significance (P<5×10^–8^) with nine metabolic traits (body mass index, waist-to-hip ratio, type 2 diabetes, fasting insulin, fasting glucose, systolic blood pressure, high density cholesterol (HDLC) and low density lipoprotein cholesterol (LDLC), and triglycerides) and six lifestyle factors (smoking initiation, alcohol, coffee and caffeine consumption, strenuous sports, and television watching) from corresponding genome-wide association studies (table 1). Linkage disequilibrium among these single nucleotide polymorphisms was estimated based on the 1000 Genomes European reference panel.^8^ Single nucleotide polymorphisms in linkage disequilibrium (*r^2^* >0.01) were removed and the single nucleotide polymorphisms with the smallest P value for the genetic association were retained. Three additional smoking related traits (age of smoking initiation, cigarettes per day, and lifetime smoking index^9^) with selected single nucleotide polymorphisms based on the same approach as mentioned previously were included to further explore the smoking association. These smoking related traits were considered as supplementary exposures because of sample overlap in the population used to measure them with the liver fat outcome, and their smaller explained phenotypical variance as compared with the analysis consider smoking initiation, which could potentially introduce bias or reduce statistical power. We followed the same criteria for instrumental variable selection in multivariable mendelian randomisation analysis, first combining single nucleotide polymorphisms associated with either exposure at P<5×10^–8^ and then removing single nucleotide polymorphisms in high linkage disequilibrium.

**Table 1:** Data sources on metabolic and lifestyle factors and drug targets

Exposure	Instrumental variables	Unit	Participants included in analysis	Sample overlap (%)	Adjustments
**Metabolic factor**					
Body mass index^33^	312	SD	806 834 individuals of European descent	4.1	Age, sex, and genetic 1-5 principal components
Waist-to-hip ratio^33^	581	SD	697 734 individuals of European descent	4.7	Age, sex, and genetic 1-5 principal components
Type 2 diabetes^34^	497	One unit in log-transformed odds	228 499 type 2 diabetes cases and 1 178 783 non-cases of multi-ancestries	0.0	Age, sex, and the first 10 genetic principal components
Fasting insulin^35^	38	log pmol/L	Up to 196 991 individuals of European descent	0.0	BMI, study specific covariates, and principal components
Fasting glucose^35^	71	mmol/L	Up to 196 991 individuals of European descent	0.0	BMI index, study specific covariates, and principal components
Systolic blood pressure^36^	228	10 mm Hg	Up to 1 006 863 individuals of European descent	3.3	Age, sex, BMI, genotyping chips
HDL cholesterol^37^	473	SD	403 943 individuals of European descent	8.2	Age, sex, and genotyping chips
LDL cholesterol^37^	199	SD	440 546 individuals of European descent	7.5	Age, sex, and genotyping chips
Triglycerides^37^	392	SD	441 016 individuals of European descent	7.5	Age, sex, and genotyping chips
**Lifestyle factor**					
Smoking initiation^38^	314	SD in log-transformer odds	1 232 091 individuals of European descent	2.7	Age, sex, and the first 10 genetic principal components
Age of smoking initiation^38^	7	SD	Up to 262 990 individuals of European descent	12.5	Age, sex, and the first 10 genetic principal components
Cigarettes per day^38^	19	SD	Up to 263 954 individuals of European descent	12.5	Age, sex, and the first 10 genetic principal components
Lifetime smoking index^9^	126	SD change of lifetime smoking index	462 690 individuals of European descent	7.1	Genotyping chip and sex
Alcohol drinking^38^	84	SD increase of log-transformed alcoholic drinks/week	941 280 individuals of European descent	3.5	Age, sex, and the first 10 genetic principal components
Coffee consumption^39^	12	50% change	375 833 individuals of European descent	8.8	Age, sex, BMI, total energy, proportion of typical food intake, and 20 genetic principal components
Caffeine consumption^40^	24	SD	362 316 individuals of European descent	9.1	Age, sex, genotyping array, and the first 30 genetic principal components
Strenuous sports^41^	6	≥ 2–3 *v* 0 days/week	350 492 individuals of European descent	9.4	Age, sex, genotyping chip, first 10 genomic principal components, centre, and season
Television watching^42^	112	Hours/day	422 218 individuals of European descent	7.8	Age squared, age, sex, age-sex interaction, the first 30 principal components
**Drug target**					
Lipid lowering target^10^	One for HMGCR, two for LDLR, one for NPC1L1, two for PCSK9	10 mg/dL LDL cholesterol	80 959 to 295 826 individuals of European descent	0	Age, sex, study sample, and five principal components
Antihypertensive target^11^	One for ACEi, six for β blockers, 23 for CCBs	10 mm Hg systolic blood pressure	757 601 individuals of European descent	4.4	Age squared, age, sex, BMI, genotyping chips

Sample overlap was calculated by the sample size of the outcome (n=32 974) divided by the sample size of the exposure whose data sources included UK Biobank. ACEi=angiotensin converting enzyme inhibitor; BMI=body mass index; CCBs=calcium channel blockers; HDL=high density lipoprotein; LDL=low density lipoprotein; SD=standard deviation.

Genetic variants proxying the effects of lipid lowering and antihypertensive drug treatments were obtained by use of approaches similar to previous mendelian randomisation studies.^10 11^ For lipid lowering drugs, single nucleotide polymorphisms associated with LDLC concentrations at the genome-wide significance level (P<5×10^–8^) and located in gene regions corresponding to four drug targets (HMGCR (3-hydroxy-3-methyl-glutaryl-coenzyme A reductase), LDLR (low density lipoprotein receptor), NPC1L1 (Niemann-Pick C1-like 1), and PCSK9 (proprotein convertase subtilisin/kexin type 9)) were obtained from the Global Lipids Genetics Consortium.^10^ For antihypertensive drugs, genes encoding drug targets for ACEi (angiotensin converting enzyme inhibitor), β blockers, and calcium channel blockers were identified in DrugBank.^11^ Single nucleotide polymorphisms in identified gene regions associated with systolic blood pressure at the genome-wide significance level (P<5×10^–8^) were obtained from the International Consortium of Blood Pressure.^11^ Single nucleotide polymorphisms with *r^2^* <0.01 were selected as instrumental variables.^11^ Details for data sources are displayed in table 1 and information about genetic instruments online supplemental table 1.

10.1136/bmjmed-2022-000277.supp1 Supplementary data

### Outcome data source

Summary level data for hepatic fat measured by abdominal magnetic resonance imaging were obtained from a genome-wide association analysis of 32 974 generally healthy adults of European descent in the UK Biobank study.^12^ The UK Biobank study is an ongoing cohort study collecting phenotypic and genetic data from more than 500 000 individuals since its initiation in 2006-10.^13^ The genetic associations with liver fat were scaled to one standard deviation (SD) of liver fat percentage and one SD increase equals about 4.25 unit increase of absolute liver fat percentage points.^12^ The characteristics of the population are described in online supplemental table 2. Liver fat was quantified by a machine learning algorithm trained on a small subset (n=4511) with previously quantified liver fat values.^12^ Single nucleotide polymorphismsassociated with liver fat were adjusted for sex, year of birth, age at time of MRI, age at time of MRI squared, genotyping array, MRI device serial number, and the first 10 principal components of genetic variation. These adjustments were likely made in the original study to account for potential population stratification and confounding effects.^12^

### Statistical analysis

We harmonised all variants serving as instrumental variables between the exposure and outcome data by effect allele. Given that a few single nucleotide polymorphisms were unavailable in the outcome data, we did not find proxies to replace missing single nucleotide polymorphisms. We calculate *F* statistic for metabolic and lifestyle factors in univariable mendelian randomisation analysis.^14^ Similarly, conditional *F* statistic was estimated to inform the instrument strength in multivariable mendelian randomisation analysis.

The inverse variance weighted mendelian randomisation method was used as the main analysis. The model under the multiplicative random effects was used for the traits with at least three single nucleotide polymorphisms and that model under the fixed effects was used for the traits with one or two single nucleotide polymorphisms. The weighted median,^15^ mendelian randomisation-Egger,^16^ mendelian randomisation pleiotropy residual sum and outlier (MR-PRESSO),^17^ and contamination mixture,^18^ are mendelian randomisation sensitivity analysis methods that were used to examine the robustness of the mendelian randomisation associations to pleiotropic variants that might be biasing the underlying assumptions of mendelian randomisation. The weighted median method can provide a robust mendelian randomisation estimate if 50% of the genetic instruments are valid.^19^ Mendelian randomisation-Egger regression can detect horizontal pleiotropy (violation of the exclusion restriction assumption that single nucleotide polymorphisms affect fat liver not merely through the exposure) by its intercept test and generate corrected mendelian randomisation estimates after adjusting for pleiotropic effects, although with a relatively low level of precision.^16^ MR-PRESSO can identify variants outlying in their mendelian randomisation estimates and provide a corrected estimate after removal of such outlier variants.^17^ The contamination mixture method can generate accurate mendelian randomisation estimates when a large number of variants are available, even if a proportion are invalid.^18^ To additionally minimise the direct influence of variants selected as instruments on the outcome, we performed a sensitivity analysis where we excluded single nucleotide polymorphisms that were associated with liver fat at the loci-wide significance level (P<1×10^–5^). We also conducted a Steiger directionality test to examine the possible reverse causality.^19^ Cochran’s Q value was used to assess heterogeneity among single nucleotide polymorphism estimates as an indicator of their pleiotropic effects.^20^

We performed multivariable mendelian randomisation analysis for the metabolic traits, with adjustment for genetically predicted body mass index. This analysis had two aims: to minimise potential pleiotropic effects from body mass index on the associations between metabolic factors and liver fat, and to examine for potential mediating roles of metabolic factors in the association between body mass index and liver fat. Network mendelian randomisation was used to estimate the proportion of the total effect of body mass index on liver fat that is mediated through other metabolic factors.^21^ Given correlations across three lipid biomarkers and correlation between smoking and alcohol consumption, we also conducted multivariable mendelian randomisation analyses with mutual adjustments for these sets of traits. The Benjamini-Hochberg false discovery rate correction was used to account for multiple testing.^22^ All tests were two sided and done using the TwoSampleMR,^23^ MR-PRESSO,^17^ and MendelianRandomization,^19^ packages in the R software (version 4.0.2).

### Patients and public involvement

No patients or members of the public were involved in the design or reporting of this study. On publication, the study findings will be disseminated to the public through the authors’ institutional research media offices.

## Results

**Figure 2:**
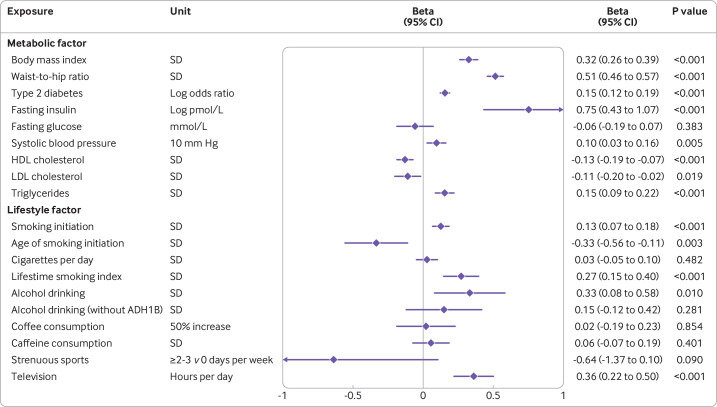
Associations of genetically proxied metabolic and lifestyle factors with hepatic fat. The x axis unit is standard deviation (SD) change in liver fat percentage. One SD increase is approximately a 4.25 unit increase of absolute liver fat percentage points. CI=confidence interval; HDL=high density lipoprotein; LDL=low density lipoprotein

Sample overlap was 0-12.5% between the exposures and the outcome data sources (table 1). All estimated *F* statistics were more than 10, which indicated limited bias caused by sample overlap (online supplemental table 3). For eight of the nine metabolic factors and three of the six lifestyle factors considered, significant associations with hepatic fat were noted (figure 2). All associations persisted after the false discovery rate correction for multiple testing (online supplemental table 4). The change of liver fat was 0.32 SD units (95% confidence interval 0.26 to 0.39) per one SD increase in genetically predicted body mass index, 0.51 (0.45 to 0.57) per one SD increase in waist-to-hip ratio; 0.15 (0.12 to 0.19) per one unit increase in log-transformed odds ratio of type 2 diabetes; 0.75 (0.43 to 1.07) per one log-transformed pmol/L increase in fasting insulin levels; 0.10 (0.03 to 0.16) per 10 mm Hg increase in systolic blood pressure; −0.13 (−0.19 to –0.07), −0.11 (−0.20 to –0.02), and 0.15 (0.09 to 0.22) per one SD increase in levels of HDLC, LDLC, and triglycerides, respectively; 0.13 (0.07 to 0.18) per one SD increase in log-transformed odds ratio of smoking initiation; 0.33 (0.08 to 0.58) per one SD increase in log-transformed alcoholic drinks per week; and 0.36 (0.22 to 0.50) per one hour increase in television watching time. No strong associations with fat liver were recorded with genetically predicted fasting glucose concentrations, coffee consumption, caffeine (either from coffee or tea) consumption, or strenuous sports (figure 2). We observed an inverse association of genetically predicted age of smoking initiation and a positive association of genetically predicted lifetime smoking index with liver fat (figure 1). However, genetically predicted cigarettes smoked per day showed no strong association with liver fat (figure 1). The association for genetically predicted alcohol consumption did not remain significant after excluding single nucleotide polymorphisms in the *ADH1B* gene region (figure 2).

The associations for genetically predicted metabolic and lifestyle factors were overall consistent in sensitivity analyses, although with wider 95% confidence interval with the weighted median and mendelian randomisation-Egger regression analyses (online supplemental table 5). Moderate to high heterogeneity was observed in the analyses of waist-to-hip ratio, type 2 diabetes, the three lipid biomarkers, alcohol and coffee consumption, strenuous sports, and television watching (online supplemental table 5). We observed evidence of bias in the mendelian randomisation-Egger intercept test for type 2 diabetes, lipids, and alcohol consumption (P for intercept test<0.05). One to 15 outliers were identified in MR-PRESSO analyses; however, all observed associations remained consistent after removal of outliers (online supplemental table 5). The associations were also consistent in the sensitivity analysis after removing single nucleotide polymorphisms that were strongly associated with liver fat (online supplemental table 6). However, Steiger directionality tests indicated possible reverse causality for the associations of alcohol consumption and strenuous sports, respectively, with liver fat (online supplemental table 3).

**Figure 3:**
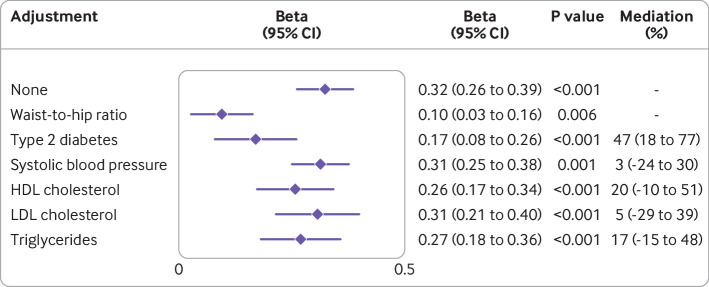
Associations of genetically predicted body mass index with hepatic fat, with and without adjustment for other metabolic factors. CI=confidence interval; HDL=high-density lipoprotein; LDL=low-density lipoprotein

Conditional *F* statistics for traits included in multivariable mendelian randomisation analysis were more than 10 except for smoking initiation in the multivariable mendelian randomisation analysis with mutual adjustment for smoking initiation and alcohol consumption. The association attenuated for genetically predicted body mass index in a multivariable analysis that adjusted for genetically predicted levels of other metabolic factors (figure 3). Among five possible mediators, liability to type 2 diabetes appeared to mediate almost half (47% (95% confidence interval 18% to 77%)) of the effect of body mass index on liver fat. Adjustment for genetically predicted levels of other metabolic factors resulted in relatively little attenuation of the association for genetically predicted body mass index (table 2). Similarly, the pattern of the mendelian randomisation associations for lipid biomarkers only changed slightly in the multivariable model with mutual adjustment (table 2). The associations for genetically predicted smoking initiation and alcohol consumption also attenuated only slightly after mutual adjustment (table 2).

**Table 2:** Multivariable associations of genetically proxied metabolic and lifestyle factors with hepatic fat

Exposure	Adjustment	Without adjustment		With adjustment	
		Beta (95% CI)	P value	Beta (95% CI)	P value
Waist-to-hip ratio	Body mass index	0.51 (0.46 to 0.57)	<0.001	0.49 (0.42 to 0.55)	<0.001
Type 2 diabetes	Body mass index	0.15 (0.12 to 0.19)	<0.001	0.14 (0.11 to 0.17)	<0.001
Systolic blood pressure	Body mass index	0.10 (0.03 to 0.16)	0.005	0.10 (0.04 to 0.15)	0.001
HDL cholesterol	Body mass index	−0.13 (−0.19 to −0.07)	<0.001	−0.11 (−0.16 to −0.06)	<0.001
LDL cholesterol	Body mass index	−0.11 (−0.2 to −0.02)	0.019	−0.11 (−0.18 to −0.04)	0.002
Triglycerides	Body mass index	0.15 (0.09 to 0.22)	<0.001	0.14 (0.08 to 0.21)	<0.001
HDL cholesterol	LDL cholesterol and triglycerides	−0.13 (−0.19 to −0.07)	<0.001	−0.11 (−0.18 to −0.04)	0.004
LDL cholesterol	HDL cholesterol and triglycerides	−0.11 (−0.20 to −0.02)	0.019	−0.13 (−0.20 to −0.06)	0.001
Triglycerides	LDL and HDL cholesterol	0.15 (0.09 to 0.22)	<0.001	0.16 (0.07 to 0.24)	0.001
Smoking initiation	Alcohol drinking	0.13 (0.07 to 0.18)	<0.001	0.09 (0.02 to 0.15)	0.009
Alcohol drinking	Smoking initiation	0.33 (0.08 to 0.58)	0.010	0.18 (0.01 to 0.35)	0.036
Alcohol drinking (without *ADH1B*)	Smoking initiation	0.15 (−0.12 to 0.42)	0.281	−0.04 (−0.24 to 0.15)	0.659

The unit for liver fat was standard deviation of liver fat percentage. HDL=high density lipoprotein; LDL=low density lipoprotein.

**Figure 4:**
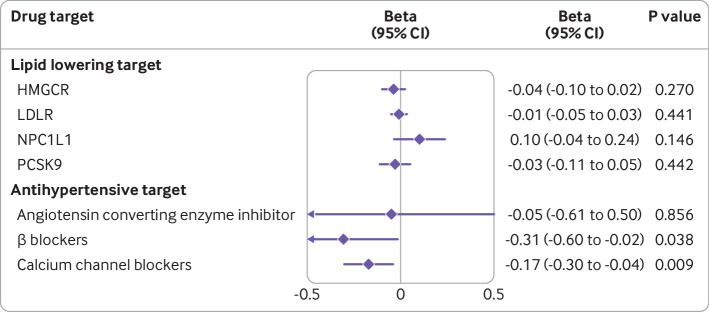
Associations of genetically proxied lipid lowering and antihypertensive therapies with hepatic fat. The associations for genetically proxied lipid lowering target was scaled to a decrease of 10 mg/dL low density lipoprotein cholesterol concentrations. The associations for genetically proxied antihypertensive target was scaled to a decrease of 10 mm Hg systolic blood pressure. The x axis unit is standard deviation (SD) change in liver fat percentage. One SD increase is approximately a 4.25 unit increase of absolute liver fat percentage points. CI=confidence interval; HMGCR=3-hydroxy-3-methyl-glutaryl-coenzyme A reductase; LDLR=low density lipoprotein receptor; NPC1L1=Niemann-Pick C1-like 1; PCSK9=proprotein convertase subtilisin/kexin type 9

Genetically proxied β blocker and calcium channel blocker effects, but not lipid lowering or ACEi antihypertensive drug effects, were associated with lower levels of liver fat (figure 4). In genetically predicted systolic blood pressure via β blocker, a change in liver fat of −0.31 (95% confidence interval −0.60 to –0.02) per 10 mm Hg decrease was observed and via calcium channel blocker effect, −0.17 (−0.30 to –0.04) was observed. The associations were consistent in sensitivity analyses and unlikely to be biased by horizontal pleiotropy (online supplemental table 5). However, the association for genetically proxied β blockers did not pass the false discovery rate correction (online supplemental table 4).

## Discussion

### Principal findings

This mendelian randomisation analysis identified evidence supporting causal effects on increasing liver fat of increased adiposity, type 2 diabetes (including raised fasting insulin levels), systolic blood pressure, smoking, alcohol consumption, and sedentary time watching television. Additionally, genetic evidence supports the protective effects on liver fat of higher LDLC and HDLC concentrations, but detrimental effects of higher triglyceride levels. No strong evidence supported the effects of lipid lowering drug targets on liver fat but some evidence showed that blood pressure lowering through β blocker and calcium channel blocker antihypertensive drugs might reduce liver fat. Our findings are consistent with existing understanding on the determinants of fat accumulation in the liver; namely, increased uptake of free fatty acids into the liver, impaired metabolism within the liver, and increased de novo lipogenesis.^24^ In this way, the findings that genetically predicted higher LDLC and HDLC concentrations are associated with lower liver fat levels might be explained by their role in lipid cycling. Regarding our novel genetic evidence for a potential protective effect of β blocker and calcium channel blocker antihypertensive drugs on liver fat, the point estimates are consistent with the mechanism being through blood pressure reduction. Although the relation between blood pressure and liver fat is complicated and can be mediated through insulin resistance,^25^ the mendelian randomisation approach leveraged here supports a causal effect, which warrants further evaluation in clinical studies.

### Strengths

These findings make important advances in our understanding of hepatic steatosis and related diseases.^6^ Firstly, the mendelian randomisation paradigm strengthens the evidence for causal effects of these risk factors, rather than only an association, which could also be attributable to environmental confounding factors and reverse causation. This factor is particularly important for identifying therapeutic targets that reduce disease risk and burden. Secondly, our findings offer novel mechanistic insight. For example, the evidence for a causal effect of insulin resistance on liver fat supports that this mediating mechanism is likely underlying the effect of type 2 diabetes. Previous work has used mendelian randomisation to find evidence supporting an effect of higher liver fat on increasing type 2 diabetes mellitus risk, supporting potential bi-directional effects in this relation.^26^ Similarly, the incorporation of multivariable mendelian randomisation provides evidence that effects of smoking on liver fat occur independently of alcohol consumption, and further evidence that body mass index is having direct effects even after accounting for its effects on other cardiometabolic risk factors, such as diabetes and blood pressure. The mendelian randomisation mediation analysis supported that approximately half of the effect of body mass index on liver fat was occurring through increased liability to type 2 diabetes mellitus, while around a fifth of the effect of body mass index can be mediated through dyslipidaemia. Thirdly, this work is consistent with safety of lipid lowering drugs, given that we did not identify any strong evidence for an effect on increasing liver fat. For the β blocker and calcium channel blocker antihypertensive drugs, evidence suggested that these agents might potentially be reducing liver fat, likely through their effects on lowering blood pressure.

### Comparison with other studies

Our current work is largely consistent with previous epidemiological and genetic analyses. Existing epidemiological investigation has identified strong associations of fatty liver disease with obesity, type 2 diabetes, hyperlipidemia, hypertension, and metabolic syndrome.^27^ Considering lifestyle factors, observational associations have also previously been established for alcohol consumption, smoking, and physical activity, consistent with the pattern of our current findings.^5^ Although evidence for a role of lipid lowering drugs in non-alcoholic fatty liver disease has been mixed, we did not identify any strong support in clinical studies,^28^ which is consistent with the findings of our genetic analyses. This finding might be explained by these drugs primarily targeting homeostasis of lipid levels in the blood and periphery, rather than liver fat. A previous mendelian randomisation analysis considering the outcome of non-alcoholic fatty liver disease similarly found associations for obesity traits, type 2 diabetes liability, blood pressure, and smoking similar to those associations observed in our current work considering liver fat as the outcome,^29^ thus supporting its conclusions. Our estimates for type 2 diabetes mediating approximately half of the effect of body mass index on liver fat are also similar to those obtained when using the mendelian randomisation paradigm to explore the mediating effects of type 2 diabetes for body mass index on cardiovascular disease outcomes.^30^ Our current mendelian randomisation investigation into the effects of drugs is novel and supports further investigation into the notion of β blocker and calcium channel blocker antihypertensive drug treatment for reducing liver fat, thus highlighting the possibility of interventions beyond lifestyle modification, which might also be difficult to implement and maintain in practice.^31^

### Limitations

The main advantage of the mendelian randomisation approach taken in this work is that the analyses is time and cost efficient by use of pre-existing, large scale genetic association data. The random allocation of genetic variants at conception means that the approach is less susceptible to the bias from environmental confounding and reverse causation that can hinder causal inference in a traditional epidemiological study design. However, this approach also has weaknesses. Firstly, the analyses were largely restricted to individuals of European genetic ancestry, thus limiting generalisability to other ancestry groups. Secondly, the mendelian randomisation paradigm explores the effects of small, lifelong changes in the genetically predicted levels of a risk factor on an outcome, in this case liver fat. This differs from estimating the effect of a clinical intervention, which might have a larger magnitude but over a shorter period of life. Similarly, our approach could not inform on the dose-response association between these risk factors and levels of liver fat. Thirdly, the mendelian randomisation approach is susceptible to bias from variants that maybe having pleiotropic effects on the outcome through pathways unrelated to the exposure being considered. Although we incorporated a range of sensitivity analyses to prevent confounding affecting our conclusions, this possibility cannot be entirely excluded. Fourthly, we considered the outcome of liver fat in this work, which in itself, might not directly cause disease. Additionally, we quantified liver fat was by use of a machine learning algorithm,^12^ and the potential for misspecification with this approach should be acknowledged. Further work is required to investigate the relation between high levels of liver fat and consequent liver and cardiometabolic disease risk. Fifthly, even though around 10% of sample overlapped for certain exposures with the outcome, which might inflate type one error rate, *F* statistics for these associations were more than 10, which indicated that the bias caused by this mild sample overlap should have been limited. However, conditional *F* statistic for smoking initiation was less than 10, which might introduce weak instrument bias in multivariable analysis of smoking initiation and alcohol consumption in relation to liver fat. Sixthly, use of genetic instruments from the genome-wide association studies with additional adjustment except for age, sex, and population structure factors (eg, body mass index) might introduce collider bias in mendelian randomisation analysis.^32^ Thus, the associations for fasting insulin and glucose, coffee consumption, strenuous sports, and antihypertensive drugs need to be verified. Seventhly, one in four individuals were defined as excessive alcohol consumption according to the UK criteria in the outcome study,^12^ which might drive the observed associations specific to alcohol-related liver fat accumulation. However, the genetic associations for liver fat appeared to be largely unrelated of alcohol consumption with consistent findings in two supplementary liver fat genome-wide association analyses where individuals who reported having stopped drinking alcohol or who reported excessive alcohol intake were removed and where the self-reported number of alcoholic drinks consumed per week were adjusted for.^12^ Finally, a priori sample size calculations were not performed in this study. Instead, the statistical power of the various analyses can be interpreted through the confidence intervals of the point estimates.

### Conclusion

In conclusion, we provided a wide angled investigation into the effects of metabolic traits, lifestyle factors, and pharmacological interventions on liver fat. The findings largely support the existing evidence on the role of cardiometabolic traits on hepatic steatosis, and further identify potential mediating mechanisms and pharmacological strategies for reducing the burden of related disease. Further work is now warranted to explore these findings in a clinical setting.

## Data Availability

Data are available in a public, open access repository. Data used are publicly available and the original sources are detailed in Table 1.
