# Supplementary material for: Effects of metabolic traits, lifestyle factors, and pharmacological interventions on liver fat: mendelian randomisation study
Source: BMJ Med. 2022 Dec 20;1(1):e000277. doi: 10.1136/bmjmed-2022-000277 (PMC9978690; doi:10.1136/bmjmed-2022-000277)
Supplement: Supplementary data [file bmjmed-2022-000277supp001.pdf]

Mendelian randomization exploration of metabolic and lifestyle factors, lipid-lowering and anti-hypertension therapies in relation to hepatic steatosis in healthy middle-aged adults

Shuai Yuan, Jie Chen, Marijana Vujkovic, Kyong-Mi Chang, Xue Li, Susanna C. Larsson, Dipender Gill

Supplementary Table 1. Genetic instruments in relation to liver fat
Supplementary Table 2. Characteristics of participants in the genome-wide association analysis of hepatic fat
Supplementary Table 3. F statistics and Steiger directionality test for studied traits
Supplementary Table 4. Results of false discovery rate correction
Supplementary Table 5. Results of MR sensitivity analyses
Supplementary Table 6. Sensitivity analysis of removing SNPs associated with liver fat at the loci-wide significance level

Supplementary Table 1. Genetic instruments in relation to liver fat

				Exposure			Liver fat		
**Exposure**	**SNP**	**EA**	**EAF**	**Beta**	**SE**	**P**	**Beta**	**SE**	**P**
AgeSomIni	rs11780471	A	0.08	0.038	0.006	7.07E-11	-0.024	0.016	1.40E-01
AgeSomIni	rs11915747	G	0.37	0.021	0.003	3.89E-13	-0.013	0.008	1.20E-01
AgeSomIni	rs140485736	A	0.01	0.065	0.012	1.39E-08	-0.036	0.035	2.50E-01
AgeSomIni	rs319748	A	0.72	-0.017	0.003	3.01E-08	0.004	0.009	7.90E-01
AgeSomIni	rs3768886	C	0.33	0.017	0.003	7.50E-09	-0.003	0.008	8.30E-01
AgeSomIni	rs624833	G	0.33	0.017	0.003	8.75E-09	-0.004	0.008	5.50E-01
AgeSomIni	rs7599208	T	0.57	-0.020	0.003	1.70E-12	-0.002	0.008	7.20E-01
BMI	rs10920678	A	0.43	0.015	0.002	1.25E-20	-0.001	0.008	9.90E-01
BMI	rs11121210	C	0.59	0.011	0.002	6.60E-11	0.012	0.008	1.10E-01
BMI	rs112566467	T	0.20	0.018	0.002	1.77E-14	0.010	0.010	2.90E-01
BMI	rs11577094	T	0.05	0.019	0.003	5.65E-10	-0.007	0.014	6.30E-01
BMI	rs12049202	T	0.18	0.024	0.002	5.20E-30	0.018	0.010	4.80E-02
BMI	rs12072739	G	0.22	0.017	0.002	2.01E-13	0.014	0.009	1.10E-01
BMI	rs12121950	T	0.36	0.019	0.002	8.88E-25	0.011	0.008	1.80E-01
BMI	rs12140153	G	0.93	0.035	0.003	2.98E-25	0.006	0.013	6.10E-01
BMI	rs17024393	C	0.03	0.064	0.005	1.87E-39	0.044	0.025	5.20E-02
BMI	rs1730859	G	0.32	0.012	0.002	1.68E-12	0.012	0.008	2.00E-01
BMI	rs1884429	T	0.23	0.013	0.002	1.76E-12	-0.004	0.009	5.80E-01
BMI	rs2400414	C	0.64	0.013	0.002	2.56E-12	-0.009	0.008	1.40E-01
BMI	rs2820295	A	0.30	0.024	0.002	5.91E-39	0.009	0.008	2.80E-01
BMI	rs2968487	T	0.28	0.018	0.002	1.40E-22	0.013	0.009	1.80E-01
BMI	rs3101336	C	0.64	0.025	0.002	9.44E-57	-0.005	0.008	4.80E-01
BMI	rs543874	G	0.19	0.048	0.002	9.24E-127	0.002	0.010	7.90E-01
BMI	rs561136	C	0.16	0.019	0.002	9.19E-15	-0.009	0.011	5.00E-01
BMI	rs61813324	T	0.13	0.029	0.003	5.64E-25	0.007	0.012	6.30E-01
BMI	rs61828641	A	0.11	0.022	0.003	1.06E-13	-0.001	0.012	8.50E-01
BMI	rs6661316	T	0.57	0.012	0.002	6.38E-14	0.001	0.008	9.20E-01
BMI	rs6690398	A	0.57	0.013	0.002	1.94E-13	0.010	0.008	2.70E-01
BMI	rs7534091	G	0.26	0.012	0.002	2.62E-11	-0.014	0.009	1.10E-01
BMI	rs79113395	G	0.74	0.020	0.002	9.82E-20	0.009	0.009	2.30E-01
BMI	rs946824	T	0.14	0.020	0.003	3.27E-15	0.000	0.011	8.60E-01
BMI	rs10203386	A	0.43	0.032	0.002	4.43E-78	0.010	0.008	2.30E-01
BMI	rs10497870	A	0.59	0.012	0.002	3.95E-14	-0.017	0.008	2.70E-02
BMI	rs10929925	C	0.52	0.014	0.002	6.99E-19	-0.013	0.008	9.90E-02
BMI	rs11692326	T	0.22	0.015	0.002	1.02E-14	-0.002	0.009	8.40E-01
BMI	rs12692596	T	0.36	0.012	0.002	1.68E-12	0.004	0.008	5.40E-01
BMI	rs12714199	C	0.40	0.014	0.002	1.09E-16	0.005	0.008	4.60E-01
BMI	rs13002946	T	0.72	0.018	0.002	1.21E-20	0.023	0.009	7.80E-03
BMI	rs13021737	G	0.83	0.058	0.002	9.11E-167	-0.004	0.010	7.00E-01
BMI	rs13033310	A	0.25	0.015	0.002	3.22E-11	0.007	0.009	5.30E-01
BMI	rs1451077	G	0.43	0.017	0.002	5.86E-19	0.007	0.008	5.80E-01
BMI	rs1470545	T	0.04	0.037	0.004	7.65E-18	0.044	0.020	2.10E-02
BMI	rs1561554	G	0.39	0.012	0.002	1.34E-11	0.000	0.008	9.40E-01
BMI	rs264962	C	0.60	0.012	0.002	2.56E-12	-0.002	0.008	8.00E-01
BMI	rs2861685	T	0.60	0.017	0.002	3.81E-18	-0.002	0.008	8.30E-01
BMI	rs3770799	G	0.39	0.011	0.002	2.40E-10	0.014	0.008	8.50E-02
BMI	rs4671328	T	0.43	0.021	0.002	2.45E-36	0.017	0.008	3.10E-02
BMI	rs4973618	G	0.34	0.015	0.002	2.00E-16	0.018	0.008	2.20E-02
BMI	rs6545714	G	0.38	0.019	0.002	7.79E-34	-0.009	0.008	3.30E-01
BMI	rs6720868	T	0.34	0.015	0.002	1.17E-17	0.008	0.008	3.20E-01
BMI	rs7561278	T	0.77	0.017	0.002	8.44E-16	0.018	0.009	3.10E-02
BMI	rs7570446	A	0.52	0.011	0.002	5.15E-09	0.006	0.008	3.60E-01

BMI	rs7575118	T	0.13	0.014	0.002	5.43E-09	0.003	0.011	9.00E-01
BMI	rs7588437	G	0.61	0.017	0.002	2.85E-22	0.020	0.008	7.70E-03
BMI	rs7599312	G	0.73	0.018	0.002	4.93E-24	-0.003	0.009	6.10E-01
BMI	rs7607490	A	0.11	0.016	0.003	1.25E-09	-0.017	0.012	1.30E-01
BMI	rs930295	A	0.17	0.021	0.002	1.52E-19	0.004	0.010	5.60E-01
BMI	rs9808302	G	0.48	0.012	0.002	1.03E-09	0.006	0.008	2.80E-01
BMI	rs10510419	G	0.85	0.017	0.002	2.79E-13	-0.004	0.011	9.40E-01
BMI	rs11915371	C	0.19	0.015	0.002	2.24E-13	0.031	0.010	1.50E-03
BMI	rs12635553	A	0.53	0.010	0.002	5.76E-09	-0.003	0.008	7.60E-01
BMI	rs1436344	C	0.59	0.015	0.002	5.28E-18	0.013	0.008	1.20E-01
BMI	rs16851483	T	0.06	0.035	0.003	4.06E-25	0.048	0.016	2.10E-03
BMI	rs17681451	G	0.93	0.023	0.003	3.93E-13	0.008	0.014	5.00E-01
BMI	rs1916801	A	0.62	0.017	0.002	2.76E-23	0.005	0.008	5.00E-01
BMI	rs28350	A	0.19	0.017	0.002	5.36E-15	0.003	0.010	7.00E-01
BMI	rs355777	C	0.42	0.015	0.002	6.55E-19	0.010	0.008	2.50E-01
BMI	rs39654	G	0.54	0.016	0.002	8.96E-22	0.006	0.008	4.90E-01
BMI	rs4857968	G	0.76	0.013	0.002	3.80E-12	0.016	0.009	5.60E-02
BMI	rs59302296	A	0.09	0.022	0.003	1.19E-11	0.002	0.013	9.40E-01
BMI	rs6443750	C	0.82	0.015	0.002	4.55E-13	0.011	0.010	2.70E-01
BMI	rs6804181	A	0.82	0.014	0.002	8.76E-10	0.002	0.010	6.90E-01
BMI	rs6804842	G	0.56	0.014	0.002	1.22E-18	-0.017	0.008	4.20E-02
BMI	rs7616009	G	0.85	0.016	0.002	6.08E-11	0.018	0.011	9.70E-02
BMI	rs7631156	A	0.30	0.022	0.002	6.94E-33	0.012	0.008	1.60E-01
BMI	rs9816226	T	0.81	0.032	0.002	7.34E-51	-0.021	0.010	1.90E-02
BMI	rs9818122	C	0.22	0.023	0.002	4.18E-30	-0.004	0.010	6.40E-01
BMI	rs9826775	A	0.87	0.016	0.002	1.06E-10	-0.003	0.011	7.80E-01
BMI	rs9862795	T	0.51	0.026	0.002	6.82E-44	0.020	0.008	8.10E-03
BMI	rs10938397	G	0.42	0.032	0.002	4.46E-90	0.016	0.008	5.40E-02
BMI	rs12509234	C	0.28	0.012	0.002	1.03E-09	-0.004	0.008	6.40E-01
BMI	rs13107325	T	0.08	0.047	0.003	1.95E-48	0.011	0.015	4.00E-01
BMI	rs13110266	G	0.59	0.012	0.002	9.19E-15	0.013	0.008	1.40E-01
BMI	rs1346841	G	0.58	0.013	0.002	1.25E-13	-0.004	0.008	6.40E-01
BMI	rs1437842	G	0.52	0.011	0.002	4.51E-10	-0.004	0.008	6.10E-01
BMI	rs1451109	G	0.32	0.016	0.002	1.48E-20	0.015	0.008	7.90E-02
BMI	rs1481012	A	0.91	0.019	0.003	4.80E-13	0.006	0.012	7.20E-01
BMI	rs1522569	T	0.83	0.014	0.002	1.46E-10	-0.004	0.010	6.70E-01
BMI	rs17001561	A	0.18	0.015	0.002	2.89E-10	-0.004	0.011	7.50E-01
BMI	rs17276464	T	0.43	0.010	0.002	1.97E-09	-0.003	0.008	8.10E-01
BMI	rs2051559	C	0.12	0.017	0.003	2.39E-11	-0.002	0.012	9.70E-01
BMI	rs2192158	A	0.47	0.014	0.002	7.70E-16	0.006	0.008	4.00E-01
BMI	rs2391540	T	0.33	0.014	0.002	1.14E-14	0.006	0.008	4.30E-01
BMI	rs34811474	G	0.78	0.029	0.002	3.58E-37	0.011	0.009	2.70E-01
BMI	rs35851183	G	0.37	0.012	0.002	1.45E-09	0.008	0.008	3.90E-01
BMI	rs4834272	C	0.31	0.011	0.002	2.11E-10	0.000	0.008	9.30E-01
BMI	rs57800857	A	0.66	0.016	0.002	4.16E-15	0.013	0.008	9.50E-02
BMI	rs6850421	A	0.49	0.011	0.002	2.72E-09	0.010	0.008	1.70E-01
BMI	rs7678054	G	0.55	0.010	0.002	5.76E-09	-0.001	0.008	9.90E-01
BMI	rs10044136	G	0.44	0.016	0.002	1.58E-21	0.011	0.008	2.20E-01
BMI	rs10942267	A	0.72	0.015	0.002	2.00E-16	0.000	0.008	9.80E-01
BMI	rs11739877	T	0.61	0.012	0.002	1.16E-10	0.008	0.008	3.90E-01
BMI	rs13174863	G	0.17	0.020	0.002	1.08E-17	-0.004	0.011	6.90E-01
BMI	rs1501673	A	0.14	0.029	0.003	6.57E-31	0.009	0.012	4.80E-01
BMI	rs2053682	A	0.68	0.017	0.002	3.57E-21	0.012	0.008	1.70E-01
BMI	rs2112347	T	0.63	0.028	0.002	2.84E-59	0.016	0.008	3.60E-02
BMI	rs2861089	A	0.38	0.011	0.002	6.56E-10	0.001	0.008	9.10E-01
BMI	rs288230	T	0.83	0.024	0.002	4.28E-25	0.008	0.010	3.80E-01

BMI	rs459552	T	0.23	0.013	0.002	2.56E-12	0.014	0.009	1.10E-01
BMI	rs4700608	C	0.52	0.016	0.002	7.68E-20	-0.011	0.008	1.90E-01
BMI	rs6451675	G	0.66	0.014	0.002	7.38E-15	0.002	0.008	8.30E-01
BMI	rs6556301	G	0.67	0.011	0.002	2.99E-11	0.010	0.008	2.30E-01
BMI	rs6864049	G	0.53	0.012	0.002	3.95E-14	0.001	0.008	8.30E-01
BMI	rs6890310	G	0.73	0.012	0.002	3.77E-10	0.012	0.009	2.80E-01
BMI	rs698147	A	0.50	0.012	0.002	8.88E-12	0.000	0.008	8.90E-01
BMI	rs7713317	G	0.28	0.017	0.002	2.91E-20	0.005	0.009	5.00E-01
BMI	rs7734385	G	0.55	0.010	0.002	2.75E-10	0.000	0.008	9.60E-01
BMI	rs11757278	T	0.68	0.013	0.002	2.56E-12	0.019	0.008	3.30E-02
BMI	rs12206094	C	0.68	0.014	0.002	1.95E-15	-0.014	0.009	1.10E-01
BMI	rs1269175	A	0.51	0.011	0.002	6.56E-10	-0.002	0.008	7.90E-01
BMI	rs13191362	A	0.87	0.024	0.003	5.46E-21	0.008	0.012	4.80E-01
BMI	rs2206277	T	0.18	0.041	0.002	4.43E-84	-0.008	0.010	4.00E-01
BMI	rs2228213	G	0.67	0.014	0.002	2.44E-17	0.011	0.008	1.10E-01
BMI	rs2246012	C	0.17	0.016	0.002	2.51E-13	0.004	0.010	8.10E-01
BMI	rs2357760	A	0.67	0.014	0.002	4.04E-17	0.017	0.008	2.80E-02
BMI	rs2622274	G	0.58	0.011	0.002	3.09E-10	0.017	0.008	6.10E-02
BMI	rs3806114	G	0.29	0.012	0.002	2.62E-11	0.012	0.008	1.10E-01
BMI	rs487152	A	0.48	0.011	0.002	1.48E-11	-0.004	0.008	6.90E-01
BMI	rs6922607	G	0.22	0.013	0.002	3.44E-09	-0.021	0.010	2.40E-02
BMI	rs6932930	G	0.20	0.026	0.002	1.09E-41	-0.010	0.009	3.90E-01
BMI	rs765875	C	0.50	0.013	0.002	8.18E-15	0.003	0.008	5.30E-01
BMI	rs7769594	T	0.15	0.016	0.002	1.00E-12	0.003	0.011	7.90E-01
BMI	rs816367	G	0.35	0.011	0.002	3.09E-10	0.001	0.008	8.70E-01
BMI	rs9320823	C	0.59	0.017	0.002	2.85E-22	0.022	0.008	3.80E-03
BMI	rs1030015	T	0.53	0.011	0.002	1.44E-10	-0.004	0.008	6.10E-01
BMI	rs1048303	C	0.44	0.011	0.002	9.76E-11	0.017	0.008	3.50E-02
BMI	rs10499694	A	0.52	0.013	0.002	4.47E-16	-0.008	0.008	3.10E-01
BMI	rs11525873	T	0.90	0.023	0.003	4.17E-13	0.007	0.013	6.60E-01
BMI	rs1167821	T	0.42	0.020	0.002	3.73E-26	0.019	0.008	6.50E-03
BMI	rs1852006	G	0.65	0.015	0.002	1.90E-17	0.013	0.008	9.20E-02
BMI	rs1899689	T	0.38	0.012	0.002	1.68E-12	0.000	0.008	8.90E-01
BMI	rs215614	G	0.34	0.014	0.002	1.24E-15	0.019	0.008	3.30E-02
BMI	rs2396625	T	0.60	0.018	0.002	4.06E-25	0.003	0.008	6.50E-01
BMI	rs2907948	G	0.75	0.015	0.002	2.32E-14	0.007	0.009	5.00E-01
BMI	rs4307239	G	0.48	0.012	0.002	1.34E-11	0.002	0.008	8.40E-01
BMI	rs4721089	T	0.76	0.017	0.002	3.85E-13	-0.004	0.009	7.90E-01
BMI	rs6463489	T	0.07	0.017	0.003	1.34E-10	0.016	0.012	1.90E-01
BMI	rs6962280	G	0.51	0.014	0.002	1.73E-13	0.011	0.008	2.50E-01
BMI	rs740157	A	0.43	0.012	0.002	1.64E-13	0.011	0.008	1.10E-01
BMI	rs7777084	A	0.45	0.013	0.002	1.30E-14	-0.001	0.008	8.50E-01
BMI	rs7802342	G	0.27	0.012	0.002	6.74E-11	0.004	0.009	6.00E-01
BMI	rs10099330	G	0.44	0.012	0.002	2.56E-12	0.007	0.008	2.90E-01
BMI	rs10954772	T	0.30	0.016	0.002	1.57E-13	0.004	0.008	5.60E-01
BMI	rs1106761	A	0.35	0.013	0.002	2.08E-11	-0.002	0.008	7.80E-01
BMI	rs12680842	A	0.62	0.014	0.002	6.66E-17	0.012	0.008	1.30E-01
BMI	rs12681792	A	0.19	0.015	0.002	9.14E-13	0.007	0.010	5.70E-01
BMI	rs1383592	A	0.23	0.012	0.002	6.27E-09	0.022	0.010	4.00E-02
BMI	rs1431659	A	0.28	0.019	0.002	8.94E-24	0.001	0.009	7.80E-01
BMI	rs1658820	T	0.25	0.013	0.002	4.11E-11	0.003	0.009	7.90E-01
BMI	rs17405819	T	0.70	0.021	0.002	9.81E-32	0.023	0.008	7.20E-03
BMI	rs1982441	T	0.14	0.017	0.003	1.05E-11	0.018	0.011	9.50E-02
BMI	rs2196618	G	0.75	0.014	0.002	5.57E-13	0.007	0.009	3.10E-01
BMI	rs354508	C	0.84	0.015	0.002	2.18E-10	0.005	0.011	5.90E-01
BMI	rs36061954	T	0.38	0.012	0.002	3.77E-10	0.003	0.008	8.60E-01

BMI	rs3808477	C	0.76	0.018	0.002	9.80E-22	0.002	0.009	6.70E-01
BMI	rs4123853	T	0.37	0.014	0.002	1.79E-16	0.009	0.008	2.50E-01
BMI	rs4841504	C	0.50	0.017	0.002	3.22E-25	0.004	0.008	4.90E-01
BMI	rs72673947	G	0.12	0.022	0.003	6.31E-13	0.021	0.013	1.20E-01
BMI	rs7826312	C	0.59	0.011	0.002	9.59E-12	0.001	0.008	5.90E-01
BMI	rs10116186	G	0.52	0.012	0.002	1.03E-09	-0.007	0.008	4.20E-01
BMI	rs10761247	G	0.43	0.011	0.002	2.99E-11	0.000	0.008	8.90E-01
BMI	rs10858334	G	0.13	0.015	0.003	3.22E-09	-0.021	0.011	8.70E-02
BMI	rs10984756	G	0.09	0.018	0.003	3.26E-10	0.011	0.013	3.10E-01
BMI	rs1928295	T	0.56	0.013	0.002	5.52E-17	-0.012	0.008	1.30E-01
BMI	rs2134858	C	0.46	0.012	0.002	5.89E-12	0.007	0.008	4.30E-01
BMI	rs2183824	T	0.31	0.023	0.002	4.16E-43	0.007	0.008	4.90E-01
BMI	rs2777768	A	0.74	0.012	0.002	3.77E-10	0.002	0.009	6.40E-01
BMI	rs3739514	A	0.35	0.013	0.002	4.11E-11	0.014	0.008	5.10E-02
BMI	rs3811125	C	0.73	0.015	0.002	4.55E-13	0.004	0.009	5.00E-01
BMI	rs4740619	T	0.52	0.019	0.002	3.36E-32	0.012	0.008	1.20E-01
BMI	rs6476617	G	0.64	0.015	0.002	1.87E-18	0.036	0.008	6.50E-06
BMI	rs6477694	C	0.33	0.013	0.002	1.25E-13	0.009	0.008	3.60E-01
BMI	rs7024334	T	0.23	0.014	0.002	1.48E-11	0.002	0.009	7.50E-01
BMI	rs7037266	C	0.65	0.011	0.002	3.89E-09	-0.002	0.008	9.60E-01
BMI	rs7357754	G	0.52	0.012	0.002	1.68E-12	0.007	0.008	2.60E-01
BMI	rs867560	G	0.46	0.014	0.002	1.24E-15	-0.005	0.008	5.40E-01
BMI	rs10761785	G	0.49	0.013	0.002	9.37E-17	-0.009	0.008	3.30E-01
BMI	rs10823893	A	0.39	0.012	0.002	6.74E-11	-0.001	0.008	9.10E-01
BMI	rs10887584	A	0.45	0.013	0.002	3.32E-11	0.004	0.008	6.30E-01
BMI	rs12098284	T	0.12	0.018	0.003	1.47E-12	0.013	0.012	2.20E-01
BMI	rs12765914	T	0.08	0.023	0.003	3.09E-13	0.014	0.014	4.10E-01
BMI	rs1277733	T	0.78	0.012	0.002	1.97E-09	0.008	0.009	5.30E-01
BMI	rs17636031	C	0.27	0.015	0.002	1.17E-17	-0.001	0.009	8.10E-01
BMI	rs2439823	G	0.55	0.017	0.002	2.85E-22	0.012	0.008	6.90E-02
BMI	rs34277166	A	0.45	0.014	0.002	3.48E-14	0.009	0.008	3.30E-01
BMI	rs4097319	T	0.58	0.011	0.002	3.09E-10	0.001	0.008	9.50E-01
BMI	rs4290163	T	0.41	0.013	0.002	2.06E-14	-0.011	0.008	2.40E-01
BMI	rs4880341	C	0.47	0.013	0.002	2.06E-14	0.003	0.008	8.10E-01
BMI	rs7070670	C	0.68	0.013	0.002	1.97E-09	0.008	0.008	3.10E-01
BMI	rs7084454	A	0.34	0.020	0.002	3.82E-28	0.013	0.008	1.70E-01
BMI	rs7893571	T	0.67	0.013	0.002	3.80E-12	0.001	0.008	7.90E-01
BMI	rs7899106	G	0.04	0.033	0.004	9.76E-19	0.040	0.018	3.70E-02
BMI	rs7903146	C	0.68	0.018	0.002	4.65E-23	-0.010	0.009	2.10E-01
BMI	rs845084	A	0.25	0.014	0.002	8.19E-13	0.004	0.009	7.20E-01
BMI	rs11218510	G	0.63	0.014	0.002	2.56E-12	0.004	0.008	4.80E-01
BMI	rs12282785	C	0.75	0.016	0.002	8.73E-12	0.006	0.010	3.70E-01
BMI	rs12286929	G	0.48	0.018	0.002	1.91E-28	0.005	0.008	4.80E-01
BMI	rs12364470	G	0.16	0.019	0.002	1.90E-17	0.019	0.010	6.90E-02
BMI	rs1982350	G	0.65	0.016	0.002	4.45E-18	0.006	0.008	5.00E-01
BMI	rs2065418	T	0.65	0.014	0.002	1.14E-14	0.008	0.008	3.10E-01
BMI	rs2605603	G	0.50	0.010	0.002	1.21E-10	0.001	0.008	8.10E-01
BMI	rs2862996	G	0.34	0.022	0.002	5.48E-37	0.019	0.008	1.20E-02
BMI	rs349088	C	0.48	0.013	0.002	2.06E-14	0.002	0.008	4.90E-01
BMI	rs3825061	T	0.35	0.014	0.002	1.79E-16	0.007	0.008	3.00E-01
BMI	rs4256980	G	0.63	0.019	0.002	3.82E-28	0.022	0.008	4.60E-03
BMI	rs5215	T	0.65	0.011	0.002	2.99E-11	0.000	0.008	9.50E-01
BMI	rs592483	C	0.42	0.014	0.002	7.70E-16	0.004	0.008	6.10E-01
BMI	rs6265	C	0.80	0.041	0.002	4.16E-86	0.034	0.010	1.40E-03
BMI	rs6591407	C	0.81	0.012	0.002	3.53E-09	0.012	0.010	2.70E-01
BMI	rs7124681	A	0.42	0.026	0.002	4.67E-58	0.000	0.008	9.70E-01

BMI	rs76942203	A	0.06	0.026	0.004	1.41E-10	0.004	0.017	7.50E-01
BMI	rs7944782	G	0.51	0.014	0.002	2.44E-17	0.024	0.008	3.30E-03
BMI	rs10744146	G	0.44	0.011	0.002	2.99E-11	0.003	0.008	7.30E-01
BMI	rs11047132	G	0.09	0.023	0.003	2.43E-13	0.018	0.014	2.20E-01
BMI	rs11105839	T	0.65	0.011	0.002	2.99E-11	0.000	0.008	7.20E-01
BMI	rs11115176	T	0.78	0.013	0.002	5.40E-12	0.011	0.009	2.30E-01
BMI	rs11170468	A	0.77	0.013	0.002	7.80E-12	0.002	0.009	7.60E-01
BMI	rs11611246	T	0.18	0.022	0.002	7.16E-29	0.011	0.009	1.80E-01
BMI	rs11836108	A	0.28	0.011	0.002	3.43E-10	0.001	0.008	9.80E-01
BMI	rs12321904	T	0.53	0.010	0.002	1.97E-09	0.008	0.008	4.40E-01
BMI	rs12369179	C	0.93	0.034	0.003	5.46E-28	0.003	0.013	7.40E-01
BMI	rs12422552	G	0.71	0.013	0.002	5.40E-12	0.005	0.009	4.60E-01
BMI	rs650198	C	0.31	0.014	0.002	5.57E-13	-0.001	0.009	5.80E-01
BMI	rs6539064	C	0.72	0.019	0.002	1.78E-24	0.007	0.009	4.10E-01
BMI	rs704061	C	0.44	0.014	0.002	6.66E-17	-0.004	0.008	7.60E-01
BMI	rs7138803	A	0.34	0.030	0.002	2.40E-68	0.001	0.008	8.30E-01
BMI	rs7975187	G	0.22	0.014	0.002	6.85E-11	0.019	0.009	2.40E-02
BMI	rs1045411	C	0.73	0.014	0.002	2.56E-13	0.023	0.009	5.50E-03
BMI	rs12429545	A	0.11	0.031	0.002	7.09E-39	0.002	0.012	8.20E-01
BMI	rs12868881	A	0.43	0.014	0.002	4.75E-16	0.010	0.008	1.80E-01
BMI	rs1536053	C	0.69	0.012	0.002	1.35E-10	-0.001	0.008	9.20E-01
BMI	rs1927790	C	0.45	0.014	0.002	2.13E-18	0.023	0.008	2.90E-03
BMI	rs41284828	G	0.96	0.034	0.006	2.20E-09	-0.028	0.023	1.60E-01
BMI	rs4421883	C	0.55	0.010	0.002	1.37E-09	0.002	0.008	8.20E-01
BMI	rs77432547	G	0.28	0.017	0.002	5.72E-16	0.005	0.009	5.90E-01
BMI	rs8181823	C	0.78	0.013	0.002	4.10E-10	0.005	0.009	7.00E-01
BMI	rs9540493	A	0.47	0.013	0.002	3.24E-14	-0.013	0.008	8.60E-02
BMI	rs9595908	T	0.59	0.015	0.002	1.32E-19	0.009	0.008	3.00E-01
BMI	rs9603697	T	0.35	0.013	0.002	9.74E-14	-0.001	0.008	7.30E-01
BMI	rs10132280	C	0.68	0.021	0.002	1.35E-32	-0.002	0.008	6.30E-01
BMI	rs1491905	T	0.48	0.015	0.002	5.28E-18	0.013	0.008	1.30E-01
BMI	rs1951455	C	0.74	0.015	0.002	6.73E-15	-0.019	0.009	2.50E-02
BMI	rs1954494	T	0.54	0.010	0.002	1.34E-09	-0.001	0.008	8.60E-01
BMI	rs217669	C	0.25	0.017	0.002	2.60E-16	0.023	0.009	8.60E-03
BMI	rs4906263	G	0.33	0.018	0.002	1.40E-22	0.015	0.008	4.10E-02
BMI	rs709400	A	0.61	0.015	0.002	3.15E-18	0.013	0.008	7.80E-02
BMI	rs7144011	T	0.21	0.026	0.002	1.70E-39	0.000	0.009	9.80E-01
BMI	rs872281	C	0.83	0.015	0.002	3.88E-11	0.005	0.010	4.60E-01
BMI	rs942066	G	0.62	0.020	0.002	5.52E-24	0.022	0.008	1.00E-02
BMI	rs11633626	C	0.38	0.016	0.002	2.73E-18	0.008	0.008	3.20E-01
BMI	rs11636611	T	0.51	0.010	0.002	9.50E-10	0.013	0.008	1.10E-01
BMI	rs11856579	G	0.78	0.016	0.002	2.38E-17	0.012	0.009	1.80E-01
BMI	rs12438629	C	0.97	0.035	0.005	2.50E-11	0.003	0.027	8.90E-01
BMI	rs1471212	A	0.47	0.011	0.002	1.44E-10	0.010	0.008	1.80E-01
BMI	rs1996120	G	0.59	0.017	0.002	1.67E-25	0.000	0.008	1.00E+00
BMI	rs2715423	G	0.77	0.012	0.002	1.42E-09	-0.011	0.009	2.00E-01
BMI	rs6493498	T	0.46	0.014	0.002	1.10E-17	0.005	0.008	6.10E-01
BMI	rs7181610	A	0.89	0.015	0.003	5.22E-09	0.025	0.011	1.40E-02
BMI	rs8024806	T	0.94	0.025	0.004	3.95E-13	0.022	0.016	1.80E-01
BMI	rs11075489	C	0.53	0.012	0.002	8.88E-12	0.010	0.008	1.90E-01
BMI	rs11866815	C	0.76	0.015	0.002	8.10E-16	0.013	0.009	1.60E-01
BMI	rs12449219	G	0.14	0.016	0.003	2.83E-10	0.011	0.012	3.40E-01
BMI	rs12922346	C	0.23	0.013	0.002	2.93E-11	0.018	0.009	4.10E-02
BMI	rs194809	A	0.18	0.013	0.002	1.02E-08	0.012	0.010	2.30E-01
BMI	rs2342892	T	0.45	0.013	0.002	1.25E-13	-0.002	0.008	8.70E-01
BMI	rs2601777	G	0.62	0.014	0.002	4.04E-17	-0.003	0.008	7.30E-01

BMI	rs4609871	T	0.51	0.022	0.002	5.66E-38	-0.002	0.008	8.60E-01
BMI	rs7206608	G	0.31	0.013	0.002	5.11E-13	0.016	0.008	4.00E-02
BMI	rs7498665	G	0.33	0.029	0.002	4.42E-63	0.016	0.008	3.90E-02
BMI	rs889398	C	0.58	0.020	0.002	3.62E-34	0.016	0.008	4.90E-02
BMI	rs9937053	A	0.44	0.072	0.002	0.00E+00	0.021	0.008	7.50E-03
BMI	rs1106908	G	0.55	0.016	0.002	8.09E-24	-0.002	0.008	7.10E-01
BMI	rs11655587	C	0.68	0.021	0.002	8.64E-26	0.001	0.008	9.20E-01
BMI	rs12939549	A	0.57	0.018	0.002	2.32E-29	-0.002	0.008	7.40E-01
BMI	rs1320251	C	0.51	0.018	0.002	2.19E-25	0.008	0.008	1.80E-01
BMI	rs2619976	T	0.38	0.011	0.002	5.43E-09	0.004	0.008	5.10E-01
BMI	rs3744017	A	0.20	0.014	0.002	2.65E-10	0.000	0.010	8.00E-01
BMI	rs3923783	C	0.82	0.022	0.002	6.06E-24	0.023	0.010	2.10E-02
BMI	rs3930349	C	0.77	0.014	0.002	2.56E-12	-0.002	0.009	7.40E-01
BMI	rs6607337	C	0.70	0.012	0.002	6.74E-11	0.020	0.009	1.30E-02
BMI	rs8075273	C	0.74	0.014	0.002	2.72E-14	-0.002	0.009	9.10E-01
BMI	rs1356506	T	0.64	0.014	0.002	2.72E-14	0.020	0.008	1.10E-02
BMI	rs594821	C	0.93	0.019	0.003	1.93E-10	0.006	0.014	7.70E-01
BMI	rs6567160	C	0.24	0.055	0.002	1.43E-185	-0.004	0.009	8.60E-01
BMI	rs7238896	G	0.14	0.022	0.003	6.93E-15	-0.003	0.011	8.10E-01
BMI	rs7239114	A	0.53	0.012	0.002	1.68E-12	0.008	0.008	2.20E-01
BMI	rs891387	T	0.53	0.021	0.002	2.01E-34	0.007	0.008	4.30E-01
BMI	rs10518269	C	0.82	0.017	0.002	5.41E-14	0.008	0.010	4.20E-01
BMI	rs11672660	C	0.79	0.034	0.002	2.76E-58	-0.015	0.010	1.00E-01
BMI	rs12151152	G	0.59	0.021	0.002	8.64E-26	0.003	0.008	7.60E-01
BMI	rs12462975	A	0.29	0.019	0.002	8.01E-27	-0.011	0.008	2.60E-01
BMI	rs273505	C	0.45	0.017	0.002	8.61E-20	0.035	0.008	1.20E-05
BMI	rs56356382	T	0.81	0.022	0.002	1.54E-19	0.018	0.010	7.70E-02
BMI	rs1409818	T	0.10	0.020	0.003	3.30E-12	0.024	0.013	1.00E-01
BMI	rs16989232	A	0.35	0.012	0.002	1.34E-11	0.000	0.008	8.70E-01
BMI	rs17201143	C	0.69	0.011	0.002	4.90E-10	0.004	0.008	5.90E-01
BMI	rs17806224	G	0.82	0.026	0.002	3.14E-32	0.031	0.010	2.00E-03
BMI	rs1884389	C	0.57	0.011	0.002	2.11E-10	-0.010	0.008	2.10E-01
BMI	rs1884897	G	0.61	0.018	0.002	2.66E-27	-0.007	0.008	3.20E-01
BMI	rs615568	G	0.49	0.010	0.002	2.83E-09	0.009	0.008	2.80E-01
BMI	rs6512302	C	0.74	0.013	0.002	2.08E-11	0.017	0.009	1.00E-01
BMI	rs8122855	A	0.35	0.014	0.002	2.72E-14	0.028	0.008	2.60E-04
BMI	rs140733155	G	0.01	0.056	0.009	2.73E-09	0.020	0.038	5.40E-01
BMI	rs2832283	A	0.27	0.012	0.002	8.92E-09	-0.019	0.009	3.50E-02
BMI	rs427943	C	0.56	0.018	0.002	2.19E-25	0.012	0.008	1.00E-01
BMI	rs8134638	C	0.41	0.013	0.002	2.93E-11	0.003	0.008	7.90E-01
BMI	rs12628051	T	0.68	0.016	0.002	3.74E-19	-0.009	0.008	2.80E-01
BMI	rs12628891	C	0.71	0.012	0.002	1.42E-09	-0.004	0.008	6.50E-01
Caffeine	rs115454798	A	0.87	-0.023	0.003	1.10E-10	0.023	0.012	6.40E-02
Caffeine	rs117810762	G	0.98	-0.068	0.009	1.30E-14	0.031	0.030	2.60E-01
Caffeine	rs1228024	C	0.34	0.014	0.002	1.30E-08	-0.002	0.008	7.90E-01
Caffeine	rs12514566	G	0.66	0.017	0.002	2.20E-12	0.012	0.008	1.20E-01
Caffeine	rs12591786	C	0.84	0.020	0.003	3.90E-10	0.002	0.011	9.90E-01
Caffeine	rs1260326	T	0.39	-0.023	0.002	4.20E-22	0.039	0.008	4.10E-07
Caffeine	rs12785227	A	0.69	0.015	0.003	6.50E-09	-0.001	0.008	8.10E-01
Caffeine	rs1490384	C	0.50	-0.016	0.002	8.30E-12	-0.006	0.008	5.60E-01
Caffeine	rs17685	G	0.72	-0.041	0.003	3.80E-56	-0.005	0.009	4.70E-01
Caffeine	rs215601	A	0.37	0.015	0.002	6.00E-10	0.018	0.008	4.50E-02
Caffeine	rs2231142	G	0.89	0.039	0.004	1.10E-26	0.008	0.012	6.20E-01
Caffeine	rs2472297	C	0.73	-0.105	0.003	1.00E-200	-0.006	0.009	5.40E-01
Caffeine	rs2667773	A	0.69	0.015	0.003	2.50E-09	0.010	0.008	2.20E-01
Caffeine	rs4240624	G	0.09	0.027	0.004	1.90E-11	-0.022	0.014	1.00E-01

Caffeine	rs4410790	C	0.62	0.150	0.017	2.36E-19	0.005	0.008	3.50E-01
Caffeine	rs4418728	G	0.55	0.014	0.002	7.00E-10	0.014	0.008	6.20E-02
Caffeine	rs489693	C	0.67	-0.018	0.002	1.30E-12	-0.003	0.008	5.70E-01
Caffeine	rs56113850	T	0.42	-0.026	0.002	1.90E-29	-0.012	0.008	1.40E-01
Caffeine	rs6062679	T	0.53	-0.023	0.002	4.50E-22	0.002	0.008	9.90E-01
Caffeine	rs62332762	C	0.60	0.018	0.002	1.20E-14	0.000	0.008	9.50E-01
Caffeine	rs6265	C	0.81	0.022	0.003	3.40E-13	0.034	0.010	1.40E-03
Caffeine	rs7105462	G	0.41	0.014	0.002	2.10E-09	0.000	0.008	8.60E-01
Caffeine	rs78456557	C	0.90	-0.024	0.004	1.20E-09	-0.020	0.013	1.70E-01
Caffeine	rs9611527	G	0.66	0.020	0.002	1.00E-15	0.012	0.008	1.10E-01
Caffeine_coffee	rs1057868	C	0.71	-0.024	0.003	5.50E-22	-0.006	0.009	4.10E-01
Caffeine_coffee	rs11127048	G	0.38	-0.020	0.002	8.10E-17	0.036	0.008	3.80E-06
Caffeine_coffee	rs117810762	G	0.98	-0.050	0.009	1.10E-08	0.031	0.030	2.60E-01
Caffeine_coffee	rs12514566	G	0.66	0.015	0.002	1.00E-09	0.012	0.008	1.20E-01
Caffeine_coffee	rs1327259	A	0.61	0.016	0.002	3.10E-11	0.001	0.008	9.80E-01
Caffeine_coffee	rs181251778	A	0.99	0.073	0.010	1.50E-13	0.086	0.033	7.00E-03
Caffeine_coffee	rs2298527	G	0.41	0.015	0.002	1.60E-10	-0.001	0.008	8.30E-01
Caffeine_coffee	rs2472297	C	0.73	-0.065	0.003	3.10E-140	-0.006	0.009	5.40E-01
Caffeine_coffee	rs2521501	A	0.68	0.015	0.002	1.60E-09	0.010	0.008	2.50E-01
Caffeine_coffee	rs2726513	G	0.59	0.015	0.002	7.00E-11	-0.001	0.008	7.80E-01
Caffeine_coffee	rs34060476	A	0.87	-0.025	0.003	7.10E-14	-0.013	0.011	2.30E-01
Caffeine_coffee	rs35198275	A	0.87	0.021	0.003	8.20E-10	-0.017	0.011	1.30E-01
Caffeine_coffee	rs4410790	T	0.36	-0.052	0.002	2.80E-104	-0.005	0.008	3.50E-01
Caffeine_coffee	rs4615895	G	0.26	-0.017	0.003	8.60E-11	-0.001	0.009	9.00E-01
Caffeine_coffee	rs56113850	T	0.42	-0.021	0.002	4.90E-19	-0.012	0.008	1.40E-01
Caffeine_coffee	rs6063085	A	0.63	-0.016	0.002	3.40E-11	-0.024	0.008	1.90E-03
Caffeine_coffee	rs66723169	C	0.77	-0.022	0.003	1.80E-16	0.008	0.009	5.70E-01
Caffeine_coffee	rs6893807	A	0.84	-0.019	0.003	1.30E-09	-0.022	0.011	4.90E-02
Caffeine_coffee	rs7571970	T	0.17	-0.021	0.003	9.40E-12	0.005	0.010	6.30E-01
Caffeine_coffee	rs76881016	A	0.93	-0.026	0.004	6.60E-09	-0.026	0.015	9.60E-02
Caffeine_tea	rs10741694	T	0.37	-0.015	0.002	1.80E-10	-0.007	0.008	5.10E-01
Caffeine_tea	rs11022752	A	0.73	-0.015	0.003	1.20E-09	-0.004	0.009	6.00E-01
Caffeine_tea	rs11204711	A	0.62	-0.014	0.002	6.40E-10	0.011	0.008	1.90E-01
Caffeine_tea	rs12591786	C	0.84	0.020	0.003	3.00E-10	0.002	0.011	9.90E-01
Caffeine_tea	rs132919	G	0.23	-0.017	0.003	2.40E-10	-0.007	0.009	3.90E-01
Caffeine_tea	rs140775622	C	0.83	-0.023	0.003	1.80E-12	0.002	0.011	9.70E-01
Caffeine_tea	rs1481012	A	0.89	0.026	0.004	1.90E-13	0.006	0.012	7.20E-01
Caffeine_tea	rs17685	G	0.72	-0.024	0.002	7.20E-22	-0.005	0.009	4.70E-01
Caffeine_tea	rs192084998	G	0.70	0.016	0.002	1.20E-10	0.006	0.008	3.70E-01
Caffeine_tea	rs2117137	A	0.59	-0.013	0.002	2.90E-09	0.000	0.008	9.60E-01
Caffeine_tea	rs2465018	G	0.77	-0.022	0.003	1.10E-16	-0.001	0.009	7.80E-01
Caffeine_tea	rs2472297	C	0.73	-0.054	0.003	3.30E-102	-0.006	0.009	5.40E-01
Caffeine_tea	rs28429148	G	0.57	0.013	0.002	8.20E-09	-0.020	0.008	1.30E-02
Caffeine_tea	rs4410790	T	0.37	-0.040	0.002	1.10E-67	-0.005	0.008	3.50E-01
Caffeine_tea	rs4817505	T	0.61	-0.015	0.002	4.20E-11	0.009	0.008	1.80E-01
Caffeine_tea	rs56188862	T	0.61	0.017	0.002	2.40E-13	-0.007	0.008	3.80E-01
Caffeine_tea	rs62534435	C	0.80	-0.016	0.003	4.40E-09	-0.003	0.010	7.30E-01
Caffeine_tea	rs73053413	C	0.84	0.022	0.003	7.10E-13	0.007	0.010	5.10E-01
Caffeine_tea	rs9624470	G	0.42	-0.025	0.002	6.80E-29	-0.004	0.008	6.30E-01
Coffee	rs574367	T	0.21	0.021	0.004	5.43E-09	0.001	0.010	8.70E-01
Coffee	rs10865548	G	0.83	0.031	0.004	5.26E-16	-0.004	0.010	6.80E-01
Coffee	rs1260326	C	0.61	0.027	0.003	1.23E-19	-0.039	0.008	4.10E-07
Coffee	rs1057868	T	0.29	0.039	0.003	7.76E-35	0.006	0.009	4.10E-01
Coffee	rs34060476	G	0.13	0.038	0.004	8.63E-18	0.013	0.011	2.30E-01
Coffee	rs4410790	C	0.63	0.079	0.003	4.61E-152	0.005	0.008	3.50E-01
Coffee	rs73073176	C	0.87	0.046	0.004	8.64E-26	0.011	0.011	3.80E-01

Coffee	rs597045	A	0.69	0.021	0.003	2.27E-11	-0.006	0.009	3.60E-01
Coffee	rs1956218	G	0.56	0.016	0.003	4.59E-08	-0.012	0.008	1.30E-01
Coffee	rs2472297	T	0.27	0.091	0.003	4.02E-157	0.006	0.009	5.40E-01
Coffee	rs66723169	A	0.23	0.029	0.004	3.17E-16	-0.008	0.009	5.70E-01
Coffee	rs2330783	G	0.99	0.091	0.013	6.46E-13	0.087	0.033	8.10E-03
CigPDay	rs11725618	C	0.31	0.036	0.006	4.72E-09	0.002	0.009	7.80E-01
CigPDay	rs11852372	C	0.36	0.182	0.006	2.96E-209	-0.010	0.008	1.40E-01
CigPDay	rs13141210	T	0.50	-0.031	0.006	2.72E-08	0.000	0.008	6.20E-01
CigPDay	rs2072659	G	0.10	-0.065	0.009	1.70E-12	0.018	0.013	2.20E-01
CigPDay	rs2084533	T	0.31	0.034	0.006	1.19E-08	-0.012	0.008	1.10E-01
CigPDay	rs215600	A	0.67	-0.049	0.006	1.11E-17	-0.021	0.008	2.00E-02
CigPDay	rs2273500	C	0.15	0.068	0.008	2.44E-18	0.004	0.011	6.80E-01
CigPDay	rs2386571	C	0.58	-0.032	0.006	1.02E-08	-0.011	0.008	1.40E-01
CigPDay	rs2424888	A	0.36	0.033	0.006	2.83E-09	-0.007	0.008	4.30E-01
CigPDay	rs3025383	C	0.18	-0.058	0.007	2.22E-16	-0.003	0.010	9.30E-01
CigPDay	rs4785587	A	0.48	-0.034	0.006	1.24E-09	-0.006	0.008	4.40E-01
CigPDay	rs56113850	C	0.59	0.107	0.006	1.39E-81	0.012	0.008	1.40E-01
CigPDay	rs58379124	C	0.77	0.067	0.007	7.37E-25	0.014	0.009	1.90E-01
CigPDay	rs632811	G	0.35	-0.037	0.006	1.02E-08	0.015	0.008	5.00E-02
CigPDay	rs7431710	A	0.66	-0.035	0.006	1.78E-09	0.004	0.008	5.20E-01
CigPDay	rs76164573	G	0.05	-0.072	0.013	3.70E-08	-0.012	0.017	5.50E-01
CigPDay	rs790564	C	0.73	-0.041	0.006	4.03E-11	-0.014	0.009	1.30E-01
CigPDay	rs7928017	A	0.41	-0.033	0.006	3.13E-09	-0.008	0.008	4.00E-01
CigPDay	rs806798	C	0.53	-0.031	0.006	2.45E-08	-0.010	0.008	1.80E-01
Drinking	rs10753661	A	0.68	-0.009	0.002	3.70E-08	-0.001	0.008	7.30E-01
Drinking	rs12088813	C	0.27	-0.009	0.002	1.54E-08	-0.006	0.009	7.00E-01
Drinking	rs28680958	A	0.22	-0.011	0.002	5.20E-10	-0.001	0.009	8.40E-01
Drinking	rs5024204	T	0.28	0.010	0.002	2.55E-09	-0.012	0.009	1.20E-01
Drinking	rs58107686	A	0.33	-0.010	0.002	7.84E-10	0.001	0.008	9.40E-01
Drinking	rs705687	G	0.79	-0.011	0.002	8.25E-10	-0.001	0.009	8.80E-01
Drinking	rs823114	A	0.55	0.009	0.001	2.30E-09	0.000	0.008	9.50E-01
Drinking	rs11692435	A	0.09	0.017	0.003	2.54E-11	0.015	0.015	3.60E-01
Drinking	rs1260326	C	0.60	0.021	0.001	9.43E-45	-0.039	0.008	4.10E-07
Drinking	rs13024996	A	0.36	-0.011	0.002	5.84E-13	-0.010	0.008	3.60E-01
Drinking	rs13032049	G	0.28	0.010	0.002	2.96E-10	0.009	0.009	2.40E-01
Drinking	rs13383034	T	0.33	0.015	0.002	6.40E-22	0.015	0.008	3.40E-02
Drinking	rs56337305	C	0.38	-0.010	0.001	1.60E-10	0.001	0.008	9.80E-01
Drinking	rs72859280	T	0.04	0.023	0.004	4.49E-09	0.019	0.021	3.70E-01
Drinking	rs77165542	T	0.03	-0.026	0.004	5.76E-11	-0.049	0.021	2.20E-02
Drinking	rs828867	A	0.55	0.009	0.001	2.19E-09	0.001	0.008	9.80E-01
Drinking	rs13066454	T	0.40	-0.009	0.001	4.05E-09	-0.018	0.008	1.80E-02
Drinking	rs13094887	T	0.30	-0.010	0.002	8.67E-11	-0.023	0.008	7.60E-03
Drinking	rs2011092	C	0.34	-0.009	0.002	7.50E-09	0.006	0.008	5.60E-01
Drinking	rs62250685	G	0.61	-0.014	0.002	1.06E-21	-0.001	0.008	9.90E-01
Drinking	rs6787172	G	0.55	-0.008	0.001	4.32E-08	-0.006	0.008	4.60E-01
Drinking	rs9838144	C	0.21	-0.010	0.002	2.72E-08	0.011	0.010	2.40E-01
Drinking	rs11940694	G	0.60	0.026	0.001	2.68E-68	0.011	0.008	2.70E-01
Drinking	rs1229984	C	0.96	0.151	0.004	0.00E+00	0.158	0.025	7.00E-10
Drinking	rs12651313	G	0.44	-0.009	0.001	3.85E-09	-0.007	0.008	3.70E-01
Drinking	rs13107325	T	0.07	-0.028	0.003	1.56E-22	0.011	0.015	4.00E-01
Drinking	rs2165670	A	0.11	0.023	0.002	1.64E-22	0.042	0.013	1.80E-03
Drinking	rs36052336	G	0.06	-0.018	0.003	1.24E-09	0.002	0.016	1.00E+00
Drinking	rs3748034	T	0.14	-0.012	0.002	1.71E-08	-0.011	0.011	4.20E-01
Drinking	rs4501255	G	0.24	0.011	0.002	4.94E-10	-0.012	0.009	1.90E-01
Drinking	rs4690727	G	0.72	0.011	0.002	2.42E-11	-0.014	0.009	1.00E-01
Drinking	rs4699791	A	0.10	0.019	0.002	6.55E-14	0.016	0.013	2.50E-01

Drinking	rs79139602	T	0.02	0.060	0.005	1.61E-32	0.050	0.028	1.20E-01
Drinking	rs12655091	A	0.53	-0.008	0.001	1.25E-08	0.000	0.008	8.40E-01
Drinking	rs4916723	C	0.42	-0.010	0.001	1.69E-11	0.001	0.008	8.80E-01
Drinking	rs55872084	T	0.24	0.010	0.002	6.43E-09	0.006	0.009	5.20E-01
Drinking	rs10236149	G	0.12	-0.013	0.002	1.18E-09	-0.055	0.012	3.90E-06
Drinking	rs35034355	A	0.52	-0.008	0.001	2.86E-08	0.007	0.008	4.10E-01
Drinking	rs6460047	C	0.21	0.012	0.002	9.61E-11	0.018	0.010	5.10E-02
Drinking	rs6951574	C	0.46	0.013	0.001	1.58E-19	0.003	0.008	7.20E-01
Drinking	rs1217091	C	0.81	0.012	0.002	7.07E-11	-0.017	0.010	9.90E-02
Drinking	rs13250583	T	0.21	-0.010	0.002	4.79E-08	0.007	0.009	4.30E-01
Drinking	rs28601761	G	0.42	0.009	0.001	7.07E-10	-0.047	0.008	2.00E-09
Drinking	rs10978550	C	0.21	-0.012	0.002	7.07E-11	0.006	0.010	4.10E-01
Drinking	rs55932213	G	0.74	0.009	0.002	9.70E-09	0.001	0.009	9.70E-01
Drinking	rs17665139	T	0.15	-0.012	0.002	1.62E-08	-0.003	0.011	7.50E-01
Drinking	rs7074871	A	0.26	-0.009	0.002	1.89E-08	0.012	0.009	1.90E-01
Drinking	rs10750025	T	0.69	0.010	0.002	4.94E-11	0.011	0.008	3.10E-01
Drinking	rs11030084	T	0.18	-0.011	0.002	1.71E-08	-0.035	0.010	1.20E-03
Drinking	rs12795042	C	0.62	-0.008	0.002	3.17E-08	-0.024	0.008	2.80E-03
Drinking	rs1713676	G	0.52	-0.008	0.001	4.32E-08	0.013	0.008	1.00E-01
Drinking	rs4938230	A	0.84	0.013	0.002	1.45E-10	0.026	0.011	1.40E-02
Drinking	rs56030824	A	0.32	-0.012	0.002	1.15E-13	-0.001	0.008	9.50E-01
Drinking	rs682011	C	0.56	0.008	0.001	2.21E-08	-0.007	0.008	3.80E-01
Drinking	rs7950166	T	0.64	-0.010	0.002	1.01E-10	0.024	0.008	1.90E-03
Drinking	rs10506274	T	0.48	-0.009	0.001	5.76E-10	0.000	0.008	9.80E-01
Drinking	rs10876188	T	0.46	-0.008	0.001	4.79E-08	-0.008	0.008	3.50E-01
Drinking	rs3809162	G	0.40	0.009	0.001	1.18E-09	0.004	0.008	5.30E-01
Drinking	rs4842786	A	0.58	-0.009	0.001	2.68E-09	-0.001	0.008	9.50E-01
Drinking	rs500321	T	0.74	-0.010	0.002	4.97E-09	0.005	0.009	4.80E-01
Drinking	rs1123285	G	0.34	-0.009	0.002	8.32E-09	-0.014	0.008	7.60E-02
Drinking	rs11625650	A	0.23	-0.010	0.002	2.86E-08	0.000	0.009	8.20E-01
Drinking	rs2180870	C	0.14	-0.012	0.002	1.13E-08	0.015	0.011	9.50E-02
Drinking	rs28929474	T	0.02	-0.037	0.005	1.31E-11	-0.026	0.028	2.80E-01
Drinking	rs12907323	G	0.41	0.008	0.001	9.70E-09	0.003	0.008	7.80E-01
Drinking	rs2472297	T	0.25	0.011	0.002	3.12E-10	0.006	0.009	5.40E-01
Drinking	rs1104608	C	0.43	-0.011	0.001	1.03E-13	-0.004	0.008	6.80E-01
Drinking	rs113443718	A	0.31	-0.010	0.002	1.18E-10	0.017	0.008	2.80E-02
Drinking	rs17177078	T	0.06	-0.022	0.003	1.27E-13	-0.016	0.017	3.10E-01
Drinking	rs2764771	A	0.31	0.010	0.002	4.03E-10	0.002	0.008	8.50E-01
Drinking	rs378421	A	0.40	-0.011	0.001	4.82E-14	0.013	0.008	7.40E-02
Drinking	rs62044525	G	0.18	-0.012	0.002	1.01E-10	-0.017	0.010	6.00E-02
Drinking	rs7185555	C	0.15	-0.011	0.002	4.32E-08	-0.002	0.011	9.80E-01
Drinking	rs79616692	C	0.11	0.016	0.002	4.05E-12	-0.003	0.013	8.90E-01
Drinking	rs10438820	T	0.70	0.009	0.002	1.80E-08	0.007	0.008	4.60E-01
Drinking	rs2854334	G	0.62	0.009	0.001	7.45E-10	0.002	0.008	6.70E-01
Drinking	rs3803800	G	0.79	0.011	0.002	1.52E-10	-0.022	0.010	2.10E-02
Drinking	rs4548913	A	0.63	-0.008	0.002	3.17E-08	0.001	0.008	9.90E-01
Drinking	rs4092465	G	0.64	-0.008	0.002	4.32E-08	0.007	0.008	3.10E-01
Drinking	rs9950000	T	0.40	-0.009	0.001	9.62E-10	0.004	0.008	4.90E-01
Drinking	rs281379	A	0.51	0.014	0.001	4.83E-21	0.009	0.008	2.20E-01
Drinking	rs4815364	A	0.62	0.009	0.001	1.02E-08	0.005	0.008	4.50E-01
Drinking	rs9607814	A	0.20	-0.010	0.002	4.32E-08	-0.004	0.010	6.00E-01
FG	rs2075423	T	0.38	-0.016	0.002	2.78E-21	-0.010	0.008	1.80E-01
FG	rs348330	A	0.63	-0.012	0.002	1.06E-09	-0.012	0.008	9.80E-02
FG	rs6662924	A	0.20	0.014	0.002	5.05E-10	-0.016	0.010	1.10E-01
FG	rs78132593	A	0.20	-0.015	0.002	2.36E-11	0.016	0.009	9.90E-02
FG	rs1057394	A	0.63	-0.012	0.002	5.62E-12	0.026	0.008	1.30E-03

FG	rs1260326	T	0.41	-0.028	0.002	8.48E-62	0.039	0.008	4.10E-07
FG	rs13389076	A	0.03	0.061	0.005	1.83E-35	0.045	0.033	2.30E-01
FG	rs13431652	T	0.68	0.069	0.002	0.00E+00	-0.002	0.008	6.60E-01
FG	rs140809953	A	0.02	0.037	0.007	4.31E-07	-0.040	0.032	2.20E-01
FG	rs180935712	A	0.01	0.057	0.008	3.62E-12	-0.030	0.029	2.70E-01
FG	rs189548	A	0.72	-0.012	0.002	7.75E-10	0.002	0.009	8.10E-01
FG	rs7584277	A	0.08	0.027	0.004	1.48E-13	0.012	0.015	4.20E-01
FG	rs77981966	T	0.06	-0.025	0.004	2.09E-12	0.003	0.015	8.60E-01
FG	rs11708067	A	0.82	0.028	0.002	7.70E-45	-0.023	0.009	1.10E-02
FG	rs1604038	T	0.29	-0.020	0.002	3.82E-28	0.015	0.009	4.40E-02
FG	rs16851397	A	0.96	0.033	0.004	6.93E-15	0.007	0.018	7.80E-01
FG	rs17437560	T	0.11	-0.018	0.003	4.53E-08	-0.017	0.013	2.50E-01
FG	rs6808574	T	0.39	-0.013	0.002	7.98E-14	-0.001	0.008	8.60E-01
FG	rs4862423	T	0.40	0.012	0.002	9.56E-11	0.009	0.008	2.70E-01
FG	rs157512	T	0.73	0.013	0.002	1.76E-10	0.011	0.009	2.80E-01
FG	rs1820176	T	0.70	0.025	0.002	4.87E-35	-0.005	0.009	4.70E-01
FG	rs7708285	A	0.69	-0.013	0.002	2.56E-12	-0.009	0.008	2.70E-01
FG	rs10305457	T	0.06	0.024	0.003	2.08E-13	-0.009	0.013	5.40E-01
FG	rs12055786	T	0.38	0.012	0.002	1.68E-12	0.009	0.008	4.40E-01
FG	rs3778321	A	0.18	-0.019	0.002	8.21E-19	-0.013	0.010	2.40E-01
FG	rs9348441	A	0.27	0.018	0.002	1.40E-22	0.001	0.009	8.30E-01
FG	rs10487796	A	0.48	-0.026	0.002	8.04E-60	-0.001	0.008	7.40E-01
FG	rs17168486	T	0.18	0.028	0.002	1.48E-40	0.005	0.010	7.10E-01
FG	rs194518	A	0.52	0.010	0.002	1.46E-08	-0.004	0.008	5.00E-01
FG	rs2595701	A	0.32	0.019	0.002	2.26E-19	-0.023	0.008	4.70E-03
FG	rs58925536	T	0.03	0.031	0.005	7.76E-09	-0.015	0.021	4.90E-01
FG	rs878521	A	0.25	0.055	0.002	6.95E-166	-0.003	0.009	8.60E-01
FG	rs12541643	T	0.48	0.012	0.002	5.28E-10	0.008	0.008	2.70E-01
FG	rs7012637	A	0.47	-0.018	0.002	3.38E-26	0.002	0.008	8.40E-01
FG	rs896854	T	0.46	0.010	0.002	6.11E-10	0.007	0.008	3.40E-01
FG	rs9650069	T	0.28	-0.029	0.002	7.57E-57	0.006	0.008	5.80E-01
FG	rs10811660	A	0.17	-0.022	0.002	3.81E-24	0.003	0.010	7.10E-01
FG	rs10974438	A	0.62	-0.020	0.002	2.38E-31	0.008	0.008	3.30E-01
FG	rs16913693	T	0.97	0.039	0.005	8.92E-16	0.012	0.025	6.80E-01
FG	rs3829109	A	0.28	-0.016	0.002	3.64E-16	-0.018	0.009	4.30E-02
FG	rs507666	A	0.19	0.016	0.002	5.74E-15	0.023	0.010	1.90E-02
FG	rs12784552	A	0.92	0.033	0.003	5.53E-28	-0.042	0.013	1.70E-03
FG	rs2839671	A	0.16	-0.016	0.002	3.52E-13	-0.008	0.010	3.10E-01
FG	rs7095788	T	0.36	-0.011	0.002	3.89E-09	-0.017	0.008	4.00E-02
FG	rs7903146	T	0.31	0.026	0.002	2.60E-42	0.010	0.009	2.10E-01
FG	rs10838524	A	0.48	0.024	0.002	4.79E-50	0.004	0.008	6.00E-01
FG	rs10838693	C	0.31	0.018	0.002	8.09E-23	-0.008	0.008	3.30E-01
FG	rs11020124	T	0.72	-0.060	0.002	3.79E-218	0.000	0.009	8.50E-01
FG	rs11603349	T	0.83	0.024	0.002	7.58E-27	0.004	0.011	6.70E-01
FG	rs174583	T	0.38	-0.017	0.002	4.97E-23	-0.001	0.008	9.90E-01
FG	rs192701415	A	0.90	0.020	0.003	3.38E-09	0.014	0.014	3.10E-01
FG	rs3842753	T	0.28	0.013	0.002	1.12E-09	-0.002	0.009	7.60E-01
FG	rs11610045	A	0.45	0.014	0.002	3.48E-14	0.006	0.008	5.20E-01
FG	rs2657879	A	0.80	-0.012	0.002	6.33E-08	-0.015	0.010	1.90E-01
FG	rs4760278	A	0.18	-0.011	0.002	3.80E-08	-0.006	0.009	5.40E-01
FG	rs6489811	A	0.49	-0.011	0.002	9.89E-10	-0.002	0.008	8.90E-01
FG	rs6538804	C	0.62	0.014	0.002	7.80E-14	-0.001	0.008	9.00E-01
FG	rs11619319	A	0.77	-0.017	0.002	5.15E-18	-0.002	0.009	6.70E-01
FG	rs12888855	A	0.19	-0.014	0.002	1.48E-11	-0.003	0.009	8.00E-01
FG	rs35889227	T	0.62	-0.013	0.002	7.80E-12	-0.004	0.008	6.90E-01
FG	rs12898997	T	0.60	-0.010	0.002	8.18E-09	0.005	0.008	5.60E-01

FG	rs17270243	A	0.76	-0.010	0.002	7.33E-07	0.003	0.009	7.20E-01
FG	rs6598541	A	0.35	0.011	0.002	2.00E-11	-0.006	0.008	4.50E-01
FG	rs7163757	T	0.43	-0.022	0.002	6.68E-42	0.008	0.008	2.70E-01
FG	rs7178572	A	0.32	-0.012	0.002	1.79E-11	-0.020	0.009	1.80E-02
FG	rs2238435	C	0.38	0.011	0.002	3.75E-09	-0.009	0.008	2.70E-01
FG	rs17265513	T	0.80	-0.016	0.002	5.32E-14	-0.008	0.010	2.80E-01
FG	rs6113722	A	0.07	-0.042	0.004	5.61E-22	-0.006	0.021	8.30E-01
FG	rs39713	T	0.06	-0.017	0.003	4.99E-08	-0.009	0.013	5.00E-01
FI	rs6674544	A	0.57	0.018	0.002	8.75E-19	0.016	0.008	6.30E-02
FI	rs1260326	T	0.41	-0.023	0.002	5.21E-34	0.039	0.008	4.10E-07
FI	rs13389219	T	0.41	-0.020	0.002	1.14E-25	-0.036	0.008	1.00E-05
FI	rs2943646	A	0.38	-0.025	0.002	1.53E-39	-0.018	0.008	1.90E-02
FI	rs5017305	A	0.24	0.014	0.003	1.37E-07	0.004	0.010	7.30E-01
FI	rs10865959	C	0.30	0.014	0.002	3.55E-10	0.030	0.008	1.80E-04
FI	rs11708067	A	0.82	-0.014	0.002	4.37E-09	-0.023	0.009	1.10E-02
FI	rs17036126	T	0.13	0.021	0.003	3.25E-12	0.005	0.011	5.90E-01
FI	rs17331151	T	0.11	-0.016	0.003	1.73E-07	-0.018	0.012	1.70E-01
FI	rs35000407	T	0.88	0.026	0.003	3.13E-20	0.048	0.012	8.90E-05
FI	rs62271373	A	0.06	0.026	0.005	9.64E-08	0.046	0.017	6.40E-03
FI	rs11727676	T	0.92	-0.020	0.004	1.94E-07	-0.008	0.013	5.00E-01
FI	rs3775380	A	0.50	-0.012	0.002	3.81E-11	-0.013	0.008	6.60E-02
FI	rs6855363	T	0.65	0.013	0.002	4.10E-10	0.018	0.008	2.80E-02
FI	rs9884482	T	0.61	-0.013	0.002	4.74E-11	0.013	0.008	1.30E-01
FI	rs10050393	T	0.54	0.009	0.002	2.17E-06	0.015	0.008	6.40E-02
FI	rs459193	A	0.29	-0.018	0.002	6.75E-18	-0.014	0.009	1.60E-01
FI	rs4865796	A	0.71	0.017	0.002	1.58E-16	0.025	0.008	2.10E-03
FI	rs116141873	T	0.03	0.043	0.006	4.04E-13	0.041	0.018	2.50E-02
FI	rs1474696	A	0.52	-0.015	0.002	3.17E-16	-0.024	0.008	2.80E-03
FI	rs2780215	A	0.96	0.039	0.006	4.90E-10	0.013	0.017	4.60E-01
FI	rs6905288	A	0.60	0.011	0.002	3.75E-09	0.018	0.008	3.30E-02
FI	rs73013411	A	0.12	-0.018	0.003	1.86E-08	-0.028	0.011	2.20E-02
FI	rs2108349	A	0.69	-0.012	0.002	8.92E-09	-0.011	0.008	1.40E-01
FI	rs972283	A	0.46	-0.011	0.002	3.27E-08	-0.012	0.008	8.10E-02
FI	rs13258890	T	0.75	0.013	0.003	3.06E-07	0.016	0.009	9.10E-02
FI	rs7012814	A	0.47	-0.022	0.002	9.72E-31	0.002	0.008	8.40E-01
FI	rs75179845	T	0.92	-0.022	0.004	6.77E-10	-0.030	0.017	6.90E-02
FI	rs118164457	T	0.96	-0.035	0.006	1.42E-09	-0.017	0.019	4.50E-01
FI	rs7903146	T	0.31	-0.012	0.002	3.32E-08	0.010	0.009	2.10E-01
FI	rs2845885	T	0.93	-0.020	0.004	1.69E-07	-0.012	0.017	5.10E-01
FI	rs1351394	T	0.47	-0.011	0.002	6.97E-10	-0.022	0.008	6.30E-03
FI	rs6487237	A	0.79	0.015	0.003	3.16E-09	-0.009	0.010	3.70E-01
FI	rs7133378	A	0.34	-0.013	0.002	2.15E-10	-0.028	0.008	1.30E-03
FI	rs860598	A	0.82	0.018	0.003	1.44E-12	0.001	0.011	8.10E-01
FI	rs12454712	T	0.60	0.014	0.003	1.35E-08	0.016	0.008	5.70E-02
FI	rs731839	A	0.66	-0.012	0.002	1.91E-10	-0.009	0.008	2.60E-01
FI	rs1206760	A	0.52	-0.011	0.002	3.75E-09	-0.008	0.008	3.50E-01
HDLC	rs1043897	G	0.58	-0.020	0.002	8.45E-25	0.007	0.008	3.20E-01
HDLC	rs10916239	T	0.62	-0.012	0.002	1.47E-09	0.016	0.008	5.10E-02
HDLC	rs11118320	C	0.44	0.015	0.002	1.21E-14	-0.013	0.008	1.20E-01
HDLC	rs113261881	G	0.94	0.024	0.004	1.31E-09	0.005	0.016	6.00E-01
HDLC	rs1168114	A	0.35	-0.016	0.002	6.63E-15	0.006	0.008	6.60E-01
HDLC	rs12045101	C	0.76	0.015	0.002	6.66E-11	0.004	0.009	6.10E-01
HDLC	rs12119128	G	0.70	0.012	0.002	3.35E-08	-0.006	0.009	4.40E-01
HDLC	rs12740374	G	0.78	-0.029	0.002	2.20E-36	-0.010	0.009	2.00E-01
HDLC	rs140584594	A	0.27	-0.031	0.002	5.51E-48	-0.039	0.009	1.20E-05
HDLC	rs141440048	C	0.98	-0.044	0.008	6.71E-09	-0.008	0.031	9.50E-01

HDLC	rs1663626	C	0.60	-0.013	0.002	2.92E-11	-0.003	0.008	7.20E-01
HDLC	rs17185038	C	0.93	-0.021	0.004	2.69E-08	0.024	0.016	1.30E-01
HDLC	rs1771582	T	0.44	-0.013	0.002	7.08E-11	-0.001	0.008	9.20E-01
HDLC	rs185073199	T	0.99	-0.064	0.010	2.50E-10	0.005	0.041	8.30E-01
HDLC	rs193084249	A	0.98	0.083	0.006	3.84E-38	-0.029	0.026	2.40E-01
HDLC	rs2281718	A	0.39	-0.060	0.002	8.57E-205	0.014	0.008	9.00E-02
HDLC	rs2298214	C	0.42	0.012	0.002	1.40E-10	-0.002	0.008	8.50E-01
HDLC	rs2298632	C	0.50	-0.014	0.002	1.59E-13	0.014	0.008	2.70E-02
HDLC	rs2642438	A	0.30	-0.028	0.002	1.92E-40	-0.052	0.008	1.70E-09
HDLC	rs267738	T	0.78	-0.021	0.002	1.16E-20	-0.018	0.009	6.50E-02
HDLC	rs3747973	A	0.41	-0.014	0.002	2.94E-13	0.000	0.008	9.10E-01
HDLC	rs3768321	G	0.80	0.045	0.002	1.28E-79	-0.005	0.010	6.10E-01
HDLC	rs3903399	T	0.79	0.013	0.002	2.89E-08	0.002	0.009	7.60E-01
HDLC	rs4233367	T	0.39	-0.011	0.002	4.69E-08	0.002	0.008	8.50E-01
HDLC	rs4650994	G	0.47	0.018	0.002	2.00E-21	0.011	0.008	2.30E-01
HDLC	rs4654395	C	0.48	0.012	0.002	2.16E-10	-0.011	0.008	2.00E-01
HDLC	rs4997370	G	0.41	-0.011	0.002	2.22E-08	0.012	0.008	1.60E-01
HDLC	rs557933	A	0.48	-0.015	0.002	1.04E-15	-0.016	0.008	5.30E-02
HDLC	rs56105022	G	0.96	0.034	0.005	3.49E-10	0.005	0.022	7.60E-01
HDLC	rs61805075	G	0.67	0.026	0.002	7.22E-37	-0.001	0.008	9.00E-01
HDLC	rs6664374	C	0.65	-0.014	0.002	2.43E-12	-0.004	0.008	4.90E-01
HDLC	rs72654647	G	0.75	0.014	0.002	3.11E-10	0.001	0.009	9.40E-01
HDLC	rs74328314	A	0.93	-0.023	0.004	1.28E-09	-0.029	0.015	6.10E-02
HDLC	rs75246752	G	0.99	-0.049	0.008	5.53E-09	0.006	0.034	9.70E-01
HDLC	rs771481	T	0.82	-0.029	0.002	8.55E-32	-0.003	0.010	7.80E-01
HDLC	rs1047891	C	0.68	0.019	0.002	2.69E-20	0.004	0.008	7.40E-01
HDLC	rs1083470	G	0.38	-0.012	0.002	3.80E-09	0.005	0.008	7.40E-01
HDLC	rs11687520	T	0.83	-0.017	0.003	2.88E-11	-0.006	0.010	4.00E-01
HDLC	rs11688682	G	0.73	-0.015	0.002	2.35E-11	0.017	0.009	3.80E-02
HDLC	rs11883967	A	0.34	-0.013	0.002	4.44E-10	0.004	0.008	4.50E-01
HDLC	rs12692596	C	0.63	0.011	0.002	1.34E-08	-0.004	0.008	5.40E-01
HDLC	rs12713007	C	0.50	0.011	0.002	2.07E-08	-0.014	0.008	9.40E-02
HDLC	rs12986742	T	0.52	0.011	0.002	3.85E-08	-0.011	0.008	2.30E-01
HDLC	rs12998038	C	0.74	-0.013	0.002	1.44E-09	0.001	0.009	7.90E-01
HDLC	rs13389219	C	0.61	-0.028	0.002	8.16E-46	0.036	0.008	1.00E-05
HDLC	rs1446585	A	0.76	-0.017	0.002	1.14E-14	0.003	0.009	8.00E-01
HDLC	rs17041868	T	0.94	0.025	0.004	2.38E-10	-0.032	0.016	4.00E-02
HDLC	rs17326656	G	0.76	0.023	0.002	8.22E-24	-0.022	0.009	1.40E-02
HDLC	rs2362541	T	0.49	0.011	0.002	1.19E-08	-0.010	0.008	1.50E-01
HDLC	rs2364723	G	0.68	-0.012	0.002	2.71E-09	0.004	0.008	6.00E-01
HDLC	rs2723065	A	0.62	-0.015	0.002	2.53E-14	0.001	0.008	9.30E-01
HDLC	rs2943645	C	0.35	0.043	0.002	8.60E-106	-0.018	0.008	2.00E-02
HDLC	rs35706812	A	0.57	0.013	0.002	8.20E-12	-0.004	0.008	5.50E-01
HDLC	rs4550673	A	0.92	0.019	0.003	2.05E-08	-0.036	0.014	3.00E-02
HDLC	rs4599108	C	0.51	-0.014	0.002	1.23E-12	-0.005	0.008	5.80E-01
HDLC	rs56131490	G	0.85	-0.015	0.003	1.95E-08	0.016	0.011	1.70E-01
HDLC	rs57074291	C	0.74	-0.013	0.002	1.44E-09	0.003	0.009	7.40E-01
HDLC	rs59104589	C	0.64	-0.015	0.002	3.59E-14	0.021	0.008	9.30E-03
HDLC	rs6542680	C	0.18	0.025	0.002	1.78E-23	-0.016	0.010	1.40E-01
HDLC	rs6738438	C	0.64	0.011	0.002	1.12E-08	0.002	0.008	9.20E-01
HDLC	rs676210	G	0.79	-0.059	0.002	3.69E-140	0.004	0.010	6.10E-01
HDLC	rs72926946	C	0.70	0.021	0.002	2.35E-23	-0.003	0.008	9.10E-01
HDLC	rs7583067	C	0.76	-0.015	0.002	7.36E-11	0.018	0.009	5.40E-02
HDLC	rs7595075	C	0.65	-0.014	0.002	5.45E-13	0.012	0.008	2.00E-01
HDLC	rs78058190	G	0.95	0.078	0.005	1.58E-57	-0.024	0.020	2.70E-01
HDLC	rs78456138	C	0.98	-0.035	0.006	3.49E-08	0.020	0.026	4.00E-01

HDLC	rs907866	G	0.55	0.018	0.002	1.56E-21	0.011	0.008	2.40E-01
HDLC	rs9646934	C	0.32	-0.012	0.002	3.27E-09	0.008	0.008	1.80E-01
HDLC	rs10513801	T	0.86	0.030	0.003	8.29E-28	-0.018	0.011	6.20E-02
HDLC	rs1086056	T	0.16	0.017	0.003	3.61E-10	-0.017	0.011	8.20E-02
HDLC	rs11546878	C	0.83	-0.014	0.003	2.84E-08	0.004	0.010	5.50E-01
HDLC	rs1225053	T	0.74	0.015	0.002	4.68E-12	-0.011	0.009	2.40E-01
HDLC	rs12485478	A	0.97	0.052	0.006	1.10E-18	-0.041	0.024	1.20E-01
HDLC	rs13066793	A	0.91	-0.022	0.003	3.29E-11	0.031	0.013	1.90E-02
HDLC	rs13076933	T	0.74	0.017	0.002	2.25E-14	0.007	0.009	4.70E-01
HDLC	rs13097947	T	0.35	-0.016	0.002	3.67E-15	0.018	0.008	2.90E-02
HDLC	rs13323506	A	0.31	-0.013	0.002	1.87E-10	-0.010	0.008	2.70E-01
HDLC	rs139828053	T	0.97	0.032	0.006	1.47E-08	-0.005	0.022	9.30E-01
HDLC	rs2159607	G	0.19	0.024	0.002	9.11E-23	0.002	0.010	8.20E-01
HDLC	rs2268840	T	0.77	-0.017	0.002	3.62E-14	-0.005	0.009	5.40E-01
HDLC	rs34717889	T	0.80	-0.014	0.002	3.52E-09	0.020	0.010	3.20E-02
HDLC	rs3732356	G	0.07	0.030	0.004	8.70E-15	0.029	0.016	6.40E-02
HDLC	rs4686739	A	0.37	-0.017	0.002	2.84E-17	-0.003	0.008	6.70E-01
HDLC	rs4855582	C	0.57	-0.011	0.002	7.97E-09	-0.005	0.008	4.60E-01
HDLC	rs62271373	T	0.94	0.041	0.004	2.22E-23	-0.046	0.017	6.40E-03
HDLC	rs6762415	T	0.46	0.011	0.002	1.20E-08	-0.006	0.008	2.90E-01
HDLC	rs6765484	C	0.53	-0.022	0.002	6.51E-32	0.020	0.008	7.50E-03
HDLC	rs6790951	C	0.52	0.012	0.002	1.64E-09	0.009	0.008	2.70E-01
HDLC	rs6806529	A	0.44	-0.011	0.002	6.93E-09	0.023	0.008	4.50E-03
HDLC	rs7622114	C	0.42	-0.012	0.002	2.71E-09	0.007	0.008	4.70E-01
HDLC	rs7650845	C	0.19	0.014	0.002	9.75E-09	-0.005	0.010	7.40E-01
HDLC	rs78595810	G	0.99	0.053	0.008	2.27E-10	-0.086	0.033	1.70E-02
HDLC	rs830620	C	0.58	-0.015	0.002	8.24E-15	0.006	0.008	4.80E-01
HDLC	rs9647335	A	0.81	-0.028	0.002	2.41E-30	0.009	0.010	3.80E-01
HDLC	rs9877304	G	0.74	0.014	0.002	4.03E-10	0.001	0.009	9.50E-01
HDLC	rs1055582	C	0.49	-0.014	0.002	2.82E-13	0.007	0.008	4.10E-01
HDLC	rs11938781	T	0.83	0.015	0.003	6.40E-09	-0.014	0.010	1.40E-01
HDLC	rs12510382	A	0.54	-0.017	0.002	7.06E-18	0.009	0.008	1.90E-01
HDLC	rs12650112	C	0.65	-0.016	0.002	1.03E-14	-0.006	0.008	4.80E-01
HDLC	rs13107325	C	0.93	0.080	0.004	2.23E-108	-0.011	0.015	4.00E-01
HDLC	rs13111599	A	0.26	-0.013	0.002	2.63E-09	0.005	0.009	5.40E-01
HDLC	rs13144151	A	0.15	-0.018	0.003	3.90E-11	-0.010	0.011	3.60E-01
HDLC	rs13147189	T	0.68	0.014	0.002	2.53E-12	-0.009	0.008	3.00E-01
HDLC	rs1349852	A	0.52	-0.011	0.002	5.75E-09	-0.005	0.008	3.70E-01
HDLC	rs1395221	G	0.60	0.011	0.002	1.12E-08	0.005	0.008	5.50E-01
HDLC	rs2098918	C	0.54	-0.012	0.002	6.43E-10	-0.007	0.008	3.80E-01
HDLC	rs2175766	A	0.46	-0.011	0.002	1.80E-08	0.008	0.008	3.30E-01
HDLC	rs2237035	G	0.61	-0.014	0.002	1.28E-12	0.004	0.008	5.50E-01
HDLC	rs28455602	A	0.81	-0.014	0.002	1.99E-08	0.006	0.010	5.50E-01
HDLC	rs35777071	T	0.35	-0.016	0.002	3.48E-15	-0.005	0.008	4.50E-01
HDLC	rs3775228	C	0.60	0.020	0.002	1.92E-24	-0.005	0.008	5.60E-01
HDLC	rs4691379	C	0.68	-0.012	0.002	6.09E-09	0.019	0.008	1.90E-02
HDLC	rs546436456	T	0.97	0.032	0.006	3.65E-08	-0.036	0.023	7.40E-02
HDLC	rs62338910	A	0.76	-0.016	0.002	3.99E-12	0.001	0.009	9.00E-01
HDLC	rs6824451	G	0.54	0.020	0.002	9.68E-26	-0.015	0.008	4.60E-02
HDLC	rs73243877	A	0.83	0.025	0.003	2.21E-23	-0.017	0.010	7.60E-02
HDLC	rs7658082	T	0.45	0.011	0.002	3.16E-09	-0.004	0.008	6.90E-01
HDLC	rs78025076	C	0.98	0.049	0.007	3.00E-13	0.009	0.028	5.00E-01
HDLC	rs880674	T	0.86	-0.015	0.003	4.24E-08	0.006	0.011	5.90E-01
HDLC	rs9884482	T	0.63	0.013	0.002	1.74E-10	0.013	0.008	1.30E-01
HDLC	rs1045241	C	0.73	-0.016	0.002	2.13E-14	0.005	0.009	5.40E-01
HDLC	rs115912456	A	0.96	-0.028	0.005	4.16E-09	0.012	0.019	6.90E-01

HDLC	rs116006942	G	0.94	0.030	0.004	1.02E-13	-0.003	0.016	8.50E-01
HDLC	rs11948445	A	0.59	0.011	0.002	1.49E-08	-0.010	0.008	1.80E-01
HDLC	rs12516070	T	0.48	-0.012	0.002	1.01E-10	0.004	0.008	5.80E-01
HDLC	rs138354839	C	0.98	0.060	0.008	9.71E-13	0.006	0.034	7.70E-01
HDLC	rs16885512	C	0.92	0.020	0.004	1.79E-08	-0.011	0.015	5.70E-01
HDLC	rs1862205	G	0.60	-0.011	0.002	6.52E-09	-0.004	0.008	4.30E-01
HDLC	rs2307111	T	0.61	-0.019	0.002	1.99E-22	0.015	0.008	3.70E-02
HDLC	rs2339234	G	0.32	0.012	0.002	6.69E-09	-0.013	0.008	1.30E-01
HDLC	rs248653	T	0.96	0.032	0.005	3.43E-10	0.020	0.020	2.90E-01
HDLC	rs254024	G	0.56	0.010	0.002	4.94E-08	-0.014	0.008	4.70E-02
HDLC	rs254559	C	0.60	0.011	0.002	3.38E-09	0.004	0.008	6.00E-01
HDLC	rs2910949	T	0.65	-0.013	0.002	1.98E-11	0.001	0.008	9.90E-01
HDLC	rs2963468	A	0.76	0.020	0.002	3.47E-18	-0.014	0.009	1.20E-01
HDLC	rs2964007	A	0.34	-0.014	0.002	7.04E-12	0.011	0.008	1.40E-01
HDLC	rs32578	G	0.69	-0.013	0.002	1.11E-10	-0.009	0.008	3.60E-01
HDLC	rs3733890	G	0.71	0.014	0.002	4.10E-11	0.002	0.009	8.80E-01
HDLC	rs3749748	C	0.75	-0.017	0.002	7.85E-14	-0.009	0.009	4.10E-01
HDLC	rs3936511	A	0.81	0.030	0.002	3.61E-36	-0.041	0.010	3.90E-05
HDLC	rs445841	G	0.63	0.011	0.002	4.47E-08	-0.009	0.008	2.30E-01
HDLC	rs454968	T	0.36	-0.011	0.002	4.03E-08	0.009	0.008	3.30E-01
HDLC	rs4976033	A	0.60	0.012	0.002	1.02E-09	-0.012	0.008	1.60E-01
HDLC	rs6874833	C	0.37	-0.011	0.002	8.97E-09	0.007	0.008	3.90E-01
HDLC	rs6893139	G	0.60	0.011	0.002	2.36E-08	-0.004	0.008	7.20E-01
HDLC	rs72801474	G	0.91	-0.021	0.003	1.13E-10	0.040	0.013	2.70E-03
HDLC	rs7725218	G	0.66	0.012	0.002	1.48E-09	0.008	0.008	3.80E-01
HDLC	rs1155347	T	0.78	-0.014	0.002	5.87E-09	0.003	0.010	6.20E-01
HDLC	rs1240820	G	0.71	-0.013	0.002	3.50E-10	0.012	0.009	2.50E-01
HDLC	rs13195251	T	0.29	0.012	0.002	2.03E-08	-0.005	0.009	5.90E-01
HDLC	rs2263329	T	0.64	0.027	0.002	7.47E-43	-0.006	0.008	3.20E-01
HDLC	rs2750411	T	0.51	0.011	0.002	1.37E-08	-0.002	0.008	6.60E-01
HDLC	rs2781668	C	0.83	0.017	0.003	7.61E-11	-0.005	0.010	7.70E-01
HDLC	rs3763236	T	0.49	-0.015	0.002	8.16E-15	-0.009	0.008	1.80E-01
HDLC	rs4947121	T	0.23	0.014	0.002	4.77E-10	-0.001	0.009	7.70E-01
HDLC	rs502627	A	0.90	-0.018	0.003	3.50E-08	0.012	0.013	3.20E-01
HDLC	rs571848809	G	0.89	0.057	0.003	6.73E-75	-0.014	0.013	2.70E-01
HDLC	rs62428831	T	0.86	-0.018	0.003	1.10E-10	0.023	0.011	1.20E-02
HDLC	rs632057	T	0.37	-0.020	0.002	1.64E-23	0.010	0.008	1.50E-01
HDLC	rs6457807	T	0.83	-0.019	0.003	1.33E-13	0.016	0.010	1.60E-01
HDLC	rs6902116	A	0.68	0.027	0.002	1.93E-40	0.008	0.008	2.30E-01
HDLC	rs6924387	A	0.59	0.014	0.002	2.01E-12	-0.006	0.008	4.30E-01
HDLC	rs6934962	C	0.60	-0.016	0.002	5.56E-17	0.016	0.008	6.70E-02
HDLC	rs6940493	A	0.31	-0.012	0.002	5.02E-09	0.000	0.008	7.40E-01
HDLC	rs72959041	G	0.95	0.047	0.004	2.39E-26	-0.038	0.018	2.30E-02
HDLC	rs74419430	T	0.58	-0.016	0.002	4.12E-17	0.008	0.008	5.20E-01
HDLC	rs75479205	A	0.81	-0.014	0.002	1.04E-08	0.013	0.010	2.60E-01
HDLC	rs7750688	C	0.24	-0.021	0.002	4.25E-22	-0.010	0.009	3.70E-01
HDLC	rs7757193	G	0.63	0.019	0.002	7.62E-22	0.015	0.008	6.70E-02
HDLC	rs7769059	G	0.72	0.015	0.002	1.15E-12	0.003	0.009	7.90E-01
HDLC	rs9274346	C	0.61	-0.015	0.002	2.72E-14	-0.017	0.008	3.60E-02
HDLC	rs9347737	A	0.57	0.014	0.002	3.49E-12	-0.007	0.008	5.70E-01
HDLC	rs968050	C	0.52	-0.014	0.002	1.28E-12	0.027	0.008	2.80E-04
HDLC	rs998584	C	0.52	0.034	0.002	2.92E-71	-0.018	0.008	2.20E-02
HDLC	rs10233430	T	0.57	0.020	0.002	2.49E-26	0.002	0.008	7.00E-01
HDLC	rs10252234	C	0.76	-0.016	0.002	2.39E-12	0.004	0.009	6.20E-01
HDLC	rs10950390	C	0.79	0.015	0.002	1.28E-10	-0.008	0.010	4.60E-01
HDLC	rs12705595	G	0.63	-0.011	0.002	2.39E-08	0.008	0.008	3.10E-01

HDLC	rs13235365	C	0.73	-0.026	0.002	2.92E-33	-0.004	0.009	5.00E-01
HDLC	rs144567740	T	0.81	-0.014	0.003	2.46E-08	0.014	0.010	1.70E-01
HDLC	rs150300171	A	0.95	0.029	0.005	9.21E-11	-0.034	0.019	8.70E-02
HDLC	rs1534696	C	0.46	-0.017	0.002	2.65E-18	0.004	0.008	5.60E-01
HDLC	rs17138358	G	0.60	0.027	0.002	4.22E-44	0.009	0.008	3.30E-01
HDLC	rs183906992	T	0.96	-0.029	0.005	5.43E-10	-0.036	0.019	9.50E-02
HDLC	rs201441	T	0.42	0.011	0.002	1.11E-08	0.003	0.008	7.90E-01
HDLC	rs2534596	A	0.62	-0.011	0.002	8.83E-09	0.012	0.008	1.10E-01
HDLC	rs34940374	G	0.82	0.017	0.002	5.78E-12	-0.014	0.010	1.90E-01
HDLC	rs35493868	C	0.80	-0.037	0.002	2.25E-55	-0.001	0.010	8.90E-01
HDLC	rs35580606	A	0.48	0.011	0.002	1.18E-08	-0.006	0.008	3.50E-01
HDLC	rs38166	T	0.25	-0.013	0.002	1.41E-08	0.015	0.009	5.70E-02
HDLC	rs42125	A	0.98	0.040	0.007	1.29E-08	0.010	0.029	9.20E-01
HDLC	rs4410790	T	0.37	0.011	0.002	1.96E-08	-0.005	0.008	3.50E-01
HDLC	rs4731701	C	0.51	-0.030	0.002	1.04E-54	0.012	0.008	7.50E-02
HDLC	rs55935382	C	0.67	-0.018	0.002	5.46E-18	0.003	0.008	5.90E-01
HDLC	rs62492368	G	0.69	0.017	0.002	1.51E-16	-0.007	0.009	3.80E-01
HDLC	rs66763009	T	0.56	0.016	0.002	9.69E-17	-0.002	0.008	9.50E-01
HDLC	rs73151974	C	0.86	0.016	0.003	3.81E-09	-0.011	0.011	2.70E-01
HDLC	rs77605964	G	0.77	-0.017	0.002	7.51E-14	-0.021	0.009	2.10E-02
HDLC	rs10504477	T	0.59	0.015	0.002	6.42E-15	0.000	0.008	9.10E-01
HDLC	rs10955991	T	0.32	0.020	0.002	2.83E-22	-0.001	0.008	9.30E-01
HDLC	rs13263821	G	0.81	0.017	0.002	1.21E-11	-0.023	0.010	3.60E-02
HDLC	rs13269725	A	0.92	0.026	0.004	2.12E-13	-0.025	0.014	7.50E-02
HDLC	rs140064750	T	0.98	0.044	0.006	4.10E-12	-0.002	0.026	9.10E-01
HDLC	rs142288236	C	0.99	0.078	0.008	1.95E-22	0.033	0.033	4.10E-01
HDLC	rs1431659	A	0.27	-0.013	0.002	2.52E-09	0.001	0.009	7.80E-01
HDLC	rs17740942	T	0.88	-0.020	0.003	4.74E-11	0.006	0.013	7.90E-01
HDLC	rs2247355	C	0.82	-0.021	0.002	5.82E-17	0.017	0.010	9.10E-02
HDLC	rs308	T	0.98	-0.126	0.007	1.41E-79	-0.024	0.028	5.30E-01
HDLC	rs330042	G	0.73	-0.015	0.002	1.34E-11	0.001	0.009	8.50E-01
HDLC	rs343	C	0.92	-0.134	0.003	0.00E+00	0.034	0.014	2.80E-02
HDLC	rs4871603	C	0.35	-0.036	0.002	3.81E-73	0.035	0.008	9.30E-06
HDLC	rs4871624	T	0.71	0.020	0.002	4.09E-22	-0.019	0.009	2.50E-02
HDLC	rs4875043	A	0.78	0.015	0.002	1.24E-10	0.002	0.009	7.90E-01
HDLC	rs61435086	T	0.99	-0.089	0.009	4.37E-25	0.029	0.035	4.50E-01
HDLC	rs61596977	C	0.86	0.016	0.003	3.51E-09	-0.023	0.011	3.30E-02
HDLC	rs62486442	G	0.67	0.014	0.002	3.24E-12	0.002	0.008	8.00E-01
HDLC	rs62491176	T	0.86	-0.019	0.003	1.15E-12	-0.003	0.011	7.10E-01
HDLC	rs6469605	C	0.43	-0.032	0.002	2.59E-61	0.017	0.008	3.10E-02
HDLC	rs67344323	T	0.74	0.015	0.002	2.08E-11	-0.020	0.009	2.70E-02
HDLC	rs67934334	G	0.37	-0.011	0.002	3.85E-08	0.004	0.008	5.50E-01
HDLC	rs6987377	A	0.42	-0.011	0.002	1.05E-08	0.001	0.008	8.20E-01
HDLC	rs72647336	G	0.94	0.044	0.004	2.07E-23	-0.018	0.018	3.10E-01
HDLC	rs75032664	C	0.99	0.055	0.009	1.51E-09	0.006	0.036	9.40E-01
HDLC	rs75609851	G	0.99	-0.170	0.010	1.67E-69	-0.009	0.040	7.40E-01
HDLC	rs75662196	G	0.97	-0.068	0.006	8.49E-31	-0.005	0.024	8.70E-01
HDLC	rs7817574	T	0.81	-0.033	0.002	4.37E-42	0.002	0.010	9.50E-01
HDLC	rs7826177	T	0.36	-0.011	0.002	1.20E-08	0.014	0.008	1.20E-01
HDLC	rs79153732	C	0.98	0.094	0.007	5.34E-38	0.080	0.030	4.00E-03
HDLC	rs80005209	T	0.97	0.144	0.006	4.95E-144	-0.028	0.023	2.20E-01
HDLC	rs9657541	C	0.80	0.017	0.002	1.88E-13	0.011	0.010	2.00E-01
HDLC	rs9987289	A	0.09	-0.087	0.003	6.57E-155	-0.023	0.014	9.50E-02
HDLC	rs117435644	G	0.76	-0.012	0.002	3.07E-08	-0.001	0.009	9.30E-01
HDLC	rs11789603	C	0.89	-0.069	0.003	6.66E-110	0.010	0.012	3.60E-01
HDLC	rs12686780	C	0.83	0.016	0.003	1.24E-10	-0.007	0.010	4.70E-01

HDLC	rs13300004	A	0.99	0.049	0.009	1.22E-08	-0.006	0.036	8.60E-01
HDLC	rs1411432	A	0.81	0.014	0.002	1.71E-08	-0.007	0.010	4.90E-01
HDLC	rs1412234	T	0.67	0.012	0.002	3.49E-09	-0.004	0.008	7.20E-01
HDLC	rs1567353	C	0.69	0.011	0.002	4.52E-08	-0.004	0.008	7.10E-01
HDLC	rs2066714	T	0.87	-0.047	0.003	4.20E-60	0.009	0.012	6.30E-01
HDLC	rs2297402	C	0.98	0.068	0.007	6.88E-26	0.020	0.027	5.30E-01
HDLC	rs2417125	A	0.72	0.013	0.002	4.32E-10	-0.009	0.009	3.50E-01
HDLC	rs2520096	A	0.73	-0.015	0.002	7.21E-12	-0.005	0.009	5.30E-01
HDLC	rs2740488	A	0.73	0.069	0.002	3.99E-222	-0.009	0.009	3.40E-01
HDLC	rs28712486	C	0.77	-0.015	0.002	5.29E-11	0.014	0.009	1.20E-01
HDLC	rs532436	G	0.82	-0.023	0.002	3.03E-21	-0.022	0.010	2.20E-02
HDLC	rs62565259	C	0.83	-0.016	0.003	6.13E-10	0.011	0.010	2.50E-01
HDLC	rs686030	C	0.14	-0.050	0.003	9.76E-74	-0.010	0.011	4.10E-01
HDLC	rs7036107	A	0.49	0.012	0.002	2.73E-10	-0.011	0.008	9.40E-02
HDLC	rs7039168	A	0.33	-0.012	0.002	1.59E-08	0.001	0.008	9.00E-01
HDLC	rs74500135	T	0.99	-0.067	0.010	2.27E-11	0.029	0.040	4.20E-01
HDLC	rs75572195	G	0.91	-0.023	0.003	4.67E-12	0.016	0.013	2.70E-01
HDLC	rs76530346	G	0.91	0.019	0.003	3.78E-09	0.009	0.013	5.20E-01
HDLC	rs10761737	T	0.58	-0.014	0.002	4.82E-13	-0.002	0.008	9.40E-01
HDLC	rs10786114	C	0.13	-0.024	0.003	1.55E-16	0.007	0.012	6.70E-01
HDLC	rs10826337	G	0.59	0.011	0.002	5.81E-09	-0.003	0.008	6.20E-01
HDLC	rs10901802	G	0.48	-0.011	0.002	1.64E-08	-0.006	0.008	4.50E-01
HDLC	rs11000468	C	0.74	-0.013	0.002	1.49E-08	-0.005	0.009	6.10E-01
HDLC	rs11009262	G	0.94	0.023	0.004	1.08E-08	-0.025	0.017	1.40E-01
HDLC	rs11202156	G	0.73	0.012	0.002	2.57E-08	-0.004	0.009	7.00E-01
HDLC	rs11239536	T	0.76	-0.029	0.002	5.30E-38	0.000	0.009	9.50E-01
HDLC	rs11254464	T	0.58	-0.013	0.002	5.54E-11	-0.015	0.008	5.90E-02
HDLC	rs12411732	G	0.85	0.031	0.003	7.59E-29	0.032	0.011	3.20E-03
HDLC	rs12411959	A	0.78	0.016	0.002	1.50E-11	-0.013	0.009	1.70E-01
HDLC	rs1970811	T	0.54	0.012	0.002	1.49E-09	-0.001	0.008	9.20E-01
HDLC	rs2068888	G	0.55	-0.019	0.002	1.21E-23	0.013	0.008	8.30E-02
HDLC	rs2456722	A	0.40	-0.011	0.002	1.46E-08	0.004	0.008	6.90E-01
HDLC	rs2792751	T	0.27	0.036	0.002	3.29E-64	0.053	0.009	2.10E-09
HDLC	rs2804894	G	0.27	-0.017	0.002	2.20E-15	0.004	0.009	6.60E-01
HDLC	rs2862954	T	0.50	-0.015	0.002	9.68E-15	0.019	0.008	1.80E-02
HDLC	rs577525	T	0.44	0.014	0.002	2.57E-13	-0.011	0.008	8.10E-02
HDLC	rs703966	G	0.58	-0.016	0.002	5.43E-16	0.004	0.008	6.70E-01
HDLC	rs72805692	A	0.88	0.018	0.003	9.41E-10	-0.020	0.012	1.50E-01
HDLC	rs72823020	T	0.87	-0.029	0.003	4.49E-25	-0.010	0.011	3.70E-01
HDLC	rs7903537	C	0.48	0.011	0.002	5.77E-09	-0.006	0.008	4.10E-01
HDLC	rs1052373	C	0.68	-0.045	0.002	1.41E-106	0.008	0.008	3.30E-01
HDLC	rs10750766	C	0.29	0.019	0.002	6.99E-19	-0.009	0.009	3.00E-01
HDLC	rs10896581	G	0.88	0.018	0.003	5.41E-10	-0.022	0.012	5.70E-02
HDLC	rs11021232	T	0.82	0.017	0.002	1.54E-11	-0.014	0.010	1.60E-01
HDLC	rs112192770	C	0.95	-0.029	0.005	3.34E-10	0.007	0.018	6.70E-01
HDLC	rs11231138	C	0.63	0.015	0.002	3.03E-14	-0.006	0.008	4.10E-01
HDLC	rs112771035	C	0.93	0.032	0.004	8.33E-18	0.012	0.015	4.00E-01
HDLC	rs11601507	C	0.93	0.025	0.004	9.16E-12	-0.009	0.015	4.10E-01
HDLC	rs11605837	G	0.69	0.018	0.002	1.13E-17	0.001	0.008	8.80E-01
HDLC	rs116978226	C	0.97	-0.068	0.005	2.23E-36	0.012	0.022	5.60E-01
HDLC	rs12146566	A	0.80	0.014	0.002	1.25E-08	0.006	0.010	5.50E-01
HDLC	rs12803463	G	0.92	-0.024	0.003	1.10E-11	-0.002	0.014	9.20E-01
HDLC	rs140201358	C	0.99	0.058	0.008	1.46E-12	-0.154	0.032	2.70E-06
HDLC	rs141469619	A	0.99	0.204	0.010	4.63E-89	-0.076	0.041	7.10E-02
HDLC	rs143474489	T	0.98	-0.048	0.007	1.18E-13	0.037	0.026	1.50E-01
HDLC	rs16928809	G	0.91	0.026	0.003	1.40E-15	-0.010	0.013	4.00E-01

HDLC	rs17309930	C	0.79	0.022	0.002	1.32E-20	-0.019	0.010	5.30E-02
HDLC	rs174566	A	0.65	0.056	0.002	9.53E-175	0.000	0.008	1.00E+00
HDLC	rs17566828	G	0.96	-0.030	0.005	4.32E-10	0.004	0.019	1.00E+00
HDLC	rs2155220	C	0.56	0.011	0.002	3.23E-08	-0.016	0.008	3.30E-02
HDLC	rs2218793	C	0.72	0.015	0.002	1.11E-12	0.006	0.009	4.80E-01
HDLC	rs28876383	C	0.98	0.052	0.007	4.07E-13	0.046	0.029	1.00E-01
HDLC	rs4356188	C	0.30	0.016	0.002	3.82E-14	0.000	0.008	9.50E-01
HDLC	rs4755720	C	0.39	-0.011	0.002	1.41E-08	0.020	0.008	5.10E-03
HDLC	rs4930352	G	0.51	-0.016	0.002	8.71E-17	0.011	0.008	1.70E-01
HDLC	rs514924	A	0.10	0.018	0.003	2.05E-08	0.019	0.013	1.90E-01
HDLC	rs550916783	G	0.99	0.053	0.010	4.52E-08	-0.034	0.039	3.50E-01
HDLC	rs559355	A	0.84	0.035	0.003	8.14E-41	0.032	0.011	2.80E-03
HDLC	rs56271783	G	0.95	0.056	0.005	2.92E-34	-0.020	0.019	3.40E-01
HDLC	rs567056	G	0.62	0.011	0.002	7.01E-09	-0.018	0.008	2.70E-02
HDLC	rs58473820	C	0.62	-0.026	0.002	7.45E-39	-0.015	0.008	6.10E-02
HDLC	rs61884030	T	0.86	-0.018	0.003	1.27E-10	0.013	0.011	2.60E-01
HDLC	rs6590207	A	0.71	0.015	0.002	8.99E-12	0.000	0.009	8.10E-01
HDLC	rs73457437	G	0.97	0.035	0.005	8.05E-11	0.006	0.022	7.70E-01
HDLC	rs77916918	G	0.97	-0.032	0.005	7.42E-10	0.000	0.021	9.30E-01
HDLC	rs7925100	G	0.60	0.018	0.002	2.08E-21	-0.007	0.008	2.80E-01
HDLC	rs79634051	G	0.97	-0.039	0.006	1.08E-11	0.011	0.023	6.10E-01
HDLC	rs964184	G	0.13	-0.105	0.003	0.00E+00	0.007	0.011	5.10E-01
HDLC	rs1012306	C	0.44	-0.011	0.002	2.87E-08	-0.009	0.008	2.00E-01
HDLC	rs1054852	A	0.62	-0.035	0.002	7.97E-65	0.024	0.008	5.60E-03
HDLC	rs10774439	G	0.19	-0.021	0.002	1.41E-16	0.007	0.010	5.00E-01
HDLC	rs10774579	T	0.52	0.016	0.002	1.61E-17	0.019	0.008	1.20E-02
HDLC	rs10846690	T	0.15	-0.021	0.003	1.31E-15	0.011	0.011	3.30E-01
HDLC	rs10879184	C	0.50	0.011	0.002	1.47E-08	0.004	0.008	6.30E-01
HDLC	rs11043221	A	0.70	-0.013	0.002	4.30E-10	0.003	0.009	7.40E-01
HDLC	rs11045171	A	0.80	-0.028	0.002	5.04E-32	0.010	0.010	2.60E-01
HDLC	rs11170516	G	0.85	0.019	0.003	1.78E-12	0.014	0.011	2.40E-01
HDLC	rs113740515	G	0.79	-0.038	0.002	1.04E-58	0.010	0.010	3.50E-01
HDLC	rs118128493	C	0.95	0.025	0.005	5.05E-08	-0.004	0.018	9.30E-01
HDLC	rs1252425	C	0.34	-0.012	0.002	6.70E-10	0.015	0.008	7.20E-02
HDLC	rs12814794	G	0.25	-0.015	0.002	1.74E-11	0.010	0.009	1.90E-01
HDLC	rs2204886	T	0.48	0.013	0.002	2.24E-11	-0.009	0.008	2.40E-01
HDLC	rs2302367	T	0.51	0.011	0.002	2.73E-08	-0.005	0.008	4.10E-01
HDLC	rs2645979	G	0.64	-0.011	0.002	8.49E-09	0.019	0.008	2.30E-02
HDLC	rs3184504	T	0.48	-0.027	0.002	3.48E-44	0.003	0.008	8.40E-01
HDLC	rs4078216	G	0.76	-0.014	0.002	3.55E-10	0.002	0.009	8.30E-01
HDLC	rs58298943	C	0.92	-0.020	0.003	3.89E-09	0.021	0.014	1.40E-01
HDLC	rs61352607	G	0.76	-0.031	0.002	6.36E-43	0.006	0.009	4.90E-01
HDLC	rs61926301	G	0.60	-0.012	0.002	2.44E-10	0.011	0.008	1.60E-01
HDLC	rs7136506	T	0.78	0.036	0.002	1.84E-53	-0.009	0.010	3.00E-01
HDLC	rs7308864	A	0.48	-0.024	0.002	1.08E-35	-0.004	0.008	7.40E-01
HDLC	rs7316878	T	0.57	-0.012	0.002	1.11E-08	0.004	0.008	6.20E-01
HDLC	rs7488780	G	0.80	-0.015	0.002	8.64E-10	0.015	0.010	7.40E-02
HDLC	rs838777	C	0.86	-0.018	0.003	3.88E-11	-0.002	0.011	7.50E-01
HDLC	rs921919	G	0.33	0.042	0.002	2.99E-90	-0.009	0.008	2.90E-01
HDLC	rs117230571	A	0.92	0.027	0.004	1.97E-13	0.012	0.015	4.10E-01
HDLC	rs183078	A	0.60	-0.012	0.002	1.96E-10	-0.009	0.008	3.30E-01
HDLC	rs374039502	T	0.98	0.047	0.007	1.81E-11	0.065	0.030	3.60E-02
HDLC	rs549058	G	0.88	-0.017	0.003	5.15E-09	0.009	0.012	4.30E-01
HDLC	rs76428106	T	0.99	0.062	0.009	1.73E-12	-0.003	0.035	8.50E-01
HDLC	rs9526023	G	0.97	-0.033	0.006	4.04E-09	0.034	0.023	1.00E-01
HDLC	rs9604045	G	0.75	-0.018	0.002	1.76E-14	0.006	0.009	4.20E-01

HDLC	rs11622947	C	0.46	0.011	0.002	3.43E-08	0.004	0.008	5.70E-01
HDLC	rs13379043	T	0.72	-0.020	0.002	6.31E-20	0.032	0.009	3.50E-04
HDLC	rs17124112	C	0.92	0.021	0.004	4.11E-09	0.002	0.014	9.70E-01
HDLC	rs1760940	A	0.75	-0.012	0.002	4.22E-08	-0.003	0.009	6.00E-01
HDLC	rs1955512	G	0.42	-0.011	0.002	2.34E-08	0.004	0.008	5.90E-01
HDLC	rs2498786	C	0.38	0.025	0.002	4.45E-38	0.003	0.008	6.60E-01
HDLC	rs35828909	A	0.45	0.011	0.002	1.46E-08	0.004	0.008	7.80E-01
HDLC	rs367677	A	0.76	-0.017	0.002	1.03E-13	0.012	0.009	1.90E-01
HDLC	rs3825669	A	0.20	-0.014	0.002	4.65E-09	-0.003	0.010	8.70E-01
HDLC	rs61993685	T	0.92	-0.020	0.004	2.98E-08	0.020	0.014	1.50E-01
HDLC	rs7158166	T	0.41	-0.014	0.002	5.68E-13	0.002	0.008	6.70E-01
HDLC	rs72729582	A	0.93	-0.025	0.004	1.88E-11	0.012	0.015	4.80E-01
HDLC	rs8014289	A	0.44	-0.015	0.002	5.76E-15	0.005	0.008	5.60E-01
HDLC	rs9646167	C	0.48	0.014	0.002	3.35E-13	-0.002	0.008	7.90E-01
HDLC	rs10162642	G	0.79	0.048	0.002	2.26E-93	-0.008	0.010	4.30E-01
HDLC	rs10438354	C	0.65	-0.012	0.002	4.67E-09	0.012	0.008	2.10E-01
HDLC	rs11631178	T	0.90	-0.021	0.003	1.55E-11	0.013	0.013	2.40E-01
HDLC	rs147525635	G	0.44	0.013	0.002	3.64E-12	-0.009	0.008	1.80E-01
HDLC	rs150844304	A	0.97	0.090	0.006	1.99E-51	-0.027	0.025	2.80E-01
HDLC	rs1800588	C	0.78	-0.118	0.002	0.00E+00	0.009	0.009	3.40E-01
HDLC	rs1818917	C	0.49	-0.011	0.002	2.33E-09	0.007	0.008	2.90E-01
HDLC	rs200540247	A	0.29	-0.013	0.002	3.31E-10	-0.003	0.009	7.40E-01
HDLC	rs2245477	C	0.63	0.011	0.002	8.07E-09	-0.011	0.008	1.70E-01
HDLC	rs2414178	T	0.77	-0.013	0.002	2.48E-08	0.017	0.009	4.80E-02
HDLC	rs2663924	T	0.25	-0.012	0.002	2.92E-08	-0.001	0.009	9.40E-01
HDLC	rs28362901	C	0.91	0.024	0.003	1.03E-12	0.008	0.014	6.60E-01
HDLC	rs28510484	G	0.83	0.015	0.003	1.26E-09	-0.010	0.010	2.40E-01
HDLC	rs34675318	G	0.71	0.017	0.002	3.75E-15	-0.010	0.009	2.10E-01
HDLC	rs3803501	G	0.43	-0.018	0.002	2.75E-20	-0.015	0.008	4.70E-02
HDLC	rs7170463	A	0.69	-0.019	0.002	5.49E-20	0.005	0.008	6.70E-01
HDLC	rs11075985	C	0.58	0.015	0.002	1.76E-14	-0.021	0.008	7.20E-03
HDLC	rs113710278	C	0.99	-0.055	0.008	4.83E-12	-0.024	0.033	4.30E-01
HDLC	rs11640494	G	0.54	0.015	0.002	4.67E-16	-0.006	0.008	4.40E-01
HDLC	rs11645157	T	0.51	-0.019	0.002	4.76E-23	0.012	0.008	1.70E-01
HDLC	rs12926854	A	0.73	-0.012	0.002	6.99E-09	-0.004	0.009	6.50E-01
HDLC	rs12928099	C	0.70	-0.021	0.002	4.57E-25	0.015	0.008	9.80E-02
HDLC	rs138026745	G	0.97	-0.034	0.006	4.71E-08	0.002	0.026	9.30E-01
HDLC	rs142493909	T	0.98	-0.067	0.007	1.07E-21	0.033	0.031	4.00E-01
HDLC	rs2925979	T	0.30	-0.037	0.002	1.86E-73	0.019	0.008	2.20E-02
HDLC	rs34830321	C	0.99	0.048	0.008	5.62E-09	0.018	0.034	6.60E-01
HDLC	rs3814883	C	0.52	0.015	0.002	7.28E-16	-0.001	0.008	8.40E-01
HDLC	rs545858676	A	0.96	0.098	0.006	1.18E-58	0.017	0.024	5.40E-01
HDLC	rs55781197	A	0.88	-0.059	0.003	2.65E-88	0.004	0.012	7.80E-01
HDLC	rs6499102	A	0.06	-0.035	0.004	1.79E-18	-0.010	0.016	5.80E-01
HDLC	rs7188071	T	0.35	-0.011	0.002	3.11E-08	0.018	0.008	2.70E-02
HDLC	rs75152587	G	0.99	0.094	0.009	1.14E-28	-0.051	0.035	1.70E-01
HDLC	rs79311290	A	0.89	0.018	0.003	8.65E-09	-0.010	0.013	4.50E-01
HDLC	rs79600951	C	0.91	0.107	0.003	2.04E-235	-0.022	0.013	1.30E-01
HDLC	rs79616633	C	0.98	0.036	0.006	2.13E-08	-0.014	0.026	7.20E-01
HDLC	rs9989419	A	0.39	-0.144	0.002	0.00E+00	0.014	0.008	7.30E-02
HDLC	rs112001035	G	0.94	0.047	0.004	2.79E-30	0.033	0.017	5.10E-02
HDLC	rs11653260	A	0.79	0.013	0.002	8.70E-09	0.007	0.009	4.40E-01
HDLC	rs11658786	G	0.33	-0.031	0.002	1.52E-51	0.025	0.008	4.00E-03
HDLC	rs11658872	C	0.94	0.033	0.004	1.18E-15	-0.006	0.017	6.90E-01
HDLC	rs117499775	T	0.96	0.028	0.005	5.88E-09	-0.044	0.020	1.90E-02
HDLC	rs141062196	G	0.81	0.019	0.002	5.70E-15	0.002	0.010	8.10E-01

HDLC	rs16975758	G	0.62	-0.012	0.002	5.03E-09	-0.003	0.008	5.60E-01
HDLC	rs2011614	G	0.65	0.014	0.002	4.24E-13	-0.007	0.008	4.40E-01
HDLC	rs2071379	A	0.40	0.013	0.002	4.24E-12	0.002	0.008	9.10E-01
HDLC	rs2314338	T	0.73	0.016	0.002	2.94E-13	0.005	0.009	5.60E-01
HDLC	rs2585398	C	0.45	0.013	0.002	1.83E-11	0.003	0.008	8.80E-01
HDLC	rs34138141	G	0.72	0.018	0.002	1.51E-16	-0.008	0.009	3.40E-01
HDLC	rs3794752	T	0.72	-0.013	0.002	1.97E-09	-0.003	0.009	7.80E-01
HDLC	rs4969141	C	0.51	-0.030	0.002	3.44E-54	0.014	0.008	6.60E-02
HDLC	rs595767	A	0.48	0.014	0.002	5.30E-14	-0.003	0.008	5.90E-01
HDLC	rs7218647	G	0.44	-0.011	0.002	1.74E-08	0.002	0.008	6.40E-01
HDLC	rs72836561	C	0.97	0.171	0.005	2.18E-212	-0.012	0.022	6.10E-01
HDLC	rs74456742	G	0.96	-0.036	0.005	2.18E-12	0.026	0.021	1.90E-01
HDLC	rs8081548	T	0.34	-0.018	0.002	7.87E-20	0.020	0.008	1.50E-02
HDLC	rs11664369	C	0.73	0.023	0.002	1.45E-26	0.006	0.009	5.40E-01
HDLC	rs117687565	C	0.99	-0.083	0.009	1.23E-19	0.036	0.038	4.10E-01
HDLC	rs150237291	T	0.98	-0.045	0.007	5.59E-12	-0.055	0.026	3.90E-02
HDLC	rs2298624	C	0.87	-0.030	0.003	1.02E-26	-0.016	0.011	1.30E-01
HDLC	rs2435307	C	0.51	-0.016	0.002	3.32E-17	0.006	0.008	4.90E-01
HDLC	rs4800356	C	0.71	0.016	0.002	1.61E-12	-0.004	0.009	4.10E-01
HDLC	rs56017932	G	0.80	-0.015	0.003	8.02E-09	0.017	0.011	9.00E-02
HDLC	rs62092069	G	0.63	-0.013	0.002	3.78E-11	0.020	0.008	1.20E-02
HDLC	rs680321	T	0.54	-0.011	0.002	1.35E-08	0.000	0.008	9.60E-01
HDLC	rs73455693	G	0.96	-0.028	0.005	3.67E-08	0.003	0.021	8.90E-01
HDLC	rs77960347	A	0.99	-0.291	0.008	3.87E-269	-0.076	0.033	2.60E-02
HDLC	rs8086351	C	0.18	-0.084	0.003	1.80E-245	-0.007	0.010	4.90E-01
HDLC	rs116843064	G	0.98	-0.206	0.007	6.37E-195	-0.005	0.028	8.80E-01
HDLC	rs11878235	G	0.41	-0.013	0.002	8.25E-11	0.021	0.008	9.10E-03
HDLC	rs11880219	T	0.82	0.021	0.002	4.85E-17	0.006	0.010	4.50E-01
HDLC	rs12150914	T	0.40	0.015	0.002	1.68E-14	-0.003	0.008	6.80E-01
HDLC	rs12975319	G	0.70	0.014	0.002	7.11E-11	-0.021	0.008	6.80E-03
HDLC	rs12976395	G	0.50	-0.041	0.002	1.26E-90	-0.008	0.008	3.50E-01
HDLC	rs144311893	C	0.98	-0.081	0.007	8.71E-34	-0.025	0.028	3.70E-01
HDLC	rs1468642	T	0.65	0.017	0.002	7.61E-18	-0.004	0.008	5.50E-01
HDLC	rs15052	T	0.82	-0.016	0.003	1.60E-10	0.013	0.010	2.10E-01
HDLC	rs2289863	C	0.25	0.018	0.002	6.03E-16	-0.008	0.009	4.20E-01
HDLC	rs2965169	A	0.61	-0.012	0.002	5.46E-10	-0.025	0.008	3.50E-03
HDLC	rs34559316	C	0.81	0.014	0.002	1.62E-08	-0.015	0.010	1.40E-01
HDLC	rs35137994	C	0.94	-0.046	0.004	3.41E-29	0.002	0.017	9.10E-01
HDLC	rs36092527	T	0.88	0.017	0.003	1.49E-08	-0.001	0.012	9.30E-01
HDLC	rs367070	A	0.77	-0.042	0.002	1.36E-74	0.001	0.009	8.80E-01
HDLC	rs429358	T	0.85	0.075	0.003	7.21E-180	0.121	0.011	1.50E-29
HDLC	rs4760	A	0.84	0.019	0.003	3.67E-13	0.014	0.011	2.00E-01
HDLC	rs59737437	C	0.73	-0.012	0.002	4.22E-08	0.004	0.009	6.50E-01
HDLC	rs62102718	A	0.71	0.024	0.002	2.91E-29	-0.012	0.009	1.60E-01
HDLC	rs62112763	C	0.56	0.014	0.002	2.40E-13	-0.022	0.008	8.60E-04
HDLC	rs7251640	T	0.81	-0.014	0.002	4.27E-09	0.008	0.010	5.60E-01
HDLC	rs737338	C	0.96	0.089	0.005	1.40E-66	-0.017	0.021	4.60E-01
HDLC	rs76213248	C	0.59	-0.013	0.002	6.95E-12	-0.013	0.008	9.60E-02
HDLC	rs76246107	G	0.92	0.021	0.004	7.46E-09	0.005	0.015	6.80E-01
HDLC	rs8102873	C	0.42	0.011	0.002	1.42E-08	0.003	0.008	7.70E-01
HDLC	rs1132274	C	0.85	0.022	0.003	1.23E-16	0.007	0.011	5.40E-01
HDLC	rs144033177	A	0.98	0.056	0.008	1.07E-12	-0.087	0.031	4.90E-03
HDLC	rs1800961	C	0.97	0.139	0.006	1.44E-140	-0.008	0.022	6.20E-01
HDLC	rs1884589	A	0.43	0.013	0.002	1.52E-11	-0.010	0.008	1.90E-01
HDLC	rs3736802	T	0.48	-0.017	0.002	1.10E-18	-0.005	0.008	5.60E-01
HDLC	rs3859588	T	0.78	0.013	0.002	1.04E-08	-0.011	0.009	2.80E-01

HDLC	rs4239651	T	0.21	-0.026	0.002	3.72E-28	-0.019	0.009	5.70E-02
HDLC	rs6021914	T	0.63	-0.012	0.002	1.20E-09	0.018	0.008	2.20E-02
HDLC	rs6059958	C	0.83	-0.015	0.003	9.77E-09	-0.012	0.010	2.40E-01
HDLC	rs6073958	T	0.80	0.061	0.002	6.45E-143	-0.001	0.010	9.50E-01
HDLC	rs6123685	G	0.75	-0.016	0.002	3.15E-13	0.011	0.009	1.40E-01
HDLC	rs6131012	A	0.60	-0.011	0.002	9.64E-09	0.008	0.008	3.10E-01
HDLC	rs76602912	T	0.98	0.043	0.006	4.22E-12	0.008	0.026	7.90E-01
HDLC	rs8126001	C	0.51	-0.012	0.002	1.57E-09	0.008	0.008	2.30E-01
HDLC	rs235314	C	0.47	0.018	0.002	2.31E-20	-0.001	0.008	7.20E-01
HDLC	rs3746915	A	0.42	-0.011	0.002	1.98E-08	0.003	0.008	7.70E-01
HDLC	rs407133	G	0.44	0.011	0.002	4.77E-08	-0.013	0.008	9.40E-02
HDLC	rs8127283	G	0.51	0.012	0.002	5.23E-09	0.003	0.009	7.30E-01
HDLC	rs9980311	A	0.74	0.015	0.002	1.16E-11	-0.001	0.009	8.80E-01
HDLC	rs133015	C	0.56	-0.021	0.002	1.47E-26	0.000	0.008	9.40E-01
HDLC	rs1894544	G	0.55	0.011	0.002	5.19E-09	-0.007	0.008	3.00E-01
HDLC	rs2256609	A	0.81	0.033	0.002	2.32E-41	0.023	0.010	1.90E-02
HDLC	rs55652051	A	0.77	0.015	0.002	4.55E-11	-0.022	0.009	2.50E-02
HDLC	rs6002946	G	0.32	-0.012	0.002	1.31E-08	0.005	0.008	5.50E-01
HDLC	rs738409	C	0.78	0.014	0.002	5.14E-10	-0.195	0.009	5.60E-95
HDLC	rs9608972	T	0.76	0.017	0.002	1.56E-14	-0.003	0.009	6.40E-01
HDLC	rs9610329	C	0.57	0.011	0.002	1.66E-08	-0.025	0.008	1.30E-03
HDLC	rs9622830	C	0.65	0.016	0.002	4.74E-16	-0.004	0.008	6.60E-01
LDLC	rs10910476	C	0.44	-0.012	0.002	4.33E-09	0.003	0.008	7.50E-01
LDLC	rs11206517	T	0.97	-0.068	0.006	5.90E-32	0.024	0.022	3.30E-01
LDLC	rs11206788	G	0.41	-0.014	0.002	1.99E-10	0.005	0.008	5.90E-01
LDLC	rs114165349	G	0.98	-0.056	0.007	8.04E-16	-0.027	0.026	2.70E-01
LDLC	rs115458560	T	0.98	0.050	0.008	6.62E-11	-0.018	0.029	4.90E-01
LDLC	rs11591147	G	0.98	0.349	0.008	0.00E+00	-0.027	0.029	3.10E-01
LDLC	rs1168127	C	0.35	-0.039	0.002	1.87E-70	0.004	0.008	7.70E-01
LDLC	rs12078100	C	0.38	-0.013	0.002	9.32E-10	-0.001	0.008	7.20E-01
LDLC	rs140584594	A	0.27	0.014	0.002	5.80E-10	-0.039	0.009	1.20E-05
LDLC	rs1556562	G	0.21	-0.019	0.002	1.99E-14	0.002	0.009	6.80E-01
LDLC	rs2642438	A	0.30	-0.025	0.002	7.59E-29	-0.052	0.008	1.70E-09
LDLC	rs28631087	T	0.79	0.016	0.003	2.17E-10	-0.008	0.009	4.00E-01
LDLC	rs472495	G	0.35	-0.043	0.002	5.51E-85	0.003	0.008	7.90E-01
LDLC	rs4970834	C	0.81	0.105	0.003	0.00E+00	-0.012	0.010	1.50E-01
LDLC	rs541155877	C	0.99	0.069	0.011	6.30E-10	-0.021	0.041	5.10E-01
LDLC	rs556107	C	0.48	-0.035	0.002	8.04E-64	-0.016	0.008	5.60E-02
LDLC	rs61775180	C	0.58	0.026	0.002	1.67E-35	-0.008	0.008	3.20E-01
LDLC	rs61778883	T	0.13	-0.019	0.003	2.37E-09	0.007	0.012	4.80E-01
LDLC	rs6667939	C	0.28	-0.015	0.002	7.06E-11	-0.002	0.009	6.90E-01
LDLC	rs6693893	T	0.96	0.075	0.006	3.11E-40	0.015	0.021	5.40E-01
LDLC	rs77257036	C	0.65	0.012	0.002	3.79E-08	-0.009	0.008	3.40E-01
LDLC	rs880315	T	0.66	0.015	0.002	6.83E-12	0.011	0.008	7.60E-02
LDLC	rs11568318	C	0.93	-0.026	0.004	7.39E-10	-0.002	0.015	7.50E-01
LDLC	rs12471768	T	0.30	-0.014	0.002	2.47E-09	-0.018	0.008	2.30E-02
LDLC	rs1250258	C	0.26	-0.014	0.002	6.80E-09	-0.004	0.009	6.00E-01
LDLC	rs1260326	T	0.40	0.035	0.002	5.58E-60	0.039	0.008	4.10E-07
LDLC	rs12990177	A	0.48	-0.015	0.002	1.07E-12	-0.004	0.008	7.00E-01
LDLC	rs150474434	G	0.90	0.035	0.003	1.42E-23	-0.027	0.013	3.60E-02
LDLC	rs17050272	G	0.59	0.021	0.002	3.14E-22	-0.005	0.008	4.90E-01
LDLC	rs1731243	C	0.39	-0.013	0.002	7.07E-10	-0.006	0.008	3.00E-01
LDLC	rs2287622	A	0.40	0.021	0.002	3.20E-23	0.000	0.008	8.20E-01
LDLC	rs2718717	A	0.86	-0.019	0.003	2.64E-10	0.000	0.011	9.40E-01
LDLC	rs4299376	G	0.32	0.054	0.002	3.47E-131	0.011	0.008	1.20E-01
LDLC	rs430096	A	0.78	0.054	0.003	2.35E-103	-0.004	0.009	5.80E-01

LDLC	rs4954192	C	0.63	-0.015	0.002	5.69E-12	-0.003	0.008	6.40E-01
LDLC	rs56236159	T	0.87	-0.018	0.003	7.04E-09	0.006	0.011	6.50E-01
LDLC	rs6709904	A	0.89	0.044	0.003	8.74E-40	0.006	0.012	6.80E-01
LDLC	rs72774870	C	0.93	0.022	0.004	3.88E-08	0.012	0.015	4.50E-01
LDLC	rs7562734	G	0.68	0.020	0.002	2.24E-19	-0.009	0.008	2.60E-01
LDLC	rs7569317	T	0.47	-0.018	0.002	8.70E-18	-0.008	0.008	2.40E-01
LDLC	rs907866	G	0.55	0.018	0.002	3.73E-17	0.011	0.008	2.40E-01
LDLC	rs934197	G	0.66	-0.083	0.002	0.00E+00	0.005	0.008	6.20E-01
LDLC	rs113177823	G	0.95	0.041	0.005	1.24E-18	-0.031	0.017	6.10E-02
LDLC	rs13076933	T	0.74	0.021	0.002	3.03E-18	0.007	0.009	4.70E-01
LDLC	rs3732359	G	0.22	0.017	0.003	4.74E-12	0.003	0.009	6.10E-01
LDLC	rs3932048	C	0.68	-0.014	0.002	5.75E-10	-0.013	0.008	1.30E-01
LDLC	rs55921103	G	0.35	-0.014	0.002	5.07E-10	-0.002	0.008	6.60E-01
LDLC	rs71311871	A	0.92	0.028	0.004	6.65E-14	0.018	0.014	3.10E-01
LDLC	rs9832727	C	0.66	0.015	0.002	2.06E-11	0.006	0.008	3.20E-01
LDLC	rs9834932	A	0.91	0.032	0.004	1.11E-18	-0.008	0.014	5.50E-01
LDLC	rs9841897	T	0.84	-0.019	0.003	1.31E-10	0.009	0.011	3.90E-01
LDLC	rs1229984	T	0.03	-0.053	0.006	7.41E-17	-0.158	0.025	7.00E-10
LDLC	rs13107325	C	0.93	0.025	0.004	6.02E-10	-0.011	0.015	4.00E-01
LDLC	rs13108218	A	0.39	0.018	0.002	2.07E-16	0.005	0.008	5.60E-01
LDLC	rs1458038	C	0.71	0.019	0.002	6.94E-16	0.000	0.009	9.20E-01
LDLC	rs576573069	T	0.33	-0.014	0.002	2.86E-09	-0.015	0.008	8.30E-02
LDLC	rs9884390	T	0.77	-0.025	0.002	7.16E-24	-0.013	0.009	1.50E-01
LDLC	rs990619	C	0.48	0.012	0.002	1.85E-08	0.003	0.008	5.50E-01
LDLC	rs1016988	T	0.81	0.017	0.003	5.19E-11	0.008	0.010	4.00E-01
LDLC	rs116734477	C	0.96	0.047	0.005	2.76E-19	0.018	0.019	3.50E-01
LDLC	rs12916	T	0.60	-0.062	0.002	2.23E-187	0.009	0.008	2.40E-01
LDLC	rs146433259	C	0.99	0.057	0.010	4.57E-08	0.019	0.038	7.40E-01
LDLC	rs2925677	C	0.79	0.019	0.003	1.09E-13	0.006	0.009	5.70E-01
LDLC	rs6874202	T	0.37	-0.032	0.002	1.24E-50	0.018	0.008	2.40E-02
LDLC	rs7734476	G	0.45	-0.019	0.002	3.93E-19	-0.001	0.008	9.70E-01
LDLC	rs9686661	C	0.80	-0.015	0.003	1.13E-08	-0.041	0.010	2.70E-05
LDLC	rs117733303	A	0.98	-0.084	0.008	2.48E-27	0.048	0.029	1.20E-01
LDLC	rs118039278	G	0.92	-0.084	0.004	2.23E-102	0.007	0.014	6.80E-01
LDLC	rs12208357	C	0.93	-0.057	0.004	3.44E-44	0.057	0.015	8.20E-05
LDLC	rs146534110	G	0.99	-0.068	0.009	8.69E-14	0.013	0.033	7.20E-01
LDLC	rs3179865	G	0.60	-0.021	0.002	1.89E-19	-0.014	0.009	1.00E-01
LDLC	rs34392107	G	0.88	-0.046	0.003	5.78E-45	0.008	0.012	4.30E-01
LDLC	rs34568880	C	0.99	-0.056	0.009	6.12E-10	0.018	0.035	6.70E-01
LDLC	rs3822855	G	0.60	-0.018	0.002	3.49E-17	0.016	0.008	5.70E-02
LDLC	rs73025516	A	0.96	0.031	0.005	9.23E-10	-0.013	0.019	5.30E-01
LDLC	rs76967117	G	0.89	0.028	0.003	2.06E-17	-0.003	0.012	7.70E-01
LDLC	rs7746081	G	0.70	0.023	0.002	4.59E-25	0.004	0.008	5.10E-01
LDLC	rs7776054	A	0.74	0.016	0.002	7.05E-12	-0.006	0.009	4.10E-01
LDLC	rs79220007	T	0.92	0.057	0.004	1.54E-48	-0.044	0.015	1.80E-03
LDLC	rs913499	A	0.49	0.012	0.002	9.75E-09	-0.002	0.008	9.00E-01
LDLC	rs9471975	T	0.42	0.013	0.002	1.11E-09	-0.006	0.008	3.70E-01
LDLC	rs9491699	C	0.52	-0.014	0.002	1.93E-11	-0.016	0.008	6.10E-02
LDLC	rs9496567	G	0.76	0.018	0.002	5.85E-13	-0.013	0.009	1.80E-01
LDLC	rs10231941	T	0.82	-0.020	0.003	3.04E-13	-0.009	0.010	2.70E-01
LDLC	rs111338114	A	0.94	0.029	0.005	4.67E-09	-0.024	0.019	2.20E-01
LDLC	rs112758337	G	0.81	0.016	0.003	1.64E-09	-0.001	0.010	9.40E-01
LDLC	rs2073547	A	0.82	-0.036	0.003	2.44E-40	-0.014	0.010	1.80E-01
LDLC	rs28406917	C	0.57	-0.012	0.002	1.76E-08	0.001	0.008	9.10E-01
LDLC	rs4148826	T	0.82	0.016	0.003	6.51E-09	0.006	0.010	4.10E-01
LDLC	rs4722551	T	0.84	-0.024	0.003	1.30E-17	0.016	0.011	8.70E-02

LDLC	rs56130071	G	0.78	-0.033	0.003	4.75E-39	0.007	0.009	3.60E-01
LDLC	rs67050321	T	0.70	-0.015	0.002	2.25E-11	-0.015	0.009	7.20E-02
LDLC	rs836550	A	0.59	-0.012	0.002	4.65E-08	-0.004	0.008	5.30E-01
LDLC	rs869412	T	0.77	0.014	0.003	1.17E-08	0.006	0.009	4.80E-01
LDLC	rs117139027	G	0.98	0.057	0.008	7.20E-13	-0.013	0.030	5.50E-01
LDLC	rs11786083	G	0.63	-0.016	0.002	7.12E-13	0.013	0.008	1.40E-01
LDLC	rs1350559	C	0.60	-0.014	0.002	1.48E-10	0.004	0.008	5.40E-01
LDLC	rs1495741	G	0.22	0.017	0.003	4.62E-12	-0.008	0.009	3.70E-01
LDLC	rs2737265	A	0.72	0.020	0.002	1.88E-18	0.002	0.009	6.70E-01
LDLC	rs28601761	C	0.58	0.062	0.002	1.15E-184	0.047	0.008	2.00E-09
LDLC	rs28768427	G	0.48	-0.012	0.002	1.26E-08	-0.011	0.008	1.40E-01
LDLC	rs4620259	A	0.81	-0.015	0.003	4.65E-08	-0.007	0.010	5.00E-01
LDLC	rs4738684	A	0.34	0.032	0.002	3.33E-46	0.014	0.008	5.70E-02
LDLC	rs59328596	G	0.85	0.018	0.003	8.93E-10	0.001	0.011	8.00E-01
LDLC	rs72638977	A	0.97	0.033	0.006	1.33E-08	-0.011	0.021	7.50E-01
LDLC	rs9298506	A	0.79	-0.019	0.003	2.52E-13	-0.001	0.010	9.80E-01
LDLC	rs9987289	A	0.09	-0.045	0.004	4.79E-36	-0.023	0.014	9.50E-02
LDLC	rs10448340	T	0.68	0.015	0.002	1.41E-11	0.015	0.008	7.50E-02
LDLC	rs115478735	A	0.82	-0.056	0.003	8.98E-96	-0.023	0.010	2.00E-02
LDLC	rs11789603	C	0.89	-0.025	0.003	1.21E-13	0.010	0.012	3.60E-01
LDLC	rs13283282	C	0.85	0.021	0.003	2.62E-12	-0.028	0.011	2.70E-02
LDLC	rs2066714	T	0.87	-0.021	0.003	1.59E-11	0.009	0.012	6.30E-01
LDLC	rs2740488	A	0.73	0.025	0.002	7.77E-27	-0.009	0.009	3.40E-01
LDLC	rs3780181	A	0.93	0.028	0.004	2.88E-11	0.004	0.016	6.50E-01
LDLC	rs6475606	C	0.52	0.020	0.002	1.98E-22	0.003	0.008	7.30E-01
LDLC	rs6560499	G	0.42	0.012	0.002	8.07E-09	-0.011	0.008	1.70E-01
LDLC	rs11014204	C	0.72	-0.014	0.002	3.28E-09	-0.016	0.009	3.90E-02
LDLC	rs12246352	A	0.90	-0.026	0.003	6.77E-14	0.004	0.013	7.60E-01
LDLC	rs17476364	T	0.89	0.022	0.003	9.05E-11	-0.020	0.012	1.60E-01
LDLC	rs2068888	G	0.55	0.019	0.002	5.27E-20	0.013	0.008	8.30E-02
LDLC	rs2250802	G	0.28	0.018	0.002	5.99E-15	0.054	0.009	1.40E-09
LDLC	rs7090758	T	0.53	0.012	0.002	1.61E-08	0.012	0.008	1.80E-01
LDLC	rs72823020	T	0.87	0.021	0.003	5.62E-12	-0.010	0.011	3.70E-01
LDLC	rs79828839	C	0.80	-0.015	0.003	2.44E-08	-0.013	0.010	2.10E-01
LDLC	rs10832963	T	0.26	-0.017	0.002	4.10E-13	0.003	0.009	7.10E-01
LDLC	rs11227247	A	0.87	-0.018	0.003	5.74E-09	0.005	0.011	7.30E-01
LDLC	rs115739682	T	0.81	0.016	0.003	1.39E-09	0.005	0.010	6.20E-01
LDLC	rs11601507	C	0.93	-0.032	0.004	1.69E-15	-0.009	0.015	4.10E-01
LDLC	rs174564	A	0.65	0.032	0.002	3.11E-48	0.000	0.008	9.90E-01
LDLC	rs59379014	C	0.93	-0.055	0.004	3.25E-43	0.011	0.015	4.30E-01
LDLC	rs6589939	A	0.62	-0.013	0.002	7.00E-10	-0.015	0.008	5.60E-02
LDLC	rs7108486	T	0.98	0.039	0.007	1.84E-08	-0.007	0.026	6.60E-01
LDLC	rs74869459	T	0.76	0.017	0.002	2.97E-12	0.018	0.009	6.60E-02
LDLC	rs964184	G	0.13	0.057	0.003	1.61E-78	0.007	0.011	5.10E-01
LDLC	rs1007938	A	0.59	-0.012	0.002	1.26E-08	0.017	0.008	1.60E-02
LDLC	rs112403212	C	0.86	-0.017	0.003	1.20E-08	0.003	0.011	8.30E-01
LDLC	rs1169294	G	0.69	-0.025	0.002	2.00E-27	-0.014	0.008	7.30E-02
LDLC	rs2160994	T	0.35	-0.018	0.002	4.07E-17	0.015	0.008	9.90E-02
LDLC	rs35882350	A	0.74	-0.014	0.002	5.00E-09	-0.015	0.009	9.70E-02
LDLC	rs597808	A	0.48	-0.027	0.002	2.00E-38	0.005	0.008	6.80E-01
LDLC	rs61754230	C	0.98	-0.043	0.007	1.17E-08	-0.104	0.028	4.60E-04
LDLC	rs2238162	C	0.48	0.016	0.002	3.45E-15	-0.014	0.008	6.70E-02
LDLC	rs551473284	T	0.37	-0.013	0.002	1.39E-09	-0.018	0.008	1.40E-02
LDLC	rs6602912	T	0.72	-0.022	0.002	5.48E-22	0.001	0.009	9.90E-01
LDLC	rs11621792	C	0.55	-0.019	0.002	1.29E-19	-0.023	0.008	2.60E-03
LDLC	rs145730801	T	0.96	-0.036	0.005	3.57E-12	0.020	0.019	2.60E-01

LDLC	rs61988556	T	0.91	0.022	0.004	2.11E-09	-0.018	0.014	1.90E-01
LDLC	rs6573971	G	0.44	0.013	0.002	3.41E-10	0.003	0.008	7.00E-01
LDLC	rs8008068	A	0.84	-0.016	0.003	3.68E-08	0.005	0.011	6.50E-01
LDLC	rs10851478	T	0.58	0.012	0.002	6.01E-09	0.003	0.008	5.30E-01
LDLC	rs112987086	G	0.28	-0.016	0.002	1.15E-11	-0.004	0.009	7.00E-01
LDLC	rs1532085	A	0.39	0.017	0.002	8.91E-16	-0.013	0.008	8.90E-02
LDLC	rs261334	G	0.21	0.022	0.003	8.67E-18	-0.009	0.009	3.20E-01
LDLC	rs62011285	T	0.66	-0.012	0.002	1.59E-08	0.015	0.008	7.60E-02
LDLC	rs72733928	A	0.94	-0.026	0.004	2.30E-09	-0.007	0.017	6.80E-01
LDLC	rs12445804	G	0.93	-0.023	0.004	7.97E-09	-0.034	0.015	2.10E-02
LDLC	rs34042070	C	0.81	-0.049	0.003	2.23E-73	-0.010	0.010	4.30E-01
LDLC	rs3764261	C	0.68	0.033	0.002	1.02E-49	-0.004	0.008	6.00E-01
LDLC	rs4782568	C	0.55	0.016	0.002	5.98E-15	0.025	0.008	1.10E-03
LDLC	rs62033400	A	0.61	0.014	0.002	1.15E-11	-0.025	0.008	1.40E-03
LDLC	rs7202323	T	0.77	0.026	0.002	7.52E-25	0.002	0.009	8.10E-01
LDLC	rs77013160	G	0.98	-0.043	0.008	1.73E-08	-0.029	0.030	2.50E-01
LDLC	rs9929977	T	0.63	-0.017	0.002	1.03E-14	-0.008	0.008	3.30E-01
LDLC	rs12603885	G	0.30	-0.018	0.002	1.39E-14	-0.002	0.008	8.40E-01
LDLC	rs12936113	T	0.24	-0.014	0.002	2.27E-08	0.004	0.009	6.00E-01
LDLC	rs12948394	C	0.52	0.018	0.002	5.13E-18	-0.012	0.008	1.10E-01
LDLC	rs1801689	A	0.97	-0.062	0.006	1.60E-24	-0.109	0.023	9.40E-07
LDLC	rs36043200	G	0.48	0.026	0.002	1.21E-36	0.011	0.008	1.70E-01
LDLC	rs55714927	C	0.81	0.026	0.003	3.66E-23	0.004	0.010	8.10E-01
LDLC	rs72631343	C	0.87	0.029	0.003	4.87E-21	0.015	0.012	2.40E-01
LDLC	rs77542162	A	0.98	-0.128	0.007	5.46E-74	0.065	0.026	1.80E-02
LDLC	rs9894946	A	0.16	0.018	0.003	1.48E-09	0.020	0.011	5.80E-02
LDLC	rs7241918	G	0.18	-0.016	0.003	1.23E-08	-0.006	0.010	5.50E-01
LDLC	rs77960347	A	0.99	-0.071	0.009	7.50E-15	-0.076	0.033	2.60E-02
LDLC	rs143020224	C	0.88	0.169	0.003	0.00E+00	-0.019	0.012	1.40E-01
LDLC	rs1551891	G	0.91	0.174	0.004	0.00E+00	-0.023	0.014	1.00E-01
LDLC	rs188247550	C	0.99	0.121	0.010	9.20E-37	-0.252	0.034	3.20E-14
LDLC	rs2021092	T	0.81	0.016	0.003	8.71E-10	-0.012	0.010	2.20E-01
LDLC	rs204469	A	0.04	-0.029	0.005	8.59E-09	0.056	0.019	2.80E-03
LDLC	rs2738447	A	0.41	-0.042	0.002	3.47E-89	-0.007	0.008	3.50E-01
LDLC	rs35081008	C	0.85	0.032	0.003	1.05E-27	-0.018	0.011	1.20E-01
LDLC	rs4452060	C	0.58	-0.083	0.002	0.00E+00	0.017	0.008	3.60E-02
LDLC	rs4808360	T	0.49	-0.013	0.002	8.12E-10	0.005	0.008	6.40E-01
LDLC	rs516316	G	0.49	-0.030	0.002	9.52E-47	-0.013	0.008	1.10E-01
LDLC	rs549956721	A	0.97	0.042	0.007	1.80E-10	0.015	0.024	5.80E-01
LDLC	rs56113850	T	0.42	-0.013	0.002	1.84E-10	-0.012	0.008	1.40E-01
LDLC	rs62116889	T	0.93	0.050	0.004	2.36E-33	0.001	0.016	8.10E-01
LDLC	rs62118464	G	0.88	-0.028	0.003	4.88E-17	0.010	0.012	3.30E-01
LDLC	rs62120394	G	0.71	-0.016	0.002	5.49E-12	-0.039	0.009	4.40E-06
LDLC	rs6857	C	0.83	-0.158	0.003	0.00E+00	0.105	0.010	2.10E-24
LDLC	rs8101801	C	0.96	0.045	0.006	6.16E-16	-0.015	0.021	5.20E-01
LDLC	rs8107974	A	0.92	0.105	0.004	1.77E-158	-0.288	0.015	6.10E-85
LDLC	rs1800961	C	0.97	0.060	0.006	4.04E-23	-0.008	0.022	6.20E-01
LDLC	rs1883711	G	0.97	-0.103	0.006	1.37E-63	-0.068	0.023	1.80E-03
LDLC	rs224424	A	0.79	0.021	0.003	2.58E-16	-0.013	0.010	2.00E-01
LDLC	rs2256814	G	0.80	-0.015	0.003	6.71E-09	0.015	0.010	8.00E-02
LDLC	rs2618566	G	0.34	0.025	0.002	1.20E-29	-0.016	0.008	7.50E-02
LDLC	rs438568	A	0.39	-0.013	0.002	4.03E-09	0.016	0.008	6.10E-02
LDLC	rs6050463	G	0.51	-0.013	0.002	1.82E-09	-0.024	0.008	9.70E-04
LDLC	rs6073958	T	0.80	-0.017	0.003	2.61E-11	-0.001	0.010	9.50E-01
LDLC	rs6090101	G	0.80	-0.018	0.003	3.06E-11	-0.002	0.010	7.10E-01
LDLC	rs6093446	G	0.71	-0.022	0.002	7.41E-22	-0.003	0.009	7.50E-01

LDLC	rs61433703	G	0.84	-0.016	0.003	2.67E-08	0.015	0.011	2.50E-01
LDLC	rs73075609	C	0.97	-0.037	0.007	1.63E-08	0.031	0.024	1.80E-01
LDLC	rs4818025	A	0.43	-0.014	0.002	8.67E-11	-0.003	0.008	7.90E-01
LDLC	rs12162782	T	0.66	-0.013	0.002	4.35E-09	0.020	0.008	1.00E-02
LDLC	rs960596	C	0.66	-0.013	0.002	1.33E-09	-0.022	0.008	7.70E-03
SBP	rs1043069	T	0.62	0.016	0.003	5.14E-15	0.002	0.008	8.50E-01
SBP	rs10923038	A	0.61	-0.015	0.003	2.57E-10	-0.002	0.008	7.00E-01
SBP	rs11210029	A	0.63	0.021	0.003	5.14E-09	0.010	0.008	1.70E-01
SBP	rs11579440	T	0.85	-0.016	0.002	9.45E-10	0.005	0.011	7.70E-01
SBP	rs12042924	T	0.53	-0.017	0.003	6.22E-11	-0.002	0.008	6.70E-01
SBP	rs17396055	A	0.33	0.015	0.003	6.08E-11	-0.001	0.008	9.50E-01
SBP	rs260508	T	0.61	0.018	0.003	5.02E-09	0.004	0.008	7.30E-01
SBP	rs2807337	T	0.37	-0.035	0.006	3.49E-12	-0.004	0.008	5.70E-01
SBP	rs33996239	T	0.06	0.030	0.005	1.70E-10	-0.031	0.016	4.60E-02
SBP	rs3737801	C	0.91	0.019	0.003	2.99E-09	-0.023	0.015	9.30E-02
SBP	rs4651224	T	0.45	0.030	0.004	7.49E-15	-0.004	0.008	6.00E-01
SBP	rs4926499	C	0.82	0.020	0.003	2.83E-15	-0.011	0.010	2.90E-01
SBP	rs7514579	A	0.77	0.020	0.003	1.82E-11	-0.010	0.009	2.20E-01
SBP	rs7555285	C	0.80	-0.024	0.004	1.11E-10	0.016	0.010	6.00E-02
SBP	rs76719272	T	0.13	0.059	0.008	1.93E-10	0.005	0.012	4.20E-01
SBP	rs79598313	T	0.03	-0.023	0.003	1.82E-13	0.033	0.025	1.70E-01
SBP	rs839755	A	0.62	0.014	0.003	1.31E-19	-0.004	0.008	6.00E-01
SBP	rs10189186	A	0.53	-0.023	0.003	1.28E-08	-0.005	0.008	5.00E-01
SBP	rs1044822	T	0.15	-0.020	0.003	6.26E-11	-0.014	0.011	9.80E-02
SBP	rs1047891	A	0.32	-0.015	0.003	9.43E-14	-0.004	0.008	7.40E-01
SBP	rs11694601	A	0.59	-0.018	0.003	2.81E-09	-0.009	0.008	2.50E-01
SBP	rs12694277	T	0.30	0.055	0.008	7.37E-11	-0.011	0.009	1.90E-01
SBP	rs139354822	T	0.97	0.015	0.003	1.03E-12	-0.014	0.024	6.70E-01
SBP	rs1837164	A	0.37	-0.025	0.005	3.28E-09	-0.014	0.008	1.10E-01
SBP	rs2059752	T	0.90	0.019	0.003	2.41E-08	0.011	0.014	5.20E-01
SBP	rs2300481	T	0.39	-0.019	0.003	8.17E-15	-0.002	0.008	6.00E-01
SBP	rs28377357	A	0.29	-0.019	0.003	3.09E-12	-0.031	0.009	5.80E-04
SBP	rs28558491	T	0.74	0.019	0.003	1.57E-11	0.003	0.009	7.60E-01
SBP	rs2920899	T	0.79	0.016	0.002	1.14E-09	0.002	0.010	8.40E-01
SBP	rs296797	T	0.41	-0.022	0.003	3.73E-11	-0.005	0.008	5.30E-01
SBP	rs35590893	A	0.27	-0.024	0.004	5.06E-15	-0.007	0.009	3.70E-01
SBP	rs55732192	T	0.09	0.017	0.003	1.53E-08	-0.017	0.013	1.40E-01
SBP	rs6545155	T	0.78	-0.016	0.003	9.91E-09	-0.002	0.009	9.20E-01
SBP	rs6712203	T	0.37	0.023	0.004	9.27E-10	-0.030	0.008	1.60E-04
SBP	rs6723509	T	0.86	0.017	0.003	2.24E-11	0.002	0.011	8.90E-01
SBP	rs6739913	A	0.28	0.020	0.003	6.38E-10	0.004	0.009	7.70E-01
SBP	rs67720684	A	0.24	0.022	0.003	2.44E-12	-0.004	0.009	7.00E-01
SBP	rs72816333	A	0.83	0.021	0.004	5.64E-12	-0.010	0.010	3.50E-01
SBP	rs72844590	T	0.15	0.022	0.003	4.88E-09	-0.002	0.011	7.80E-01
SBP	rs72847885	A	0.66	-0.026	0.004	4.61E-17	0.000	0.008	8.70E-01
SBP	rs79523138	A	0.88	-0.061	0.011	3.96E-11	-0.003	0.012	6.20E-01
SBP	rs111464338	T	0.98	0.021	0.003	1.89E-08	-0.007	0.034	7.40E-01
SBP	rs12638085	A	0.35	0.023	0.003	8.60E-16	0.006	0.008	3.60E-01
SBP	rs1375564	T	0.64	-0.022	0.004	5.57E-20	0.002	0.008	7.20E-01
SBP	rs1882289	A	0.88	-0.078	0.012	1.22E-08	0.006	0.012	5.40E-01
SBP	rs189267552	A	0.01	-0.019	0.002	1.16E-11	0.016	0.035	6.50E-01
SBP	rs262986	A	0.47	-0.016	0.003	7.22E-14	-0.018	0.008	1.00E-02
SBP	rs3729931	A	0.36	-0.020	0.003	5.59E-10	-0.011	0.008	1.10E-01
SBP	rs4499560	A	0.32	0.042	0.006	3.43E-14	-0.021	0.008	1.20E-02
SBP	rs62271373	A	0.06	0.021	0.002	2.38E-14	0.046	0.017	6.40E-03
SBP	rs6438857	T	0.57	0.025	0.004	5.77E-18	0.004	0.008	7.80E-01

SBP	rs6774721	C	0.88	0.026	0.004	2.98E-10	0.002	0.011	7.40E-01
SBP	rs6788984	A	0.86	-0.021	0.003	4.28E-13	-0.023	0.011	4.80E-02
SBP	rs78151625	T	0.83	0.016	0.002	1.68E-10	0.012	0.011	3.00E-01
SBP	rs863930	A	0.54	-0.014	0.002	2.95E-11	0.004	0.008	4.90E-01
SBP	rs893982	C	0.40	0.018	0.003	2.82E-08	-0.006	0.008	3.60E-01
SBP	rs9311344	A	0.18	0.014	0.002	1.13E-08	0.005	0.010	7.10E-01
SBP	rs9842387	T	0.46	-0.015	0.003	1.82E-08	-0.003	0.008	8.70E-01
SBP	rs9869437	A	0.35	-0.017	0.002	3.65E-09	0.002	0.008	7.40E-01
SBP	rs9875380	T	0.46	0.019	0.002	1.60E-11	0.008	0.008	3.60E-01
SBP	rs10008637	T	0.54	-0.022	0.003	1.35E-14	0.016	0.008	2.60E-02
SBP	rs12511987	T	0.82	0.025	0.003	2.64E-11	-0.012	0.010	4.40E-01
SBP	rs13149209	T	0.78	-0.018	0.003	1.81E-17	-0.004	0.009	7.70E-01
SBP	rs1347345	A	0.62	0.029	0.003	1.41E-12	0.002	0.008	7.80E-01
SBP	rs17035181	T	0.85	-0.023	0.003	2.37E-17	0.014	0.011	2.00E-01
SBP	rs231708	C	0.69	-0.026	0.003	5.61E-18	0.019	0.008	3.70E-02
SBP	rs2610990	A	0.26	-0.017	0.003	3.95E-20	-0.025	0.009	4.50E-03
SBP	rs6823767	T	0.72	-0.023	0.003	2.29E-10	-0.010	0.009	1.80E-01
SBP	rs72719160	A	0.68	0.024	0.003	5.00E-19	0.000	0.008	1.00E+00
SBP	rs7439567	T	0.42	-0.038	0.007	6.44E-21	-0.011	0.008	1.40E-01
SBP	rs78231605	T	0.04	0.027	0.003	4.79E-08	-0.006	0.020	7.90E-01
SBP	rs10069690	T	0.26	-0.025	0.004	4.06E-21	-0.014	0.009	1.10E-01
SBP	rs12153395	A	0.11	0.020	0.003	3.20E-10	-0.018	0.012	1.10E-01
SBP	rs13179413	T	0.28	0.023	0.003	5.99E-13	0.022	0.009	5.80E-03
SBP	rs1694068	A	0.61	0.022	0.003	1.18E-20	0.018	0.008	2.40E-02
SBP	rs246973	T	0.29	-0.015	0.002	6.92E-16	-0.006	0.009	3.90E-01
SBP	rs3121685	T	0.48	0.014	0.003	2.02E-09	0.000	0.008	9.90E-01
SBP	rs4957026	A	0.34	0.026	0.004	2.67E-08	-0.009	0.008	3.70E-01
SBP	rs62373688	A	0.13	0.019	0.002	5.32E-12	-0.014	0.011	2.40E-01
SBP	rs702395	T	0.44	-0.025	0.003	8.21E-15	-0.002	0.008	7.60E-01
SBP	rs709668	A	0.20	-0.017	0.003	1.22E-16	0.003	0.010	6.50E-01
SBP	rs74774746	C	0.26	0.020	0.002	2.20E-09	0.007	0.009	4.70E-01
SBP	rs10782230	A	0.48	0.014	0.003	5.07E-16	0.001	0.008	8.80E-01
SBP	rs12661604	A	0.40	0.015	0.003	4.20E-08	-0.007	0.008	3.10E-01
SBP	rs179972	T	0.43	-0.018	0.003	1.15E-08	0.012	0.008	1.20E-01
SBP	rs2395622	T	0.82	-0.019	0.003	3.26E-08	0.001	0.010	9.40E-01
SBP	rs2498586	T	0.17	0.019	0.003	9.66E-09	-0.003	0.010	7.30E-01
SBP	rs2745599	A	0.55	-0.018	0.003	4.65E-14	-0.005	0.008	6.10E-01
SBP	rs7763294	T	0.32	0.014	0.002	6.92E-12	-0.001	0.008	9.20E-01
SBP	rs7765526	A	0.47	0.023	0.003	6.66E-09	-0.001	0.008	9.50E-01
SBP	rs9368222	A	0.27	0.016	0.003	3.50E-17	0.001	0.009	8.50E-01
SBP	rs9401090	T	0.75	0.021	0.003	9.25E-09	-0.016	0.009	6.20E-02
SBP	rs9885632	T	0.73	0.014	0.003	4.92E-14	-0.002	0.009	7.50E-01
SBP	rs1011390	T	0.39	0.030	0.004	1.56E-08	0.005	0.008	5.00E-01
SBP	rs10233127	A	0.11	0.016	0.002	1.40E-13	-0.010	0.013	4.50E-01
SBP	rs10274928	A	0.49	0.017	0.003	8.14E-11	0.024	0.008	2.70E-03
SBP	rs11771693	A	0.67	0.015	0.003	2.73E-10	-0.005	0.008	4.80E-01
SBP	rs12703989	A	0.49	0.017	0.003	1.17E-09	0.014	0.008	6.60E-02
SBP	rs1870735	C	0.45	-0.022	0.002	1.78E-11	0.007	0.008	2.40E-01
SBP	rs34072724	A	0.49	-0.015	0.003	3.94E-19	-0.012	0.008	7.00E-02
SBP	rs3807925	A	0.65	0.015	0.003	4.02E-09	-0.003	0.008	6.90E-01
SBP	rs6466878	T	0.32	0.016	0.003	7.74E-09	-0.008	0.008	3.00E-01
SBP	rs6593297	A	0.30	-0.015	0.003	7.92E-09	-0.022	0.009	1.60E-02
SBP	rs6953231	T	0.27	-0.020	0.003	3.26E-08	-0.010	0.009	2.40E-01
SBP	rs6959688	A	0.60	-0.018	0.003	2.51E-15	0.005	0.008	5.10E-01
SBP	rs6963105	A	0.43	-0.020	0.003	1.34E-11	-0.021	0.008	4.50E-03
SBP	rs848445	T	0.28	0.017	0.003	6.83E-13	-0.004	0.009	7.20E-01

SBP	rs13253358	T	0.30	0.017	0.003	6.32E-11	0.003	0.008	7.00E-01
SBP	rs1405349	T	0.72	-0.039	0.006	3.01E-10	0.023	0.009	3.30E-03
SBP	rs142449193	T	0.05	0.027	0.003	3.53E-10	-0.023	0.019	3.70E-01
SBP	rs1906672	A	0.23	0.024	0.003	1.06E-20	0.002	0.009	6.50E-01
SBP	rs1986971	A	0.70	0.019	0.003	4.13E-18	0.004	0.009	5.20E-01
SBP	rs2354862	A	0.64	-0.014	0.003	7.74E-14	-0.001	0.008	8.50E-01
SBP	rs2978398	A	0.42	0.016	0.003	3.31E-08	-0.001	0.008	9.70E-01
SBP	rs2979470	T	0.49	0.017	0.002	9.67E-11	-0.006	0.008	5.00E-01
SBP	rs4129585	A	0.44	0.018	0.003	1.16E-12	-0.002	0.008	8.10E-01
SBP	rs4598218	T	0.61	0.030	0.003	1.50E-12	-0.003	0.008	4.60E-01
SBP	rs4873492	T	0.18	0.018	0.003	1.17E-19	-0.002	0.010	9.00E-01
SBP	rs4875958	A	0.71	0.015	0.003	7.93E-11	-0.011	0.009	2.20E-01
SBP	rs61040371	T	0.63	0.023	0.003	3.61E-09	-0.004	0.008	5.80E-01
SBP	rs62523863	A	0.22	-0.024	0.003	5.22E-15	0.019	0.009	4.50E-02
SBP	rs72688070	T	0.17	-0.015	0.003	1.44E-13	-0.014	0.010	1.80E-01
SBP	rs10820855	T	0.68	0.020	0.003	2.59E-08	0.000	0.008	9.80E-01
SBP	rs1332813	T	0.35	-0.017	0.003	3.28E-14	-0.003	0.008	6.60E-01
SBP	rs184457	A	0.30	-0.018	0.003	1.07E-09	-0.010	0.008	1.30E-01
SBP	rs1891730	T	0.62	-0.022	0.003	3.11E-12	-0.001	0.008	9.10E-01
SBP	rs28558845	C	0.16	-0.017	0.003	1.27E-10	-0.008	0.011	5.10E-01
SBP	rs60191654	A	0.81	-0.024	0.002	3.20E-08	-0.008	0.010	4.80E-01
SBP	rs7023828	T	0.42	-0.018	0.003	1.33E-22	0.000	0.008	8.00E-01
SBP	rs7045409	A	0.37	-0.017	0.003	8.74E-13	-0.007	0.008	4.00E-01
SBP	rs7874646	T	0.20	0.024	0.004	1.79E-08	-0.003	0.010	7.60E-01
SBP	rs11187142	T	0.11	-0.018	0.003	1.91E-09	-0.006	0.013	7.20E-01
SBP	rs11197813	A	0.70	-0.030	0.005	1.82E-11	0.012	0.008	2.00E-01
SBP	rs11252324	T	0.08	-0.026	0.003	6.02E-11	-0.009	0.015	5.80E-01
SBP	rs1133400	A	0.79	0.023	0.003	7.70E-18	0.003	0.009	7.00E-01
SBP	rs11592107	A	0.31	-0.036	0.005	2.44E-18	0.011	0.008	2.00E-01
SBP	rs12572586	T	0.94	-0.027	0.004	1.76E-12	-0.014	0.016	2.80E-01
SBP	rs34130368	T	0.12	0.019	0.002	5.55E-12	0.005	0.012	8.70E-01
SBP	rs3802517	A	0.46	0.017	0.003	6.27E-15	0.003	0.008	7.30E-01
SBP	rs3858217	C	0.26	-0.021	0.004	3.32E-09	-0.015	0.009	1.30E-01
SBP	rs56085433	A	0.14	0.020	0.004	6.19E-09	-0.001	0.011	8.90E-01
SBP	rs56352451	T	0.13	-0.018	0.003	2.63E-08	-0.005	0.011	6.00E-01
SBP	rs72799495	A	0.16	-0.028	0.004	2.99E-08	0.003	0.010	7.30E-01
SBP	rs72834453	T	0.88	0.047	0.006	7.56E-13	0.012	0.012	3.90E-01
SBP	rs77413490	T	0.04	0.019	0.003	4.16E-14	-0.009	0.020	5.20E-01
SBP	rs7912283	A	0.35	-0.018	0.003	6.29E-14	-0.005	0.008	5.20E-01
SBP	rs10743086	A	0.21	0.031	0.004	1.48E-09	0.018	0.010	3.80E-02
SBP	rs1076485	T	0.14	0.017	0.003	1.09E-17	0.003	0.012	8.90E-01
SBP	rs10766533	A	0.71	-0.019	0.003	9.28E-10	0.010	0.009	2.30E-01
SBP	rs11031051	A	0.69	0.027	0.005	6.33E-13	0.011	0.008	2.00E-01
SBP	rs190194639	T	0.08	-0.029	0.002	4.42E-09	0.000	0.015	9.70E-01
SBP	rs4754196	A	0.52	0.021	0.003	5.48E-32	-0.006	0.008	5.40E-01
SBP	rs4980515	T	0.50	0.020	0.003	9.51E-17	0.004	0.008	7.10E-01
SBP	rs67976715	C	0.23	-0.016	0.003	2.37E-11	0.010	0.009	2.20E-01
SBP	rs7128707	A	0.26	0.039	0.004	3.07E-09	-0.005	0.009	5.00E-01
SBP	rs75905900	A	0.87	0.020	0.003	6.77E-26	-0.002	0.011	8.80E-01
SBP	rs871004	A	0.35	-0.035	0.004	1.18E-14	-0.019	0.008	1.90E-02
SBP	rs10437954	A	0.90	0.025	0.003	2.69E-16	-0.009	0.013	5.50E-01
SBP	rs10858966	C	0.29	0.046	0.006	4.74E-20	0.017	0.009	7.80E-02
SBP	rs11112548	A	0.95	-0.019	0.003	1.40E-13	-0.031	0.019	9.50E-02
SBP	rs11571376	C	0.70	0.022	0.004	3.60E-12	0.005	0.009	4.20E-01
SBP	rs117206641	A	0.14	-0.023	0.003	1.52E-09	0.015	0.013	3.10E-01
SBP	rs2024385	A	0.42	0.018	0.003	1.03E-19	0.013	0.008	9.40E-02

SBP	rs4143175	T	0.24	-0.022	0.003	2.96E-10	-0.004	0.009	6.60E-01
SBP	rs5742643	A	0.25	-0.020	0.003	2.21E-15	-0.017	0.009	4.30E-02
SBP	rs78998485	C	0.75	-0.020	0.003	2.93E-13	-0.001	0.009	9.10E-01
SBP	rs7963801	T	0.41	0.016	0.003	2.41E-15	-0.018	0.008	1.60E-02
SBP	rs7976167	T	0.69	0.016	0.003	7.37E-10	-0.006	0.008	3.90E-01
SBP	rs1331012	T	0.27	-0.015	0.003	2.95E-09	-0.004	0.009	5.60E-01
SBP	rs17245822	A	0.63	0.014	0.003	3.66E-09	0.002	0.008	8.30E-01
SBP	rs2152258	T	0.35	0.023	0.003	2.47E-08	0.006	0.008	4.30E-01
SBP	rs606950	A	0.62	-0.026	0.004	4.09E-20	-0.004	0.008	5.60E-01
SBP	rs73187288	A	0.89	0.039	0.003	1.61E-10	0.000	0.012	9.90E-01
SBP	rs7331680	T	0.15	-0.048	0.006	1.34E-29	0.012	0.011	1.40E-01
SBP	rs78474310	A	0.96	0.015	0.002	8.51E-16	-0.009	0.018	5.90E-01
SBP	rs7988232	A	0.42	0.022	0.003	2.79E-09	0.014	0.008	8.60E-02
SBP	rs912434	T	0.76	-0.023	0.004	6.64E-14	-0.014	0.009	1.10E-01
SBP	rs9507885	T	0.11	-0.019	0.003	3.70E-09	0.009	0.013	4.00E-01
SBP	rs9526707	A	0.32	0.027	0.002	2.06E-13	0.004	0.008	5.70E-01
SBP	rs9532243	A	0.48	0.018	0.002	7.31E-29	-0.014	0.008	6.50E-02
SBP	rs11159091	A	0.46	0.020	0.003	3.13E-13	0.005	0.008	5.30E-01
SBP	rs11623535	A	0.74	0.016	0.003	2.89E-13	0.008	0.009	4.40E-01
SBP	rs17115145	T	0.40	-0.016	0.002	3.47E-10	0.016	0.008	5.20E-02
SBP	rs34983854	A	0.61	0.098	0.009	6.93E-11	0.020	0.008	1.90E-02
SBP	rs72683923	T	0.98	-0.034	0.004	1.33E-25	0.066	0.027	1.50E-02
SBP	rs8014182	T	0.14	0.020	0.002	4.96E-21	0.002	0.012	9.70E-01
SBP	rs11629850	A	0.53	0.021	0.002	2.93E-16	-0.004	0.008	4.50E-01
SBP	rs11632436	C	0.50	0.023	0.003	4.79E-17	-0.004	0.008	6.30E-01
SBP	rs11634028	A	0.21	0.014	0.002	8.96E-13	-0.013	0.009	2.20E-01
SBP	rs12595031	T	0.58	0.015	0.003	2.03E-08	-0.007	0.008	4.90E-01
SBP	rs12901664	T	0.35	0.026	0.003	3.26E-09	0.010	0.008	2.80E-01
SBP	rs3743157	A	0.17	0.014	0.003	1.18E-15	0.000	0.010	9.30E-01
SBP	rs4775376	T	0.38	-0.025	0.003	4.35E-08	-0.002	0.008	8.80E-01
SBP	rs4965529	T	0.17	0.017	0.003	1.55E-14	0.006	0.010	5.90E-01
SBP	rs2060664	T	0.75	-0.020	0.003	1.45E-09	-0.003	0.009	5.50E-01
SBP	rs2379829	C	0.73	-0.023	0.003	5.90E-13	0.007	0.009	4.00E-01
SBP	rs34941092	A	0.15	-0.016	0.003	1.44E-11	0.004	0.011	6.60E-01
SBP	rs35450617	T	0.70	0.016	0.003	2.21E-09	0.001	0.009	9.40E-01
SBP	rs6540125	T	0.35	-0.018	0.003	4.50E-10	0.003	0.008	6.60E-01
SBP	rs7187540	A	0.34	-0.023	0.003	1.15E-10	0.012	0.008	1.40E-01
SBP	rs1036902	T	0.84	0.022	0.004	1.76E-11	-0.010	0.011	3.10E-01
SBP	rs112260610	T	0.14	-0.017	0.003	3.85E-10	-0.006	0.011	6.00E-01
SBP	rs112280096	A	0.36	0.019	0.003	1.03E-10	0.006	0.008	5.70E-01
SBP	rs1551355	T	0.23	-0.021	0.003	1.97E-11	-0.006	0.009	5.20E-01
SBP	rs34430710	A	0.68	0.026	0.003	4.61E-15	-0.004	0.008	6.30E-01
SBP	rs4480845	T	0.37	0.023	0.002	8.46E-24	0.011	0.008	2.00E-01
SBP	rs4925159	A	0.43	-0.024	0.003	2.59E-20	0.005	0.008	5.50E-01
SBP	rs6504213	T	0.42	-0.014	0.002	7.24E-22	0.002	0.008	8.10E-01
SBP	rs7218708	A	0.49	0.019	0.002	8.07E-09	-0.008	0.008	2.30E-01
SBP	rs9302885	A	0.44	-0.015	0.003	2.04E-15	0.003	0.008	5.60E-01
SBP	rs9899540	A	0.61	-0.023	0.003	6.29E-09	0.004	0.008	7.00E-01
SBP	rs10048404	T	0.37	0.021	0.003	3.36E-17	0.009	0.008	3.60E-01
SBP	rs10460108	A	0.48	-0.015	0.003	5.84E-13	0.003	0.008	8.00E-01
SBP	rs1154214	T	0.40	-0.022	0.003	4.09E-09	-0.018	0.008	5.60E-02
SBP	rs11876341	A	0.69	0.020	0.003	1.71E-16	-0.008	0.008	3.00E-01
SBP	rs12454712	T	0.62	-0.031	0.003	4.56E-15	0.016	0.008	5.70E-02
SBP	rs34413141	A	0.18	-0.016	0.003	2.33E-21	0.002	0.010	9.80E-01
SBP	rs62082230	A	0.28	-0.024	0.003	5.32E-09	0.011	0.009	1.50E-01
SBP	rs10409243	T	0.58	0.014	0.003	3.96E-21	-0.019	0.008	1.40E-02

SBP	rs1236093	T	0.40	-0.066	0.010	4.81E-08	0.011	0.008	1.50E-01
SBP	rs138877676	T	0.02	-0.023	0.002	2.29E-10	-0.010	0.030	7.90E-01
SBP	rs2613765	A	0.47	-0.020	0.004	1.03E-21	-0.012	0.008	1.40E-01
SBP	rs3810299	T	0.87	0.015	0.003	3.46E-08	-0.019	0.012	1.10E-01
SBP	rs7256564	A	0.31	-0.028	0.004	4.88E-09	0.010	0.008	2.00E-01
SBP	rs73046792	A	0.15	-0.022	0.003	3.99E-15	-0.002	0.010	8.70E-01
SBP	rs1764975	T	0.18	-0.016	0.003	1.64E-11	0.015	0.010	9.80E-02
SBP	rs2801008	T	0.68	0.020	0.002	7.70E-10	-0.019	0.008	3.10E-02
SBP	rs6021247	A	0.53	0.023	0.003	4.50E-17	0.013	0.008	1.10E-01
SBP	rs1882961	T	0.31	0.020	0.003	1.54E-18	0.000	0.008	9.50E-01
SBP	rs28578714	T	0.61	-0.030	0.005	3.97E-14	0.003	0.008	6.50E-01
SBP	rs9608690	A	0.07	0.029	0.002	2.81E-09	-0.017	0.016	2.40E-01
Strenuous sports	rs10946808	G	0.70	0.030	0.008	9.90E-10	-0.019	0.016	8.73E-03
Strenuous sports	rs111901094	G	0.89	0.039	0.010	3.00E-09	-0.106	0.000	1.02E-02
Strenuous sports	rs159544	G	0.61	0.030	0.008	1.30E-09	-0.009	0.220	7.91E-03
Strenuous sports	rs166840	G	0.41	0.030	0.007	3.10E-11	-0.013	0.110	7.94E-03
Strenuous sports	rs62253088	T	0.64	0.049	0.008	1.00E-19	-0.009	0.250	8.28E-03
Strenuous sports	rs75930676	C	0.03	0.073	0.019	2.00E-09	-0.018	0.310	1.82E-02
SmoIndex	rs10918701	G	0.37	0.012	0.002	1.97E-09	0.009	0.008	3.10E-01
SmoIndex	rs10922907	A	0.45	0.015	0.002	6.38E-14	0.012	0.008	1.40E-01
SmoIndex	rs11210229	A	0.38	0.017	0.002	1.90E-17	-0.007	0.008	4.40E-01
SmoIndex	rs1193237	G	0.44	-0.011	0.002	3.80E-08	-0.005	0.008	5.50E-01
SmoIndex	rs1931263	G	0.51	-0.011	0.002	3.80E-08	-0.012	0.008	9.70E-02
SmoIndex	rs1933270	T	0.36	0.013	0.002	8.03E-11	0.016	0.008	4.40E-02
SmoIndex	rs4949465	T	0.87	-0.017	0.003	1.46E-08	0.003	0.012	8.90E-01
SmoIndex	rs549845	G	0.30	0.016	0.002	1.24E-15	-0.012	0.008	1.60E-01
SmoIndex	rs7519626	C	0.32	0.012	0.002	1.97E-09	0.013	0.008	1.80E-01
SmoIndex	rs7528604	G	0.57	0.014	0.002	2.56E-12	0.011	0.008	2.20E-01
SmoIndex	rs7553348	G	0.44	0.014	0.002	2.56E-12	-0.015	0.008	3.50E-02
SmoIndex	rs9435340	T	0.34	0.012	0.002	1.97E-09	0.011	0.008	2.20E-01
SmoIndex	rs12623702	A	0.61	-0.014	0.002	2.56E-12	-0.008	0.008	2.80E-01
SmoIndex	rs13009008	A	0.33	0.012	0.002	1.97E-09	-0.007	0.008	3.70E-01
SmoIndex	rs13016665	C	0.58	-0.012	0.002	1.97E-09	-0.014	0.008	6.70E-02
SmoIndex	rs2678670	A	0.49	0.013	0.002	8.03E-11	0.010	0.008	2.20E-01
SmoIndex	rs2867112	T	0.84	0.021	0.003	2.56E-12	-0.006	0.011	6.10E-01
SmoIndex	rs2890772	G	0.41	-0.020	0.002	1.52E-23	-0.003	0.008	7.20E-01
SmoIndex	rs359243	T	0.39	-0.013	0.002	8.03E-11	-0.024	0.008	3.50E-03
SmoIndex	rs3769949	T	0.53	-0.012	0.002	1.97E-09	-0.010	0.008	2.00E-01
SmoIndex	rs3811038	T	0.72	-0.014	0.002	2.56E-12	-0.019	0.009	3.90E-02
SmoIndex	rs4473348	A	0.25	-0.015	0.002	6.38E-14	0.010	0.009	2.10E-01
SmoIndex	rs4671357	T	0.52	-0.014	0.002	2.56E-12	-0.008	0.008	2.80E-01
SmoIndex	rs62135536	C	0.97	0.035	0.006	5.43E-09	0.029	0.022	1.10E-01
SmoIndex	rs62155874	A	0.87	-0.024	0.003	1.24E-15	-0.025	0.012	2.70E-02
SmoIndex	rs62175972	T	0.97	0.031	0.006	2.38E-07	-0.019	0.022	4.10E-01
SmoIndex	rs6741228	T	0.43	0.011	0.002	3.80E-08	-0.008	0.008	3.10E-01
SmoIndex	rs7569203	A	0.69	-0.016	0.002	1.24E-15	-0.015	0.008	3.70E-02
SmoIndex	rs326341	G	0.53	0.014	0.002	2.56E-12	-0.018	0.008	3.60E-02
SmoIndex	rs421983	T	0.52	0.013	0.002	8.03E-11	-0.009	0.008	2.30E-01
SmoIndex	rs6778080	T	0.27	0.016	0.002	1.24E-15	-0.008	0.009	3.30E-01
SmoIndex	rs6779302	G	0.63	-0.013	0.002	8.03E-11	0.009	0.008	1.80E-01
SmoIndex	rs73220544	A	0.84	-0.016	0.003	9.64E-08	-0.005	0.011	6.20E-01
SmoIndex	rs775758	A	0.43	0.012	0.002	1.97E-09	0.013	0.008	1.30E-01
SmoIndex	rs9842947	C	0.33	-0.013	0.002	8.03E-11	0.003	0.008	7.50E-01
SmoIndex	rs17576594	G	0.72	0.016	0.002	1.24E-15	-0.008	0.009	4.80E-01
SmoIndex	rs317021	T	0.81	-0.017	0.003	1.46E-08	-0.013	0.010	2.80E-01
SmoIndex	rs61796681	A	0.91	-0.019	0.004	2.03E-06	0.011	0.014	4.60E-01

SmoIndex	rs624833	T	0.70	0.013	0.002	8.03E-11	0.004	0.008	5.50E-01
SmoIndex	rs72678864	G	0.83	0.018	0.003	1.97E-09	0.016	0.010	1.10E-01
SmoIndex	rs10052591	T	0.57	0.012	0.002	1.97E-09	0.003	0.008	9.40E-01
SmoIndex	rs11948770	T	0.77	-0.015	0.002	6.38E-14	0.003	0.009	7.10E-01
SmoIndex	rs13153393	A	0.88	-0.020	0.003	2.62E-11	-0.018	0.012	1.40E-01
SmoIndex	rs2080870	A	0.26	0.012	0.002	1.97E-09	-0.001	0.009	7.60E-01
SmoIndex	rs245774	A	0.27	-0.013	0.002	8.03E-11	-0.016	0.009	8.00E-02
SmoIndex	rs329120	C	0.58	0.014	0.002	2.56E-12	0.017	0.008	3.20E-02
SmoIndex	rs4571506	C	0.54	0.011	0.002	3.80E-08	0.010	0.008	1.90E-01
SmoIndex	rs4957528	A	0.21	-0.015	0.002	6.38E-14	0.016	0.010	1.30E-01
SmoIndex	rs71627581	G	0.89	0.019	0.003	2.40E-10	0.006	0.012	6.20E-01
SmoIndex	rs986391	G	0.37	0.016	0.002	1.24E-15	0.012	0.008	2.30E-01
SmoIndex	rs12202536	A	0.51	-0.012	0.002	1.97E-09	-0.010	0.008	3.60E-01
SmoIndex	rs2254710	C	0.24	0.013	0.002	8.03E-11	-0.016	0.009	1.00E-01
SmoIndex	rs2894808	T	0.92	-0.022	0.004	3.80E-08	-0.018	0.015	2.10E-01
SmoIndex	rs6935954	A	0.42	0.014	0.002	2.56E-12	0.014	0.008	6.70E-02
SmoIndex	rs7766610	C	0.18	0.018	0.003	1.97E-09	-0.003	0.010	9.40E-01
SmoIndex	rs10226228	A	0.63	-0.016	0.002	1.24E-15	-0.020	0.008	3.00E-02
SmoIndex	rs10282292	C	0.36	0.013	0.002	8.03E-11	0.001	0.008	9.90E-01
SmoIndex	rs11768481	C	0.67	0.013	0.002	8.03E-11	0.006	0.008	5.70E-01
SmoIndex	rs1922018	C	0.36	0.014	0.002	2.56E-12	0.016	0.008	2.80E-02
SmoIndex	rs2401924	G	0.50	0.015	0.002	6.38E-14	-0.008	0.008	3.60E-01
SmoIndex	rs4731925	C	0.32	-0.012	0.002	1.97E-09	-0.001	0.008	8.50E-01
SmoIndex	rs6957896	C	0.50	-0.011	0.002	3.80E-08	-0.005	0.008	6.10E-01
SmoIndex	rs6962772	A	0.85	0.016	0.003	9.64E-08	0.043	0.011	8.40E-05
SmoIndex	rs7807019	A	0.54	-0.015	0.002	6.38E-14	0.000	0.008	9.50E-01
SmoIndex	rs11783093	C	0.84	0.023	0.003	1.77E-14	0.012	0.011	1.50E-01
SmoIndex	rs2062882	G	0.59	-0.012	0.002	1.97E-09	0.008	0.008	3.20E-01
SmoIndex	rs35169606	T	0.61	0.013	0.002	8.03E-11	-0.002	0.008	7.50E-01
SmoIndex	rs72674867	A	0.77	0.013	0.002	8.03E-11	0.014	0.009	1.30E-01
SmoIndex	rs113382419	C	0.89	-0.041	0.003	1.61E-42	-0.002	0.012	8.70E-01
SmoIndex	rs1221148	C	0.59	0.013	0.002	8.03E-11	0.009	0.008	2.10E-01
SmoIndex	rs1246265	T	0.31	-0.013	0.002	8.03E-11	0.005	0.008	4.80E-01
SmoIndex	rs13296519	G	0.61	-0.014	0.002	2.56E-12	0.000	0.008	7.50E-01
SmoIndex	rs4543592	T	0.52	-0.012	0.002	1.97E-09	0.001	0.008	8.30E-01
SmoIndex	rs7039819	G	0.43	0.013	0.002	8.03E-11	-0.004	0.008	7.70E-01
SmoIndex	rs10823968	A	0.63	0.012	0.002	1.97E-09	-0.006	0.008	4.40E-01
SmoIndex	rs11255908	T	0.74	-0.015	0.002	6.38E-14	-0.009	0.009	3.60E-01
SmoIndex	rs12244388	G	0.66	-0.019	0.002	2.10E-21	0.006	0.008	5.50E-01
SmoIndex	rs17553262	A	0.89	-0.018	0.003	1.97E-09	0.002	0.012	8.40E-01
SmoIndex	rs2675638	G	0.58	0.012	0.002	1.97E-09	0.022	0.008	5.50E-03
SmoIndex	rs3896224	A	0.59	0.014	0.002	2.56E-12	-0.001	0.008	9.30E-01
SmoIndex	rs7077678	C	0.62	0.012	0.002	1.97E-09	-0.003	0.008	7.60E-01
SmoIndex	rs112282219	G	0.96	-0.033	0.005	4.11E-11	0.026	0.020	2.20E-01
SmoIndex	rs17309874	G	0.74	-0.016	0.002	1.24E-15	-0.017	0.009	8.60E-02
SmoIndex	rs34866095	A	0.69	-0.012	0.002	1.97E-09	-0.002	0.008	6.40E-01
SmoIndex	rs4391802	A	0.71	0.015	0.002	6.38E-14	0.002	0.008	9.10E-01
SmoIndex	rs75742406	G	0.74	0.014	0.002	2.56E-12	-0.015	0.009	1.00E-01
SmoIndex	rs9919670	G	0.61	-0.022	0.002	3.82E-28	-0.002	0.008	9.60E-01
SmoIndex	rs10879871	T	0.34	-0.014	0.002	2.56E-12	-0.002	0.008	8.10E-01
SmoIndex	rs12831617	C	0.76	-0.013	0.002	8.03E-11	-0.014	0.009	1.60E-01
SmoIndex	rs7297175	T	0.43	-0.012	0.002	1.97E-09	-0.012	0.008	1.80E-01
SmoIndex	rs74086911	G	0.93	0.021	0.004	1.52E-07	0.005	0.015	6.30E-01
SmoIndex	rs6562474	C	0.65	0.012	0.002	1.97E-09	-0.014	0.008	7.60E-02
SmoIndex	rs7333559	G	0.21	0.015	0.002	6.38E-14	0.006	0.010	3.70E-01
SmoIndex	rs3742365	T	0.60	-0.016	0.002	1.24E-15	-0.013	0.008	8.30E-02

SmoIndex	rs7155595	A	0.67	-0.013	0.002	8.03E-11	0.001	0.008	9.80E-01
SmoIndex	rs860326	C	0.43	0.012	0.002	1.97E-09	0.019	0.008	2.10E-02
SmoIndex	rs28485305	C	0.63	0.012	0.002	1.97E-09	0.008	0.008	5.00E-01
SmoIndex	rs35175834	G	0.79	-0.024	0.002	3.55E-33	0.003	0.009	9.40E-01
SmoIndex	rs6598539	T	0.49	-0.012	0.002	1.97E-09	-0.002	0.008	9.90E-01
SmoIndex	rs8042134	T	0.54	-0.014	0.002	2.56E-12	0.005	0.008	6.30E-01
SmoIndex	rs8042849	T	0.34	0.028	0.002	1.56E-44	0.009	0.008	1.70E-01
SmoIndex	rs1050847	C	0.43	0.011	0.002	3.80E-08	-0.009	0.008	3.00E-01
SmoIndex	rs11861214	G	0.78	0.014	0.002	2.56E-12	0.018	0.009	2.80E-02
SmoIndex	rs12708665	A	0.29	-0.013	0.002	8.03E-11	-0.023	0.009	1.00E-02
SmoIndex	rs369230	G	0.31	-0.013	0.002	8.03E-11	-0.020	0.008	1.80E-02
SmoIndex	rs57611503	G	0.49	0.011	0.002	3.80E-08	0.010	0.008	1.80E-01
SmoIndex	rs60952428	T	0.91	0.019	0.003	2.40E-10	0.004	0.014	7.70E-01
SmoIndex	rs889398	C	0.59	0.013	0.002	8.03E-11	0.016	0.008	4.90E-02
SmoIndex	rs67596067	G	0.65	-0.013	0.002	8.03E-11	0.001	0.008	8.80E-01
SmoIndex	rs732083	G	0.33	0.012	0.002	1.97E-09	0.017	0.008	5.60E-02
SmoIndex	rs8614	C	0.82	-0.017	0.003	1.46E-08	-0.011	0.010	3.50E-01
SmoIndex	rs9904288	T	0.71	0.012	0.002	1.97E-09	0.004	0.008	6.20E-01
SmoIndex	rs12967855	A	0.33	0.012	0.002	1.97E-09	-0.003	0.008	7.60E-01
SmoIndex	rs62098013	G	0.64	-0.012	0.002	1.97E-09	0.005	0.008	5.80E-01
SmoIndex	rs71367545	G	0.79	-0.015	0.002	6.38E-14	0.010	0.010	3.90E-01
SmoIndex	rs35343344	C	0.73	0.013	0.002	8.03E-11	-0.008	0.009	3.70E-01
SmoIndex	rs76608582	C	0.95	0.031	0.005	5.65E-10	0.039	0.019	6.20E-02
SmoIndex	rs12481282	G	0.72	-0.013	0.002	8.03E-11	-0.002	0.009	8.90E-01
SmoIndex	rs348809	A	0.35	-0.012	0.002	1.97E-09	0.001	0.008	8.30E-01
SmoIndex	rs4814873	C	0.77	0.014	0.002	2.56E-12	0.001	0.009	9.60E-01
SmoIndex	rs6011779	C	0.19	0.028	0.003	1.03E-20	0.007	0.010	5.00E-01
SmoIndex	rs6119897	G	0.76	-0.018	0.002	2.26E-19	0.010	0.009	2.30E-01
SmoIndex	rs147412694	G	0.85	-0.017	0.003	1.46E-08	0.002	0.011	9.10E-01
SmoIndex	rs2838834	C	0.70	-0.013	0.002	8.03E-11	-0.006	0.008	4.10E-01
SmoIndex	rs136233	A	0.81	-0.014	0.003	3.06E-06	-0.008	0.010	3.40E-01
SmoIndex	rs202645	A	0.20	-0.015	0.002	6.38E-14	-0.006	0.010	4.40E-01
SmoInit	rs1008078	T	0.40	0.023	0.003	1.63E-18	0.009	0.008	2.90E-01
SmoInit	rs10789369	G	0.62	-0.023	0.003	3.38E-19	0.006	0.008	4.90E-01
SmoInit	rs10873871	G	0.21	0.017	0.003	2.86E-08	-0.012	0.009	2.60E-01
SmoInit	rs10914684	A	0.32	-0.016	0.003	6.43E-09	0.002	0.008	8.40E-01
SmoInit	rs11162019	T	0.36	-0.015	0.003	4.97E-09	0.001	0.008	9.70E-01
SmoInit	rs12022778	C	0.20	0.027	0.003	3.23E-17	0.028	0.009	2.50E-03
SmoInit	rs12027999	C	0.12	-0.024	0.004	5.20E-10	0.005	0.012	8.00E-01
SmoInit	rs12130857	A	0.32	-0.018	0.003	3.64E-11	-0.019	0.008	1.30E-02
SmoInit	rs12563365	A	0.56	0.017	0.003	1.06E-10	0.014	0.008	8.70E-02
SmoInit	rs12739243	C	0.22	-0.021	0.003	4.49E-12	-0.002	0.009	7.60E-01
SmoInit	rs12740789	A	0.18	-0.028	0.003	1.17E-17	-0.003	0.010	8.50E-01
SmoInit	rs12755632	G	0.32	-0.015	0.003	1.89E-08	-0.010	0.008	2.10E-01
SmoInit	rs147052174	T	0.02	0.062	0.010	2.29E-10	0.023	0.029	5.00E-01
SmoInit	rs1514176	A	0.58	-0.019	0.003	7.63E-14	0.014	0.008	6.70E-02
SmoInit	rs1889571	G	0.13	0.022	0.004	4.26E-09	-0.004	0.012	8.40E-01
SmoInit	rs1937443	G	0.56	0.020	0.003	1.78E-15	0.008	0.008	4.10E-01
SmoInit	rs2637869	A	0.30	0.018	0.003	6.38E-11	0.004	0.009	8.20E-01
SmoInit	rs2901785	A	0.45	-0.017	0.003	1.45E-11	0.004	0.008	6.50E-01
SmoInit	rs301807	G	0.57	0.018	0.003	2.56E-12	0.005	0.008	4.80E-01
SmoInit	rs35656245	A	0.28	0.016	0.003	2.21E-08	-0.025	0.009	2.20E-03
SmoInit	rs3820277	T	0.53	-0.019	0.003	1.55E-13	0.002	0.008	8.30E-01
SmoInit	rs45444697	G	0.21	0.020	0.003	2.67E-10	0.012	0.009	1.80E-01
SmoInit	rs4912332	T	0.49	0.014	0.003	3.01E-08	0.006	0.008	2.60E-01
SmoInit	rs876793	C	0.35	-0.018	0.003	5.76E-11	0.006	0.008	4.50E-01

SmoInit	rs925524	G	0.71	0.016	0.003	3.01E-08	0.009	0.008	2.80E-01
SmoInit	rs951740	A	0.63	0.030	0.003	3.07E-29	0.005	0.008	5.00E-01
SmoInit	rs1004787	A	0.55	0.028	0.003	1.39E-28	0.017	0.008	1.30E-02
SmoInit	rs1022376	C	0.52	-0.015	0.003	1.62E-08	-0.010	0.008	2.20E-01
SmoInit	rs10490159	T	0.39	0.017	0.003	3.83E-11	-0.007	0.008	3.70E-01
SmoInit	rs114976176	C	0.35	-0.016	0.003	6.11E-09	-0.012	0.008	2.00E-01
SmoInit	rs11692435	A	0.08	0.025	0.005	4.55E-08	0.015	0.015	3.60E-01
SmoInit	rs12474587	T	0.43	0.024	0.003	4.83E-21	0.007	0.008	4.70E-01
SmoInit	rs12714017	C	0.51	0.015	0.003	3.65E-09	0.003	0.008	6.30E-01
SmoInit	rs13007361	A	0.21	0.018	0.003	2.33E-08	0.013	0.010	2.10E-01
SmoInit	rs13392222	C	0.14	-0.023	0.004	1.97E-10	0.008	0.011	4.80E-01
SmoInit	rs1445649	C	0.54	0.021	0.003	8.29E-16	-0.012	0.008	9.40E-02
SmoInit	rs1518393	C	0.62	0.017	0.003	1.31E-10	0.015	0.008	8.00E-02
SmoInit	rs16826827	C	0.12	-0.022	0.004	9.22E-09	0.003	0.012	8.00E-01
SmoInit	rs17229285	T	0.51	-0.015	0.003	1.24E-09	0.013	0.008	1.40E-01
SmoInit	rs17616642	G	0.25	-0.017	0.003	2.10E-08	0.011	0.009	2.40E-01
SmoInit	rs1863161	A	0.56	0.015	0.003	2.30E-09	0.011	0.008	1.40E-01
SmoInit	rs1901477	G	0.51	0.030	0.003	2.00E-31	-0.001	0.008	9.20E-01
SmoInit	rs2539706	A	0.53	0.016	0.003	1.97E-10	0.003	0.008	5.10E-01
SmoInit	rs2710634	C	0.52	-0.018	0.003	3.30E-12	-0.002	0.008	7.40E-01
SmoInit	rs3115418	C	0.45	-0.014	0.003	2.72E-08	-0.009	0.008	2.20E-01
SmoInit	rs34399632	G	0.23	0.019	0.003	1.45E-10	-0.018	0.009	3.60E-02
SmoInit	rs357304	C	0.73	0.017	0.003	5.51E-09	0.001	0.009	8.50E-01
SmoInit	rs359247	T	0.64	0.022	0.003	9.85E-17	0.029	0.008	4.60E-04
SmoInit	rs3811038	C	0.28	0.019	0.003	1.61E-11	0.019	0.009	3.90E-02
SmoInit	rs4674916	A	0.33	-0.018	0.003	3.12E-11	-0.003	0.008	7.90E-01
SmoInit	rs4674993	G	0.20	-0.024	0.003	4.82E-14	-0.007	0.010	4.80E-01
SmoInit	rs56208390	G	0.12	0.022	0.004	2.72E-08	-0.008	0.012	6.40E-01
SmoInit	rs61533748	C	0.38	0.017	0.003	2.82E-11	-0.003	0.008	7.00E-01
SmoInit	rs62106258	C	0.05	-0.045	0.006	3.38E-14	-0.043	0.018	2.00E-02
SmoInit	rs62137126	G	0.12	-0.024	0.004	1.31E-09	-0.017	0.012	1.40E-01
SmoInit	rs62180324	A	0.21	-0.020	0.003	3.83E-10	0.016	0.009	1.10E-01
SmoInit	rs62193862	A	0.10	0.024	0.004	1.99E-08	-0.003	0.013	8.60E-01
SmoInit	rs6731872	G	0.83	0.032	0.003	5.35E-21	-0.005	0.010	6.50E-01
SmoInit	rs6750107	A	0.39	0.015	0.003	2.58E-08	0.006	0.008	4.90E-01
SmoInit	rs6750529	T	0.74	0.020	0.003	9.16E-12	-0.010	0.009	2.00E-01
SmoInit	rs6756212	T	0.54	-0.034	0.003	3.62E-40	-0.002	0.008	7.00E-01
SmoInit	rs72790288	A	0.03	-0.046	0.008	3.30E-09	-0.009	0.023	7.60E-01
SmoInit	rs7598402	G	0.49	-0.015	0.003	7.50E-09	-0.010	0.008	2.50E-01
SmoInit	rs7600835	A	0.34	-0.015	0.003	1.80E-08	-0.011	0.008	2.20E-01
SmoInit	rs10446419	G	0.21	-0.020	0.003	4.94E-10	-0.006	0.010	3.80E-01
SmoInit	rs10935779	T	0.42	-0.014	0.003	3.01E-08	-0.006	0.008	4.10E-01
SmoInit	rs11128203	A	0.53	0.020	0.003	1.31E-15	0.017	0.008	2.30E-02
SmoInit	rs11713899	C	0.17	0.019	0.003	3.17E-08	-0.003	0.010	8.20E-01
SmoInit	rs1187820	T	0.44	-0.014	0.003	2.72E-08	-0.005	0.008	5.50E-01
SmoInit	rs12633090	C	0.18	-0.023	0.003	3.14E-12	0.001	0.010	7.20E-01
SmoInit	rs13066050	T	0.21	0.019	0.003	1.97E-09	0.019	0.010	7.30E-02
SmoInit	rs1449012	T	0.46	-0.015	0.003	1.78E-09	-0.012	0.008	2.10E-01
SmoInit	rs1549979	T	0.62	-0.025	0.003	8.87E-21	-0.001	0.008	1.00E+00
SmoInit	rs16828799	T	0.16	0.020	0.004	1.80E-08	-0.017	0.011	1.30E-01
SmoInit	rs1714521	C	0.41	-0.016	0.003	3.12E-10	-0.004	0.008	6.60E-01
SmoInit	rs221988	C	0.38	-0.015	0.003	1.39E-08	0.006	0.008	4.70E-01
SmoInit	rs2276825	C	0.25	0.019	0.003	1.87E-10	0.002	0.009	6.20E-01
SmoInit	rs2279829	T	0.22	-0.017	0.003	1.99E-08	-0.008	0.009	3.20E-01
SmoInit	rs2306866	T	0.61	-0.017	0.003	1.87E-10	-0.001	0.008	7.70E-01
SmoInit	rs2319545	A	0.15	0.023	0.004	8.24E-11	0.010	0.011	5.30E-01

SmoInit	rs2526390	T	0.33	0.020	0.003	3.56E-14	0.001	0.008	8.00E-01
SmoInit	rs2734390	G	0.37	0.015	0.003	2.10E-08	-0.001	0.008	6.30E-01
SmoInit	rs3172494	T	0.12	-0.029	0.004	3.34E-13	0.001	0.013	9.90E-01
SmoInit	rs4543050	T	0.82	0.022	0.003	1.45E-11	0.010	0.010	4.20E-01
SmoInit	rs62246017	A	0.32	-0.016	0.003	2.98E-09	-0.009	0.008	2.70E-01
SmoInit	rs6437769	T	0.58	0.014	0.003	3.70E-08	0.003	0.008	6.40E-01
SmoInit	rs6438436	T	0.82	0.025	0.003	5.34E-14	0.003	0.010	9.30E-01
SmoInit	rs6782116	T	0.42	-0.015	0.003	1.46E-08	-0.003	0.008	8.30E-01
SmoInit	rs73831818	G	0.06	0.032	0.005	5.51E-09	0.024	0.016	1.60E-01
SmoInit	rs748832	G	0.37	0.017	0.003	6.72E-11	-0.006	0.008	3.20E-01
SmoInit	rs7631379	C	0.21	0.021	0.003	4.03E-11	-0.001	0.010	9.60E-01
SmoInit	rs7640107	T	0.43	-0.014	0.003	3.52E-08	-0.020	0.008	7.50E-03
SmoInit	rs9288999	A	0.74	0.017	0.003	1.53E-09	-0.006	0.009	4.60E-01
SmoInit	rs9826984	A	0.54	-0.014	0.003	3.90E-08	-0.006	0.008	3.80E-01
SmoInit	rs9841807	T	0.27	0.016	0.003	1.32E-08	-0.005	0.009	5.80E-01
SmoInit	rs9850597	A	0.82	-0.019	0.003	1.62E-08	-0.007	0.010	4.60E-01
SmoInit	rs1116690	G	0.74	0.016	0.003	2.21E-08	-0.008	0.009	2.50E-01
SmoInit	rs112725451	T	0.17	0.026	0.003	1.66E-14	-0.010	0.010	5.80E-01
SmoInit	rs1160685	G	0.45	0.015	0.003	2.30E-09	-0.013	0.008	1.00E-01
SmoInit	rs13109980	A	0.33	-0.022	0.003	3.33E-16	-0.016	0.008	4.70E-02
SmoInit	rs13110073	C	0.40	-0.025	0.003	3.23E-21	0.001	0.008	9.50E-01
SmoInit	rs1435479	T	0.29	0.016	0.003	5.80E-09	0.004	0.008	6.40E-01
SmoInit	rs3934797	A	0.18	-0.021	0.003	1.12E-10	-0.012	0.010	1.80E-01
SmoInit	rs4140932	A	0.43	-0.014	0.003	4.79E-08	-0.015	0.008	5.50E-02
SmoInit	rs55944129	C	0.27	-0.018	0.003	1.07E-09	-0.005	0.009	7.10E-01
SmoInit	rs58400863	A	0.35	-0.020	0.003	4.82E-14	0.005	0.008	4.90E-01
SmoInit	rs59537158	T	0.21	0.022	0.003	4.53E-13	0.011	0.009	2.60E-01
SmoInit	rs62340589	C	0.20	0.017	0.003	4.32E-08	0.011	0.010	2.20E-01
SmoInit	rs71602617	T	0.22	-0.018	0.003	2.10E-08	0.001	0.010	8.10E-01
SmoInit	rs7657022	G	0.49	0.018	0.003	7.16E-13	0.007	0.008	2.90E-01
SmoInit	rs7696257	A	0.37	0.015	0.003	6.77E-09	-0.008	0.008	3.00E-01
SmoInit	rs10042827	C	0.68	0.017	0.003	9.62E-10	0.014	0.008	7.20E-02
SmoInit	rs10060196	A	0.58	0.018	0.003	1.32E-12	-0.017	0.008	5.10E-02
SmoInit	rs10805858	T	0.34	0.018	0.003	1.87E-11	0.002	0.008	9.90E-01
SmoInit	rs1173461	T	0.33	0.017	0.003	9.62E-10	-0.011	0.008	1.50E-01
SmoInit	rs11956866	G	0.57	-0.015	0.003	7.90E-09	-0.003	0.008	7.30E-01
SmoInit	rs12517438	G	0.54	0.015	0.003	1.87E-09	-0.002	0.008	7.80E-01
SmoInit	rs1385108	T	0.24	0.019	0.003	3.83E-10	0.019	0.009	4.30E-02
SmoInit	rs17165769	G	0.39	0.016	0.003	9.62E-10	0.003	0.008	6.40E-01
SmoInit	rs2028269	A	0.40	0.016	0.003	5.20E-10	-0.015	0.008	7.60E-02
SmoInit	rs2173019	A	0.18	0.028	0.003	2.92E-17	0.020	0.010	5.70E-02
SmoInit	rs329124	G	0.43	-0.016	0.003	1.97E-10	-0.017	0.008	2.90E-02
SmoInit	rs35375873	C	0.11	-0.027	0.004	3.28E-11	-0.001	0.012	8.90E-01
SmoInit	rs359431	T	0.56	-0.014	0.003	3.17E-08	0.006	0.008	5.90E-01
SmoInit	rs3843905	T	0.40	-0.015	0.003	5.51E-09	-0.005	0.008	5.90E-01
SmoInit	rs3909281	G	0.54	0.021	0.003	1.63E-16	0.015	0.008	6.00E-02
SmoInit	rs4044321	G	0.64	-0.023	0.003	1.76E-17	-0.013	0.008	1.60E-01
SmoInit	rs42417	T	0.69	0.017	0.003	8.25E-10	-0.006	0.008	4.80E-01
SmoInit	rs6452785	T	0.47	-0.027	0.003	5.92E-26	-0.011	0.008	1.40E-01
SmoInit	rs6874731	G	0.48	0.015	0.003	1.87E-09	-0.009	0.008	2.90E-01
SmoInit	rs6890961	T	0.62	-0.019	0.003	2.11E-13	0.011	0.008	1.50E-01
SmoInit	rs71592686	C	0.27	0.021	0.003	3.89E-13	0.001	0.009	9.30E-01
SmoInit	rs72780746	C	0.17	-0.026	0.003	2.03E-14	-0.016	0.010	1.50E-01
SmoInit	rs72789626	A	0.14	-0.026	0.004	5.23E-12	0.003	0.011	8.40E-01
SmoInit	rs986714	T	0.45	-0.016	0.003	4.03E-10	-0.005	0.008	6.90E-01
SmoInit	rs10698713	A	0.05	-0.034	0.006	2.42E-09	0.000	0.017	9.40E-01

SmoInit	rs10945141	A	0.26	0.018	0.003	3.63E-10	-0.008	0.009	3.00E-01
SmoInit	rs1150668	G	0.42	-0.019	0.003	8.34E-13	-0.002	0.008	9.20E-01
SmoInit	rs118202	T	0.81	-0.037	0.003	1.86E-29	0.002	0.010	9.90E-01
SmoInit	rs12195240	A	0.29	0.025	0.003	1.08E-18	-0.008	0.009	3.70E-01
SmoInit	rs160631	G	0.73	-0.017	0.003	1.87E-09	0.005	0.009	6.70E-01
SmoInit	rs1632941	C	0.46	-0.016	0.003	6.72E-10	-0.014	0.008	5.90E-02
SmoInit	rs1737329	G	0.74	0.017	0.003	4.97E-09	-0.004	0.009	6.40E-01
SmoInit	rs17554906	C	0.44	0.014	0.003	3.17E-08	0.002	0.008	7.50E-01
SmoInit	rs3218116	T	0.26	-0.020	0.003	1.07E-11	0.003	0.009	9.50E-01
SmoInit	rs3800227	G	0.74	0.017	0.003	3.65E-09	-0.013	0.009	1.70E-01
SmoInit	rs619087	G	0.42	0.014	0.003	3.17E-08	-0.001	0.008	8.10E-01
SmoInit	rs6568832	A	0.75	0.019	0.003	1.77E-10	-0.015	0.009	1.10E-01
SmoInit	rs6936160	T	0.70	0.020	0.003	4.30E-13	0.007	0.008	4.80E-01
SmoInit	rs73008357	C	0.12	-0.022	0.004	2.45E-08	0.000	0.012	9.10E-01
SmoInit	rs7743165	G	0.50	0.019	0.003	4.14E-14	0.011	0.008	2.50E-01
SmoInit	rs9331343	C	0.57	-0.014	0.003	3.90E-08	0.011	0.008	1.40E-01
SmoInit	rs10233018	G	0.52	0.025	0.003	4.73E-22	0.000	0.008	9.80E-01
SmoInit	rs10279261	A	0.62	-0.019	0.003	6.15E-13	0.004	0.008	8.80E-01
SmoInit	rs1030015	T	0.52	0.014	0.003	2.10E-08	-0.004	0.008	6.10E-01
SmoInit	rs10953957	A	0.39	0.014	0.003	3.70E-08	-0.004	0.008	6.20E-01
SmoInit	rs11766326	C	0.51	-0.018	0.003	1.78E-11	-0.001	0.008	8.50E-01
SmoInit	rs11768481	A	0.34	-0.019	0.003	5.23E-12	-0.006	0.008	5.70E-01
SmoInit	rs13237637	C	0.49	-0.024	0.003	1.55E-20	-0.004	0.008	6.30E-01
SmoInit	rs13437771	G	0.16	-0.027	0.004	1.35E-14	-0.042	0.011	9.10E-05
SmoInit	rs1799068	T	0.38	0.017	0.003	2.54E-10	-0.011	0.008	9.70E-02
SmoInit	rs4727189	C	0.34	0.015	0.003	3.01E-08	0.001	0.008	9.90E-01
SmoInit	rs6948707	G	0.42	0.024	0.003	4.15E-21	0.004	0.008	5.80E-01
SmoInit	rs6968380	A	0.68	-0.023	0.003	1.06E-17	0.002	0.008	8.70E-01
SmoInit	rs76841737	G	0.10	-0.023	0.004	3.34E-08	0.000	0.013	9.50E-01
SmoInit	rs77283305	A	0.31	-0.015	0.003	3.90E-08	0.000	0.008	9.00E-01
SmoInit	rs7802996	T	0.17	-0.021	0.003	1.07E-09	-0.008	0.010	6.00E-01
SmoInit	rs7809303	A	0.33	-0.021	0.003	3.43E-15	0.022	0.008	9.50E-03
SmoInit	rs11783093	T	0.16	-0.047	0.003	1.77E-41	-0.012	0.011	1.50E-01
SmoInit	rs13261666	T	0.52	-0.020	0.003	4.43E-15	-0.014	0.008	5.50E-02
SmoInit	rs2063976	T	0.66	-0.020	0.003	7.63E-14	0.000	0.008	8.50E-01
SmoInit	rs290601	T	0.27	0.016	0.003	1.13E-08	-0.004	0.009	6.50E-01
SmoInit	rs3850736	G	0.47	0.019	0.003	6.55E-14	-0.013	0.008	1.40E-01
SmoInit	rs4326350	G	0.49	-0.018	0.003	5.23E-12	-0.004	0.008	5.20E-01
SmoInit	rs6986430	C	0.22	-0.024	0.003	1.96E-15	-0.009	0.009	3.10E-01
SmoInit	rs7836565	T	0.72	-0.016	0.003	4.32E-08	0.005	0.009	5.30E-01
SmoInit	rs9987376	G	0.57	-0.020	0.003	1.96E-15	-0.018	0.008	1.50E-02
SmoInit	rs10858334	G	0.14	0.023	0.004	1.18E-09	-0.021	0.011	8.70E-02
SmoInit	rs10966092	C	0.27	-0.020	0.003	1.13E-12	-0.008	0.009	3.70E-01
SmoInit	rs10969352	A	0.50	0.014	0.003	1.80E-08	-0.005	0.008	4.40E-01
SmoInit	rs11791671	T	0.07	0.028	0.005	4.32E-08	0.001	0.015	9.00E-01
SmoInit	rs1759433	A	0.48	0.015	0.003	1.69E-09	-0.008	0.008	2.00E-01
SmoInit	rs1927901	C	0.55	-0.014	0.003	3.17E-08	0.012	0.008	1.00E-01
SmoInit	rs1930371	T	0.24	-0.017	0.003	7.13E-09	0.004	0.009	5.00E-01
SmoInit	rs1931431	C	0.48	0.018	0.003	8.78E-13	0.006	0.008	3.30E-01
SmoInit	rs2378662	A	0.54	0.015	0.003	2.68E-09	0.005	0.008	4.40E-01
SmoInit	rs34553878	G	0.11	0.025	0.004	1.18E-09	0.005	0.013	5.80E-01
SmoInit	rs3847244	T	0.47	0.019	0.003	2.59E-13	-0.004	0.008	5.70E-01
SmoInit	rs4837631	T	0.45	-0.015	0.003	2.08E-09	-0.014	0.008	6.70E-02
SmoInit	rs4877285	A	0.67	-0.018	0.003	2.07E-11	0.008	0.008	4.10E-01
SmoInit	rs7024924	C	0.17	0.019	0.003	1.89E-08	0.016	0.010	1.40E-01
SmoInit	rs7026534	G	0.70	-0.017	0.003	2.68E-09	-0.015	0.008	3.80E-02

SmoInit	rs7867822	G	0.67	-0.015	0.003	2.72E-08	-0.004	0.008	4.90E-01
SmoInit	rs10885480	C	0.28	-0.019	0.003	3.83E-11	0.001	0.009	9.40E-01
SmoInit	rs10905461	C	0.75	-0.016	0.003	2.33E-08	-0.008	0.009	3.90E-01
SmoInit	rs11192347	A	0.10	-0.026	0.004	6.07E-10	-0.013	0.012	2.90E-01
SmoInit	rs11258417	T	0.39	-0.015	0.003	2.72E-08	-0.004	0.008	5.50E-01
SmoInit	rs11594623	C	0.23	0.027	0.003	7.42E-20	-0.003	0.009	5.20E-01
SmoInit	rs12244388	A	0.35	0.026	0.003	4.27E-22	-0.006	0.008	5.50E-01
SmoInit	rs1291821	G	0.53	0.014	0.003	1.39E-08	0.002	0.008	8.50E-01
SmoInit	rs1733760	C	0.51	0.015	0.003	6.77E-09	-0.008	0.008	4.30E-01
SmoInit	rs2796793	A	0.45	0.014	0.003	1.54E-08	0.002	0.008	8.80E-01
SmoInit	rs28408682	G	0.60	0.017	0.003	1.45E-10	0.001	0.008	8.90E-01
SmoInit	rs4752018	A	0.23	0.019	0.003	4.46E-10	0.017	0.009	1.20E-01
SmoInit	rs7072776	G	0.71	-0.022	0.003	5.71E-15	-0.013	0.009	1.90E-01
SmoInit	rs7920501	A	0.47	-0.016	0.003	1.24E-09	0.002	0.008	8.30E-01
SmoInit	rs7921378	C	0.48	-0.023	0.003	6.06E-20	0.003	0.008	5.80E-01
SmoInit	rs9423279	G	0.65	-0.019	0.003	2.98E-12	-0.007	0.008	3.50E-01
SmoInit	rs1106363	T	0.34	0.017	0.003	9.13E-11	0.009	0.008	2.20E-01
SmoInit	rs1381775	C	0.71	-0.016	0.003	2.86E-08	-0.004	0.009	5.00E-01
SmoInit	rs1713676	G	0.52	-0.017	0.003	5.47E-11	0.013	0.008	1.00E-01
SmoInit	rs2010921	A	0.31	0.017	0.003	2.41E-10	-0.003	0.008	6.90E-01
SmoInit	rs2155646	C	0.40	0.038	0.003	8.31E-48	0.002	0.008	9.50E-01
SmoInit	rs238896	A	0.49	-0.017	0.003	3.64E-11	0.015	0.008	2.90E-02
SmoInit	rs2939756	A	0.48	-0.016	0.003	7.45E-10	-0.002	0.008	8.00E-01
SmoInit	rs2959084	A	0.70	0.017	0.003	9.62E-10	0.003	0.009	5.20E-01
SmoInit	rs3740977	C	0.17	0.019	0.003	1.19E-08	-0.023	0.010	2.80E-02
SmoInit	rs540860	G	0.54	0.018	0.003	5.79E-12	-0.003	0.008	7.10E-01
SmoInit	rs586699	A	0.54	-0.015	0.003	7.13E-09	0.007	0.008	4.80E-01
SmoInit	rs61884449	T	0.15	0.020	0.004	2.33E-08	-0.005	0.011	7.20E-01
SmoInit	rs61886926	T	0.38	-0.018	0.003	7.47E-12	0.002	0.008	7.10E-01
SmoInit	rs62618693	T	0.04	-0.035	0.006	2.10E-08	-0.034	0.019	1.00E-01
SmoInit	rs6265	T	0.19	-0.029	0.003	2.76E-19	-0.034	0.010	1.40E-03
SmoInit	rs644740	T	0.46	-0.014	0.003	3.70E-08	-0.010	0.008	2.50E-01
SmoInit	rs76460663	G	0.04	-0.042	0.006	4.24E-11	0.012	0.020	4.20E-01
SmoInit	rs7929518	G	0.77	0.019	0.003	2.54E-10	0.008	0.009	3.40E-01
SmoInit	rs7943721	A	0.83	-0.021	0.003	3.63E-10	-0.002	0.010	9.70E-01
SmoInit	rs11057005	G	0.44	-0.016	0.003	9.14E-10	-0.005	0.008	4.70E-01
SmoInit	rs1109480	A	0.38	-0.017	0.003	1.87E-10	-0.003	0.008	8.30E-01
SmoInit	rs11611651	A	0.09	0.027	0.005	2.08E-09	-0.012	0.014	4.40E-01
SmoInit	rs13906	T	0.11	-0.025	0.004	1.97E-09	-0.006	0.013	5.90E-01
SmoInit	rs4759229	G	0.66	0.016	0.003	6.43E-09	0.015	0.008	8.70E-02
SmoInit	rs7134009	C	0.29	-0.016	0.003	4.32E-08	-0.011	0.009	2.20E-01
SmoInit	rs77215829	C	0.13	-0.024	0.004	2.07E-10	-0.018	0.011	1.10E-01
SmoInit	rs7969559	G	0.71	-0.017	0.003	1.53E-09	0.001	0.009	5.40E-01
SmoInit	rs1108130	A	0.21	0.024	0.003	1.58E-14	0.003	0.010	8.80E-01
SmoInit	rs12855717	T	0.54	0.016	0.003	1.24E-09	0.004	0.008	6.20E-01
SmoInit	rs17197663	A	0.13	-0.022	0.004	2.10E-08	0.004	0.011	6.50E-01
SmoInit	rs1772572	A	0.32	-0.017	0.003	5.76E-10	0.013	0.008	1.40E-01
SmoInit	rs4264267	T	0.53	0.015	0.003	6.77E-09	0.000	0.008	1.00E+00
SmoInit	rs4886207	C	0.64	-0.016	0.003	8.68E-10	0.012	0.008	2.20E-01
SmoInit	rs55786907	G	0.16	0.019	0.003	1.80E-08	0.006	0.011	5.20E-01
SmoInit	rs61959481	A	0.21	-0.020	0.003	7.83E-11	0.010	0.010	3.50E-01
SmoInit	rs7333559	A	0.78	-0.023	0.003	5.91E-14	-0.006	0.010	3.70E-01
SmoInit	rs75674569	A	0.10	-0.025	0.004	2.55E-09	0.004	0.013	6.40E-01
SmoInit	rs9538162	C	0.42	0.017	0.003	1.78E-11	-0.013	0.008	9.80E-02
SmoInit	rs9540731	T	0.51	-0.018	0.003	3.48E-12	-0.008	0.008	3.50E-01
SmoInit	rs9545155	C	0.48	-0.016	0.003	2.96E-10	-0.004	0.008	5.50E-01

SmoInit	rs12878369	A	0.41	0.017	0.003	1.61E-11	0.011	0.008	2.20E-01
SmoInit	rs1381287	T	0.47	0.018	0.003	1.79E-12	-0.004	0.008	6.20E-01
SmoInit	rs1811739	A	0.25	0.018	0.003	6.07E-10	0.009	0.009	2.90E-01
SmoInit	rs2925128	T	0.39	0.017	0.003	3.63E-10	0.001	0.008	9.00E-01
SmoInit	rs34940743	G	0.35	0.016	0.003	2.83E-09	0.023	0.008	3.10E-03
SmoInit	rs55913542	T	0.18	0.019	0.003	3.17E-08	0.009	0.010	3.20E-01
SmoInit	rs8005334	G	0.36	0.017	0.003	3.45E-10	0.008	0.008	3.20E-01
SmoInit	rs9323328	G	0.54	-0.014	0.003	2.58E-08	0.000	0.008	1.00E+00
SmoInit	rs1435672	C	0.56	0.014	0.003	3.90E-08	0.006	0.008	4.40E-01
SmoInit	rs2289791	T	0.25	-0.018	0.003	1.97E-09	-0.006	0.009	6.10E-01
SmoInit	rs281296	A	0.36	0.025	0.003	1.63E-20	0.000	0.008	7.20E-01
SmoInit	rs4310804	G	0.25	-0.018	0.003	7.45E-10	-0.003	0.009	7.30E-01
SmoInit	rs56902655	G	0.14	-0.022	0.004	4.05E-09	0.004	0.011	6.60E-01
SmoInit	rs60833441	G	0.46	-0.014	0.003	2.33E-08	-0.003	0.008	9.50E-01
SmoInit	rs62007780	T	0.42	-0.016	0.003	7.45E-10	0.001	0.008	8.80E-01
SmoInit	rs8027457	C	0.51	0.015	0.003	1.87E-09	0.002	0.008	9.90E-01
SmoInit	rs1050847	T	0.56	-0.015	0.003	7.50E-09	0.009	0.008	3.00E-01
SmoInit	rs11076962	C	0.28	0.018	0.003	1.18E-10	0.034	0.009	3.10E-05
SmoInit	rs1139897	A	0.23	-0.024	0.003	1.78E-15	-0.018	0.009	3.20E-02
SmoInit	rs11642231	A	0.37	-0.016	0.003	3.47E-09	-0.009	0.008	3.30E-01
SmoInit	rs117657830	G	0.04	-0.038	0.006	3.13E-09	-0.022	0.019	1.80E-01
SmoInit	rs12918191	G	0.24	-0.020	0.003	3.12E-11	0.016	0.009	1.00E-01
SmoInit	rs4785187	A	0.22	0.020	0.003	6.72E-11	0.010	0.009	2.90E-01
SmoInit	rs4788676	C	0.23	-0.018	0.003	4.97E-09	0.004	0.009	7.00E-01
SmoInit	rs62052916	T	0.07	-0.032	0.005	1.60E-10	0.005	0.015	7.90E-01
SmoInit	rs6497840	A	0.71	0.023	0.003	1.96E-15	0.003	0.009	5.70E-01
SmoInit	rs7188873	G	0.61	0.020	0.003	8.57E-15	0.015	0.008	5.10E-02
SmoInit	rs7192140	C	0.50	-0.017	0.003	3.46E-11	-0.005	0.008	5.60E-01
SmoInit	rs8050598	T	0.25	0.019	0.003	1.77E-10	0.013	0.009	2.10E-01
SmoInit	rs9302604	G	0.44	0.019	0.003	3.34E-13	0.002	0.008	7.60E-01
SmoInit	rs9922607	T	0.20	-0.022	0.003	3.48E-12	-0.002	0.010	8.00E-01
SmoInit	rs9941217	G	0.35	-0.019	0.003	3.48E-12	0.000	0.008	9.70E-01
SmoInit	rs11651955	A	0.50	-0.014	0.003	3.70E-08	0.000	0.008	7.90E-01
SmoInit	rs17692129	T	0.33	0.020	0.003	4.53E-13	-0.007	0.008	4.50E-01
SmoInit	rs2344976	C	0.61	-0.015	0.003	7.90E-09	-0.009	0.008	3.10E-01
SmoInit	rs2587507	C	0.50	-0.015	0.003	8.75E-09	0.015	0.008	5.30E-02
SmoInit	rs28441558	C	0.06	-0.036	0.006	1.24E-10	0.022	0.016	1.90E-01
SmoInit	rs4790874	T	0.53	0.017	0.003	8.27E-12	-0.007	0.008	4.20E-01
SmoInit	rs67777803	T	0.17	-0.025	0.003	3.17E-13	0.006	0.010	4.60E-01
SmoInit	rs75919030	C	0.27	-0.021	0.003	3.34E-13	0.012	0.009	3.30E-01
SmoInit	rs11872397	A	0.25	-0.017	0.003	5.24E-09	0.004	0.009	6.20E-01
SmoInit	rs1373178	G	0.59	-0.020	0.003	4.21E-15	-0.006	0.008	5.50E-01
SmoInit	rs2359180	G	0.37	-0.014	0.003	5.04E-08	0.000	0.013	9.70E-01
SmoInit	rs34342129	C	0.51	-0.014	0.003	2.10E-08	0.004	0.008	5.90E-01
SmoInit	rs4476253	A	0.24	-0.018	0.003	5.76E-10	-0.002	0.009	9.30E-01
SmoInit	rs62098013	A	0.37	0.018	0.003	2.18E-11	-0.005	0.008	5.80E-01
SmoInit	rs67050670	G	0.23	-0.020	0.003	2.30E-11	-0.030	0.009	8.30E-04
SmoInit	rs71367544	T	0.20	0.021	0.003	8.67E-11	-0.009	0.010	4.20E-01
SmoInit	rs72898831	G	0.16	-0.024	0.004	4.05E-12	-0.011	0.010	3.40E-01
SmoInit	rs72938304	A	0.11	-0.027	0.004	1.38E-11	0.013	0.012	2.30E-01
SmoInit	rs7505855	T	0.59	-0.017	0.003	5.20E-11	-0.005	0.008	6.20E-01
SmoInit	rs8096225	C	0.70	0.016	0.003	2.58E-08	-0.005	0.008	5.00E-01
SmoInit	rs10853981	A	0.33	0.015	0.003	4.79E-08	0.020	0.008	2.10E-02
SmoInit	rs1126757	T	0.47	0.014	0.003	2.86E-08	0.003	0.008	6.30E-01
SmoInit	rs113230003	A	0.26	-0.019	0.003	1.06E-10	0.011	0.009	2.10E-01
SmoInit	rs117734003	C	0.07	0.030	0.005	2.55E-09	0.034	0.016	2.70E-02

SmoInit	rs76608582	A	0.05	-0.035	0.006	4.97E-09	-0.039	0.019	6.20E-02
SmoInit	rs8103660	C	0.35	0.016	0.003	2.98E-09	0.002	0.008	6.90E-01
SmoInit	rs3810496	C	0.62	0.016	0.003	1.53E-09	0.019	0.008	1.00E-02
SmoInit	rs6011779	T	0.81	-0.019	0.003	2.83E-09	-0.007	0.010	5.00E-01
SmoInit	rs6050446	G	0.97	0.054	0.008	8.78E-13	0.034	0.022	1.10E-01
SmoInit	rs6073075	A	0.82	-0.019	0.003	2.45E-08	-0.013	0.010	2.00E-01
SmoInit	rs910912	C	0.74	-0.017	0.003	7.90E-09	0.013	0.009	1.50E-01
SmoInit	rs4818005	A	0.58	-0.020	0.003	1.10E-14	0.002	0.008	9.40E-01
SmoInit	rs4822102	T	0.62	-0.017	0.003	2.81E-10	0.014	0.008	6.50E-02
SmoInit	rs9627272	C	0.41	-0.015	0.003	2.42E-09	0.008	0.008	2.30E-01
T2DM	rs10159026	C	0.75	0.027	0.005	2.94E-09	0.009	0.009	2.40E-01
T2DM	rs10737818	G	0.62	0.035	0.005	1.29E-11	0.004	0.008	4.10E-01
T2DM	rs10889560	C	0.89	-0.038	0.006	2.40E-09	-0.004	0.014	8.60E-01
T2DM	rs10916780	G	0.25	-0.034	0.005	1.05E-13	0.001	0.010	9.80E-01
T2DM	rs11240351	G	0.47	0.026	0.004	1.55E-10	0.004	0.008	5.10E-01
T2DM	rs1127215	C	0.60	0.041	0.004	4.37E-26	-0.011	0.008	8.70E-02
T2DM	rs11583755	C	0.33	0.037	0.004	2.26E-19	-0.005	0.008	6.10E-01
T2DM	rs1194592	G	0.47	-0.024	0.004	5.47E-10	-0.018	0.008	2.40E-02
T2DM	rs12041243	G	0.24	-0.029	0.005	4.55E-10	-0.023	0.010	3.30E-02
T2DM	rs12116935	G	0.35	0.024	0.004	1.82E-08	0.004	0.008	5.80E-01
T2DM	rs12128213	G	0.47	-0.026	0.004	1.09E-09	-0.004	0.008	4.80E-01
T2DM	rs12746673	C	0.21	0.031	0.005	3.26E-10	0.003	0.009	1.00E+00
T2DM	rs1327123	G	0.64	-0.024	0.004	5.47E-10	-0.003	0.008	7.50E-01
T2DM	rs145904381	C	0.01	-0.161	0.022	3.56E-13	-0.049	0.036	2.30E-01
T2DM	rs197374	C	0.59	-0.022	0.004	9.27E-09	-0.006	0.008	6.00E-01
T2DM	rs2269247	C	0.81	0.036	0.005	3.72E-13	-0.001	0.010	9.60E-01
T2DM	rs2282456	G	0.72	-0.035	0.004	6.43E-17	0.004	0.008	3.90E-01
T2DM	rs2453051	C	0.15	0.061	0.006	1.80E-23	0.000	0.013	8.90E-01
T2DM	rs2613499	G	0.20	-0.036	0.005	7.43E-12	-0.003	0.010	8.50E-01
T2DM	rs2816177	G	0.40	0.022	0.004	9.27E-09	-0.011	0.008	1.90E-01
T2DM	rs2820446	G	0.30	-0.041	0.004	3.31E-22	-0.024	0.008	5.80E-03
T2DM	rs3020781	G	0.37	0.029	0.004	8.08E-12	0.024	0.009	2.50E-03
T2DM	rs340874	C	0.50	0.054	0.004	4.59E-44	0.015	0.008	3.50E-02
T2DM	rs348330	G	0.45	0.050	0.005	3.46E-20	0.012	0.008	9.80E-02
T2DM	rs3753693	C	0.55	0.026	0.004	1.83E-10	-0.003	0.008	8.90E-01
T2DM	rs4129858	G	0.42	0.022	0.004	4.40E-09	-0.007	0.008	3.80E-01
T2DM	rs41304257	G	0.24	-0.032	0.005	1.32E-11	-0.001	0.009	9.10E-01
T2DM	rs4655617	C	0.48	0.026	0.004	1.55E-10	0.013	0.008	1.00E-01
T2DM	rs4658234	G	0.77	-0.030	0.005	7.28E-09	0.013	0.009	3.40E-01
T2DM	rs539515	C	0.21	0.036	0.005	7.10E-15	0.002	0.010	8.20E-01
T2DM	rs59020573	G	0.91	-0.050	0.009	1.09E-08	-0.034	0.023	1.40E-01
T2DM	rs61779284	G	0.80	-0.070	0.005	1.43E-48	-0.009	0.010	3.50E-01
T2DM	rs61817176	C	0.45	-0.026	0.004	4.87E-11	0.000	0.008	9.60E-01
T2DM	rs7546252	G	0.57	-0.026	0.004	1.66E-10	-0.018	0.008	1.80E-02
T2DM	rs7554251	C	0.70	0.030	0.005	4.11E-11	0.000	0.009	9.90E-01
T2DM	rs79090772	C	0.10	-0.073	0.007	2.73E-28	-0.008	0.014	5.40E-01
T2DM	rs9438610	G	0.20	-0.031	0.005	8.40E-10	-0.003	0.010	7.40E-01
T2DM	rs10184004	C	0.60	0.064	0.004	2.87E-54	0.036	0.008	7.70E-06
T2DM	rs10188334	C	0.84	0.054	0.005	1.02E-24	-0.005	0.010	6.80E-01
T2DM	rs10469860	G	0.28	-0.024	0.004	3.11E-08	-0.009	0.009	4.00E-01
T2DM	rs11096542	G	0.38	-0.025	0.004	3.48E-09	-0.014	0.008	7.40E-02
T2DM	rs113135335	G	0.10	-0.053	0.007	2.85E-13	-0.033	0.013	7.60E-03
T2DM	rs113414093	G	0.95	-0.072	0.013	1.55E-08	-0.004	0.021	9.80E-01
T2DM	rs1260326	C	0.58	0.063	0.004	8.46E-58	-0.039	0.008	4.10E-07
T2DM	rs12986742	C	0.47	0.035	0.004	1.55E-20	0.011	0.008	2.30E-01
T2DM	rs13406280	C	0.57	0.027	0.004	5.29E-12	-0.003	0.008	7.20E-01

T2DM	rs13415288	C	0.37	0.026	0.004	5.75E-11	0.018	0.008	2.30E-02
T2DM	rs1877712	G	0.45	0.021	0.004	6.29E-08	0.018	0.008	1.50E-02
T2DM	rs243018	G	0.50	0.051	0.004	8.75E-39	-0.003	0.008	6.30E-01
T2DM	rs2723065	G	0.38	-0.042	0.004	2.75E-27	-0.001	0.008	9.30E-01
T2DM	rs28758542	G	0.34	-0.026	0.004	1.09E-09	-0.011	0.008	2.30E-01
T2DM	rs2943650	C	0.36	-0.081	0.004	7.03E-82	-0.018	0.008	1.80E-02
T2DM	rs34329895	G	0.54	-0.026	0.004	1.83E-10	-0.007	0.008	4.70E-01
T2DM	rs34506349	G	0.96	0.067	0.012	9.88E-09	0.015	0.019	4.40E-01
T2DM	rs34845373	G	0.26	-0.033	0.005	3.44E-12	-0.019	0.009	1.40E-02
T2DM	rs3828242	G	0.27	-0.025	0.004	4.62E-09	-0.013	0.008	1.30E-01
T2DM	rs3845843	C	0.49	-0.026	0.004	3.72E-12	0.004	0.008	6.40E-01
T2DM	rs4482463	C	0.24	0.032	0.006	1.67E-08	0.024	0.015	1.20E-01
T2DM	rs4671799	G	0.67	-0.026	0.004	5.75E-11	0.003	0.008	9.30E-01
T2DM	rs4673712	C	0.29	-0.027	0.005	2.26E-09	-0.020	0.008	2.80E-02
T2DM	rs4832290	C	0.76	-0.027	0.005	5.07E-08	-0.005	0.009	5.60E-01
T2DM	rs5010712	G	0.81	0.030	0.005	4.04E-09	-0.007	0.011	3.80E-01
T2DM	rs62182438	T	0.26	0.026	0.005	1.12E-08	-0.003	0.009	5.70E-01
T2DM	rs66877183	T	0.43	-0.050	0.009	2.60E-08	-0.007	0.010	6.00E-01
T2DM	rs6710938	C	0.23	-0.034	0.005	1.05E-13	-0.019	0.009	2.90E-02
T2DM	rs6712905	C	0.32	0.028	0.004	3.08E-11	-0.010	0.009	2.80E-01
T2DM	rs6714523	G	0.88	-0.040	0.006	4.91E-11	0.007	0.012	6.60E-01
T2DM	rs6715901	G	0.57	0.023	0.004	8.92E-09	0.006	0.008	4.50E-01
T2DM	rs6716394	G	0.48	0.028	0.004	1.07E-13	0.007	0.008	4.80E-01
T2DM	rs6741676	G	0.31	-0.032	0.004	1.22E-14	-0.022	0.008	3.10E-03
T2DM	rs72803684	C	0.96	-0.067	0.012	6.98E-09	0.033	0.018	6.20E-02
T2DM	rs74677818	C	0.86	-0.036	0.006	2.68E-09	-0.025	0.011	4.20E-02
T2DM	rs76675804	C	0.09	-0.121	0.007	6.63E-60	-0.004	0.013	7.50E-01
T2DM	rs77424687	C	0.70	0.027	0.005	2.58E-09	0.025	0.010	1.40E-02
T2DM	rs838720	G	0.38	0.034	0.004	2.83E-18	0.006	0.008	4.00E-01
T2DM	rs9308614	G	0.13	-0.057	0.006	8.56E-23	-0.035	0.011	4.80E-04
T2DM	rs11708067	G	0.22	-0.080	0.005	3.52E-58	0.023	0.009	1.10E-02
T2DM	rs11929640	G	0.65	-0.025	0.004	5.43E-09	-0.013	0.009	1.90E-01
T2DM	rs12494424	G	0.96	-0.066	0.012	2.06E-08	-0.043	0.020	1.70E-02
T2DM	rs13059382	G	0.72	0.036	0.006	1.60E-09	-0.032	0.015	2.20E-02
T2DM	rs13094957	C	0.22	-0.064	0.005	7.59E-42	0.005	0.010	6.20E-01
T2DM	rs13099581	C	0.82	0.037	0.006	1.59E-10	-0.002	0.011	9.20E-01
T2DM	rs13434089	C	0.20	-0.061	0.005	1.75E-31	-0.002	0.012	8.00E-01
T2DM	rs1470560	G	0.64	-0.025	0.004	9.07E-10	-0.024	0.008	8.00E-03
T2DM	rs17036160	C	0.89	0.101	0.007	3.91E-54	0.046	0.012	2.50E-04
T2DM	rs2255703	C	0.40	-0.026	0.004	5.23E-11	-0.003	0.008	7.00E-01
T2DM	rs28712435	C	0.55	0.025	0.004	7.38E-11	-0.003	0.008	5.40E-01
T2DM	rs2872246	C	0.60	-0.027	0.004	2.93E-11	-0.010	0.008	1.90E-01
T2DM	rs3872707	G	0.84	-0.037	0.005	7.29E-12	-0.008	0.012	6.00E-01
T2DM	rs56243018	C	0.05	-0.083	0.010	1.15E-15	-0.010	0.018	6.30E-01
T2DM	rs59489841	C	0.70	0.024	0.004	2.20E-08	0.009	0.009	3.00E-01
T2DM	rs61579137	G	0.66	-0.031	0.005	4.88E-11	-0.011	0.011	2.80E-01
T2DM	rs62255926	T	0.26	-0.025	0.005	4.30E-08	-0.008	0.008	2.60E-01
T2DM	rs62271373	T	0.95	-0.065	0.010	1.70E-10	-0.046	0.017	6.40E-03
T2DM	rs6549112	G	0.33	-0.027	0.004	2.08E-11	-0.012	0.008	2.20E-01
T2DM	rs667920	G	0.23	-0.032	0.005	6.02E-13	-0.016	0.009	1.10E-01
T2DM	rs66815886	G	0.69	0.040	0.004	1.67E-21	0.009	0.009	3.70E-01
T2DM	rs6766859	C	0.39	0.028	0.004	6.20E-12	0.000	0.008	8.40E-01
T2DM	rs6777684	G	0.63	0.055	0.004	1.32E-35	0.002	0.008	8.30E-01
T2DM	rs6792892	C	0.48	0.031	0.004	2.01E-14	0.020	0.008	6.80E-03
T2DM	rs73146095	C	0.90	0.041	0.007	6.63E-09	0.001	0.012	7.20E-01
T2DM	rs76263492	G	0.96	-0.068	0.011	3.20E-09	-0.012	0.019	6.70E-01

T2DM	rs7633673	G	0.59	0.031	0.004	8.10E-16	0.005	0.008	4.80E-01
T2DM	rs7645613	C	0.36	0.034	0.005	1.41E-12	0.005	0.012	7.80E-01
T2DM	rs8192675	C	0.32	-0.045	0.004	5.21E-27	0.014	0.009	5.30E-02
T2DM	rs853866	T	0.61	0.024	0.004	2.93E-08	0.003	0.008	7.70E-01
T2DM	rs924753	G	0.46	0.026	0.004	1.55E-11	0.015	0.008	5.30E-02
T2DM	rs978444	G	0.41	0.023	0.004	4.31E-09	0.011	0.008	1.10E-01
T2DM	rs9814945	C	0.71	-0.032	0.005	4.60E-11	0.011	0.009	1.70E-01
T2DM	rs9857204	G	0.71	-0.027	0.004	2.52E-10	-0.012	0.008	1.60E-01
T2DM	rs9859406	G	0.65	-0.112	0.004	1.33E-171	-0.001	0.008	7.70E-01
T2DM	rs9869477	G	0.56	0.023	0.004	2.72E-09	0.010	0.008	2.20E-01
T2DM	rs9872347	C	0.67	0.032	0.004	3.69E-14	0.001	0.008	9.70E-01
T2DM	rs9873519	C	0.53	-0.037	0.004	9.63E-23	-0.005	0.008	4.30E-01
T2DM	rs10937721	G	0.39	-0.076	0.004	5.15E-69	-0.003	0.008	6.60E-01
T2DM	rs10938398	G	0.61	-0.045	0.004	3.72E-30	-0.017	0.008	3.60E-02
T2DM	rs12505942	C	0.31	-0.029	0.004	2.56E-12	-0.015	0.008	7.10E-02
T2DM	rs13107325	C	0.93	-0.047	0.009	3.80E-08	-0.011	0.015	4.00E-01
T2DM	rs17035289	C	0.21	0.036	0.005	1.03E-13	0.007	0.011	5.10E-01
T2DM	rs1996617	C	0.39	0.029	0.004	5.01E-13	-0.007	0.008	2.60E-01
T2DM	rs2125799	C	0.33	0.024	0.004	2.66E-09	0.003	0.008	5.90E-01
T2DM	rs2169033	C	0.34	-0.033	0.004	2.25E-15	-0.024	0.008	3.60E-03
T2DM	rs28408270	G	0.49	0.024	0.004	8.89E-10	-0.001	0.008	9.70E-01
T2DM	rs28819812	C	0.66	0.033	0.004	3.93E-15	0.018	0.008	3.10E-02
T2DM	rs2952858	G	0.60	-0.024	0.004	7.45E-09	0.000	0.009	9.90E-01
T2DM	rs34617913	G	0.30	-0.024	0.004	2.73E-08	-0.009	0.009	4.30E-01
T2DM	rs35901985	G	0.19	-0.033	0.005	3.75E-10	-0.008	0.010	4.00E-01
T2DM	rs362307	C	0.93	-0.048	0.009	3.44E-08	0.009	0.015	7.00E-01
T2DM	rs4440243	C	0.42	-0.025	0.004	1.06E-08	0.014	0.008	7.20E-02
T2DM	rs4833687	C	0.74	-0.027	0.005	2.97E-09	-0.008	0.009	4.30E-01
T2DM	rs4834232	C	0.79	-0.027	0.005	7.16E-09	0.001	0.009	9.80E-01
T2DM	rs55691245	G	0.87	0.056	0.006	1.86E-18	0.006	0.011	6.30E-01
T2DM	rs56337234	C	0.50	0.043	0.004	2.75E-25	-0.003	0.008	6.30E-01
T2DM	rs6813195	C	0.65	0.046	0.004	5.52E-31	-0.009	0.009	3.10E-01
T2DM	rs6835992	G	0.70	0.029	0.004	6.90E-11	-0.008	0.008	3.70E-01
T2DM	rs730831	G	0.19	-0.088	0.007	3.33E-34	-0.032	0.021	1.10E-01
T2DM	rs73221116	G	0.96	-0.096	0.011	2.07E-19	0.015	0.020	5.00E-01
T2DM	rs7659468	G	0.50	-0.029	0.004	8.71E-15	-0.004	0.008	5.40E-01
T2DM	rs7664347	C	0.66	-0.023	0.004	1.15E-08	-0.003	0.008	8.10E-01
T2DM	rs7674402	G	0.77	-0.052	0.006	3.04E-16	-0.013	0.020	4.30E-01
T2DM	rs993380	G	0.64	-0.027	0.004	3.07E-12	-0.012	0.008	1.40E-01
T2DM	rs9991328	C	0.48	-0.023	0.004	2.32E-09	-0.015	0.008	4.10E-02
T2DM	rs114136102	C	0.04	0.063	0.011	3.11E-08	0.027	0.020	1.30E-01
T2DM	rs12187734	C	0.57	0.029	0.004	1.85E-13	0.005	0.008	5.80E-01
T2DM	rs144052331	C	0.05	-0.071	0.010	4.67E-13	-0.067	0.024	8.10E-03
T2DM	rs1650505	G	0.77	-0.041	0.005	1.00E-19	-0.023	0.010	1.80E-02
T2DM	rs217256	C	0.49	0.021	0.004	1.38E-08	0.005	0.008	6.60E-01
T2DM	rs2591392	G	0.62	0.031	0.004	4.29E-15	0.009	0.008	1.50E-01
T2DM	rs261967	C	0.41	0.024	0.004	6.24E-10	0.001	0.008	9.20E-01
T2DM	rs2662390	C	0.74	-0.028	0.005	8.92E-09	0.008	0.011	4.90E-01
T2DM	rs2913873	G	0.14	-0.032	0.006	1.67E-08	-0.008	0.011	4.40E-01
T2DM	rs302395	G	0.32	0.023	0.004	1.53E-08	0.010	0.008	1.40E-01
T2DM	rs329118	C	0.58	-0.028	0.004	1.42E-13	0.017	0.008	3.00E-02
T2DM	rs34341	T	0.52	0.040	0.004	4.98E-25	0.014	0.008	5.70E-02
T2DM	rs4343858	G	0.72	0.030	0.005	5.54E-11	0.016	0.009	7.50E-02
T2DM	rs4479849	G	0.59	-0.026	0.004	3.71E-11	-0.005	0.008	5.90E-01
T2DM	rs464605	C	0.35	-0.070	0.004	2.35E-65	-0.013	0.009	1.70E-01
T2DM	rs4865796	G	0.29	-0.048	0.004	2.29E-30	-0.025	0.008	2.10E-03

T2DM	rs4976033	G	0.43	0.025	0.004	1.23E-10	0.012	0.008	1.60E-01
T2DM	rs6870983	C	0.74	0.029	0.005	4.71E-09	0.007	0.010	4.20E-01
T2DM	rs6885157	G	0.78	0.037	0.007	4.49E-08	-0.005	0.012	7.20E-01
T2DM	rs6897117	C	0.71	-0.023	0.004	2.53E-08	-0.004	0.008	6.70E-01
T2DM	rs75432112	G	0.95	-0.132	0.010	7.38E-37	-0.030	0.018	1.00E-01
T2DM	rs7732130	G	0.30	0.055	0.005	4.54E-32	0.010	0.008	2.20E-01
T2DM	rs10440833	T	0.70	-0.129	0.004	5.91E-216	0.000	0.009	9.20E-01
T2DM	rs10806906	C	0.45	0.022	0.004	1.08E-08	0.014	0.008	1.10E-01
T2DM	rs11155073	C	0.52	-0.028	0.004	1.77E-12	-0.014	0.008	5.30E-02
T2DM	rs11759026	G	0.32	0.059	0.006	1.03E-24	0.000	0.009	8.70E-01
T2DM	rs16881572	G	0.94	-0.068	0.011	5.98E-10	-0.006	0.023	8.70E-01
T2DM	rs2031847	G	0.97	-0.075	0.013	1.57E-08	-0.016	0.028	5.60E-01
T2DM	rs2394186	G	0.22	-0.027	0.005	1.64E-08	-0.021	0.010	6.60E-02
T2DM	rs2608953	C	0.79	-0.035	0.005	3.40E-12	-0.009	0.010	4.90E-01
T2DM	rs2876354	C	0.52	0.047	0.004	1.98E-34	0.001	0.008	8.10E-01
T2DM	rs3130931	C	0.68	0.048	0.004	6.57E-32	0.006	0.008	4.60E-01
T2DM	rs33959228	C	0.98	0.092	0.016	3.56E-09	-0.010	0.027	6.40E-01
T2DM	rs34247110	G	0.52	-0.031	0.004	1.00E-15	-0.010	0.008	1.60E-01
T2DM	rs34298980	C	0.47	-0.035	0.006	1.22E-10	-0.014	0.008	5.80E-02
T2DM	rs35164294	G	0.93	-0.053	0.009	1.15E-08	0.007	0.016	7.40E-01
T2DM	rs3798519	C	0.21	0.047	0.005	6.43E-24	-0.009	0.010	3.80E-01
T2DM	rs4709746	C	0.87	0.057	0.006	1.01E-21	0.029	0.011	1.80E-02
T2DM	rs501470	G	0.46	-0.032	0.004	3.73E-17	-0.003	0.008	7.20E-01
T2DM	rs538801	T	0.43	-0.021	0.004	6.29E-08	-0.011	0.008	1.50E-01
T2DM	rs727734	T	0.24	-0.029	0.005	8.65E-11	-0.014	0.009	1.60E-01
T2DM	rs72846863	G	0.86	0.034	0.006	3.98E-08	0.023	0.011	4.00E-02
T2DM	rs75080135	C	0.21	-0.036	0.005	3.20E-13	-0.018	0.010	7.80E-02
T2DM	rs7742292	C	0.48	0.026	0.004	6.80E-11	0.000	0.008	9.70E-01
T2DM	rs7752666	C	0.70	0.031	0.004	1.31E-12	0.024	0.008	3.20E-03
T2DM	rs7758002	G	0.52	-0.032	0.004	4.36E-16	-0.009	0.008	4.50E-01
T2DM	rs7758115	G	0.50	-0.023	0.004	2.70E-09	0.005	0.008	6.10E-01
T2DM	rs80196932	C	0.18	-0.048	0.005	1.60E-19	0.000	0.011	9.20E-01
T2DM	rs9257408	G	0.49	-0.031	0.005	1.47E-10	-0.012	0.008	9.30E-02
T2DM	rs9358912	G	0.65	0.025	0.005	3.14E-08	0.020	0.009	1.30E-02
T2DM	rs9370243	G	0.83	-0.039	0.006	1.05E-10	-0.023	0.014	8.80E-02
T2DM	rs9379084	G	0.84	0.072	0.006	9.07E-31	0.003	0.013	7.10E-01
T2DM	rs9390022	C	0.34	-0.031	0.004	8.43E-14	0.000	0.008	8.90E-01
T2DM	rs9449295	C	0.52	0.022	0.004	3.05E-08	0.020	0.008	2.10E-02
T2DM	rs9472139	G	0.74	-0.038	0.004	1.28E-17	0.000	0.009	9.20E-01
T2DM	rs10228796	G	0.57	0.067	0.004	5.55E-70	0.001	0.008	7.30E-01
T2DM	rs12539264	G	0.27	0.029	0.005	1.16E-10	0.004	0.009	4.70E-01
T2DM	rs12667919	C	0.71	-0.026	0.005	5.13E-08	0.002	0.009	8.20E-01
T2DM	rs12669521	G	0.30	-0.026	0.004	2.27E-09	-0.017	0.008	3.90E-02
T2DM	rs13237518	C	0.52	-0.028	0.004	9.52E-14	-0.010	0.008	1.90E-01
T2DM	rs13238568	G	0.38	0.025	0.004	1.46E-09	0.012	0.008	1.50E-01
T2DM	rs17439448	C	0.88	-0.039	0.007	4.47E-09	-0.001	0.012	8.70E-01
T2DM	rs187653072	C	0.03	0.097	0.014	2.43E-12	0.020	0.022	2.90E-01
T2DM	rs1922879	G	0.63	0.023	0.004	2.03E-08	0.015	0.008	5.00E-02
T2DM	rs2188848	G	0.21	-0.033	0.005	1.28E-11	0.017	0.010	5.90E-02
T2DM	rs243513	G	0.71	0.023	0.004	6.84E-08	0.004	0.008	7.60E-01
T2DM	rs3735491	C	0.69	-0.025	0.004	6.10E-09	-0.005	0.009	6.90E-01
T2DM	rs3779272	T	0.58	0.021	0.004	2.08E-08	0.004	0.008	6.60E-01
T2DM	rs38221	C	0.76	-0.029	0.005	1.16E-09	0.005	0.009	8.20E-01
T2DM	rs3996350	G	0.56	0.035	0.004	5.72E-19	0.011	0.008	8.70E-02
T2DM	rs4252505	G	0.18	0.041	0.007	3.64E-09	0.016	0.016	3.10E-01
T2DM	rs506597	G	0.88	0.041	0.006	1.49E-10	-0.007	0.012	6.40E-01

T2DM	rs583769	G	0.72	-0.031	0.004	9.36E-13	-0.009	0.009	3.20E-01
T2DM	rs60251368	G	0.07	0.055	0.008	1.94E-12	0.015	0.018	5.50E-01
T2DM	rs62452060	G	0.86	-0.035	0.006	1.97E-09	-0.001	0.011	8.60E-01
T2DM	rs62492368	G	0.60	-0.031	0.004	3.32E-14	-0.007	0.009	3.80E-01
T2DM	rs6946660	C	0.41	-0.047	0.004	1.02E-33	-0.008	0.008	4.30E-01
T2DM	rs6956980	C	0.47	0.028	0.004	7.80E-14	-0.003	0.008	5.90E-01
T2DM	rs6972291	C	0.35	0.028	0.005	3.73E-09	0.008	0.010	2.60E-01
T2DM	rs6975279	C	0.71	-0.043	0.004	1.05E-24	-0.022	0.009	8.70E-03
T2DM	rs6976111	C	0.72	-0.026	0.005	2.04E-08	-0.003	0.008	7.00E-01
T2DM	rs6978327	C	0.41	-0.023	0.004	5.71E-09	-0.007	0.008	3.80E-01
T2DM	rs730497	G	0.83	-0.055	0.005	2.45E-28	0.012	0.010	3.20E-01
T2DM	rs73121277	C	0.30	0.026	0.004	6.00E-10	0.007	0.009	4.60E-01
T2DM	rs73184014	G	0.21	-0.030	0.005	1.00E-08	0.000	0.009	8.50E-01
T2DM	rs860262	C	0.57	0.075	0.004	8.99E-82	0.021	0.008	9.70E-03
T2DM	rs917195	C	0.72	0.042	0.005	3.04E-20	0.014	0.009	1.00E-01
T2DM	rs10096633	C	0.82	0.033	0.005	9.89E-10	0.012	0.012	3.10E-01
T2DM	rs10101067	G	0.91	-0.039	0.007	1.46E-08	-0.029	0.015	4.00E-02
T2DM	rs10808671	G	0.54	-0.036	0.004	4.47E-21	-0.007	0.008	3.20E-01
T2DM	rs112515915	G	0.07	-0.056	0.009	3.80E-11	-0.003	0.015	8.50E-01
T2DM	rs11994255	C	0.72	-0.024	0.004	2.73E-08	-0.011	0.009	2.40E-01
T2DM	rs11998023	G	0.18	0.032	0.005	9.76E-10	0.008	0.010	4.00E-01
T2DM	rs12056338	G	0.61	-0.025	0.004	3.50E-10	0.008	0.008	2.50E-01
T2DM	rs12680217	C	0.30	-0.040	0.007	2.25E-08	-0.002	0.017	9.60E-01
T2DM	rs13262861	C	0.83	0.102	0.005	9.91E-80	-0.017	0.010	1.50E-01
T2DM	rs13266634	C	0.68	0.102	0.004	1.13E-137	-0.006	0.008	6.50E-01
T2DM	rs13268508	C	0.63	-0.038	0.004	4.70E-20	0.012	0.008	1.30E-01
T2DM	rs13365225	G	0.26	0.033	0.005	1.17E-12	0.011	0.011	3.10E-01
T2DM	rs1561927	C	0.31	0.033	0.005	5.30E-13	0.004	0.009	4.80E-01
T2DM	rs17772814	G	0.92	0.069	0.012	3.31E-08	-0.013	0.015	3.40E-01
T2DM	rs2679745	G	0.81	0.028	0.005	1.40E-08	0.017	0.010	1.00E-01
T2DM	rs2725370	C	0.71	-0.032	0.005	4.07E-12	-0.005	0.008	4.60E-01
T2DM	rs28792187	G	0.07	0.041	0.008	5.11E-08	0.000	0.016	9.20E-01
T2DM	rs35753840	C	0.34	0.025	0.004	1.47E-09	0.012	0.008	1.40E-01
T2DM	rs3802219	C	0.61	-0.036	0.004	5.49E-20	-0.019	0.008	1.50E-02
T2DM	rs3887059	G	0.74	-0.030	0.004	6.86E-12	0.001	0.009	8.50E-01
T2DM	rs4294149	C	0.60	-0.022	0.004	2.64E-08	-0.011	0.008	1.20E-01
T2DM	rs510062	G	0.40	-0.022	0.004	1.69E-08	-0.003	0.008	7.40E-01
T2DM	rs60384372	G	0.45	-0.035	0.004	5.25E-17	-0.005	0.008	5.50E-01
T2DM	rs6558173	G	0.66	-0.025	0.004	9.07E-10	0.004	0.008	6.10E-01
T2DM	rs10119430	G	0.22	0.028	0.005	1.32E-09	-0.003	0.010	7.20E-01
T2DM	rs10758950	T	0.71	-0.026	0.005	7.57E-09	-0.018	0.010	1.10E-01
T2DM	rs10811661	C	0.24	-0.147	0.005	3.00E-206	0.003	0.010	7.10E-01
T2DM	rs10818763	C	0.68	0.036	0.005	2.22E-12	0.024	0.012	6.10E-02
T2DM	rs10993072	C	0.66	-0.032	0.004	1.55E-14	-0.004	0.008	7.10E-01
T2DM	rs12001437	C	0.41	0.030	0.004	2.36E-15	0.028	0.008	3.80E-04
T2DM	rs12380322	G	0.39	0.030	0.004	1.64E-13	-0.023	0.008	3.80E-03
T2DM	rs1412234	C	0.29	0.040	0.004	3.31E-21	0.004	0.008	7.20E-01
T2DM	rs1431819	G	0.71	0.029	0.005	1.64E-10	0.007	0.009	3.40E-01
T2DM	rs1567353	G	0.36	0.028	0.004	3.65E-12	0.004	0.008	7.10E-01
T2DM	rs1570247	G	0.60	0.024	0.004	3.94E-10	-0.014	0.008	7.00E-02
T2DM	rs1885234	G	0.39	0.024	0.004	3.94E-10	0.009	0.008	2.10E-01
T2DM	rs2150999	C	0.54	0.024	0.004	4.64E-10	-0.008	0.008	3.00E-01
T2DM	rs2796441	G	0.56	0.061	0.004	5.72E-55	-0.015	0.008	5.10E-02
T2DM	rs28642213	G	0.76	0.082	0.005	1.73E-68	0.010	0.009	3.30E-01
T2DM	rs379417	G	0.65	-0.028	0.004	3.62E-11	-0.004	0.009	5.60E-01
T2DM	rs4237150	G	0.57	-0.043	0.004	1.23E-31	0.006	0.008	4.60E-01

T2DM	rs529565	C	0.36	0.042	0.004	1.92E-26	0.023	0.008	4.70E-03
T2DM	rs654629	G	0.61	0.027	0.004	9.07E-12	-0.009	0.008	2.70E-01
T2DM	rs6597649	C	0.52	-0.023	0.004	1.97E-09	-0.019	0.008	8.80E-03
T2DM	rs67269808	G	0.07	-0.073	0.008	7.13E-21	-0.028	0.016	9.50E-02
T2DM	rs7029718	G	0.60	-0.027	0.004	2.56E-12	-0.002	0.008	7.10E-01
T2DM	rs73642097	G	0.21	0.029	0.005	2.87E-09	0.002	0.011	9.20E-01
T2DM	rs75619936	G	0.13	0.031	0.006	2.06E-08	0.011	0.013	4.20E-01
T2DM	rs9411425	G	0.35	0.024	0.004	3.64E-09	0.014	0.008	4.00E-02
T2DM	rs10787518	T	0.45	0.029	0.005	1.03E-08	0.019	0.008	2.70E-02
T2DM	rs10998338	G	0.52	-0.031	0.004	2.85E-15	-0.002	0.008	8.90E-01
T2DM	rs1111875	C	0.55	0.093	0.004	1.02E-131	0.030	0.008	3.00E-05
T2DM	rs111765639	G	0.88	0.058	0.008	4.62E-12	-0.037	0.022	7.40E-02
T2DM	rs11199116	C	0.19	0.031	0.005	9.36E-10	0.001	0.010	9.40E-01
T2DM	rs11257655	C	0.70	-0.095	0.006	5.08E-64	-0.016	0.010	9.60E-02
T2DM	rs11258422	C	0.41	0.024	0.004	4.15E-09	-0.004	0.008	5.70E-01
T2DM	rs1408579	C	0.56	0.028	0.004	5.85E-11	0.019	0.008	1.60E-02
T2DM	rs146716733	C	0.92	0.045	0.008	1.14E-08	0.010	0.018	6.60E-01
T2DM	rs2280141	G	0.51	-0.046	0.005	2.56E-21	-0.020	0.008	8.60E-03
T2DM	rs2482506	G	0.24	-0.032	0.005	2.18E-12	0.004	0.009	6.20E-01
T2DM	rs2633311	C	0.50	0.024	0.004	3.12E-09	0.010	0.008	3.00E-01
T2DM	rs3012060	T	0.19	0.034	0.006	5.03E-10	0.022	0.012	4.90E-02
T2DM	rs3122231	C	0.66	0.027	0.004	7.32E-10	0.014	0.008	1.10E-01
T2DM	rs35011184	G	0.78	-0.251	0.005	0.00E+00	-0.001	0.009	9.40E-01
T2DM	rs36062478	C	0.18	0.039	0.005	5.86E-13	0.008	0.011	6.30E-01
T2DM	rs524903	G	0.82	0.043	0.007	1.68E-10	0.028	0.010	1.70E-02
T2DM	rs620191	G	0.66	-0.023	0.004	1.15E-08	-0.001	0.009	8.00E-01
T2DM	rs697239	C	0.45	-0.059	0.004	7.86E-54	0.001	0.008	8.20E-01
T2DM	rs705145	C	0.65	-0.023	0.004	1.97E-09	0.008	0.008	2.80E-01
T2DM	rs7071036	C	0.20	-0.051	0.007	2.82E-12	0.009	0.027	7.40E-01
T2DM	rs71495046	C	0.12	0.040	0.006	2.40E-10	0.012	0.014	4.70E-01
T2DM	rs7912336	T	0.55	0.022	0.004	1.13E-08	0.013	0.008	1.10E-01
T2DM	rs827237	C	0.81	-0.029	0.005	2.90E-08	-0.007	0.010	4.60E-01
T2DM	rs945187	G	0.42	0.031	0.004	1.88E-15	0.007	0.008	3.80E-01
T2DM	rs10750397	G	0.71	-0.040	0.004	1.67E-21	-0.001	0.009	8.50E-01
T2DM	rs10750840	T	0.55	-0.024	0.004	8.89E-10	-0.006	0.008	4.90E-01
T2DM	rs10766076	T	0.71	0.026	0.004	1.48E-09	-0.001	0.009	8.30E-01
T2DM	rs10767659	G	0.35	0.026	0.004	6.00E-10	0.019	0.008	2.80E-02
T2DM	rs10830963	G	0.31	0.073	0.004	7.59E-68	-0.002	0.009	6.00E-01
T2DM	rs10835690	T	0.82	-0.029	0.005	2.45E-08	-0.032	0.010	1.80E-03
T2DM	rs11236524	C	0.24	0.036	0.006	1.41E-08	-0.031	0.014	2.20E-02
T2DM	rs11602873	T	0.15	-0.099	0.006	8.08E-63	-0.004	0.011	6.70E-01
T2DM	rs117316450	G	0.02	0.133	0.017	8.10E-15	0.030	0.027	2.40E-01
T2DM	rs12419690	G	0.51	0.030	0.004	9.33E-14	0.002	0.008	8.10E-01
T2DM	rs12789028	G	0.82	-0.046	0.005	4.31E-20	-0.007	0.010	4.90E-01
T2DM	rs149027146	T	0.06	0.074	0.010	1.28E-12	0.028	0.016	8.40E-02
T2DM	rs1662185	G	0.58	-0.023	0.004	3.54E-08	-0.012	0.009	1.40E-01
T2DM	rs16907058	G	0.94	-0.046	0.008	4.06E-08	0.008	0.018	7.40E-01
T2DM	rs174541	C	0.35	-0.028	0.004	1.42E-11	-0.001	0.008	9.10E-01
T2DM	rs2237897	C	0.76	0.220	0.007	2.03E-229	0.023	0.019	2.90E-01
T2DM	rs2513505	C	0.41	-0.024	0.004	2.69E-10	-0.016	0.008	4.10E-02
T2DM	rs2956092	C	0.65	-0.025	0.004	2.15E-10	-0.004	0.008	5.60E-01
T2DM	rs35251247	G	0.71	-0.031	0.004	1.88E-13	-0.016	0.009	4.90E-02
T2DM	rs3918298	G	0.88	0.104	0.010	8.93E-25	-0.008	0.024	6.80E-01
T2DM	rs4929965	G	0.61	-0.068	0.004	2.76E-58	-0.021	0.008	1.70E-02
T2DM	rs529623	C	0.50	-0.022	0.004	3.75E-09	-0.010	0.008	1.70E-01
T2DM	rs62618693	C	0.96	0.082	0.011	1.60E-13	0.034	0.019	1.00E-01

T2DM	rs7127212	C	0.61	-0.023	0.004	3.08E-08	-0.010	0.008	1.20E-01
T2DM	rs7130522	C	0.33	0.022	0.004	6.14E-08	-0.006	0.009	4.40E-01
T2DM	rs7483027	C	0.33	-0.027	0.004	7.41E-11	-0.001	0.008	7.20E-01
T2DM	rs757110	C	0.37	0.060	0.004	3.08E-53	-0.003	0.008	7.80E-01
T2DM	rs76789970	C	0.89	-0.039	0.006	1.12E-10	-0.019	0.012	1.00E-01
T2DM	rs7941510	C	0.65	0.035	0.004	4.29E-17	0.031	0.009	2.20E-04
T2DM	rs10773000	G	0.67	0.034	0.004	1.11E-16	-0.004	0.008	5.90E-01
T2DM	rs10844519	G	0.41	0.030	0.004	9.14E-13	-0.003	0.009	9.20E-01
T2DM	rs10879261	G	0.39	0.034	0.004	2.87E-19	0.012	0.008	1.70E-01
T2DM	rs11048457	G	0.39	0.039	0.004	3.44E-19	0.012	0.009	1.40E-01
T2DM	rs11108094	C	0.93	-0.057	0.009	8.66E-11	-0.017	0.015	3.00E-01
T2DM	rs11114650	G	0.78	-0.045	0.008	2.52E-09	0.018	0.036	6.50E-01
T2DM	rs11173646	T	0.74	-0.029	0.005	1.33E-09	-0.021	0.010	1.50E-02
T2DM	rs11614914	C	0.70	-0.039	0.004	3.41E-19	-0.002	0.008	8.60E-01
T2DM	rs11830241	C	0.90	-0.041	0.007	1.14E-08	-0.005	0.015	7.70E-01
T2DM	rs12422600	G	0.64	0.024	0.004	1.97E-09	0.007	0.008	3.40E-01
T2DM	rs12825669	G	0.67	0.023	0.004	3.54E-08	0.014	0.008	7.80E-02
T2DM	rs1426371	G	0.69	0.043	0.005	1.52E-21	0.008	0.009	3.90E-01
T2DM	rs2066827	G	0.29	0.032	0.005	3.09E-10	0.013	0.009	1.40E-01
T2DM	rs2257883	G	0.74	-0.071	0.005	1.41E-47	-0.011	0.011	3.10E-01
T2DM	rs2277339	G	0.14	0.044	0.006	6.02E-15	0.020	0.013	1.30E-01
T2DM	rs2408252	C	0.54	-0.026	0.004	8.28E-10	0.008	0.008	2.40E-01
T2DM	rs2730827	C	0.51	-0.028	0.004	2.10E-13	-0.007	0.008	4.30E-01
T2DM	rs2732469	T	0.58	0.032	0.004	5.32E-14	-0.009	0.008	3.50E-01
T2DM	rs3751239	G	0.22	-0.069	0.005	2.19E-48	-0.005	0.010	5.20E-01
T2DM	rs4930726	C	0.33	-0.035	0.004	7.32E-18	-0.025	0.008	2.50E-03
T2DM	rs56348580	G	0.71	0.051	0.005	3.96E-27	-0.008	0.008	4.20E-01
T2DM	rs6538805	C	0.47	-0.030	0.004	7.90E-15	0.004	0.008	6.00E-01
T2DM	rs7132908	G	0.64	-0.030	0.004	1.13E-13	0.001	0.008	9.40E-01
T2DM	rs7134150	G	0.90	-0.044	0.007	2.47E-10	0.011	0.016	3.80E-01
T2DM	rs7316626	G	0.80	-0.032	0.005	2.48E-09	-0.006	0.010	5.90E-01
T2DM	rs76895963	G	0.02	-0.422	0.020	4.73E-97	-0.112	0.030	1.70E-04
T2DM	rs79310463	C	0.83	-0.050	0.005	3.95E-21	-0.024	0.011	3.70E-02
T2DM	rs884847	G	0.80	-0.029	0.005	4.63E-09	-0.006	0.010	5.70E-01
T2DM	rs11616380	G	0.73	0.076	0.004	1.26E-70	-0.011	0.009	1.70E-01
T2DM	rs314879	C	0.23	0.036	0.005	2.51E-15	0.004	0.009	7.00E-01
T2DM	rs34584161	G	0.28	-0.057	0.004	9.67E-38	-0.003	0.009	6.80E-01
T2DM	rs4397977	G	0.58	-0.028	0.004	1.60E-11	0.000	0.008	9.70E-01
T2DM	rs57286125	G	0.83	-0.061	0.005	7.22E-33	0.008	0.010	3.20E-01
T2DM	rs61967710	G	0.94	-0.055	0.010	2.19E-08	-0.015	0.024	4.80E-01
T2DM	rs6561273	G	0.40	-0.030	0.005	5.65E-09	0.007	0.008	4.00E-01
T2DM	rs7991679	T	0.76	0.027	0.005	1.14E-08	0.016	0.011	8.60E-02
T2DM	rs9316500	G	0.35	-0.040	0.004	1.37E-22	0.004	0.009	5.50E-01
T2DM	rs9515905	G	0.26	-0.047	0.004	7.58E-27	0.009	0.009	4.60E-01
T2DM	rs9560114	T	0.24	-0.026	0.005	3.58E-08	-0.018	0.009	5.30E-02
T2DM	rs9564268	C	0.54	-0.024	0.004	1.24E-09	0.013	0.008	1.10E-01
T2DM	rs9587811	C	0.55	0.025	0.004	6.74E-11	-0.005	0.008	4.20E-01
T2DM	rs112324411	C	0.94	0.073	0.010	1.47E-12	-0.010	0.016	4.90E-01
T2DM	rs11848361	G	0.92	0.048	0.009	1.87E-08	0.004	0.013	8.00E-01
T2DM	rs12433335	C	0.57	-0.021	0.004	2.08E-08	-0.018	0.008	2.10E-02
T2DM	rs12586772	T	0.40	-0.023	0.004	5.03E-09	0.002	0.008	9.50E-01
T2DM	rs12883788	C	0.59	-0.033	0.004	2.96E-16	0.001	0.008	8.50E-01
T2DM	rs12892257	G	0.49	0.029	0.005	8.41E-09	0.003	0.008	7.50E-01
T2DM	rs2056857	C	0.59	0.026	0.004	5.23E-11	-0.003	0.008	6.20E-01
T2DM	rs242105	C	0.41	0.039	0.006	3.84E-11	0.015	0.009	8.30E-02
T2DM	rs4902002	G	0.35	0.025	0.004	1.97E-09	-0.003	0.009	7.30E-01

T2DM	rs4906272	C	0.73	-0.030	0.005	8.03E-11	-0.001	0.011	8.70E-01
T2DM	rs56365443	G	0.32	-0.024	0.004	1.46E-08	-0.015	0.008	4.40E-02
T2DM	rs712315	T	0.78	0.028	0.005	3.21E-08	0.013	0.011	2.20E-01
T2DM	rs7147483	C	0.34	-0.035	0.004	9.05E-18	0.003	0.009	5.90E-01
T2DM	rs7156625	G	0.77	-0.053	0.005	2.88E-27	0.001	0.009	9.60E-01
T2DM	rs723355	G	0.56	0.024	0.004	1.23E-09	0.014	0.008	9.10E-02
T2DM	rs8010382	G	0.47	0.027	0.004	2.56E-12	0.001	0.008	9.30E-01
T2DM	rs11858759	G	0.73	0.024	0.004	4.06E-08	0.020	0.008	1.60E-02
T2DM	rs12910361	G	0.61	0.069	0.004	6.29E-66	0.021	0.009	1.50E-02
T2DM	rs148106383	C	0.97	-0.093	0.016	2.70E-09	0.030	0.024	3.00E-01
T2DM	rs149336329	G	0.96	0.087	0.010	3.62E-18	0.015	0.019	3.90E-01
T2DM	rs1812707	C	0.44	-0.023	0.004	5.87E-09	0.005	0.008	5.70E-01
T2DM	rs2289739	G	0.69	-0.036	0.006	3.64E-11	-0.013	0.008	7.70E-02
T2DM	rs2290203	G	0.70	-0.051	0.004	5.74E-32	-0.006	0.010	5.90E-01
T2DM	rs2447198	C	0.22	-0.034	0.006	6.34E-10	0.003	0.013	7.10E-01
T2DM	rs4776970	T	0.45	-0.029	0.004	7.23E-13	-0.014	0.008	7.20E-02
T2DM	rs4777857	G	0.44	0.026	0.004	6.22E-11	-0.012	0.008	1.70E-01
T2DM	rs4923864	G	0.69	0.057	0.006	1.26E-20	-0.002	0.015	9.30E-01
T2DM	rs59646751	G	0.69	-0.024	0.004	4.15E-09	0.001	0.008	8.30E-01
T2DM	rs6495182	C	0.68	0.041	0.004	5.27E-22	0.009	0.009	2.70E-01
T2DM	rs7163757	C	0.56	0.043	0.004	1.10E-29	-0.008	0.008	2.70E-01
T2DM	rs7169799	C	0.45	0.021	0.004	2.08E-08	0.005	0.008	6.00E-01
T2DM	rs7178762	C	0.49	0.032	0.004	3.33E-15	0.005	0.008	4.70E-01
T2DM	rs8043085	G	0.70	-0.041	0.004	3.31E-22	-0.002	0.009	8.40E-01
T2DM	rs893617	C	0.28	0.054	0.004	3.40E-39	0.005	0.009	6.80E-01
T2DM	rs9479	G	0.52	0.027	0.004	2.40E-13	0.004	0.008	6.10E-01
T2DM	rs1075855	G	0.28	0.026	0.005	5.08E-09	-0.012	0.009	1.80E-01
T2DM	rs111283203	G	0.76	-0.026	0.005	4.55E-08	-0.006	0.010	5.90E-01
T2DM	rs11646052	G	0.41	0.026	0.004	2.62E-11	0.003	0.008	5.60E-01
T2DM	rs12918782	G	0.26	0.031	0.005	5.63E-11	0.024	0.010	2.20E-02
T2DM	rs1421085	C	0.37	0.118	0.004	6.57E-192	0.024	0.008	2.60E-03
T2DM	rs2216063	G	0.20	-0.030	0.005	4.22E-08	0.001	0.013	9.70E-01
T2DM	rs244415	G	0.63	0.040	0.004	1.37E-22	0.014	0.008	7.30E-02
T2DM	rs4384608	C	0.41	0.027	0.004	6.17E-10	0.005	0.008	5.50E-01
T2DM	rs4788815	T	0.65	0.023	0.004	7.70E-09	0.013	0.008	1.50E-01
T2DM	rs55857387	C	0.25	-0.044	0.005	9.07E-22	-0.009	0.010	4.10E-01
T2DM	rs56823429	C	0.27	0.042	0.005	1.56E-20	0.019	0.009	2.40E-02
T2DM	rs72802365	G	0.93	0.107	0.008	4.32E-41	0.030	0.014	4.10E-02
T2DM	rs8054556	G	0.57	-0.032	0.004	4.36E-16	0.001	0.008	9.70E-01
T2DM	rs8056890	G	0.68	-0.030	0.004	2.16E-12	-0.019	0.008	2.30E-02
T2DM	rs8061528	C	0.76	-0.035	0.005	1.48E-14	-0.012	0.010	2.10E-01
T2DM	rs9927842	C	0.79	-0.031	0.005	8.25E-10	-0.021	0.011	5.50E-02
T2DM	rs9937296	C	0.77	0.045	0.007	2.06E-10	-0.021	0.011	1.40E-01
T2DM	rs11078916	C	0.65	-0.033	0.004	8.37E-15	-0.027	0.009	2.40E-03
T2DM	rs1109442	C	0.46	0.022	0.004	3.82E-09	0.002	0.008	9.80E-01
T2DM	rs11150745	G	0.32	-0.031	0.005	3.94E-09	-0.004	0.008	6.30E-01
T2DM	rs11651755	C	0.47	0.066	0.004	3.60E-68	0.004	0.008	8.00E-01
T2DM	rs117642733	C	0.96	-0.076	0.013	1.01E-08	-0.013	0.019	3.70E-01
T2DM	rs11870735	C	0.82	-0.031	0.005	6.07E-09	0.002	0.010	8.60E-01
T2DM	rs12603589	C	0.31	0.042	0.005	5.58E-20	0.013	0.010	2.50E-01
T2DM	rs17810376	G	0.30	0.028	0.005	2.92E-09	-0.026	0.008	1.40E-03
T2DM	rs2040792	C	0.46	0.031	0.004	1.88E-15	-0.003	0.008	5.80E-01
T2DM	rs2297508	G	0.56	-0.031	0.004	2.44E-14	-0.027	0.008	7.30E-04
T2DM	rs35895680	C	0.71	0.050	0.005	2.48E-26	0.001	0.008	8.60E-01
T2DM	rs366577	C	0.49	0.022	0.004	2.86E-08	0.004	0.008	4.90E-01
T2DM	rs372558	G	0.42	-0.021	0.004	4.72E-08	-0.003	0.008	8.00E-01

T2DM	rs3744347	G	0.25	-0.030	0.005	6.01E-11	-0.010	0.010	3.00E-01
T2DM	rs4335	G	0.48	0.030	0.004	1.24E-15	-0.005	0.008	6.90E-01
T2DM	rs57767539	G	0.94	-0.062	0.009	8.19E-12	-0.019	0.015	2.40E-01
T2DM	rs61736066	G	0.88	0.042	0.006	4.76E-11	-0.018	0.014	2.80E-01
T2DM	rs676387	C	0.68	-0.042	0.004	3.44E-24	-0.007	0.009	4.00E-01
T2DM	rs7224711	C	0.52	0.031	0.004	6.53E-16	0.001	0.008	7.70E-01
T2DM	rs8071043	C	0.33	0.047	0.004	8.18E-30	-0.005	0.008	6.00E-01
T2DM	rs858519	C	0.50	-0.024	0.004	1.97E-09	-0.017	0.008	2.70E-02
T2DM	rs9913225	G	0.37	0.026	0.004	5.75E-11	-0.006	0.008	4.70E-01
T2DM	rs10048404	C	0.66	0.031	0.005	6.78E-09	-0.009	0.008	3.60E-01
T2DM	rs12454712	C	0.41	-0.042	0.004	1.57E-27	-0.016	0.008	5.70E-02
T2DM	rs17747955	C	0.45	0.022	0.004	2.14E-08	0.005	0.008	5.20E-01
T2DM	rs2032217	G	0.67	-0.027	0.004	2.08E-11	-0.015	0.009	1.10E-01
T2DM	rs303760	C	0.69	-0.033	0.004	3.18E-14	-0.005	0.008	3.70E-01
T2DM	rs410150	C	0.21	0.029	0.005	1.74E-09	0.012	0.010	2.00E-01
T2DM	rs6565922	C	0.58	-0.028	0.004	8.44E-13	0.024	0.008	2.90E-03
T2DM	rs6567160	C	0.24	0.054	0.004	1.68E-34	-0.004	0.009	8.60E-01
T2DM	rs7227272	G	0.82	0.041	0.006	3.20E-13	-0.016	0.013	1.90E-01
T2DM	rs7240767	C	0.48	0.034	0.005	2.62E-11	0.006	0.008	5.30E-01
T2DM	rs9319943	C	0.21	-0.031	0.005	1.39E-10	-0.009	0.010	5.30E-01
T2DM	rs9958640	G	0.60	-0.024	0.004	1.92E-08	0.004	0.009	5.30E-01
T2DM	rs10404726	C	0.48	0.026	0.004	1.27E-09	0.006	0.008	4.90E-01
T2DM	rs10408163	C	0.60	0.032	0.004	1.18E-13	-0.003	0.009	7.00E-01
T2DM	rs12185519	C	0.81	-0.036	0.005	2.62E-11	-0.025	0.010	1.20E-02
T2DM	rs142385484	C	0.85	0.034	0.006	2.14E-08	0.022	0.011	3.60E-02
T2DM	rs2115107	G	0.59	-0.036	0.004	5.49E-20	-0.017	0.008	3.20E-02
T2DM	rs2867570	G	0.57	0.026	0.004	2.62E-11	0.010	0.008	2.00E-01
T2DM	rs429358	C	0.15	-0.059	0.006	2.02E-26	-0.121	0.011	1.50E-29
T2DM	rs4805881	C	0.62	-0.038	0.004	2.53E-22	-0.010	0.008	2.20E-01
T2DM	rs4807125	C	0.85	-0.033	0.006	3.29E-08	-0.016	0.011	1.40E-01
T2DM	rs58542926	C	0.93	-0.072	0.007	1.32E-23	-0.289	0.015	2.80E-85
T2DM	rs7246440	G	0.62	0.030	0.004	3.56E-12	0.000	0.009	9.10E-01
T2DM	rs75253922	C	0.18	0.040	0.005	9.74E-14	0.017	0.010	3.90E-02
T2DM	rs8107527	G	0.62	-0.054	0.004	7.92E-42	-0.009	0.009	3.60E-01
T2DM	rs9384	G	0.66	0.040	0.004	6.61E-23	-0.001	0.008	8.00E-01
T2DM	rs12625671	C	0.23	0.060	0.006	5.72E-28	0.008	0.013	5.10E-01
T2DM	rs17265513	C	0.19	0.033	0.006	2.76E-09	0.008	0.010	2.80E-01
T2DM	rs2252115	G	0.43	-0.024	0.004	1.23E-09	-0.003	0.008	5.80E-01
T2DM	rs4809906	G	0.62	0.035	0.004	6.41E-18	0.017	0.008	2.70E-02
T2DM	rs4810145	C	0.52	0.030	0.004	1.54E-15	0.000	0.008	9.40E-01
T2DM	rs6011155	C	0.41	-0.028	0.004	3.29E-13	-0.017	0.008	2.00E-02
T2DM	rs6021276	C	0.63	-0.028	0.004	1.49E-12	0.003	0.008	8.10E-01
T2DM	rs6059662	G	0.70	0.037	0.004	9.37E-18	0.017	0.008	2.60E-02
T2DM	rs6066138	G	0.74	0.042	0.005	4.03E-19	-0.001	0.009	8.00E-01
T2DM	rs6073143	C	0.73	0.030	0.004	4.88E-12	0.015	0.009	1.30E-01
T2DM	rs6137042	G	0.70	0.026	0.005	2.20E-08	0.004	0.010	5.90E-01
T2DM	rs7274134	C	0.67	0.025	0.004	1.70E-09	0.011	0.009	3.30E-01
T2DM	rs11913442	C	0.38	-0.025	0.004	9.07E-10	-0.003	0.008	7.70E-01
T2DM	rs138771	G	0.69	-0.026	0.004	3.95E-09	-0.002	0.010	8.10E-01
T2DM	rs1801645	C	0.28	0.037	0.005	9.48E-17	-0.004	0.009	8.50E-01
T2DM	rs2240716	C	0.71	-0.026	0.004	4.43E-10	0.009	0.008	2.10E-01
T2DM	rs3747207	G	0.72	-0.041	0.004	7.70E-21	-0.192	0.009	2.60E-92
T2DM	rs56392746	G	0.92	0.058	0.008	2.57E-14	0.009	0.013	4.60E-01
T2DM	rs5762925	C	0.38	0.027	0.004	7.59E-12	0.003	0.008	8.20E-01
T2DM	rs75307421	G	0.97	-0.074	0.013	2.36E-08	-0.003	0.031	9.70E-01
Television	rs10737620	T	0.27	0.014	0.002	2.55E-09	0.004	0.009	5.10E-01

Television	rs11810109	A	0.70	0.016	0.002	5.38E-12	0.002	0.008	7.80E-01
Television	rs148648822	G	0.84	-0.018	0.003	4.22E-09	0.000	0.011	9.80E-01
Television	rs17379561	A	0.86	-0.025	0.003	1.08E-16	-0.019	0.011	5.70E-02
Television	rs4845364	A	0.50	-0.015	0.002	1.28E-12	0.003	0.008	6.00E-01
Television	rs631130	T	0.37	-0.014	0.002	5.04E-09	-0.007	0.008	4.90E-01
Television	rs6673341	T	0.47	-0.015	0.002	2.23E-11	-0.003	0.008	5.80E-01
Television	rs72725224	A	0.98	-0.046	0.008	3.74E-09	-0.022	0.028	4.70E-01
Television	rs984409	G	0.36	-0.015	0.002	4.13E-11	-0.013	0.008	1.00E-01
Television	rs10189857	A	0.57	-0.020	0.002	6.19E-21	0.007	0.008	2.90E-01
Television	rs10932837	C	0.49	-0.013	0.002	1.34E-09	-0.008	0.008	2.80E-01
Television	rs11689199	A	0.60	0.018	0.002	5.47E-17	0.005	0.008	4.70E-01
Television	rs12105701	C	0.40	-0.013	0.002	5.43E-09	-0.004	0.008	6.20E-01
Television	rs1451533	G	0.72	-0.016	0.002	1.09E-10	0.005	0.009	3.80E-01
Television	rs2971640	G	0.51	-0.013	0.002	4.20E-09	0.003	0.008	7.80E-01
Television	rs374722	G	0.15	0.024	0.003	5.49E-16	-0.003	0.011	5.30E-01
Television	rs3754970	T	0.50	-0.015	0.002	4.80E-12	-0.001	0.008	7.10E-01
Television	rs4577309	A	0.47	0.016	0.002	1.56E-13	-0.006	0.008	5.00E-01
Television	rs4973576	C	0.30	-0.015	0.002	1.04E-09	0.007	0.009	3.90E-01
Television	rs62641636	A	0.69	0.014	0.002	6.99E-10	0.009	0.008	3.10E-01
Television	rs6721975	T	0.23	-0.017	0.003	1.71E-10	-0.015	0.009	8.90E-02
Television	rs72781699	G	0.80	-0.019	0.003	3.05E-12	-0.015	0.010	1.60E-01
Television	rs7564130	T	0.64	-0.015	0.002	2.77E-11	0.004	0.008	6.80E-01
Television	rs11130793	C	0.60	0.013	0.002	5.04E-09	-0.006	0.008	6.00E-01
Television	rs114600294	G	0.79	-0.016	0.003	7.88E-10	-0.014	0.009	1.30E-01
Television	rs11714337	G	0.57	0.014	0.002	4.72E-11	0.010	0.008	2.30E-01
Television	rs12491503	G	0.67	-0.014	0.002	5.55E-10	-0.009	0.008	2.90E-01
Television	rs2034768	A	0.49	0.015	0.002	8.62E-12	0.012	0.008	9.20E-02
Television	rs3796386	G	0.57	-0.026	0.002	3.23E-33	-0.013	0.008	8.40E-02
Television	rs56398417	C	0.69	0.014	0.002	5.87E-10	0.007	0.008	3.30E-01
Television	rs6797840	A	0.46	-0.016	0.002	1.67E-13	-0.008	0.008	3.10E-01
Television	rs9834970	T	0.50	0.013	0.002	3.27E-09	0.005	0.008	5.60E-01
Television	rs9867121	C	0.82	0.019	0.003	3.88E-12	0.007	0.010	4.50E-01
Television	rs13107325	C	0.93	-0.029	0.004	1.49E-12	-0.011	0.015	4.00E-01
Television	rs34811474	G	0.77	0.015	0.003	2.28E-09	0.011	0.009	2.70E-01
Television	rs66852340	C	0.78	-0.018	0.003	7.91E-12	0.005	0.009	6.90E-01
Television	rs6825241	C	0.54	-0.017	0.002	4.92E-15	-0.008	0.008	2.00E-01
Television	rs6850494	A	0.62	-0.014	0.002	1.13E-10	-0.012	0.008	7.90E-02
Television	rs7693082	G	0.30	0.015	0.002	1.65E-10	0.013	0.009	1.20E-01
Television	rs7693703	G	0.91	0.023	0.004	2.73E-09	-0.005	0.014	8.10E-01
Television	rs10041724	T	0.81	0.018	0.003	3.92E-11	0.007	0.010	4.80E-01
Television	rs10054327	G	0.58	0.017	0.002	3.45E-15	0.024	0.008	4.10E-03
Television	rs1031423	T	0.22	-0.019	0.003	1.76E-12	0.001	0.009	7.90E-01
Television	rs262890	A	0.70	-0.019	0.002	3.21E-15	-0.002	0.008	7.20E-01
Television	rs42210	G	0.29	-0.014	0.002	7.35E-09	0.006	0.009	4.50E-01
Television	rs57585211	T	0.83	-0.017	0.003	4.33E-09	-0.005	0.010	5.60E-01
Television	rs62379379	G	0.93	-0.026	0.004	7.79E-10	-0.016	0.015	3.10E-01
Television	rs72828890	C	0.87	0.019	0.003	4.95E-09	0.020	0.012	7.00E-02
Television	rs7716447	A	0.64	-0.013	0.002	3.64E-09	-0.007	0.008	4.90E-01
Television	rs10947452	T	0.35	0.014	0.002	6.36E-10	0.005	0.008	5.90E-01
Television	rs17789218	T	0.76	0.019	0.003	1.39E-13	-0.012	0.009	2.00E-01
Television	rs2184364	A	0.78	0.016	0.003	3.00E-09	0.021	0.009	8.40E-03
Television	rs3135044	C	0.64	-0.015	0.002	8.68E-11	-0.017	0.008	3.60E-02
Television	rs6905544	A	0.40	-0.019	0.002	8.46E-18	-0.021	0.008	5.00E-03
Television	rs72834698	G	0.86	0.023	0.003	2.74E-13	0.011	0.011	3.20E-01
Television	rs77589760	C	0.96	0.037	0.006	4.08E-10	-0.002	0.021	9.50E-01
Television	rs78394231	T	0.90	-0.021	0.004	9.67E-09	0.015	0.013	2.40E-01

Television	rs9471333	C	0.45	0.013	0.002	1.54E-09	0.012	0.008	8.40E-02
Television	rs9718104	T	0.94	-0.041	0.005	9.31E-19	-0.027	0.016	1.30E-01
Television	rs17568389	T	0.49	0.015	0.002	8.57E-13	0.006	0.008	5.60E-01
Television	rs62471080	G	0.54	-0.013	0.002	3.66E-09	-0.004	0.008	4.90E-01
Television	rs7788008	G	0.57	0.013	0.002	5.88E-09	0.005	0.008	4.70E-01
Television	rs6472942	T	0.57	-0.013	0.002	1.77E-09	0.015	0.008	4.30E-02
Television	rs7834121	G	0.50	-0.014	0.002	1.11E-10	0.006	0.008	3.50E-01
Television	rs10739499	C	0.34	0.014	0.002	1.52E-09	-0.008	0.008	2.60E-01
Television	rs12342024	G	0.89	-0.021	0.003	1.74E-09	-0.041	0.012	9.50E-04
Television	rs12554512	T	0.58	0.021	0.002	3.76E-21	-0.002	0.008	7.10E-01
Television	rs2073869	C	0.83	0.019	0.003	1.54E-10	0.005	0.010	9.10E-01
Television	rs2291681	G	0.58	0.013	0.002	7.91E-09	-0.001	0.008	9.50E-01
Television	rs2616830	G	0.46	0.016	0.002	2.89E-14	-0.011	0.008	8.10E-02
Television	rs34864022	A	0.93	-0.026	0.004	1.41E-09	0.009	0.016	5.10E-01
Television	rs4382592	T	0.30	0.014	0.002	6.68E-09	0.013	0.008	5.90E-02
Television	rs7043521	A	0.43	0.014	0.002	6.49E-11	0.013	0.008	9.80E-02
Television	rs870151	T	0.53	-0.016	0.002	8.82E-13	-0.003	0.008	7.20E-01
Television	rs10786658	A	0.41	-0.014	0.002	4.63E-11	0.008	0.008	2.50E-01
Television	rs11245482	T	0.61	-0.013	0.002	2.57E-09	-0.008	0.008	2.60E-01
Television	rs1243182	C	0.69	-0.019	0.002	2.03E-15	-0.018	0.008	5.70E-02
Television	rs17727474	C	0.83	0.018	0.003	3.07E-09	0.013	0.011	1.60E-01
Television	rs2045147	A	0.45	0.013	0.002	5.87E-09	-0.011	0.008	1.80E-01
Television	rs11218575	C	0.57	0.015	0.002	2.08E-12	0.004	0.008	7.00E-01
Television	rs17207890	G	0.66	0.016	0.002	6.73E-12	0.010	0.008	3.40E-01
Television	rs648044	A	0.40	-0.014	0.002	1.03E-09	-0.014	0.008	6.40E-02
Television	rs74802478	G	0.82	0.017	0.003	3.52E-09	0.003	0.010	7.20E-01
Television	rs801733	A	0.64	0.017	0.002	7.32E-14	0.003	0.008	8.00E-01
Television	rs10771746	C	0.72	-0.014	0.002	2.46E-09	-0.002	0.009	7.60E-01
Television	rs10772643	C	0.11	0.025	0.003	1.32E-12	0.015	0.012	2.40E-01
Television	rs10876864	G	0.43	-0.013	0.002	1.01E-09	-0.011	0.008	2.50E-01
Television	rs2173650	G	0.85	0.018	0.003	4.56E-09	0.008	0.011	4.30E-01
Television	rs74996610	C	0.95	-0.031	0.005	4.10E-09	0.037	0.019	4.00E-02
Television	rs8756	C	0.48	-0.013	0.002	5.33E-10	-0.024	0.008	2.90E-03
Television	rs553397860	A	0.84	0.017	0.003	8.90E-09	0.009	0.011	3.60E-01
Television	rs56858768	G	0.70	-0.015	0.002	3.40E-10	-0.002	0.009	7.80E-01
Television	rs7991062	C	0.66	-0.018	0.002	8.82E-15	-0.004	0.008	8.10E-01
Television	rs9563168	G	0.79	0.018	0.003	4.30E-11	-0.005	0.010	6.70E-01
Television	rs9569734	A	0.84	0.019	0.003	3.38E-10	0.016	0.011	1.10E-01
Television	rs10145592	C	0.41	-0.015	0.002	1.78E-11	-0.013	0.008	1.40E-01
Television	rs2460	G	0.74	-0.015	0.002	4.96E-10	-0.010	0.009	3.10E-01
Television	rs61331678	G	0.57	0.015	0.002	6.06E-12	0.000	0.008	9.20E-01
Television	rs142710267	T	0.65	0.016	0.002	6.65E-12	-0.009	0.008	2.60E-01
Television	rs7184800	G	0.70	0.017	0.002	8.18E-13	0.016	0.008	4.70E-02
Television	rs7189927	T	0.36	0.015	0.002	3.36E-11	0.019	0.008	2.50E-02
Television	rs749671	G	0.63	0.016	0.002	2.67E-12	0.002	0.008	8.10E-01
Television	rs2447098	C	0.48	-0.015	0.002	6.82E-12	-0.004	0.008	6.90E-01
Television	rs2584597	T	0.66	0.015	0.002	2.92E-10	-0.008	0.009	3.80E-01
Television	rs9902312	T	0.68	0.015	0.002	4.55E-11	-0.010	0.008	2.00E-01
Television	rs303753	G	0.65	-0.014	0.002	2.74E-10	-0.006	0.008	3.50E-01
Television	rs9964724	C	0.32	0.018	0.002	3.34E-14	0.003	0.008	6.50E-01
Television	rs111901094	G	0.82	-0.017	0.003	1.98E-09	-0.106	0.010	1.80E-25
Television	rs7248205	C	0.40	0.014	0.002	3.35E-10	0.003	0.008	6.70E-01
Television	rs56103247	C	0.94	0.030	0.005	3.78E-10	0.042	0.017	5.70E-03
Television	rs6131281	C	0.60	0.016	0.002	3.07E-13	0.009	0.008	1.70E-01
Television	rs6141814	C	0.61	-0.014	0.002	1.31E-09	-0.004	0.008	7.60E-01
TG	rs10797996	C	0.43	0.014	0.002	5.33E-12	0.020	0.008	7.00E-03

TG	rs10863828	T	0.76	0.013	0.002	1.79E-08	-0.001	0.009	8.90E-01
TG	rs11118310	A	0.41	-0.019	0.002	2.33E-21	-0.017	0.008	4.60E-02
TG	rs11122450	T	0.39	0.048	0.002	9.96E-124	0.014	0.008	9.40E-02
TG	rs11206374	G	0.78	-0.025	0.002	8.75E-26	-0.001	0.009	8.30E-01
TG	rs11240358	G	0.61	-0.013	0.002	4.03E-11	-0.006	0.008	4.30E-01
TG	rs114052230	C	0.83	0.020	0.003	3.48E-13	0.017	0.011	6.70E-02
TG	rs114165349	G	0.98	-0.082	0.007	3.96E-35	-0.027	0.026	2.70E-01
TG	rs12138136	T	0.91	0.025	0.004	4.62E-12	0.006	0.014	7.60E-01
TG	rs12749691	A	0.70	0.020	0.002	1.09E-20	-0.003	0.008	7.30E-01
TG	rs1365297	A	0.82	0.018	0.003	9.21E-13	-0.003	0.010	8.00E-01
TG	rs184694823	A	0.92	-0.021	0.004	1.80E-08	-0.010	0.014	5.60E-01
TG	rs1938566	C	0.17	0.021	0.003	1.61E-15	0.015	0.010	1.00E-01
TG	rs2131311	A	0.29	0.012	0.002	3.32E-08	0.018	0.009	3.80E-02
TG	rs213494	C	0.35	-0.016	0.002	7.63E-14	-0.011	0.008	2.10E-01
TG	rs320369	A	0.32	0.012	0.002	1.13E-08	-0.007	0.008	2.50E-01
TG	rs36043408	G	0.50	0.013	0.002	1.04E-10	0.009	0.008	2.10E-01
TG	rs61780049	A	0.85	-0.015	0.003	3.17E-08	-0.025	0.011	2.60E-02
TG	rs61830291	A	0.90	-0.029	0.003	1.26E-17	-0.023	0.013	7.40E-02
TG	rs6424109	C	0.13	-0.020	0.003	3.13E-11	0.007	0.012	5.10E-01
TG	rs6690181	T	0.62	0.014	0.002	1.48E-11	0.015	0.008	6.50E-02
TG	rs6700266	G	0.66	0.013	0.002	2.05E-09	-0.010	0.008	3.10E-01
TG	rs72904737	G	0.91	0.027	0.004	1.22E-14	0.009	0.014	4.70E-01
TG	rs880315	T	0.66	0.012	0.002	1.57E-08	0.011	0.008	7.60E-02
TG	rs9436661	T	0.65	0.078	0.002	5.14E-304	-0.004	0.008	8.30E-01
TG	rs9970140	A	0.92	0.025	0.004	1.45E-11	-0.035	0.015	2.20E-02
TG	rs1009360	T	0.58	0.019	0.002	2.99E-20	0.009	0.008	3.20E-01
TG	rs10172544	C	0.59	0.011	0.002	1.55E-08	0.012	0.008	8.40E-02
TG	rs10176110	T	0.87	-0.024	0.003	8.37E-16	-0.010	0.012	3.40E-01
TG	rs10180284	C	0.52	0.011	0.002	8.69E-09	0.015	0.008	9.30E-02
TG	rs12472667	C	0.63	-0.014	0.002	2.21E-11	-0.018	0.008	2.60E-02
TG	rs13389219	C	0.61	0.037	0.002	7.74E-76	0.036	0.008	1.00E-05
TG	rs17326656	G	0.76	-0.018	0.002	4.13E-14	-0.022	0.009	1.40E-02
TG	rs182636083	C	0.48	0.027	0.002	7.38E-32	0.004	0.009	4.50E-01
TG	rs1861435	T	0.58	0.014	0.002	2.53E-12	0.015	0.008	5.80E-02
TG	rs2110690	A	0.49	-0.011	0.002	4.30E-08	-0.010	0.008	1.80E-01
TG	rs2382825	C	0.38	0.013	0.002	1.64E-10	0.030	0.008	8.80E-05
TG	rs2943645	C	0.35	-0.040	0.002	1.15E-83	-0.018	0.008	2.00E-02
TG	rs3731696	A	0.88	-0.022	0.003	6.42E-13	0.006	0.012	6.90E-01
TG	rs3820897	T	0.18	-0.020	0.003	3.82E-14	-0.015	0.010	1.40E-01
TG	rs4128205	A	0.49	-0.012	0.002	5.04E-09	-0.016	0.008	3.90E-02
TG	rs4662414	A	0.55	0.012	0.002	2.13E-09	-0.018	0.008	1.60E-02
TG	rs4665972	T	0.40	0.100	0.002	0.00E+00	0.037	0.008	1.50E-06
TG	rs4675812	G	0.41	0.014	0.002	9.79E-13	0.011	0.008	1.80E-01
TG	rs57074291	C	0.74	0.014	0.002	4.30E-10	0.003	0.009	7.40E-01
TG	rs58839393	A	0.84	-0.017	0.003	1.80E-10	-0.023	0.011	3.10E-02
TG	rs62130120	G	0.54	0.011	0.002	4.15E-08	-0.003	0.008	6.90E-01
TG	rs6531216	G	0.47	-0.018	0.002	2.40E-20	-0.016	0.008	7.20E-02
TG	rs6708784	A	0.51	0.013	0.002	1.90E-10	0.000	0.008	9.60E-01
TG	rs6722159	T	0.51	0.011	0.002	3.05E-08	0.009	0.008	2.00E-01
TG	rs676210	G	0.79	0.073	0.002	5.54E-197	0.004	0.010	6.10E-01
TG	rs72917533	T	0.81	0.014	0.003	3.79E-08	0.017	0.010	8.30E-02
TG	rs7424120	C	0.40	0.012	0.002	5.68E-09	-0.010	0.008	2.70E-01
TG	rs75225803	C	0.91	0.024	0.004	3.46E-11	0.018	0.014	1.70E-01
TG	rs7596814	G	0.71	0.013	0.002	7.31E-09	0.014	0.008	6.10E-02
TG	rs76172517	T	0.89	0.018	0.003	1.53E-08	0.020	0.012	1.40E-01
TG	rs77631110	A	0.98	-0.053	0.008	7.28E-11	0.029	0.031	3.70E-01

TG	rs78058190	G	0.95	-0.082	0.005	3.15E-57	-0.024	0.020	2.70E-01
TG	rs935168	G	0.35	-0.014	0.002	8.07E-12	-0.011	0.008	1.10E-01
TG	rs954244	C	0.75	-0.015	0.002	1.91E-11	-0.022	0.009	6.30E-03
TG	rs1152847	G	0.65	0.012	0.002	2.65E-08	0.006	0.008	5.00E-01
TG	rs13066793	A	0.91	0.022	0.003	1.05E-10	0.031	0.013	1.90E-02
TG	rs2194411	G	0.87	0.017	0.003	1.31E-08	0.036	0.012	2.10E-03
TG	rs2342371	G	0.27	0.013	0.002	1.16E-08	0.002	0.009	7.60E-01
TG	rs2455821	C	0.73	-0.013	0.002	2.25E-09	-0.018	0.009	3.80E-02
TG	rs3103310	A	0.76	-0.020	0.002	6.23E-18	-0.018	0.009	4.20E-02
TG	rs5402	T	0.88	-0.024	0.003	1.59E-15	-0.024	0.012	3.40E-02
TG	rs62271373	T	0.94	-0.042	0.004	5.70E-23	-0.046	0.017	6.40E-03
TG	rs6792725	A	0.31	0.015	0.002	3.51E-12	-0.005	0.009	5.00E-01
TG	rs6798755	C	0.93	0.025	0.004	2.45E-10	0.047	0.016	3.00E-03
TG	rs6800707	C	0.19	-0.030	0.003	1.24E-31	0.001	0.010	8.60E-01
TG	rs6805924	G	0.57	-0.011	0.002	4.79E-08	0.008	0.008	3.50E-01
TG	rs684773	A	0.23	-0.029	0.002	3.00E-35	-0.013	0.009	1.90E-01
TG	rs7631606	T	0.73	0.014	0.002	1.05E-09	0.014	0.009	1.10E-01
TG	rs79287178	G	0.97	-0.050	0.006	7.02E-17	-0.009	0.023	6.70E-01
TG	rs79983121	C	0.80	-0.015	0.002	9.02E-10	-0.023	0.010	9.60E-03
TG	rs9812100	G	0.52	0.013	0.002	2.25E-10	0.007	0.008	3.30E-01
TG	rs9831084	T	0.54	0.012	0.002	6.07E-09	0.012	0.008	1.60E-01
TG	rs11100083	T	0.77	0.016	0.002	1.36E-11	0.011	0.009	2.60E-01
TG	rs1126673	C	0.30	-0.014	0.002	1.40E-10	0.015	0.008	7.70E-02
TG	rs11722924	G	0.46	-0.013	0.002	1.08E-10	0.017	0.008	3.20E-02
TG	rs12504746	C	0.81	0.015	0.003	1.96E-09	0.006	0.010	5.70E-01
TG	rs13101719	T	0.58	-0.020	0.002	4.09E-16	-0.013	0.009	1.50E-01
TG	rs13101828	A	0.55	0.012	0.002	1.49E-09	0.008	0.008	2.30E-01
TG	rs13107325	C	0.93	-0.030	0.004	5.73E-15	-0.011	0.015	4.00E-01
TG	rs13108218	A	0.39	0.031	0.002	8.21E-50	0.005	0.008	5.60E-01
TG	rs1347188	A	0.75	-0.014	0.002	1.66E-09	-0.004	0.009	6.60E-01
TG	rs2035816	A	0.92	0.028	0.004	5.65E-15	0.062	0.014	9.20E-06
TG	rs2237029	G	0.40	0.014	0.002	8.47E-12	0.006	0.008	4.20E-01
TG	rs3775228	C	0.60	-0.034	0.002	2.55E-62	-0.005	0.008	5.60E-01
TG	rs3822072	G	0.55	-0.015	0.002	4.57E-14	-0.011	0.008	1.10E-01
TG	rs4450871	A	0.56	0.014	0.002	5.82E-12	0.014	0.008	1.30E-01
TG	rs71603401	A	0.86	-0.026	0.003	1.68E-19	-0.011	0.011	2.40E-01
TG	rs73243877	A	0.83	-0.029	0.003	9.68E-28	-0.017	0.010	7.60E-02
TG	rs7681288	G	0.34	-0.012	0.002	1.58E-08	0.018	0.008	3.30E-02
TG	rs7694869	A	0.38	0.011	0.002	4.92E-08	-0.007	0.008	3.30E-01
TG	rs78025076	C	0.98	-0.048	0.007	6.35E-12	0.009	0.028	5.00E-01
TG	rs1030472	A	0.79	-0.023	0.002	8.60E-21	-0.021	0.010	2.90E-02
TG	rs1045241	C	0.73	0.021	0.002	2.35E-20	0.005	0.009	5.40E-01
TG	rs112424890	C	0.82	-0.017	0.003	3.66E-11	-0.003	0.010	7.20E-01
TG	rs11746801	G	0.36	0.012	0.002	2.47E-09	0.015	0.008	6.60E-02
TG	rs1316753	G	0.61	0.015	0.002	8.34E-13	0.008	0.008	1.80E-01
TG	rs193735	G	0.96	-0.033	0.005	7.07E-10	0.020	0.020	2.90E-01
TG	rs325485	A	0.40	0.012	0.002	9.42E-09	0.017	0.008	1.90E-02
TG	rs34580448	T	0.96	0.034	0.005	1.06E-11	0.014	0.020	6.40E-01
TG	rs37538	G	0.40	0.014	0.002	1.42E-12	0.006	0.008	3.60E-01
TG	rs3936511	A	0.81	-0.046	0.003	6.03E-74	-0.041	0.010	3.90E-05
TG	rs4976033	A	0.60	-0.018	0.002	2.93E-18	-0.012	0.008	1.60E-01
TG	rs55646464	G	0.70	-0.012	0.002	1.29E-08	-0.004	0.008	4.70E-01
TG	rs62397245	C	0.78	-0.015	0.002	4.49E-10	-0.019	0.009	6.00E-02
TG	rs6882076	T	0.37	-0.033	0.002	1.93E-57	0.018	0.008	2.40E-02
TG	rs7244	G	0.83	-0.015	0.003	5.61E-09	-0.011	0.010	4.20E-01
TG	rs72801474	G	0.91	0.031	0.003	4.51E-19	0.040	0.013	2.70E-03

TG	rs76957426	C	0.70	-0.012	0.002	2.29E-08	-0.004	0.008	5.10E-01
TG	rs7704653	A	0.28	-0.016	0.002	4.00E-12	-0.002	0.009	9.90E-01
TG	rs7714361	A	0.77	-0.014	0.002	8.81E-09	-0.006	0.009	5.60E-01
TG	rs7735249	C	0.89	-0.027	0.003	1.96E-17	0.001	0.012	8.30E-01
TG	rs1281978	C	0.47	0.011	0.002	1.02E-08	-0.002	0.008	6.70E-01
TG	rs138191773	G	0.98	0.048	0.008	1.72E-09	0.033	0.031	2.30E-01
TG	rs1835346	A	0.98	0.039	0.007	3.05E-09	0.030	0.025	2.10E-01
TG	rs185139895	G	0.96	-0.043	0.005	1.28E-18	-0.052	0.019	5.40E-03
TG	rs186696265	C	0.99	0.104	0.008	4.84E-36	0.074	0.033	4.00E-02
TG	rs192955957	C	0.90	-0.046	0.004	5.43E-28	0.002	0.016	8.50E-01
TG	rs2277083	A	0.44	0.015	0.002	2.07E-14	0.013	0.008	8.50E-02
TG	rs2499797	G	0.16	0.015	0.003	9.38E-09	0.017	0.011	9.40E-02
TG	rs28383314	T	0.38	-0.038	0.002	4.20E-76	-0.008	0.008	2.80E-01
TG	rs41273040	G	0.98	-0.057	0.007	4.03E-18	0.000	0.026	9.00E-01
TG	rs4134963	C	0.81	0.019	0.003	1.50E-13	0.020	0.010	3.30E-02
TG	rs41552812	C	0.93	0.057	0.005	3.15E-29	0.032	0.020	1.50E-01
TG	rs4709746	C	0.87	0.019	0.003	6.09E-11	0.029	0.011	1.80E-02
TG	rs4714001	G	0.36	-0.012	0.002	4.40E-09	-0.003	0.008	6.20E-01
TG	rs540973884	T	0.40	0.029	0.002	3.06E-45	0.013	0.008	6.60E-02
TG	rs62427982	C	0.68	0.013	0.002	1.14E-09	0.022	0.008	7.00E-03
TG	rs6458869	C	0.36	0.018	0.002	7.10E-18	0.011	0.008	1.60E-01
TG	rs6913325	G	0.55	0.011	0.002	3.23E-08	0.004	0.008	6.40E-01
TG	rs6916318	A	0.47	-0.027	0.002	1.84E-40	-0.029	0.008	4.20E-04
TG	rs6924805	G	0.41	0.011	0.002	3.67E-08	-0.003	0.008	6.80E-01
TG	rs729761	T	0.29	-0.018	0.002	1.12E-15	0.001	0.009	7.60E-01
TG	rs73025562	G	0.75	-0.014	0.002	2.32E-09	0.011	0.009	2.40E-01
TG	rs77009508	A	0.93	-0.045	0.004	4.40E-32	-0.015	0.015	3.10E-01
TG	rs78588343	G	0.82	0.016	0.003	1.70E-09	0.014	0.010	2.10E-01
TG	rs9274390	C	0.86	0.030	0.003	1.40E-18	0.019	0.013	2.10E-01
TG	rs9375694	G	0.30	-0.017	0.002	7.38E-16	-0.020	0.008	2.50E-02
TG	rs9376511	A	0.80	0.015	0.002	9.07E-10	0.005	0.010	7.40E-01
TG	rs9480889	C	0.22	-0.016	0.002	1.30E-11	0.005	0.009	6.20E-01
TG	rs9496567	G	0.76	0.014	0.002	5.27E-09	-0.013	0.009	1.80E-01
TG	rs998584	C	0.52	-0.040	0.002	4.93E-89	-0.018	0.008	2.20E-02
TG	rs10242866	C	0.60	-0.016	0.002	1.76E-14	0.009	0.008	3.00E-01
TG	rs10243434	T	0.41	0.017	0.002	1.36E-15	0.009	0.008	1.80E-01
TG	rs111914893	C	0.95	-0.028	0.005	1.09E-09	-0.026	0.018	1.60E-01
TG	rs11980456	G	0.71	-0.012	0.002	2.64E-08	0.005	0.009	5.50E-01
TG	rs12530679	A	0.52	0.012	0.002	1.13E-09	0.009	0.008	2.30E-01
TG	rs12669911	A	0.39	0.012	0.002	1.17E-08	0.018	0.008	4.20E-02
TG	rs139453187	T	0.93	-0.023	0.004	3.28E-09	-0.029	0.015	1.10E-01
TG	rs1799831	C	0.84	-0.025	0.003	3.44E-19	-0.009	0.011	4.00E-01
TG	rs2240466	G	0.88	0.123	0.003	0.00E+00	-0.016	0.012	1.60E-01
TG	rs2699805	G	0.60	0.020	0.002	2.37E-23	0.009	0.008	2.40E-01
TG	rs41785	C	0.58	0.015	0.002	1.84E-13	-0.004	0.008	5.60E-01
TG	rs4722551	T	0.84	0.037	0.003	1.43E-42	0.016	0.011	8.70E-02
TG	rs4731701	C	0.51	0.033	0.002	3.31E-61	0.012	0.008	7.50E-02
TG	rs498475	G	0.37	0.012	0.002	1.66E-08	0.024	0.008	2.60E-03
TG	rs535241194	A	0.79	-0.021	0.002	2.85E-17	-0.018	0.010	7.90E-02
TG	rs56321085	G	0.92	-0.020	0.004	4.80E-08	-0.012	0.014	3.80E-01
TG	rs62459095	C	0.94	0.032	0.004	7.67E-14	-0.016	0.017	4.90E-01
TG	rs62473520	T	0.92	0.021	0.004	2.90E-08	-0.021	0.015	1.90E-01
TG	rs6465120	A	0.51	0.013	0.002	2.59E-10	0.000	0.008	9.10E-01
TG	rs6968865	A	0.37	-0.015	0.002	8.33E-14	-0.003	0.008	4.30E-01
TG	rs71538127	C	0.88	-0.017	0.003	1.29E-08	-0.004	0.012	8.30E-01
TG	rs72555385	A	0.95	-0.065	0.005	7.88E-45	-0.001	0.018	7.20E-01

TG	rs852388	G	0.79	-0.015	0.002	8.67E-10	-0.027	0.010	2.10E-03
TG	rs113266765	C	0.97	0.033	0.006	1.01E-08	0.022	0.023	2.70E-01
TG	rs114443260	C	0.73	-0.028	0.002	1.08E-36	-0.016	0.009	5.50E-02
TG	rs1149470	T	0.24	0.014	0.002	1.32E-09	0.022	0.009	2.00E-02
TG	rs11781692	C	0.99	-0.075	0.008	2.40E-19	0.033	0.033	4.00E-01
TG	rs13269725	A	0.92	-0.035	0.004	3.95E-21	-0.025	0.014	7.50E-02
TG	rs147011441	G	0.98	-0.042	0.007	1.20E-10	0.000	0.026	9.70E-01
TG	rs1495741	G	0.22	0.038	0.002	6.59E-56	-0.008	0.009	3.70E-01
TG	rs150564454	G	0.99	0.102	0.010	4.61E-26	0.013	0.037	7.00E-01
TG	rs1561928	A	0.12	-0.019	0.003	2.04E-09	-0.006	0.012	6.80E-01
TG	rs2054067	A	0.63	0.011	0.002	4.49E-08	0.002	0.008	6.60E-01
TG	rs2081687	T	0.34	0.026	0.002	5.82E-36	0.015	0.008	5.10E-02
TG	rs308	T	0.98	0.159	0.007	6.48E-115	-0.024	0.028	5.30E-01
TG	rs343	C	0.92	0.141	0.004	0.00E+00	0.034	0.014	2.80E-02
TG	rs34893217	G	0.89	0.018	0.003	2.31E-08	0.006	0.013	6.40E-01
TG	rs35859536	C	0.69	0.014	0.002	2.62E-11	-0.005	0.008	7.20E-01
TG	rs36061954	C	0.60	-0.012	0.002	3.66E-09	-0.003	0.008	8.60E-01
TG	rs3808477	C	0.72	0.013	0.002	2.20E-09	0.002	0.009	6.70E-01
TG	rs383091	T	0.37	-0.013	0.002	5.43E-10	-0.005	0.008	4.90E-01
TG	rs4500049	A	0.47	0.028	0.002	2.66E-44	-0.001	0.008	8.80E-01
TG	rs6999569	A	0.53	0.086	0.002	0.00E+00	0.036	0.008	2.80E-06
TG	rs7000494	G	0.97	-0.136	0.006	4.11E-120	-0.031	0.023	1.70E-01
TG	rs7018436	T	0.69	-0.015	0.002	2.73E-12	-0.008	0.008	3.50E-01
TG	rs72691637	G	0.81	0.015	0.003	2.91E-09	0.003	0.010	8.60E-01
TG	rs75609851	G	0.99	0.199	0.010	4.79E-87	-0.009	0.040	7.40E-01
TG	rs7826246	A	0.90	-0.020	0.004	4.20E-08	0.019	0.014	1.60E-01
TG	rs78376313	T	0.97	-0.048	0.006	4.45E-17	-0.008	0.023	5.50E-01
TG	rs79153732	C	0.98	-0.077	0.008	4.00E-24	0.080	0.030	4.00E-03
TG	rs10797119	T	0.46	-0.016	0.002	5.43E-15	-0.009	0.008	2.10E-01
TG	rs10811662	G	0.83	0.015	0.003	5.91E-09	-0.003	0.010	7.20E-01
TG	rs10962680	C	0.26	0.014	0.002	6.68E-09	0.002	0.009	7.20E-01
TG	rs1567353	C	0.69	-0.015	0.002	3.38E-12	-0.004	0.008	7.10E-01
TG	rs1800978	C	0.88	0.027	0.003	1.08E-18	-0.023	0.012	7.10E-02
TG	rs2131919	A	0.84	-0.017	0.003	1.34E-10	-0.011	0.010	2.70E-01
TG	rs2416759	G	0.30	-0.012	0.002	2.91E-08	-0.012	0.008	1.40E-01
TG	rs2519093	C	0.82	0.021	0.003	1.29E-16	-0.022	0.010	2.40E-02
TG	rs4382584	G	0.73	-0.013	0.002	5.71E-09	0.013	0.009	1.50E-01
TG	rs4564007	T	0.32	0.012	0.002	1.07E-08	0.015	0.008	3.90E-02
TG	rs581080	G	0.18	-0.018	0.003	1.32E-11	-0.012	0.010	1.90E-01
TG	rs62565259	C	0.83	0.017	0.003	1.58E-10	0.011	0.010	2.50E-01
TG	rs696825	C	0.75	0.020	0.002	5.72E-19	0.020	0.009	1.70E-02
TG	rs77824033	T	0.96	0.030	0.005	1.34E-08	-0.014	0.021	4.30E-01
TG	rs7855395	A	0.43	0.012	0.002	6.47E-09	0.002	0.008	7.50E-01
TG	rs10822163	C	0.53	0.032	0.002	2.30E-57	-0.003	0.008	8.50E-01
TG	rs11187027	G	0.79	-0.015	0.002	3.38E-10	-0.020	0.009	3.00E-02
TG	rs1133400	A	0.78	-0.014	0.002	1.02E-08	0.003	0.009	7.00E-01
TG	rs113344423	G	0.94	-0.043	0.004	1.60E-23	-0.016	0.016	4.50E-01
TG	rs1171617	G	0.23	-0.016	0.002	2.39E-11	0.005	0.009	7.00E-01
TG	rs12415159	A	0.85	-0.021	0.003	1.28E-13	0.030	0.011	5.30E-03
TG	rs140107293	A	0.85	0.023	0.003	2.75E-16	0.029	0.011	3.70E-03
TG	rs2068888	G	0.55	0.032	0.002	4.06E-57	0.013	0.008	8.30E-02
TG	rs2420477	T	0.47	0.011	0.002	2.62E-08	0.020	0.008	9.70E-03
TG	rs2487294	G	0.28	-0.018	0.002	1.53E-16	0.053	0.009	4.10E-09
TG	rs2773469	A	0.27	0.019	0.002	1.59E-16	0.004	0.009	7.40E-01
TG	rs3758413	T	0.58	-0.011	0.002	1.76E-08	-0.014	0.008	9.90E-02
TG	rs3829126	G	0.91	-0.023	0.003	2.20E-11	-0.003	0.013	7.50E-01

TG	rs55767272	A	0.93	0.028	0.004	5.97E-12	0.041	0.016	1.30E-02
TG	rs563296	G	0.44	-0.016	0.002	5.55E-16	-0.011	0.008	9.70E-02
TG	rs7077812	T	0.81	-0.014	0.003	2.20E-08	-0.006	0.010	3.80E-01
TG	rs71473777	A	0.88	-0.019	0.003	8.18E-10	-0.027	0.012	1.90E-02
TG	rs75398587	C	0.93	0.025	0.004	1.10E-10	-0.019	0.016	2.30E-01
TG	rs80276949	G	0.98	-0.045	0.007	1.16E-11	-0.039	0.025	1.30E-01
TG	rs878409	G	0.46	0.011	0.002	2.07E-08	0.002	0.008	7.00E-01
TG	rs973709	G	0.44	0.011	0.002	4.52E-08	-0.009	0.008	2.80E-01
TG	rs1064939	A	0.98	0.054	0.007	1.63E-15	0.076	0.026	2.60E-03
TG	rs10750766	C	0.29	-0.020	0.002	3.94E-19	-0.009	0.009	3.00E-01
TG	rs10899490	C	0.84	0.017	0.003	2.40E-10	-0.016	0.011	2.00E-01
TG	rs11030107	A	0.74	-0.016	0.002	8.55E-13	-0.019	0.009	4.80E-02
TG	rs11228377	T	0.41	0.015	0.002	2.50E-14	-0.001	0.008	8.00E-01
TG	rs11231161	A	0.63	-0.017	0.002	9.20E-16	-0.005	0.008	4.30E-01
TG	rs117291242	C	0.96	-0.030	0.005	1.38E-08	0.019	0.020	4.50E-01
TG	rs150555490	C	0.94	0.039	0.004	1.48E-19	-0.008	0.016	7.00E-01
TG	rs174566	A	0.65	-0.049	0.002	2.13E-120	0.000	0.008	1.00E+00
TG	rs2850245	G	0.37	0.017	0.002	9.24E-16	0.006	0.008	5.20E-01
TG	rs326222	T	0.30	-0.025	0.002	1.69E-31	-0.012	0.008	1.30E-01
TG	rs480823	T	0.92	-0.156	0.004	0.00E+00	-0.014	0.014	3.70E-01
TG	rs490972	G	0.53	-0.014	0.002	1.06E-12	-0.012	0.008	1.10E-01
TG	rs4909945	T	0.31	-0.013	0.002	1.24E-09	-0.009	0.008	2.60E-01
TG	rs499293	G	0.34	0.012	0.002	1.71E-08	0.017	0.008	2.10E-02
TG	rs56271783	G	0.95	-0.067	0.005	7.90E-44	-0.020	0.019	3.40E-01
TG	rs61885960	T	0.95	0.032	0.004	8.02E-13	0.039	0.017	1.40E-02
TG	rs61905078	A	0.93	-0.200	0.004	0.00E+00	-0.005	0.015	7.30E-01
TG	rs6486122	C	0.31	-0.020	0.002	2.98E-20	-0.015	0.008	8.60E-02
TG	rs75268115	A	0.92	0.020	0.004	1.23E-08	0.011	0.014	3.80E-01
TG	rs78297458	T	0.98	0.039	0.007	1.34E-08	0.039	0.026	1.30E-01
TG	rs78484485	G	0.95	0.076	0.004	6.37E-67	0.017	0.017	2.50E-01
TG	rs79357714	A	0.95	0.029	0.005	1.58E-09	-0.005	0.019	8.30E-01
TG	rs79634051	G	0.97	0.043	0.006	1.42E-12	0.011	0.023	6.10E-01
TG	rs10842703	A	0.76	-0.015	0.002	2.05E-10	-0.012	0.009	1.30E-01
TG	rs112403212	C	0.86	-0.017	0.003	1.37E-09	0.003	0.011	8.30E-01
TG	rs113439801	C	0.83	0.017	0.003	7.20E-11	-0.003	0.010	7.80E-01
TG	rs12422600	G	0.63	0.013	0.002	1.45E-09	0.007	0.008	3.40E-01
TG	rs1351394	T	0.49	-0.013	0.002	5.02E-11	-0.022	0.008	6.30E-03
TG	rs139386986	C	0.91	0.022	0.004	1.62E-09	0.009	0.014	5.30E-01
TG	rs35104374	T	0.27	0.016	0.002	3.72E-12	0.004	0.009	5.80E-01
TG	rs35763453	T	0.94	-0.028	0.004	9.10E-11	-0.015	0.017	2.80E-01
TG	rs35764600	G	0.60	-0.013	0.002	1.74E-10	0.008	0.008	3.50E-01
TG	rs4760254	G	0.76	0.028	0.002	2.90E-33	0.006	0.009	4.90E-01
TG	rs4761234	T	0.52	0.014	0.002	4.07E-12	-0.007	0.008	3.60E-01
TG	rs4930724	T	0.67	0.026	0.002	3.66E-35	0.023	0.008	6.80E-03
TG	rs580063	T	0.79	0.022	0.002	2.89E-19	0.010	0.010	3.50E-01
TG	rs67981690	A	0.87	-0.030	0.003	1.99E-23	-0.005	0.012	5.30E-01
TG	rs7134375	C	0.57	0.017	0.002	1.18E-17	0.018	0.008	1.70E-02
TG	rs7136223	A	0.72	0.013	0.002	1.81E-08	0.006	0.009	5.60E-01
TG	rs7138037	G	0.77	-0.019	0.002	4.93E-16	0.002	0.009	9.40E-01
TG	rs76895963	T	0.98	0.087	0.008	8.11E-30	0.112	0.030	1.70E-04
TG	rs775633	T	0.35	0.013	0.002	2.80E-09	0.015	0.008	7.40E-02
TG	rs863750	C	0.40	-0.029	0.002	2.04E-45	-0.014	0.008	6.60E-02
TG	rs9788220	T	0.19	-0.016	0.003	9.78E-10	0.028	0.010	4.60E-03
TG	rs9943778	A	0.76	0.015	0.002	7.91E-11	0.001	0.009	7.90E-01
TG	rs1340819	A	0.65	0.012	0.002	4.35E-09	-0.011	0.008	2.60E-01
TG	rs149778057	A	0.67	0.016	0.002	2.54E-12	0.019	0.009	1.10E-02

TG	rs1556124	G	0.23	-0.013	0.002	2.41E-08	-0.014	0.009	4.90E-02
TG	rs2774430	A	0.44	-0.011	0.002	3.53E-08	-0.011	0.008	1.60E-01
TG	rs2812208	G	0.98	0.049	0.007	2.40E-12	0.035	0.026	1.30E-01
TG	rs7140110	T	0.70	-0.028	0.002	5.67E-38	-0.005	0.008	4.40E-01
TG	rs7400002	A	0.77	-0.014	0.002	6.07E-09	0.003	0.009	6.10E-01
TG	rs79192570	G	0.86	0.025	0.003	1.02E-18	0.003	0.011	8.10E-01
TG	rs9553567	T	0.16	-0.015	0.003	2.79E-08	-0.001	0.011	8.70E-01
TG	rs9561643	A	0.69	-0.017	0.002	1.48E-14	0.023	0.008	3.60E-03
TG	rs9584870	T	0.63	0.012	0.002	7.81E-09	0.019	0.008	2.20E-02
TG	rs9600143	A	0.46	0.013	0.002	5.77E-10	-0.008	0.008	3.20E-01
TG	rs12880341	T	0.84	-0.021	0.003	2.95E-14	0.004	0.011	7.00E-01
TG	rs2070341	C	0.40	-0.011	0.002	3.77E-08	-0.010	0.008	2.10E-01
TG	rs2240533	T	0.69	0.013	0.002	2.68E-09	0.020	0.008	9.00E-03
TG	rs56902258	T	0.80	0.015	0.003	2.08E-09	0.012	0.010	3.20E-01
TG	rs61975915	C	0.70	0.012	0.002	3.77E-08	-0.003	0.009	7.90E-01
TG	rs61993685	T	0.92	0.023	0.004	3.63E-10	0.020	0.014	1.50E-01
TG	rs6572807	A	0.73	-0.013	0.002	2.85E-08	-0.005	0.009	4.20E-01
TG	rs10152471	G	0.61	0.013	0.002	4.92E-11	-0.010	0.008	1.90E-01
TG	rs1077835	A	0.78	-0.047	0.002	3.42E-86	0.009	0.009	3.30E-01
TG	rs11635675	T	0.66	-0.024	0.002	4.19E-31	0.015	0.008	7.40E-02
TG	rs12440800	A	0.74	-0.016	0.002	1.96E-12	-0.009	0.009	4.10E-01
TG	rs12591786	C	0.84	0.018	0.003	1.65E-10	0.002	0.011	9.90E-01
TG	rs138751626	A	0.92	-0.028	0.004	3.86E-14	-0.021	0.015	1.40E-01
TG	rs139974673	T	0.97	-0.143	0.006	2.21E-115	-0.037	0.025	1.40E-01
TG	rs1532085	A	0.39	0.031	0.002	3.18E-51	-0.013	0.008	8.90E-02
TG	rs2017500	G	0.49	-0.011	0.002	1.85E-08	-0.003	0.008	8.70E-01
TG	rs28624578	T	0.83	-0.015	0.003	1.91E-08	-0.010	0.010	2.40E-01
TG	rs34245505	C	0.80	-0.016	0.003	4.71E-10	-0.003	0.010	9.10E-01
TG	rs3784310	T	0.72	0.013	0.002	4.26E-09	-0.001	0.009	9.60E-01
TG	rs3826043	C	0.57	0.012	0.002	1.75E-09	0.020	0.008	4.80E-03
TG	rs8025505	C	0.74	-0.017	0.002	4.02E-14	0.002	0.009	7.80E-01
TG	rs9944241	T	0.52	0.012	0.002	2.23E-09	0.000	0.008	8.80E-01
TG	rs12446515	C	0.68	0.033	0.002	3.77E-55	-0.005	0.008	5.40E-01
TG	rs12600110	T	0.62	0.015	0.002	9.29E-13	-0.011	0.008	1.20E-01
TG	rs12926107	A	0.55	-0.013	0.002	1.38E-10	-0.009	0.008	2.00E-01
TG	rs12928099	C	0.70	0.028	0.002	9.63E-38	0.015	0.008	9.80E-02
TG	rs143076454	G	0.98	-0.040	0.007	4.26E-08	-0.007	0.028	8.50E-01
TG	rs1728407	A	0.55	-0.012	0.002	1.01E-08	-0.002	0.008	7.90E-01
TG	rs200293726	A	0.69	-0.013	0.002	3.11E-09	-0.013	0.008	1.00E-01
TG	rs2288004	G	0.62	0.015	0.002	7.35E-13	0.001	0.008	8.50E-01
TG	rs28577186	G	0.34	0.016	0.002	4.10E-14	0.017	0.008	4.80E-02
TG	rs2917677	C	0.59	0.019	0.002	2.61E-20	0.014	0.008	7.40E-02
TG	rs2925979	T	0.30	0.032	0.002	1.42E-49	0.019	0.008	2.20E-02
TG	rs3794695	C	0.81	-0.028	0.003	1.13E-27	-0.009	0.010	4.90E-01
TG	rs3814883	C	0.52	-0.015	0.002	1.38E-13	-0.001	0.008	8.40E-01
TG	rs62064941	A	0.96	0.029	0.005	4.20E-08	0.033	0.020	7.40E-02
TG	rs7186635	A	0.68	-0.012	0.002	3.47E-08	-0.001	0.008	6.80E-01
TG	rs7191623	G	0.79	0.015	0.002	4.61E-10	0.005	0.010	5.10E-01
TG	rs7199293	G	0.47	-0.011	0.002	2.02E-08	-0.007	0.008	3.20E-01
TG	rs79311290	A	0.89	-0.022	0.003	7.02E-11	-0.010	0.013	4.50E-01
TG	rs933574	A	0.52	-0.012	0.002	1.72E-09	0.014	0.008	4.20E-02
TG	rs10775406	A	0.24	-0.020	0.002	1.62E-18	-0.008	0.009	3.90E-01
TG	rs11078597	T	0.81	-0.019	0.003	1.01E-13	0.015	0.010	1.10E-01
TG	rs11078696	G	0.20	0.017	0.003	3.35E-11	0.029	0.010	6.80E-03
TG	rs112162280	C	0.71	0.012	0.002	2.25E-08	0.015	0.009	6.20E-02
TG	rs11657201	A	0.76	-0.013	0.002	2.40E-08	0.001	0.009	8.70E-01

TG	rs116878033	C	0.96	-0.043	0.005	1.12E-15	-0.014	0.021	3.90E-01
TG	rs12185242	A	0.55	-0.017	0.002	4.03E-18	-0.005	0.008	6.50E-01
TG	rs1292065	C	0.29	0.014	0.002	2.99E-10	-0.003	0.009	9.20E-01
TG	rs1801689	A	0.97	0.066	0.006	2.33E-30	-0.109	0.023	9.40E-07
TG	rs4559942	G	0.23	-0.014	0.002	1.19E-08	-0.024	0.009	1.30E-02
TG	rs4969179	T	0.40	0.018	0.002	2.73E-18	0.020	0.008	7.40E-03
TG	rs595767	A	0.48	-0.014	0.002	1.09E-11	-0.003	0.008	5.90E-01
TG	rs62084237	G	0.82	-0.024	0.003	2.91E-20	-0.006	0.010	6.10E-01
TG	rs7215055	A	0.94	-0.039	0.004	1.50E-21	0.010	0.016	5.20E-01
TG	rs72836561	C	0.97	-0.137	0.006	2.03E-126	-0.012	0.022	6.10E-01
TG	rs77244849	T	0.68	0.015	0.002	1.45E-11	0.018	0.008	4.40E-02
TG	rs8066985	A	0.48	0.013	0.002	1.73E-10	-0.003	0.008	5.70E-01
TG	rs9890200	A	0.63	0.013	0.002	1.56E-10	0.021	0.008	8.10E-03
TG	rs11664106	A	0.63	0.013	0.002	2.17E-09	0.014	0.008	6.90E-02
TG	rs12454712	T	0.62	0.012	0.002	1.55E-09	0.016	0.008	5.70E-02
TG	rs2187114	G	0.90	0.019	0.003	2.22E-08	0.003	0.013	7.80E-01
TG	rs41292412	C	0.99	-0.063	0.009	1.41E-11	0.003	0.035	7.20E-01
TG	rs6506033	C	0.93	0.023	0.004	1.20E-09	0.014	0.015	2.30E-01
TG	rs7239575	T	0.51	0.016	0.002	4.31E-15	0.007	0.008	4.70E-01
TG	rs867939	G	0.42	0.014	0.002	8.75E-12	0.009	0.008	2.40E-01
TG	rs921971	T	0.73	-0.016	0.002	2.79E-12	-0.001	0.009	7.50E-01
TG	rs10422861	C	0.33	0.019	0.002	3.23E-20	0.010	0.008	2.30E-01
TG	rs116843064	G	0.98	0.226	0.007	3.11E-215	-0.005	0.028	8.80E-01
TG	rs12610709	G	0.83	-0.022	0.003	4.98E-17	-0.004	0.010	7.60E-01
TG	rs142385484	C	0.85	0.023	0.003	3.15E-16	0.022	0.011	3.60E-02
TG	rs188247550	C	0.99	0.134	0.009	1.66E-48	-0.252	0.034	3.20E-14
TG	rs2278426	C	0.96	0.041	0.005	1.98E-14	-0.020	0.021	4.00E-01
TG	rs2860183	T	0.38	0.014	0.002	9.19E-12	0.016	0.008	5.50E-02
TG	rs296360	T	0.83	0.018	0.003	5.37E-11	0.000	0.011	8.80E-01
TG	rs3890483	G	0.56	-0.020	0.002	2.09E-22	-0.022	0.008	8.20E-04
TG	rs483082	G	0.76	-0.086	0.002	1.11E-292	0.078	0.009	2.80E-17
TG	rs5112	C	0.47	-0.068	0.002	6.50E-224	0.028	0.008	8.10E-04
TG	rs55737395	G	0.66	0.013	0.002	3.28E-10	0.007	0.008	4.50E-01
TG	rs573377651	A	0.92	-0.023	0.004	2.47E-10	0.002	0.014	9.10E-01
TG	rs58324296	A	0.65	0.015	0.002	8.67E-13	0.019	0.008	2.10E-02
TG	rs58542926	C	0.93	0.103	0.004	1.20E-162	-0.289	0.015	2.80E-85
TG	rs58895965	C	0.83	-0.027	0.003	2.17E-24	0.018	0.010	6.30E-02
TG	rs62118471	T	0.97	-0.043	0.006	5.05E-11	-0.003	0.026	8.70E-01
TG	rs7260465	C	0.74	0.016	0.002	1.88E-12	-0.016	0.009	6.50E-02
TG	rs8102873	C	0.42	-0.012	0.002	8.84E-10	0.003	0.008	7.70E-01
TG	rs838133	A	0.45	0.025	0.002	1.81E-33	0.024	0.008	2.20E-03
TG	rs149142833	C	0.84	-0.017	0.003	3.22E-10	-0.046	0.011	2.40E-05
TG	rs151235402	C	0.98	-0.053	0.008	8.80E-11	-0.090	0.031	3.60E-03
TG	rs1883711	G	0.97	-0.060	0.006	1.06E-24	-0.068	0.023	1.80E-03
TG	rs293561	T	0.64	-0.012	0.002	2.34E-08	0.006	0.008	4.70E-01
TG	rs55837381	G	0.75	0.013	0.002	9.67E-09	0.001	0.009	9.50E-01
TG	rs55966194	C	0.72	0.018	0.002	1.48E-15	-0.002	0.009	7.80E-01
TG	rs6068280	A	0.33	-0.014	0.002	6.18E-11	-0.023	0.008	3.20E-03
TG	rs6073958	T	0.80	-0.056	0.002	1.86E-109	-0.001	0.010	9.50E-01
TG	rs6093446	G	0.71	-0.015	0.002	2.24E-11	-0.003	0.009	7.50E-01
TG	rs7274718	G	0.40	-0.016	0.002	6.24E-15	-0.002	0.008	9.70E-01
TG	rs8126001	C	0.51	0.016	0.002	3.48E-16	0.008	0.008	2.30E-01
TG	rs394872	C	0.46	-0.011	0.002	1.90E-08	-0.013	0.008	9.20E-02
TG	rs6517522	T	0.50	0.013	0.002	9.83E-11	0.007	0.008	3.60E-01
TG	rs134551	C	0.66	0.012	0.002	3.01E-08	-0.003	0.008	8.30E-01
TG	rs140288	G	0.43	0.013	0.002	3.69E-11	-0.014	0.008	8.10E-02

TG	rs2071887	T	0.66	-0.016	0.002	2.41E-14	-0.006	0.008	4.60E-01
TG	rs2267373	C	0.42	-0.022	0.002	1.89E-26	-0.001	0.008	7.80E-01
TG	rs60610697	T	0.78	-0.018	0.002	2.05E-13	-0.002	0.009	6.50E-01
TG	rs9610329	C	0.57	-0.014	0.002	1.76E-11	-0.025	0.008	1.30E-03
WHR	rs10158345	C	0.70	0.012	0.002	7.37E-10	-0.001	0.009	9.40E-01
WHR	rs10919388	A	0.27	-0.027	0.002	1.56E-44	0.000	0.009	9.30E-01
WHR	rs10923724	T	0.56	0.024	0.002	2.96E-45	0.000	0.008	9.70E-01
WHR	rs11162968	T	0.68	-0.011	0.002	9.65E-09	-0.011	0.008	1.60E-01
WHR	rs11208660	T	0.07	0.023	0.003	1.77E-14	0.004	0.014	8.50E-01
WHR	rs11577194	T	0.50	0.009	0.002	3.21E-08	0.017	0.008	4.60E-02
WHR	rs12024554	T	0.23	-0.015	0.002	9.28E-11	-0.004	0.009	6.70E-01
WHR	rs12042959	A	0.86	0.016	0.003	5.65E-10	0.005	0.011	7.60E-01
WHR	rs12048743	C	0.55	-0.013	0.002	5.11E-13	-0.007	0.008	3.30E-01
WHR	rs12140153	T	0.09	-0.021	0.004	9.70E-10	-0.006	0.013	6.10E-01
WHR	rs1289021	C	0.43	0.011	0.002	2.40E-10	0.010	0.008	2.70E-01
WHR	rs1563355	T	0.33	-0.028	0.002	3.82E-48	-0.019	0.008	3.30E-02
WHR	rs17015701	A	0.21	0.012	0.002	6.27E-09	0.005	0.010	4.80E-01
WHR	rs17185933	A	0.17	0.014	0.002	5.93E-11	0.007	0.010	5.20E-01
WHR	rs1884454	T	0.27	-0.011	0.002	5.68E-08	0.004	0.009	7.10E-01
WHR	rs1891216	T	0.63	-0.010	0.002	7.57E-09	-0.002	0.008	7.70E-01
WHR	rs2061708	C	0.59	0.015	0.002	2.96E-14	-0.012	0.008	1.30E-01
WHR	rs213020	A	0.31	-0.012	0.002	4.71E-09	-0.009	0.008	3.10E-01
WHR	rs213637	T	0.53	0.013	0.002	3.80E-12	0.009	0.008	2.30E-01
WHR	rs2235529	T	0.16	-0.014	0.002	1.14E-08	-0.007	0.011	6.00E-01
WHR	rs2298632	T	0.49	-0.014	0.002	2.44E-17	-0.014	0.008	2.70E-02
WHR	rs2301453	A	0.55	-0.022	0.002	1.20E-39	-0.018	0.008	1.40E-02
WHR	rs2335077	A	0.64	-0.012	0.002	1.91E-10	-0.013	0.008	1.40E-01
WHR	rs2742690	A	0.20	0.017	0.003	1.82E-11	-0.012	0.010	2.50E-01
WHR	rs2791556	T	0.71	-0.014	0.002	8.19E-13	-0.017	0.009	6.30E-02
WHR	rs2815749	A	0.18	-0.017	0.002	2.68E-15	-0.005	0.010	6.50E-01
WHR	rs28680958	A	0.22	-0.014	0.002	4.37E-09	-0.001	0.009	8.40E-01
WHR	rs3767848	A	0.25	-0.012	0.002	3.53E-09	-0.022	0.009	1.60E-02
WHR	rs3789615	T	0.43	-0.013	0.002	5.14E-15	-0.023	0.008	3.30E-03
WHR	rs380654	C	0.60	-0.012	0.002	1.22E-11	-0.011	0.008	1.80E-01
WHR	rs4660808	T	0.22	0.015	0.002	5.03E-12	0.004	0.009	6.80E-01
WHR	rs543874	A	0.78	-0.020	0.002	4.18E-22	-0.002	0.010	7.90E-01
WHR	rs587271	T	0.69	0.011	0.002	2.42E-08	0.019	0.009	3.60E-02
WHR	rs61783470	A	0.13	-0.019	0.003	2.40E-10	-0.020	0.012	1.50E-01
WHR	rs61813324	T	0.13	0.019	0.003	7.16E-11	0.007	0.012	6.30E-01
WHR	rs6658424	A	0.28	-0.013	0.002	4.74E-11	-0.008	0.009	4.50E-01
WHR	rs6658723	T	0.43	0.015	0.002	4.17E-13	-0.005	0.008	3.80E-01
WHR	rs6688233	T	0.24	0.019	0.002	8.21E-19	0.022	0.009	1.80E-02
WHR	rs6699397	A	0.62	-0.012	0.002	3.89E-12	-0.008	0.008	3.50E-01
WHR	rs7528123	T	0.62	-0.010	0.002	1.05E-08	0.000	0.008	9.00E-01
WHR	rs7531656	A	0.33	0.016	0.002	1.36E-19	0.014	0.008	8.30E-02
WHR	rs754984	C	0.93	-0.020	0.004	1.54E-08	-0.003	0.015	8.40E-01
WHR	rs785505	A	0.95	0.024	0.004	1.10E-08	0.039	0.018	2.10E-02
WHR	rs905938	T	0.72	0.013	0.002	3.32E-11	0.014	0.009	6.10E-02
WHR	rs9659380	A	0.84	-0.018	0.002	8.76E-14	-0.016	0.011	8.50E-02
WHR	rs10153926	A	0.22	0.014	0.002	2.55E-09	0.008	0.010	4.30E-01
WHR	rs1017698	A	0.60	-0.011	0.002	3.43E-10	-0.025	0.008	1.30E-03
WHR	rs10185087	T	0.59	-0.010	0.002	1.05E-08	-0.010	0.008	1.60E-01
WHR	rs10195252	T	0.58	0.023	0.002	5.16E-41	0.036	0.008	7.40E-06
WHR	rs1020731	A	0.70	0.013	0.002	2.32E-11	0.009	0.008	2.20E-01
WHR	rs10495563	A	0.66	0.012	0.002	8.03E-11	-0.006	0.008	4.80E-01
WHR	rs1124639	T	0.44	-0.011	0.002	2.99E-11	-0.008	0.008	2.90E-01

WHR	rs11677670	T	0.18	-0.018	0.002	2.77E-14	-0.014	0.010	1.60E-01
WHR	rs11688816	A	0.48	-0.009	0.002	4.49E-08	-0.007	0.008	4.00E-01
WHR	rs11897119	T	0.59	-0.013	0.002	3.21E-15	0.000	0.008	8.20E-01
WHR	rs12466434	T	0.55	0.010	0.002	1.05E-08	0.000	0.008	9.30E-01
WHR	rs12469667	A	0.23	-0.013	0.002	6.00E-10	-0.005	0.009	6.50E-01
WHR	rs13028903	T	0.46	0.011	0.002	3.43E-10	-0.006	0.008	4.30E-01
WHR	rs13391573	T	0.18	0.021	0.002	1.35E-20	0.014	0.010	1.20E-01
WHR	rs1345203	T	0.78	0.019	0.002	6.61E-15	0.015	0.010	1.40E-01
WHR	rs1569135	A	0.54	0.022	0.002	1.16E-36	0.011	0.008	1.70E-01
WHR	rs17041868	T	0.92	-0.019	0.003	1.94E-08	-0.032	0.016	4.00E-02
WHR	rs17324331	C	0.31	0.012	0.002	2.69E-10	0.006	0.008	4.30E-01
WHR	rs17326656	T	0.23	0.015	0.002	3.20E-13	0.022	0.009	1.40E-02
WHR	rs17489259	A	0.23	-0.012	0.002	1.10E-08	-0.006	0.009	4.70E-01
WHR	rs2195086	T	0.83	-0.014	0.002	1.97E-09	-0.007	0.011	4.50E-01
WHR	rs2373078	T	0.10	0.016	0.003	5.09E-08	-0.004	0.013	7.20E-01
WHR	rs2384463	A	0.48	0.011	0.002	2.77E-09	0.029	0.008	4.10E-04
WHR	rs2709373	T	0.82	0.013	0.002	4.30E-08	-0.007	0.010	5.70E-01
WHR	rs332105	A	0.55	-0.014	0.002	1.14E-14	-0.006	0.008	3.80E-01
WHR	rs3891424	A	0.05	-0.028	0.004	2.65E-10	0.007	0.017	6.70E-01
WHR	rs399984	C	0.78	0.013	0.002	4.53E-09	-0.001	0.009	9.40E-01
WHR	rs4366921	C	0.30	-0.012	0.002	8.32E-09	0.000	0.008	9.90E-01
WHR	rs4668304	A	0.36	0.011	0.002	3.27E-08	0.020	0.008	1.30E-02
WHR	rs4671358	A	0.60	0.011	0.002	5.43E-09	0.017	0.008	2.40E-02
WHR	rs4671796	A	0.55	0.011	0.002	6.97E-10	-0.003	0.008	9.20E-01
WHR	rs4673616	T	0.64	-0.012	0.002	1.45E-09	-0.017	0.009	3.70E-02
WHR	rs4849294	T	0.61	0.012	0.002	5.62E-12	-0.007	0.008	2.80E-01
WHR	rs4851057	T	0.13	0.016	0.003	4.86E-09	0.031	0.012	8.90E-03
WHR	rs4851283	C	0.31	0.016	0.002	5.74E-15	0.018	0.008	1.40E-02
WHR	rs55920843	T	0.99	0.062	0.009	1.27E-11	0.046	0.036	2.40E-01
WHR	rs62106258	T	0.95	0.043	0.005	3.92E-21	0.043	0.018	2.00E-02
WHR	rs6430168	T	0.21	0.012	0.002	2.26E-08	0.003	0.010	8.70E-01
WHR	rs6433219	A	0.27	0.014	0.002	1.05E-11	0.005	0.009	7.10E-01
WHR	rs6545714	A	0.61	-0.014	0.002	2.92E-16	0.009	0.008	3.30E-01
WHR	rs6743060	A	0.84	0.027	0.002	1.18E-33	-0.004	0.010	7.20E-01
WHR	rs6749646	A	0.78	-0.021	0.002	1.36E-21	0.000	0.009	9.00E-01
WHR	rs711869	A	0.56	-0.017	0.002	2.10E-21	-0.001	0.008	9.60E-01
WHR	rs7561798	A	0.53	-0.010	0.002	1.16E-08	-0.023	0.008	3.60E-03
WHR	rs7587522	C	0.35	-0.011	0.002	2.77E-09	0.001	0.008	9.00E-01
WHR	rs7591387	T	0.10	0.018	0.003	1.63E-10	0.002	0.012	9.00E-01
WHR	rs7598832	T	0.33	-0.019	0.002	8.64E-26	0.008	0.008	3.70E-01
WHR	rs7599312	A	0.27	-0.011	0.002	1.97E-09	0.003	0.009	6.10E-01
WHR	rs843460	A	0.67	-0.012	0.002	1.46E-08	-0.011	0.008	2.00E-01
WHR	rs929641	A	0.59	0.013	0.002	7.98E-14	0.011	0.008	1.60E-01
WHR	rs930653	A	0.36	0.011	0.002	1.31E-08	0.007	0.008	4.10E-01
WHR	rs9630986	C	0.67	0.012	0.002	1.35E-10	0.021	0.008	5.00E-03
WHR	rs10049088	T	0.38	-0.027	0.002	3.17E-51	0.008	0.008	2.70E-01
WHR	rs10490869	A	0.80	-0.018	0.002	8.93E-17	-0.018	0.010	1.00E-01
WHR	rs112551697	A	0.03	0.032	0.006	2.19E-08	0.049	0.022	3.60E-02
WHR	rs115934382	A	0.92	0.023	0.004	2.00E-10	0.015	0.014	3.40E-01
WHR	rs11705729	A	0.68	-0.011	0.002	2.42E-08	-0.002	0.008	6.90E-01
WHR	rs11718898	T	0.32	0.014	0.002	5.57E-13	0.020	0.008	1.50E-02
WHR	rs11917047	A	0.76	-0.012	0.002	2.53E-08	-0.006	0.009	4.60E-01
WHR	rs12495178	T	0.64	0.012	0.002	5.62E-12	0.014	0.008	8.40E-02
WHR	rs12631066	C	0.22	0.012	0.002	3.53E-09	0.006	0.009	5.60E-01
WHR	rs1286769	A	0.46	-0.010	0.002	2.29E-08	-0.005	0.008	5.40E-01
WHR	rs13063979	T	0.73	-0.014	0.002	1.20E-12	-0.014	0.009	1.10E-01

WHR	rs13080520	T	0.64	-0.011	0.002	2.77E-09	-0.016	0.008	5.80E-02
WHR	rs13095863	T	0.33	-0.011	0.002	1.79E-08	-0.028	0.008	6.20E-04
WHR	rs13316065	T	0.32	0.017	0.002	3.57E-21	0.029	0.008	2.20E-04
WHR	rs1452075	T	0.73	0.012	0.002	9.56E-11	0.026	0.009	3.20E-03
WHR	rs155524	A	0.59	0.013	0.002	9.74E-14	0.006	0.008	5.30E-01
WHR	rs1672936	A	0.46	0.012	0.002	6.63E-09	0.006	0.008	3.70E-01
WHR	rs16853606	A	0.84	-0.013	0.002	9.52E-09	0.001	0.011	9.50E-01
WHR	rs17289049	A	0.91	-0.016	0.003	4.59E-08	-0.005	0.014	7.40E-01
WHR	rs2242116	A	0.38	-0.013	0.002	1.72E-12	-0.018	0.008	2.80E-02
WHR	rs2371767	C	0.27	-0.029	0.002	6.00E-53	-0.009	0.009	3.30E-01
WHR	rs2455848	T	0.31	0.013	0.002	1.15E-12	0.017	0.008	4.80E-02
WHR	rs2590440	A	0.78	-0.014	0.002	4.75E-10	-0.024	0.010	1.70E-02
WHR	rs2606737	A	0.18	0.015	0.002	5.20E-11	0.014	0.010	1.80E-01
WHR	rs310761	A	0.10	0.020	0.003	9.29E-10	0.007	0.013	4.50E-01
WHR	rs4320054	A	0.59	-0.013	0.002	2.93E-11	-0.012	0.008	1.40E-01
WHR	rs4635727	A	0.72	0.012	0.002	3.64E-09	0.012	0.009	2.70E-01
WHR	rs4686340	A	0.25	0.012	0.002	7.75E-10	0.006	0.009	5.10E-01
WHR	rs4894803	A	0.60	0.015	0.002	1.25E-16	-0.007	0.008	4.60E-01
WHR	rs56133301	A	0.01	-0.057	0.009	1.57E-11	-0.013	0.034	8.10E-01
WHR	rs56853116	A	0.71	-0.013	0.002	1.33E-08	-0.007	0.009	3.20E-01
WHR	rs6442644	T	0.70	-0.011	0.002	3.75E-09	0.007	0.008	3.00E-01
WHR	rs645040	T	0.77	0.016	0.002	6.19E-15	0.013	0.009	1.90E-01
WHR	rs6548834	A	0.36	0.012	0.002	5.54E-11	-0.008	0.008	3.10E-01
WHR	rs6794936	A	0.48	-0.010	0.002	5.76E-09	-0.011	0.008	2.20E-01
WHR	rs6795831	A	0.81	0.029	0.002	5.68E-36	-0.005	0.010	5.90E-01
WHR	rs6800707	C	0.18	-0.019	0.002	4.28E-16	0.001	0.010	8.60E-01
WHR	rs68190114	A	0.07	-0.022	0.004	1.96E-08	-0.005	0.016	8.40E-01
WHR	rs72628504	A	0.93	-0.033	0.004	2.25E-15	-0.045	0.016	6.70E-03
WHR	rs74388175	T	0.21	-0.013	0.003	8.32E-08	-0.015	0.010	1.30E-01
WHR	rs7647305	T	0.21	-0.014	0.002	1.36E-11	0.019	0.009	2.30E-02
WHR	rs793456	A	0.60	-0.011	0.002	3.89E-09	0.002	0.008	7.00E-01
WHR	rs9859117	C	0.19	0.012	0.002	2.93E-08	0.028	0.010	2.30E-03
WHR	rs9872031	A	0.43	0.020	0.002	3.17E-29	0.011	0.008	1.10E-01
WHR	rs9942009	T	0.38	0.013	0.002	2.56E-12	0.006	0.008	4.60E-01
WHR	rs998749	A	0.50	0.013	0.002	2.06E-14	0.008	0.008	2.30E-01
WHR	rs10004900	T	0.87	-0.017	0.003	9.85E-09	-0.011	0.012	3.90E-01
WHR	rs10019888	A	0.81	-0.021	0.002	1.52E-19	-0.020	0.011	3.90E-02
WHR	rs11133377	A	0.72	0.014	0.002	8.68E-13	0.008	0.009	3.30E-01
WHR	rs11724804	A	0.44	-0.017	0.002	4.88E-20	-0.008	0.008	2.30E-01
WHR	rs11726981	A	0.72	-0.011	0.002	1.20E-08	-0.004	0.009	7.40E-01
WHR	rs12643960	A	0.85	-0.017	0.003	9.89E-10	-0.008	0.011	4.20E-01
WHR	rs13130484	T	0.43	0.015	0.002	5.28E-18	0.016	0.008	4.80E-02
WHR	rs1464454	A	0.40	-0.010	0.002	7.09E-08	-0.021	0.008	8.60E-03
WHR	rs1551795	T	0.51	0.009	0.002	3.21E-08	0.004	0.008	6.70E-01
WHR	rs16896261	A	0.86	-0.014	0.003	1.35E-08	-0.011	0.011	2.50E-01
WHR	rs17644283	A	0.39	0.015	0.002	2.00E-16	-0.004	0.008	5.10E-01
WHR	rs1789882	A	0.17	0.016	0.002	3.49E-12	0.013	0.010	2.40E-01
WHR	rs1863652	A	0.35	-0.010	0.002	5.92E-08	0.002	0.008	8.50E-01
WHR	rs2167750	T	0.47	0.020	0.002	2.06E-28	0.015	0.008	3.70E-02
WHR	rs2333496	T	0.69	0.010	0.002	7.57E-09	-0.004	0.008	8.80E-01
WHR	rs3121419	T	0.30	-0.013	0.002	1.13E-11	0.003	0.008	7.50E-01
WHR	rs3804381	A	0.27	0.012	0.002	5.65E-10	0.008	0.009	4.00E-01
WHR	rs414865	A	0.68	0.011	0.002	9.65E-09	-0.008	0.009	3.70E-01
WHR	rs4450871	A	0.55	0.011	0.002	1.31E-08	0.014	0.008	1.30E-01
WHR	rs4586926	A	0.64	0.011	0.002	3.89E-09	0.015	0.008	3.40E-02
WHR	rs56185013	A	0.22	-0.013	0.002	6.07E-08	0.008	0.009	5.30E-01

WHR	rs7680787	T	0.65	0.010	0.002	7.09E-08	0.004	0.008	6.60E-01
WHR	rs789351	T	0.42	0.012	0.002	4.65E-13	0.000	0.008	1.00E+00
WHR	rs809955	A	0.37	-0.016	0.002	9.19E-15	-0.013	0.008	1.00E-01
WHR	rs10475249	C	0.55	0.013	0.002	2.08E-11	0.008	0.008	2.50E-01
WHR	rs10477191	A	0.94	-0.028	0.004	3.06E-12	0.002	0.019	9.80E-01
WHR	rs11167753	T	0.73	-0.011	0.002	1.20E-08	-0.007	0.009	3.50E-01
WHR	rs1122080	A	0.20	-0.013	0.002	2.61E-09	0.023	0.010	2.30E-02
WHR	rs11747001	A	0.76	0.015	0.002	1.36E-14	0.019	0.009	3.20E-02
WHR	rs11956399	T	0.19	0.016	0.002	6.88E-13	0.021	0.010	9.00E-02
WHR	rs13163336	A	0.17	0.018	0.003	7.59E-12	0.023	0.011	4.30E-02
WHR	rs13179413	T	0.28	0.016	0.002	1.87E-15	0.022	0.009	5.80E-03
WHR	rs1382284	A	0.49	0.010	0.002	3.80E-08	-0.010	0.008	3.30E-01
WHR	rs1382894	A	0.65	0.011	0.002	1.97E-09	0.020	0.008	1.40E-02
WHR	rs17152077	A	0.13	-0.014	0.003	4.25E-08	-0.002	0.011	9.70E-01
WHR	rs17568628	T	0.95	-0.026	0.004	1.97E-09	-0.020	0.018	2.90E-01
WHR	rs17738166	A	0.40	0.011	0.002	2.40E-10	0.003	0.008	7.20E-01
WHR	rs17764730	T	0.24	-0.011	0.002	5.68E-08	0.009	0.009	4.00E-01
WHR	rs2112347	T	0.63	0.015	0.002	1.11E-18	0.016	0.008	3.60E-02
WHR	rs2161097	T	0.44	0.013	0.002	2.56E-12	0.014	0.008	5.00E-02
WHR	rs2448	T	0.74	0.016	0.002	2.79E-15	-0.013	0.009	1.90E-01
WHR	rs2964006	T	0.46	0.013	0.002	8.03E-11	0.010	0.008	1.90E-01
WHR	rs34000	T	0.60	0.011	0.002	2.77E-09	0.012	0.008	1.60E-01
WHR	rs4395620	T	0.58	0.011	0.002	3.89E-09	0.009	0.008	2.90E-01
WHR	rs4454042	T	0.27	0.013	0.002	2.15E-10	0.002	0.009	8.90E-01
WHR	rs459193	A	0.25	0.024	0.002	2.76E-36	-0.014	0.009	1.60E-01
WHR	rs4868256	T	0.50	0.011	0.002	1.31E-08	0.013	0.008	1.10E-01
WHR	rs628763	T	0.58	-0.011	0.002	2.40E-10	-0.004	0.008	6.10E-01
WHR	rs6556301	T	0.37	0.013	0.002	3.39E-13	-0.010	0.008	2.30E-01
WHR	rs6861681	A	0.29	0.018	0.002	1.52E-23	0.004	0.008	4.60E-01
WHR	rs6870983	T	0.21	-0.016	0.002	9.19E-15	-0.007	0.010	4.20E-01
WHR	rs6874848	A	0.70	-0.014	0.002	5.20E-12	-0.006	0.008	5.50E-01
WHR	rs710380	T	0.70	-0.011	0.002	3.80E-08	0.003	0.009	6.90E-01
WHR	rs72846967	A	0.70	-0.012	0.002	3.53E-09	-0.003	0.009	6.90E-01
WHR	rs7736177	A	0.69	-0.011	0.002	3.27E-08	-0.005	0.008	5.50E-01
WHR	rs888675	T	0.39	-0.011	0.002	2.42E-08	0.003	0.008	8.60E-01
WHR	rs10499013	A	0.28	-0.013	0.002	4.10E-10	-0.008	0.009	3.10E-01
WHR	rs10806842	C	0.50	0.011	0.002	3.80E-08	0.008	0.008	2.90E-01
WHR	rs11154521	C	0.32	-0.010	0.002	1.46E-08	-0.014	0.008	3.90E-02
WHR	rs112266013	A	0.14	-0.017	0.003	2.46E-09	-0.012	0.011	2.70E-01
WHR	rs113315602	A	0.91	-0.027	0.004	3.37E-14	-0.013	0.014	3.00E-01
WHR	rs114760566	A	0.04	0.067	0.005	8.67E-45	0.050	0.019	7.30E-03
WHR	rs11752928	C	0.49	-0.011	0.002	3.27E-08	-0.020	0.008	1.10E-02
WHR	rs12527712	T	0.09	0.028	0.003	7.56E-18	0.000	0.015	9.20E-01
WHR	rs1294410	T	0.38	-0.025	0.002	7.89E-48	0.004	0.008	6.10E-01
WHR	rs13191362	A	0.86	0.015	0.003	1.25E-08	0.008	0.012	4.80E-01
WHR	rs13198178	C	0.06	0.021	0.004	7.30E-09	0.024	0.016	1.40E-01
WHR	rs1334576	A	0.43	-0.016	0.002	2.26E-19	-0.011	0.008	2.00E-01
WHR	rs1411338	T	0.34	-0.011	0.002	2.72E-09	-0.014	0.008	4.30E-02
WHR	rs145244672	T	0.88	-0.025	0.003	5.77E-13	-0.004	0.013	6.90E-01
WHR	rs148967059	A	0.20	0.018	0.003	9.44E-12	0.011	0.010	2.70E-01
WHR	rs17078048	A	0.85	-0.014	0.003	5.33E-08	-0.003	0.011	6.50E-01
WHR	rs17448885	C	0.65	0.011	0.002	7.06E-09	0.002	0.008	9.70E-01
WHR	rs17681686	C	0.30	0.015	0.002	8.10E-16	0.005	0.008	5.00E-01
WHR	rs185320691	C	0.10	0.027	0.004	1.48E-13	0.006	0.014	5.80E-01
WHR	rs211476	T	0.30	-0.013	0.002	2.32E-11	0.004	0.009	7.50E-01
WHR	rs2186059	C	0.26	-0.013	0.002	4.10E-10	-0.021	0.009	2.10E-02

WHR	rs2242416	A	0.39	0.014	0.002	2.44E-17	0.010	0.008	1.70E-01
WHR	rs2492419	A	0.58	-0.010	0.002	1.46E-08	-0.008	0.008	2.70E-01
WHR	rs2504706	T	0.77	0.013	0.002	3.26E-10	-0.001	0.009	7.80E-01
WHR	rs2524137	T	0.64	0.023	0.002	4.18E-30	0.023	0.008	4.50E-03
WHR	rs281861363	A	0.09	0.025	0.004	8.04E-12	0.014	0.014	2.90E-01
WHR	rs2844455	T	0.18	0.017	0.002	7.29E-13	0.005	0.010	6.70E-01
WHR	rs35074855	C	0.89	-0.028	0.003	2.16E-17	-0.020	0.013	8.30E-02
WHR	rs35175534	A	0.86	-0.026	0.003	1.13E-16	-0.019	0.012	1.10E-01
WHR	rs377436	A	0.29	-0.011	0.002	9.65E-09	0.001	0.008	7.80E-01
WHR	rs534148	A	0.56	0.014	0.002	1.05E-11	0.021	0.008	1.30E-02
WHR	rs575147125	T	0.84	-0.024	0.003	2.09E-14	-0.028	0.012	1.70E-02
WHR	rs575662827	T	0.90	-0.029	0.004	3.99E-16	-0.011	0.014	4.40E-01
WHR	rs605066	T	0.58	-0.019	0.002	1.46E-26	-0.013	0.008	7.00E-02
WHR	rs62424543	A	0.93	-0.022	0.004	1.53E-08	-0.040	0.015	9.90E-03
WHR	rs668871	T	0.47	-0.013	0.002	2.08E-11	-0.004	0.008	6.40E-01
WHR	rs672341	A	0.41	-0.012	0.002	1.06E-09	-0.008	0.008	4.80E-01
WHR	rs6933271	T	0.99	-0.067	0.008	1.47E-16	-0.083	0.037	1.80E-02
WHR	rs6940715	A	0.89	0.016	0.003	2.32E-08	-0.002	0.013	9.90E-01
WHR	rs6941962	A	0.16	0.024	0.003	7.84E-20	0.014	0.011	1.90E-01
WHR	rs72959041	A	0.04	0.126	0.004	2.37E-180	0.038	0.018	2.30E-02
WHR	rs7744833	A	0.68	0.013	0.002	4.74E-11	0.008	0.008	3.50E-01
WHR	rs901630	T	0.41	-0.012	0.002	2.56E-12	-0.023	0.008	2.40E-03
WHR	rs9296938	A	0.25	-0.015	0.002	4.17E-13	-0.021	0.009	3.50E-02
WHR	rs9362083	A	0.59	-0.012	0.002	8.03E-11	0.011	0.008	2.60E-01
WHR	rs9369425	A	0.71	0.020	0.002	1.03E-24	-0.001	0.009	8.20E-01
WHR	rs9370243	T	0.08	0.020	0.003	6.11E-10	0.023	0.014	8.80E-02
WHR	rs9375417	T	0.50	-0.012	0.002	1.06E-09	-0.003	0.008	6.00E-01
WHR	rs9375478	A	0.54	0.021	0.002	2.51E-30	0.017	0.008	5.20E-02
WHR	rs9400239	T	0.31	-0.013	0.002	9.74E-14	0.012	0.008	1.80E-01
WHR	rs987237	A	0.84	-0.020	0.002	9.82E-20	0.009	0.010	4.00E-01
WHR	rs998584	A	0.48	0.035	0.002	1.04E-94	0.018	0.008	2.20E-02
WHR	rs1011024	A	0.83	0.015	0.002	2.89E-11	0.041	0.011	1.40E-04
WHR	rs10269616	T	0.56	-0.011	0.002	3.80E-08	0.001	0.008	9.20E-01
WHR	rs10269783	A	0.41	0.010	0.002	1.63E-08	0.017	0.008	2.20E-02
WHR	rs1142	T	0.33	0.015	0.002	1.25E-16	0.003	0.008	7.40E-01
WHR	rs11764879	A	0.29	-0.012	0.002	7.37E-10	-0.002	0.009	9.30E-01
WHR	rs12669521	A	0.67	0.013	0.002	1.47E-09	0.017	0.008	3.90E-02
WHR	rs12705971	T	0.46	0.009	0.002	6.24E-08	0.007	0.008	3.80E-01
WHR	rs13229637	T	0.85	0.016	0.003	2.02E-10	0.004	0.011	7.00E-01
WHR	rs13232789	T	0.66	-0.011	0.002	7.06E-09	-0.016	0.008	4.80E-02
WHR	rs1534696	A	0.57	-0.023	0.002	2.50E-40	-0.004	0.008	5.60E-01
WHR	rs1708302	T	0.43	-0.011	0.002	2.40E-10	-0.022	0.008	7.80E-03
WHR	rs1718618	A	0.06	-0.024	0.004	2.69E-10	-0.012	0.017	3.90E-01
WHR	rs1722123	T	0.48	-0.011	0.002	1.60E-08	-0.005	0.008	4.30E-01
WHR	rs2057869	A	0.30	0.011	0.002	2.86E-08	0.008	0.008	3.00E-01
WHR	rs2069443	T	0.74	-0.012	0.002	1.46E-08	-0.011	0.009	2.00E-01
WHR	rs2391168	A	0.19	0.026	0.002	3.31E-35	0.021	0.010	3.40E-02
WHR	rs2715135	T	0.37	0.011	0.002	1.40E-09	0.006	0.008	4.10E-01
WHR	rs367026	T	0.61	-0.014	0.002	3.65E-12	-0.002	0.008	8.20E-01
WHR	rs3930017	A	0.39	-0.010	0.002	2.77E-08	0.004	0.008	6.50E-01
WHR	rs39312	A	0.62	-0.015	0.002	1.17E-17	-0.002	0.008	6.20E-01
WHR	rs4476935	T	0.43	-0.011	0.002	1.97E-09	-0.004	0.008	5.20E-01
WHR	rs4718966	T	0.41	0.010	0.002	1.46E-08	0.018	0.008	3.70E-02
WHR	rs4727695	A	0.90	0.022	0.003	1.23E-13	0.016	0.013	2.40E-01
WHR	rs55747707	A	0.18	-0.015	0.002	2.15E-11	0.002	0.010	8.80E-01
WHR	rs6942652	C	0.43	-0.013	0.002	2.56E-12	-0.015	0.008	7.30E-02

WHR	rs6955431	T	0.69	0.011	0.002	1.97E-09	0.016	0.008	8.00E-02
WHR	rs77497827	A	0.53	-0.013	0.002	1.02E-08	-0.023	0.009	6.50E-03
WHR	rs7801581	T	0.25	0.017	0.002	6.83E-17	-0.002	0.009	6.60E-01
WHR	rs852425	A	0.65	-0.010	0.002	1.46E-08	-0.023	0.008	3.20E-03
WHR	rs10505628	T	0.38	-0.010	0.002	7.57E-09	0.000	0.008	9.90E-01
WHR	rs11782074	T	0.37	0.011	0.002	9.65E-09	-0.003	0.008	7.00E-01
WHR	rs11992444	T	0.51	0.019	0.002	2.24E-20	-0.010	0.008	1.80E-01
WHR	rs13255070	A	0.72	-0.014	0.002	6.34E-10	-0.013	0.009	1.00E-01
WHR	rs13256367	A	0.65	0.014	0.002	1.16E-13	0.007	0.008	5.30E-01
WHR	rs1431659	A	0.27	0.014	0.002	8.68E-13	0.001	0.009	7.80E-01
WHR	rs144435165	C	0.05	0.035	0.005	1.06E-12	0.020	0.019	2.90E-01
WHR	rs1485741	T	0.12	0.018	0.003	9.08E-11	-0.008	0.012	6.70E-01
WHR	rs15285	T	0.26	-0.012	0.002	7.37E-10	-0.006	0.009	4.40E-01
WHR	rs17446091	T	0.79	-0.012	0.002	4.35E-08	-0.023	0.010	1.30E-02
WHR	rs2012485	T	0.05	-0.025	0.004	1.98E-08	-0.008	0.019	7.50E-01
WHR	rs2725371	A	0.30	0.017	0.002	2.60E-16	0.005	0.008	4.60E-01
WHR	rs35708461	T	0.22	0.014	0.002	1.86E-08	0.006	0.009	7.00E-01
WHR	rs4145698	A	0.66	0.011	0.002	1.31E-08	-0.005	0.008	6.70E-01
WHR	rs4738141	A	0.74	-0.020	0.002	6.53E-26	-0.015	0.009	9.80E-02
WHR	rs62505960	A	0.91	0.020	0.004	2.14E-08	0.007	0.014	5.80E-01
WHR	rs62506196	A	0.84	-0.016	0.003	3.89E-09	0.001	0.011	9.40E-01
WHR	rs6988242	T	0.72	0.012	0.002	2.53E-08	0.008	0.009	4.10E-01
WHR	rs7823561	A	0.65	0.016	0.002	5.84E-17	0.005	0.008	6.20E-01
WHR	rs881301	T	0.58	-0.013	0.002	9.74E-14	-0.005	0.008	6.60E-01
WHR	rs9643248	T	0.68	-0.012	0.002	1.46E-08	-0.018	0.008	2.50E-02
WHR	rs9644033	A	0.75	0.019	0.002	3.48E-19	0.019	0.009	4.90E-02
WHR	rs9969455	A	0.62	0.011	0.002	6.97E-10	-0.005	0.008	4.20E-01
WHR	rs10797116	T	0.46	-0.010	0.002	2.01E-08	-0.008	0.008	2.30E-01
WHR	rs10963067	A	0.09	-0.018	0.003	2.66E-08	0.011	0.014	3.80E-01
WHR	rs10968576	A	0.69	-0.014	0.002	1.24E-15	-0.006	0.008	5.70E-01
WHR	rs10978310	A	0.94	0.023	0.004	5.03E-09	0.018	0.018	3.30E-01
WHR	rs10980797	A	0.52	-0.015	0.002	1.36E-14	0.008	0.008	3.00E-01
WHR	rs10991433	T	0.90	-0.025	0.003	1.17E-19	0.008	0.013	5.20E-01
WHR	rs12684047	A	0.19	-0.019	0.002	8.73E-16	-0.004	0.010	5.60E-01
WHR	rs1411431	A	0.16	0.015	0.003	1.81E-08	0.003	0.011	7.90E-01
WHR	rs1680490	A	0.59	-0.012	0.002	3.64E-09	0.002	0.008	6.50E-01
WHR	rs1752169	A	0.26	0.012	0.002	7.75E-10	0.024	0.009	7.20E-03
WHR	rs1800978	C	0.88	0.020	0.003	1.07E-14	-0.023	0.012	7.10E-02
WHR	rs2043664	A	0.31	-0.011	0.002	5.04E-08	-0.003	0.008	7.00E-01
WHR	rs2165864	T	0.43	-0.010	0.002	1.46E-08	-0.010	0.008	1.30E-01
WHR	rs2398893	A	0.71	0.016	0.002	1.42E-16	0.010	0.009	2.60E-01
WHR	rs28647893	T	0.48	-0.012	0.002	6.63E-09	-0.003	0.008	6.40E-01
WHR	rs2937371	A	0.57	-0.012	0.002	1.35E-10	-0.003	0.008	7.80E-01
WHR	rs4382592	T	0.31	0.012	0.002	2.69E-10	0.013	0.008	5.90E-02
WHR	rs4837261	T	0.18	0.013	0.002	2.04E-08	0.017	0.010	8.90E-02
WHR	rs62565259	T	0.16	-0.016	0.003	6.07E-09	-0.011	0.010	2.50E-01
WHR	rs6474945	T	0.45	-0.010	0.002	2.83E-09	-0.013	0.008	1.00E-01
WHR	rs71511786	A	0.92	0.021	0.004	8.92E-09	-0.016	0.014	2.30E-01
WHR	rs753804	C	0.17	0.017	0.002	7.53E-14	0.013	0.010	2.10E-01
WHR	rs7848336	T	0.71	-0.013	0.002	4.11E-11	0.008	0.009	3.40E-01
WHR	rs7848552	C	0.20	0.013	0.002	2.62E-08	0.034	0.010	9.20E-04
WHR	rs7859156	T	0.28	-0.011	0.002	2.86E-08	0.000	0.009	9.90E-01
WHR	rs968821	C	0.34	-0.011	0.002	5.15E-09	-0.006	0.008	3.90E-01
WHR	rs9792666	A	0.96	0.040	0.005	6.11E-18	0.026	0.020	2.10E-01
WHR	rs10761785	T	0.51	-0.015	0.002	5.28E-18	0.009	0.008	3.30E-01
WHR	rs10788569	T	0.72	-0.013	0.002	1.76E-12	-0.007	0.009	6.80E-01

WHR	rs10795055	A	0.39	0.011	0.002	3.89E-09	-0.001	0.008	9.60E-01
WHR	rs10827252	A	0.49	-0.011	0.002	3.43E-10	0.009	0.008	2.30E-01
WHR	rs10883518	A	0.37	-0.011	0.002	7.06E-09	0.006	0.008	5.60E-01
WHR	rs11187537	C	0.26	0.013	0.002	8.03E-11	0.015	0.009	9.90E-02
WHR	rs117471638	A	0.02	0.042	0.007	3.60E-09	0.008	0.027	9.20E-01
WHR	rs1243188	T	0.71	-0.015	0.002	6.73E-15	-0.018	0.008	6.40E-02
WHR	rs1250552	A	0.52	-0.009	0.002	3.21E-08	-0.014	0.008	9.40E-02
WHR	rs12774134	T	0.12	-0.016	0.003	1.23E-09	-0.024	0.012	6.20E-02
WHR	rs12777288	T	0.77	-0.014	0.002	3.62E-11	-0.012	0.009	1.20E-01
WHR	rs1437	A	0.63	0.013	0.002	1.15E-12	0.012	0.008	1.20E-01
WHR	rs145952040	T	0.96	-0.033	0.005	2.21E-10	-0.003	0.021	9.60E-01
WHR	rs1494204	T	0.42	-0.011	0.002	3.89E-09	-0.012	0.008	1.40E-01
WHR	rs1757471	T	0.51	0.013	0.002	1.15E-12	-0.001	0.008	8.10E-01
WHR	rs2254069	A	0.13	0.021	0.003	9.88E-15	0.017	0.012	1.70E-01
WHR	rs2907794	A	0.25	-0.012	0.002	5.65E-10	-0.002	0.009	6.40E-01
WHR	rs3740237	C	0.14	0.015	0.003	3.99E-09	0.005	0.011	4.40E-01
WHR	rs3814614	A	0.48	0.011	0.002	1.97E-09	0.007	0.008	4.40E-01
WHR	rs4752083	A	0.62	-0.011	0.002	1.20E-08	-0.007	0.008	5.10E-01
WHR	rs704000	T	0.47	0.011	0.002	5.04E-08	-0.013	0.008	1.20E-01
WHR	rs7070670	T	0.32	-0.013	0.002	3.26E-10	-0.008	0.008	3.10E-01
WHR	rs7070749	A	0.55	0.013	0.002	2.98E-10	0.016	0.008	6.30E-02
WHR	rs708437	A	0.83	0.014	0.002	1.97E-09	-0.001	0.010	9.80E-01
WHR	rs780159	A	0.43	-0.013	0.002	5.14E-15	0.003	0.008	7.70E-01
WHR	rs7898903	T	0.13	-0.015	0.003	4.22E-08	-0.022	0.012	7.80E-02
WHR	rs7907173	A	0.45	-0.011	0.002	1.40E-09	-0.008	0.008	5.00E-01
WHR	rs7919055	T	0.96	-0.028	0.005	3.69E-10	-0.005	0.022	8.60E-01
WHR	rs10789931	T	0.12	0.015	0.003	2.77E-08	-0.008	0.012	5.10E-01
WHR	rs10791249	A	0.44	0.010	0.002	7.57E-09	0.010	0.008	1.80E-01
WHR	rs10896012	T	0.79	-0.018	0.002	2.80E-16	-0.004	0.009	7.00E-01
WHR	rs11030107	A	0.73	-0.018	0.002	7.36E-21	-0.019	0.009	4.80E-02
WHR	rs11038354	A	0.57	0.011	0.002	5.43E-09	0.003	0.008	6.50E-01
WHR	rs11214589	A	0.50	-0.011	0.002	2.40E-10	0.011	0.008	2.40E-01
WHR	rs11216183	A	0.09	0.023	0.003	2.99E-11	0.016	0.013	2.30E-01
WHR	rs11231144	T	0.65	-0.016	0.002	3.41E-16	-0.007	0.008	3.50E-01
WHR	rs12286929	A	0.50	-0.009	0.002	3.21E-08	-0.005	0.008	4.80E-01
WHR	rs12287076	C	0.63	0.019	0.002	7.99E-22	-0.012	0.009	1.80E-01
WHR	rs12575252	C	0.33	-0.015	0.002	1.17E-17	-0.023	0.008	2.70E-03
WHR	rs13642	A	0.65	0.011	0.002	2.72E-09	0.008	0.008	3.10E-01
WHR	rs140201358	C	0.99	-0.056	0.009	9.37E-11	-0.154	0.032	2.70E-06
WHR	rs140829434	A	0.02	-0.044	0.008	3.41E-08	-0.058	0.031	1.00E-01
WHR	rs2276390	T	0.34	-0.021	0.002	2.13E-28	0.003	0.008	6.70E-01
WHR	rs2509963	T	0.26	-0.014	0.002	8.68E-13	0.003	0.009	7.20E-01
WHR	rs2512885	T	0.47	-0.010	0.002	1.16E-08	-0.007	0.008	3.50E-01
WHR	rs2513987	A	0.27	-0.014	0.002	3.55E-10	-0.012	0.009	2.30E-01
WHR	rs2957658	A	0.49	-0.013	0.002	4.10E-10	-0.017	0.008	1.80E-02
WHR	rs35169799	T	0.06	0.037	0.004	9.24E-20	0.026	0.016	1.40E-01
WHR	rs3825061	T	0.39	0.014	0.002	3.05E-15	0.007	0.008	3.00E-01
WHR	rs3862386	C	0.19	0.014	0.002	1.15E-09	0.002	0.010	8.90E-01
WHR	rs3930078	A	0.16	0.014	0.002	1.86E-08	0.014	0.010	1.30E-01
WHR	rs4141261	T	0.36	-0.011	0.002	9.65E-09	0.003	0.008	8.40E-01
WHR	rs4418806	T	0.45	-0.012	0.002	8.92E-09	-0.005	0.008	4.50E-01
WHR	rs4755720	T	0.61	-0.013	0.002	2.24E-13	-0.020	0.008	5.10E-03
WHR	rs536665	A	0.80	-0.017	0.002	1.04E-12	-0.013	0.010	9.50E-02
WHR	rs579682	T	0.71	-0.013	0.002	2.93E-11	-0.020	0.009	1.80E-02
WHR	rs61876729	A	0.91	0.021	0.003	3.09E-10	0.030	0.013	2.10E-02
WHR	rs647248	A	0.54	0.011	0.002	5.43E-09	0.009	0.008	2.30E-01

WHR	rs6590683	T	0.48	0.011	0.002	3.43E-10	0.002	0.008	8.00E-01
WHR	rs6606672	A	0.65	-0.012	0.002	8.03E-11	-0.002	0.008	8.80E-01
WHR	rs67184556	T	0.32	-0.012	0.002	2.53E-08	-0.011	0.008	1.60E-01
WHR	rs68162171	A	0.71	-0.016	0.002	1.79E-13	-0.022	0.009	1.30E-02
WHR	rs72889643	T	0.06	0.023	0.004	1.03E-08	-0.014	0.016	4.60E-01
WHR	rs7395513	A	0.44	-0.017	0.002	5.15E-18	0.001	0.008	9.50E-01
WHR	rs747249	A	0.36	0.011	0.002	5.15E-09	0.016	0.008	5.90E-02
WHR	rs747601	A	0.28	-0.013	0.002	1.55E-10	0.000	0.009	9.30E-01
WHR	rs7932891	A	0.30	0.011	0.002	1.60E-08	0.003	0.008	8.10E-01
WHR	rs1026462	A	0.52	-0.013	0.002	2.56E-12	-0.012	0.008	1.80E-01
WHR	rs10506110	A	0.64	0.014	0.002	3.78E-13	0.004	0.008	6.20E-01
WHR	rs10745659	C	0.56	-0.013	0.002	1.72E-12	0.001	0.008	9.70E-01
WHR	rs10861841	A	0.42	0.011	0.002	6.97E-10	0.011	0.008	2.20E-01
WHR	rs10876528	A	0.35	0.024	0.002	2.84E-39	-0.007	0.008	3.10E-01
WHR	rs11048456	T	0.76	-0.027	0.002	1.56E-44	-0.012	0.009	1.40E-01
WHR	rs11055887	A	0.19	-0.013	0.002	7.36E-09	-0.016	0.010	6.20E-02
WHR	rs11176015	T	0.29	0.015	0.002	4.35E-14	-0.002	0.009	8.20E-01
WHR	rs11608693	A	0.17	-0.017	0.003	4.90E-10	0.000	0.011	9.60E-01
WHR	rs12823266	A	0.72	0.011	0.002	5.68E-08	0.010	0.009	2.50E-01
WHR	rs12828016	T	0.39	-0.011	0.002	2.40E-10	-0.011	0.008	1.60E-01
WHR	rs12828318	A	0.83	0.013	0.002	1.58E-08	0.011	0.010	1.90E-01
WHR	rs1443512	A	0.22	0.028	0.002	3.80E-45	0.008	0.009	3.70E-01
WHR	rs1568427	A	0.26	-0.019	0.002	3.48E-19	-0.002	0.009	7.70E-01
WHR	rs1609725	T	0.07	0.021	0.003	1.65E-09	0.019	0.016	2.40E-01
WHR	rs2277339	T	0.89	-0.017	0.003	6.91E-09	-0.020	0.013	1.30E-01
WHR	rs317646	A	0.73	-0.011	0.002	3.80E-08	0.001	0.009	5.70E-01
WHR	rs34322	T	0.47	0.010	0.002	7.57E-09	0.009	0.008	2.20E-01
WHR	rs3764002	T	0.26	-0.021	0.002	5.08E-26	-0.008	0.009	3.40E-01
WHR	rs4459369	T	0.73	0.012	0.002	4.35E-08	-0.004	0.009	5.90E-01
WHR	rs4765219	A	0.34	-0.027	0.002	8.92E-50	-0.025	0.008	3.10E-03
WHR	rs4964188	T	0.50	-0.011	0.002	2.14E-08	-0.005	0.008	5.00E-01
WHR	rs544668	T	0.60	0.011	0.002	5.43E-09	0.006	0.008	3.70E-01
WHR	rs6487543	A	0.76	0.012	0.002	6.27E-09	0.004	0.009	7.00E-01
WHR	rs7138803	A	0.38	0.013	0.002	3.80E-12	0.001	0.008	8.30E-01
WHR	rs7222	T	0.50	0.011	0.002	1.40E-09	0.006	0.008	5.70E-01
WHR	rs729062	A	0.93	-0.020	0.004	1.46E-08	0.003	0.015	8.90E-01
WHR	rs7311622	T	0.45	-0.012	0.002	3.81E-11	0.012	0.008	1.20E-01
WHR	rs74628422	A	0.20	0.014	0.002	4.23E-09	-0.012	0.010	1.90E-01
WHR	rs79204097	A	0.10	-0.019	0.003	1.63E-08	-0.014	0.013	3.10E-01
WHR	rs7961979	A	0.13	0.016	0.003	6.07E-09	0.011	0.012	2.30E-01
WHR	rs863750	T	0.58	0.026	0.002	2.06E-52	0.014	0.008	6.60E-02
WHR	rs1163627	A	0.54	0.010	0.002	9.50E-10	0.012	0.008	1.20E-01
WHR	rs12430764	A	0.52	0.011	0.002	2.77E-09	-0.005	0.008	5.50E-01
WHR	rs1360485	T	0.68	0.015	0.002	3.05E-17	0.016	0.008	3.80E-02
WHR	rs1379828	T	0.81	-0.013	0.002	4.43E-10	-0.014	0.010	1.40E-01
WHR	rs1441264	A	0.59	0.011	0.002	6.97E-10	0.002	0.008	9.10E-01
WHR	rs2225226	T	0.21	0.012	0.002	3.32E-08	0.004	0.009	6.60E-01
WHR	rs2475837	T	0.12	0.015	0.003	6.33E-09	-0.003	0.012	6.50E-01
WHR	rs4055791	T	0.42	-0.012	0.002	3.64E-09	-0.014	0.008	8.50E-02
WHR	rs664532	T	0.38	0.011	0.002	2.72E-09	0.008	0.008	3.50E-01
WHR	rs7350648	T	0.80	0.014	0.003	3.39E-08	0.004	0.010	7.10E-01
WHR	rs797486	A	0.89	0.032	0.003	5.47E-34	0.011	0.012	3.20E-01
WHR	rs7985834	A	0.86	-0.017	0.003	6.20E-10	-0.007	0.012	5.10E-01
WHR	rs9515201	A	0.31	-0.012	0.002	1.79E-11	-0.020	0.009	7.90E-03
WHR	rs9556979	T	0.69	-0.011	0.002	2.72E-09	-0.031	0.008	4.60E-04
WHR	rs9596270	T	0.93	0.027	0.003	7.70E-16	0.044	0.016	8.30E-03

WHR	rs10132280	A	0.31	-0.012	0.002	1.16E-10	0.002	0.008	6.30E-01
WHR	rs10133795	A	0.51	-0.010	0.002	7.57E-09	-0.002	0.008	8.90E-01
WHR	rs1190982	T	0.30	0.016	0.002	1.42E-16	-0.003	0.008	5.10E-01
WHR	rs17109256	A	0.23	0.017	0.002	8.44E-16	0.000	0.009	1.00E+00
WHR	rs2205189	A	0.28	0.011	0.002	1.60E-08	0.002	0.009	9.20E-01
WHR	rs2412107	T	0.22	0.013	0.002	2.40E-10	-0.004	0.009	5.90E-01
WHR	rs2526886	T	0.69	0.013	0.002	2.64E-09	0.016	0.008	2.50E-02
WHR	rs4902632	A	0.18	0.016	0.002	1.06E-10	0.006	0.010	5.50E-01
WHR	rs61986159	T	0.23	0.014	0.002	3.36E-09	0.000	0.009	9.40E-01
WHR	rs699371	T	0.36	-0.011	0.002	2.42E-08	-0.003	0.008	4.80E-01
WHR	rs7143963	T	0.17	0.014	0.002	6.34E-10	0.003	0.010	7.30E-01
WHR	rs7492628	C	0.68	-0.013	0.002	4.74E-11	-0.026	0.008	1.70E-03
WHR	rs11071759	T	0.39	0.010	0.002	7.57E-09	0.008	0.008	2.90E-01
WHR	rs11854996	A	0.74	0.011	0.002	1.20E-08	-0.007	0.009	4.20E-01
WHR	rs12101393	C	0.79	0.014	0.002	1.97E-10	0.014	0.009	1.00E-01
WHR	rs12440605	A	0.52	0.012	0.002	5.54E-11	-0.003	0.008	5.80E-01
WHR	rs12440695	T	0.62	-0.010	0.002	1.05E-08	0.002	0.008	6.90E-01
WHR	rs12593088	A	0.31	-0.013	0.002	1.62E-11	-0.013	0.008	1.40E-01
WHR	rs12595496	A	0.87	-0.018	0.003	1.08E-12	-0.019	0.012	1.50E-01
WHR	rs12908637	C	0.87	0.016	0.003	7.57E-09	0.024	0.011	2.80E-02
WHR	rs13329447	T	0.61	-0.010	0.002	2.01E-08	-0.007	0.008	4.20E-01
WHR	rs1657930	A	0.79	-0.013	0.002	1.12E-09	-0.001	0.010	9.90E-01
WHR	rs17158366	A	0.10	-0.016	0.003	1.10E-08	-0.002	0.013	8.00E-01
WHR	rs1992145	A	0.55	0.011	0.002	6.97E-10	0.010	0.008	2.90E-01
WHR	rs2061007	C	0.59	-0.011	0.002	5.43E-09	-0.007	0.008	3.30E-01
WHR	rs2456530	T	0.14	0.016	0.003	3.63E-10	0.013	0.011	2.40E-01
WHR	rs2469081	T	0.27	-0.013	0.002	1.12E-10	0.020	0.009	2.20E-02
WHR	rs3092982	C	0.50	0.010	0.002	1.46E-08	-0.001	0.008	9.60E-01
WHR	rs3736485	A	0.46	0.012	0.002	3.89E-12	0.002	0.008	8.80E-01
WHR	rs4779526	A	0.76	0.013	0.002	3.26E-10	0.002	0.009	8.30E-01
WHR	rs7183908	T	0.51	-0.016	0.002	2.79E-15	-0.004	0.008	6.10E-01
WHR	rs8024294	A	0.11	0.018	0.003	2.59E-10	0.001	0.013	9.60E-01
WHR	rs8039418	T	0.43	-0.011	0.002	3.43E-10	-0.023	0.008	9.50E-04
WHR	rs8043060	A	0.22	-0.017	0.002	1.04E-16	-0.003	0.009	7.60E-01
WHR	rs1558902	A	0.41	0.040	0.002	3.24E-121	0.024	0.008	2.60E-03
WHR	rs2008514	A	0.38	0.017	0.002	1.52E-23	0.018	0.008	1.80E-02
WHR	rs2047937	T	0.52	-0.011	0.002	4.51E-10	-0.002	0.008	8.10E-01
WHR	rs220381	A	0.67	-0.011	0.002	2.42E-08	-0.003	0.008	7.20E-01
WHR	rs2531992	A	0.16	-0.013	0.002	1.58E-08	-0.016	0.011	1.70E-01
WHR	rs2925979	T	0.30	0.022	0.002	6.94E-33	0.019	0.008	2.20E-02
WHR	rs3747579	T	0.71	-0.015	0.002	7.86E-17	-0.015	0.009	8.00E-02
WHR	rs400223	T	0.48	0.010	0.002	2.77E-08	0.006	0.008	4.80E-01
WHR	rs4243130	T	0.81	-0.012	0.002	8.32E-09	-0.017	0.010	6.20E-02
WHR	rs4782289	A	0.13	-0.015	0.003	5.22E-09	-0.035	0.011	2.70E-03
WHR	rs4788204	A	0.46	0.017	0.002	6.06E-21	-0.001	0.008	9.60E-01
WHR	rs71385734	T	0.83	0.019	0.003	1.53E-13	-0.004	0.010	6.90E-01
WHR	rs7186893	T	0.26	-0.014	0.002	7.38E-12	-0.015	0.009	1.10E-01
WHR	rs7198287	T	0.22	-0.014	0.002	3.55E-10	-0.010	0.010	2.80E-01
WHR	rs7206608	C	0.68	-0.013	0.002	2.32E-11	-0.016	0.008	4.00E-02
WHR	rs8054299	C	0.69	0.011	0.002	1.79E-08	0.017	0.008	3.30E-02
WHR	rs8054765	C	0.77	-0.012	0.002	8.32E-09	-0.010	0.009	2.50E-01
WHR	rs8060576	T	0.12	0.016	0.003	2.48E-09	0.016	0.012	1.40E-01
WHR	rs889398	T	0.41	-0.017	0.002	8.40E-24	-0.016	0.008	4.90E-02
WHR	rs929069	A	0.19	0.013	0.002	2.62E-08	0.026	0.010	1.30E-02
WHR	rs10512605	T	0.08	0.023	0.003	2.56E-12	0.043	0.014	3.00E-03
WHR	rs11654387	C	0.49	0.016	0.002	3.74E-19	-0.002	0.008	5.90E-01

WHR	rs11654395	T	0.92	-0.018	0.003	4.14E-08	-0.030	0.014	1.00E-02
WHR	rs12450225	A	0.50	-0.011	0.002	6.56E-10	0.000	0.008	7.90E-01
WHR	rs12601665	C	0.58	0.010	0.002	2.77E-08	0.002	0.008	9.60E-01
WHR	rs12602912	T	0.21	0.018	0.002	5.25E-17	0.006	0.010	6.20E-01
WHR	rs2306589	T	0.47	0.016	0.002	4.88E-21	0.002	0.008	9.60E-01
WHR	rs4239275	T	0.41	0.012	0.002	5.54E-11	0.010	0.008	2.00E-01
WHR	rs55938136	A	0.78	-0.018	0.002	2.44E-14	-0.015	0.009	1.20E-01
WHR	rs591939	A	0.75	-0.018	0.002	1.41E-14	-0.011	0.009	2.40E-01
WHR	rs62063286	T	0.78	0.024	0.002	6.55E-24	0.019	0.010	5.50E-02
WHR	rs672356	A	0.30	-0.013	0.002	3.26E-10	0.005	0.008	5.80E-01
WHR	rs7209595	C	0.45	0.014	0.002	4.17E-14	0.002	0.008	7.40E-01
WHR	rs7213608	T	0.68	-0.017	0.002	2.26E-19	-0.005	0.008	4.30E-01
WHR	rs7217226	T	0.66	-0.014	0.002	2.72E-14	-0.001	0.008	8.60E-01
WHR	rs7222766	A	0.47	0.009	0.002	4.49E-08	-0.005	0.008	5.60E-01
WHR	rs7223966	A	0.29	-0.014	0.002	5.57E-13	0.001	0.009	9.50E-01
WHR	rs72820838	T	0.05	0.025	0.004	7.80E-09	0.002	0.017	7.50E-01
WHR	rs77617442	T	0.28	0.012	0.002	2.93E-08	-0.017	0.009	5.30E-02
WHR	rs8070737	T	0.17	0.016	0.002	3.49E-12	-0.009	0.010	3.20E-01
WHR	rs8071778	C	0.15	-0.016	0.003	1.55E-10	-0.007	0.011	4.70E-01
WHR	rs8074344	A	0.43	0.012	0.002	6.63E-09	0.014	0.008	8.80E-02
WHR	rs858519	T	0.45	0.012	0.002	1.45E-09	0.017	0.008	2.70E-02
WHR	rs9895436	A	0.41	-0.011	0.002	5.43E-09	0.005	0.008	5.30E-01
WHR	rs9905140	T	0.39	0.011	0.002	2.00E-11	-0.008	0.008	3.70E-01
WHR	rs9988	T	0.83	0.015	0.002	2.40E-10	0.025	0.010	2.80E-02
WHR	rs10164099	T	0.87	-0.015	0.003	2.52E-09	-0.002	0.011	7.90E-01
WHR	rs1158805	A	0.38	-0.013	0.002	1.48E-13	-0.020	0.008	1.20E-02
WHR	rs11664106	A	0.63	0.020	0.002	1.67E-21	0.014	0.008	6.90E-02
WHR	rs1787013	T	0.56	-0.011	0.002	1.97E-09	-0.013	0.008	8.80E-02
WHR	rs28434748	A	0.80	-0.015	0.002	2.40E-10	-0.005	0.010	5.00E-01
WHR	rs2981423	T	0.94	0.019	0.004	2.98E-08	0.008	0.016	6.70E-01
WHR	rs62095889	A	0.34	-0.017	0.002	2.68E-15	-0.002	0.008	8.60E-01
WHR	rs6567160	T	0.75	-0.026	0.002	1.70E-39	0.004	0.009	8.60E-01
WHR	rs7235891	T	0.52	-0.012	0.002	1.16E-10	-0.002	0.008	8.00E-01
WHR	rs7239114	A	0.54	0.010	0.002	2.29E-08	0.008	0.008	2.20E-01
WHR	rs8096564	T	0.28	0.011	0.002	1.20E-08	0.014	0.009	1.00E-01
WHR	rs9947450	T	0.04	-0.038	0.004	1.83E-18	-0.016	0.020	4.30E-01
WHR	rs9951872	A	0.16	0.021	0.002	9.22E-18	0.007	0.011	5.30E-01
WHR	rs1035942	A	0.27	0.012	0.002	1.45E-09	0.018	0.009	2.90E-02
WHR	rs11084735	A	0.68	0.013	0.002	2.32E-11	-0.009	0.008	3.20E-01
WHR	rs12459350	A	0.54	0.013	0.002	3.21E-15	0.001	0.008	9.70E-01
WHR	rs12461964	A	0.51	-0.013	0.002	4.10E-10	-0.005	0.008	5.00E-01
WHR	rs12608504	A	0.36	0.025	0.002	3.40E-44	0.031	0.008	6.20E-05
WHR	rs142927801	A	0.02	-0.041	0.007	1.42E-08	-0.059	0.028	4.70E-02
WHR	rs17724992	A	0.72	0.014	0.002	5.22E-14	-0.014	0.009	1.20E-01
WHR	rs1800437	C	0.19	-0.021	0.002	2.10E-21	0.015	0.010	1.00E-01
WHR	rs34132828	A	0.69	-0.016	0.002	4.60E-11	0.006	0.010	5.10E-01
WHR	rs350846	C	0.12	0.015	0.003	3.80E-08	-0.005	0.012	7.50E-01
WHR	rs3786897	A	0.58	-0.024	0.002	3.56E-44	0.003	0.008	6.60E-01
WHR	rs3810291	A	0.65	0.012	0.002	1.79E-11	-0.004	0.008	6.60E-01
WHR	rs429358	T	0.85	0.035	0.003	1.35E-37	0.121	0.011	1.50E-29
WHR	rs439223	A	0.08	0.024	0.003	8.87E-13	0.042	0.015	8.70E-03
WHR	rs4808845	A	0.59	0.012	0.002	1.06E-09	0.010	0.008	2.80E-01
WHR	rs55957788	A	0.30	0.013	0.002	6.00E-10	0.007	0.009	3.50E-01
WHR	rs7257330	A	0.38	0.013	0.002	2.24E-13	-0.002	0.008	8.90E-01
WHR	rs8103017	C	0.70	-0.017	0.002	6.38E-14	0.003	0.009	5.80E-01
WHR	rs998732	A	0.83	0.017	0.002	7.29E-13	0.011	0.011	3.30E-01

WHR	rs1328757	T	0.47	0.011	0.002	2.40E-10	-0.003	0.008	6.90E-01
WHR	rs143384	A	0.59	0.016	0.002	7.68E-20	-0.009	0.008	3.20E-01
WHR	rs1997833	T	0.71	-0.012	0.002	1.35E-10	-0.002	0.009	7.70E-01
WHR	rs2104574	T	0.30	-0.010	0.002	7.94E-08	-0.009	0.009	3.30E-01
WHR	rs2236519	A	0.37	0.021	0.002	5.08E-32	0.011	0.008	1.70E-01
WHR	rs3092781	T	0.45	-0.015	0.002	1.47E-17	-0.020	0.008	9.00E-03
WHR	rs4812700	T	0.14	-0.018	0.003	2.78E-10	-0.008	0.012	5.50E-01
WHR	rs6021889	A	0.71	0.020	0.002	1.21E-26	0.020	0.009	2.40E-02
WHR	rs7267979	A	0.44	0.010	0.002	2.83E-09	0.028	0.008	1.30E-04
WHR	rs805770	T	0.40	0.018	0.002	4.65E-23	0.014	0.008	9.90E-02
WHR	rs910382	A	0.49	-0.017	0.002	6.06E-21	-0.009	0.008	2.80E-01
WHR	rs979012	T	0.36	0.012	0.002	1.16E-10	0.012	0.008	8.80E-02
WHR	rs2823096	A	0.81	0.013	0.002	2.62E-08	0.007	0.010	4.90E-01
WHR	rs2836179	A	0.41	-0.013	0.002	2.24E-13	-0.017	0.008	2.80E-02
WHR	rs2838006	T	0.36	-0.011	0.002	9.65E-09	-0.005	0.008	5.80E-01
WHR	rs2839108	T	0.74	-0.011	0.002	1.60E-08	-0.010	0.009	2.40E-01
WHR	rs28451064	A	0.13	0.018	0.003	3.64E-09	0.009	0.012	3.90E-01
WHR	rs2294239	A	0.57	0.020	0.002	5.93E-32	0.007	0.008	4.60E-01
WHR	rs3788529	A	0.70	-0.012	0.002	8.32E-09	-0.013	0.009	1.20E-01
WHR	rs4820408	T	0.38	0.011	0.002	1.44E-10	-0.011	0.008	1.60E-01
WHR	rs510197	A	0.80	-0.015	0.002	4.37E-11	-0.008	0.010	4.50E-01
WHR	rs5762919	T	0.95	-0.028	0.004	1.60E-10	0.000	0.020	8.80E-01
WHR	rs713770	A	0.36	0.012	0.002	3.81E-11	0.005	0.008	5.00E-01
WHR	rs8141715	T	0.75	-0.014	0.002	1.05E-11	-0.008	0.009	4.80E-01
ACEI	rs4291	A	0.63	-0.028	0.003	9.09E-20	0.001	0.008	9.80E-01
BBs	rs11196549	G	0.96	-0.069	0.008	1.63E-18	0.033	0.019	7.10E-02
BBs	rs11196597	G	0.87	-0.029	0.005	4.37E-10	0.018	0.012	1.10E-01
BBs	rs17875473	C	0.92	-0.033	0.006	2.72E-09	0.026	0.014	5.40E-02
BBs	rs1801253	G	0.26	-0.046	0.003	3.18E-41	0.002	0.009	8.90E-01
BBs	rs4359161	A	0.18	-0.027	0.004	9.88E-12	0.023	0.010	2.70E-02
BBs	rs460718	A	0.33	-0.028	0.003	1.45E-17	-0.001	0.008	9.00E-01
CCB	rs113210396	T	0.05	-0.043	0.008	1.76E-08	-0.001	0.018	9.60E-01
CCB	rs114987861	G	0.97	-0.053	0.010	3.37E-08	0.010	0.024	6.40E-01
CCB	rs3821843	G	0.32	-0.034	0.003	7.60E-24	-0.002	0.008	9.90E-01
CCB	rs7340705	T	0.68	-0.024	0.003	5.03E-14	-0.007	0.008	4.00E-01
CCB	rs10828399	A	0.51	-0.019	0.003	1.14E-10	-0.004	0.008	6.80E-01
CCB	rs10828452	T	0.21	-0.030	0.004	4.14E-15	0.015	0.010	9.00E-02
CCB	rs11014170	A	0.02	-0.067	0.012	5.64E-09	0.025	0.029	3.40E-01
CCB	rs112133583	T	0.03	-0.055	0.010	1.20E-08	0.004	0.023	6.80E-01
CCB	rs12258967	G	0.30	-0.063	0.003	1.22E-78	0.010	0.008	1.70E-01
CCB	rs12780039	G	0.88	-0.029	0.005	1.29E-09	0.003	0.012	7.70E-01
CCB	rs16916914	T	0.97	-0.056	0.008	2.70E-12	0.002	0.022	8.80E-01
CCB	rs1779209	C	0.72	-0.027	0.003	3.86E-16	0.020	0.009	3.10E-02
CCB	rs1888693	G	0.66	-0.039	0.003	4.47E-34	0.015	0.008	6.50E-02
CCB	rs1998822	A	0.72	-0.020	0.003	1.14E-08	0.004	0.009	5.50E-01
CCB	rs2488136	G	0.71	-0.023	0.003	1.29E-11	0.009	0.008	4.10E-01
CCB	rs4748474	G	0.48	-0.019	0.003	1.54E-10	0.010	0.008	1.80E-01
CCB	rs61278674	A	0.90	-0.033	0.005	1.01E-09	0.022	0.013	1.10E-01
CCB	rs7076319	A	0.74	-0.032	0.003	4.80E-21	0.012	0.009	2.20E-01
CCB	rs72786098	A	0.03	-0.050	0.009	1.20E-08	0.038	0.021	4.60E-02
CCB	rs7923191	A	0.79	-0.037	0.004	9.82E-23	-0.009	0.010	4.00E-01
CCB	rs150857355	G	0.98	-0.094	0.011	5.15E-17	0.010	0.027	7.20E-01
CCB	rs2239046	G	0.32	-0.021	0.003	1.01E-10	-0.016	0.008	3.20E-02
CCB	rs714277	C	0.72	-0.020	0.003	2.46E-09	-0.003	0.009	7.60E-01
HMGCR	rs12916	T	0.60	-0.235	0.012	6.58E-83	0.009	0.008	2.40E-01
LDLR	rs6511720	T	0.12	-0.677	0.014	0.00E+00	0.016	0.012	2.30E-01

LDLR	rs688	C	0.55	-0.173	0.012	3.04E-48	-0.010	0.008	2.40E-01
NPC1L1	rs2073547	A	0.81	-0.150	0.015	4.53E-23	-0.014	0.010	1.80E-01
PCSK9	rs10888897	T	0.39	-0.162	0.000	0.00E+00	0.007	0.008	3.50E-01
PCSK9	rs2479394	A	0.72	-0.124	0.000	0.00E+00	0.000	0.009	9.30E-01

Supplementary Table 2. Characteristics of participants in the genome-wide association analysis of hepatic fat

Characteristics	Value
Female	19049 (51.9%)
Age at enrollment, years	54.9 (7.47)
Age at imaging, years	64.2 (7.56)
Coronary artery disease	1076 (2.9%)
Diabetes	1808 (4.9%)
Obese	6495 (17.7%)
Hypertension	10289 (28.0%)
Medications	
Anti-hypertensive therapy	4940 (13.5%)
Lipid-lowering therapy	5552 (15.1%)
Anthropometric data	
Weight, kg	76.8 (14.8)
Waist-to-hip ratio	0.86 (0.09)
Body-mass index, kg/m^2	26.6 (4.19)
Body fat, %	30.0 (8.17)
Estimated untreated systolic blood pressure, mmHg	137 (19.3)
Alcohol consumption	
Weekly drinks, U.S. standard	5.48 (6.37)
Weekly drinks, U.K. standard	9.58 (11.1)
Excessive alcohol intake, U.S.	2015 (5.5%)
Excessive alcohol intake, U.K.	9066 (24.7%)
Liver-associated biomarker concentrations	
Alanine aminotransferase, IU/L	23.0 (13.9)
Aspartate aminotransferase, IU/L	25.8 (10.5)
Gamma glutamyltransferase, IU/L	33.7 (33.9)
Estimated untreated lipid concentrations	
Total cholesterol, mg/dL	227 (40.7)
LDL cholesterol, mg/dL	144 (32.0)
HDL cholesterol, mg/dL	57.0 (14.5)
Triglycerides, mg/dL	126 [89-184]
Glycemic biomarker concentrations	
Glycated hemoglobin, %	5.36 (0.475)
Random glucose, mg/dL	89.9 (17.5)

Values correspond to number (%), mean (standard deviation), or median [interquartile range]. Obesity was defined as body-mass index ≥ 30 kg/m2 [1]; excessive alcohol intake, U.S. was defined as alcohol intake exceeding American Association for the Study of Liver Disease guidelines for NAFLD definition [2]; excessive alcohol intake, U.K. was defined as alcohol intake exceeding the UK Chief Medical Officers recommendations [3]. Diseases were defined as prevalent at time of initial assessment.

1. NHLBI Expert Panel (1998). Clinical Guidelines on the Identification, Evaluation, and Treatment of Overweight and Obesity in Adults--The Evidence Report. Obes. Res. 6 Suppl 2, 51S-209S.
2. Chalasani, N., Younossi, Z., Lavine, J.E., Charlton, M., Cusi, K., Rinella, M., Harrison, S.A., Brunt, E.M., and Sanyal, A.J. (2018). The diagnosis and management of nonalcoholic fatty liver disease: Practice guidance from the American Association for the Study of Liver Diseases. Hepatol. Baltim. Md 67, 328–357.
3. Department of Health (2016). UK Chief Medical Officers' Low Risk Drinking Guidelines.

Supplementary Table 3. F statistics and Steiger directionality test for studied traits

Exposure	N_Exp	R2	IVs	F	Correct_causal_direction	Steiger_pval
Body mass index	806834	0.037	312	99	TRUE	8.20E-29
Waist-to-hip ratio	697734	0.046	581	58	TRUE	5.89E-03
Type 2 diabetes	1407282	0.177	497	609	TRUE	5.10E-294
Fasting insulin	196991	0.014	38	74	TRUE	7.01E-13
Fasting glucose	196991	0.041	71	119	TRUE	1.01E-125
HDL cholesterol	403943	0.142	480	139	TRUE	4.30E-159
LDL cholesterol	440546	0.078	207	180	TRUE	3.76E-25
Triglycerides	441016	0.105	399	130	TRUE	6.87E-58
Smoking initiation	1232091	0.023	314	92	TRUE	2.58E-11
Alcohol consumption	941280	0.003	84	34	FALSE	2.70E-05
Coffee consumption	375833	0.005	12	157	TRUE	1.71E-11
Caffeine consumption	375833	0.005	24	79	TRUE	1.71E-11
Strenuous sports	350492	0.002	6	117	FALSE	1.49E-04
Television watching	422218	0.012	112	46	TRUE	3.43E-03

Supplementary Table 4. Results of false discovery rate correction

Exposure	Original *P* value	Benjamini-Hochberg Adjusted	Significant using an FDR of 0.05?
Waist-to-hip ratio	2.26E-68	5.66E-67	Yes
Body mass index	7.53E-24	9.41E-23	Yes
Type 2 diabetes	3.74E-18	3.11E-17	Yes
Television watching	4.82E-07	3.01E-06	Yes
Fasting insulin	3.26E-06	1.63E-05	Yes
Triglycerides	6.95E-06	2.90E-05	Yes
High-density lipoprotein cholesterol	7.57E-06	2.70E-05	Yes
Lifestime smoking index	2.14E-05	6.67E-05	Yes
Smoking initiation	2.19E-05	6.09E-05	Yes
Age of smoking initiation	3.35E-03	8.38E-03	Yes
Systolic blood pressure	4.68E-03	1.06E-02	Yes
CCB	8.51E-03	1.77E-02	Yes
Alcohol drinking	9.88E-03	1.90E-02	Yes
Low-density lipoprotein cholesterol	1.89E-02	3.38E-02	Yes
BBs	3.77E-02	6.29E-02	No
Strenuous sports	9.01E-02	1.41E-01	No
NPC1L1	1.46E-01	2.15E-01	No
HMGCR	2.70E-01	3.75E-01	No
Fasting glucose	3.83E-01	5.04E-01	No
Caffeine consumption	4.01E-01	5.01E-01	No
LDLR	4.41E-01	5.25E-01	No
PCSK9	4.42E-01	5.02E-01	No
Cigarettes per day	4.82E-01	5.24E-01	No
Coffee consumption	8.54E-01	8.89E-01	No
ACEi	8.56E-01	8.56E-01	No

Supplementary Table 5. Results of MR sensitivity analyses

					Inverse weighted median			Weighted median		
**Exposure**	**SNPs**	**Cochran' Q**	**MR-Egger int**	**P_intercept**	**Beta**	**95% CI**	**P**	**Beta**	**95% CI**	**P**
**Metabolic factor**										
Body mass index	312	417	0.002	0.120	0.32	0.26-0.39	7.53E-24	0.29	0.18-0.4	3.09E-07
Waist-to-hip ratio	580	859	0.001	0.525	0.51	0.46-0.57	2.26E-68	0.47	0.4-0.55	2.06E-33
Type 2 diabetes	493	1644	0.004	0.003	0.15	0.12-0.19	3.74E-18	0.10	0.06-0.14	9.27E-08
Fasting insulin	38	121	0.011	0.162	0.75	0.43-1.07	3.26E-06	0.75	0.43-1.07	4.34E-06
Fasting glucose	69	122	0.004	0.201	-0.06	-0.19-0.07	0.383	-0.03	-0.18-0.13	0.737
Systolic blood pressure_10mmHg	226	309	-0.001	0.791	0.10	0.03-0.16	0.005	0.06	-0.03-0.15	0.211
High-density lipoprotein cholesterol	480	1428	-0.005	3.13E-06	-0.13	-0.19--0.07	7.57E-06	-0.10	-0.17--0.03	0.004
Low-density lipoprotein cholesterol	207	1118	0.008	3.20E-04	-0.11	-0.2--0.02	0.019	-0.11	-0.19--0.04	0.002
Triglycerides	397	1471	0.008	5.80E-09	0.15	0.09-0.22	6.95E-06	0.18	0.1-0.25	2.17E-06
**Lifestyle factor**										
Smoking initiation	314	424	-0.004	0.077	0.13	0.07-0.18	2.19E-05	0.10	0.02-0.18	0.015
Age of smoking initiation	7	3	0.012	0.363	-0.33	-0.56--0.11	0.003	-0.29	-0.71-0.12	0.168
Cigarattes per day	19	29	0.005	0.286	0.03	-0.05-0.1	0.482	-0.02	-0.11-0.07	0.695
Lifestime smoking index	126	188	0.004	0.333	0.27	0.15-0.4	2.14E-05	0.21	0.05-0.37	0.012
Alcohol drinking	83	235	-0.009	0.005	0.33	0.08-0.58	0.010	0.43	0.17-0.69	0.001
Alcohol drinking_without_ADH1B	82	212	-0.005	0.281	0.15	-0.12-0.42	0.281	0.24	-0.05-0.52	0.101
Coffee consumption	12	38	-0.017	0.078	0.02	-0.19-0.23	0.854	0.07	-0.07-0.2	0.344
Caffeine consumption	24	63	0.004	0.393	0.06	-0.07-0.19	0.401	0.04	-0.06-0.14	0.392
Caffeine consumption from tea	19	14	-0.006	0.181	0.06	-0.09-0.21	0.433	0.12	-0.1-0.34	0.280
Caffeine consumption from coffee	20	53	0.001	0.884	0.13	-0.12-0.38	0.312	0.10	-0.1-0.29	0.346
Strenuous sports	6	75	-0.017	0.776	-0.64	-1.37-0.1	0.090	-0.25	-0.52-0.02	0.070
Television watching	112	224	-0.002	0.719	0.36	0.22-0.5	4.82E-07	0.36	0.21-0.5	1.32E-06
**Drug traget**										
β-blockers	6	8	-0.008	0.684	-0.31	-0.6--0.02	3.77E-02	-0.25	-0.56-0.06	1.15E-01
Calcium-channel blockers	23	23	0.003	0.579	-0.17	-0.3--0.04	8.51E-03	-0.15	-0.35-0.05	1.31E-01

	MR-Egger			MR-PRESSO				Contamination mixture		
**Exposure**	**Beta**	**95% CI**	**P**	**Outlier**	**Beta**	**95% CI**	**P**	**Beta**	**95% CI**	**P**
**Metabolic factor**										
Body mass index	0.21	0.06-0.37	0.008	2	0.31	0.25-0.37	1.53E-20	0.40	0.29-0.47	4.18E-18
Waist-to-hip ratio	0.46	0.3-0.63	6.38E-08	3	0.49	0.44-0.55	1.47E-57	0.50	0.45-0.58	2.84E-47
Type 2 diabetes	0.06	-0.01-0.13	0.079	8	0.15	0.13-0.18	3.17E-31	0.16	0.13-0.19	2.89E-31
Fasting insulin	0.11	-0.82-1.04	0.817	1	0.93	0.68-1.17	0.000	1.12	0.86-1.44	1.45E-11
Fasting glucose	-0.20	-0.45-0.05	0.124	1	-0.02	-0.14-0.1	0.797	0.02	-0.08-0.13	0.714
Systolic blood pressure_10mmHg	0.13	-0.13-0.38	0.323	0	0.10	0.03-0.16	0.005	0.03	-0.04-0.11	0.380
High-density lipoprotein cholesterol	0.03	-0.06-0.12	0.491	12	-0.17	-0.21--0.13	2.94E-15	-0.17	-0.2--0.13	6.04E-16
Low-density lipoprotein cholesterol	-0.30	-0.43--0.16	0.000	15	-0.05	-0.11-0	0.062	-0.07	-0.11--0.02	0.015
Triglycerides	-0.08	-0.18-0.02	0.119	10	0.26	0.21-0.3	4.63E-22	0.32	0.28-0.42	2.45E-22
**Lifestyle factor**										
Smoking initiation	0.34	0.1-0.57	0.006	1	0.12	0.06-0.18	4.71E-05	0.10	0.04-0.19	0.002
Age of smoking initiation	-0.86	-1.93-0.22	0.180	0	-0.33	-0.56--0.11	0.026	-0.33	-0.66-0	0.051
Cigarattes per day	-0.03	-0.16-0.1	0.634	0	0.03	-0.05-0.1	0.491	0.01	-0.05-0.18	0.636
Lifestime smoking index	0.03	-0.46-0.53	0.893	1	0.26	0.13-0.38	6.39E-05	0.28	0.15-0.4	2.12E-04
Alcohol drinking	0.92	0.46-1.38	2.01E-04	5	0.30	0.09-0.51	0.006	0.33	0.09-0.54	0.010
Alcohol drinking_without_ADH1B	0.55	-0.23-1.33	0.168	4	0.24	0.03-0.46	0.027	0.33	0.09-0.54	0.010
Coffee consumption	0.33	-0.03-0.68	0.105	1	0.08	-0.05-0.21	0.265	0.05	-0.06-0.16	0.367
Caffeine consumption	0.00	-0.17-0.18	0.978	2	0.07	-0.02-0.16	0.150	0.07	-0.01-0.14	0.095
Caffeine consumption from tea	0.31	-0.08-0.69	0.138	0	0.06	-0.09-0.21	0.443	0.09	-0.07-0.26	0.293
Caffeine consumption from coffee	0.10	-0.4-0.59	0.712	1	0.20	0-0.39	0.061	0.13	-0.04-0.31	0.138
Strenuous sports	-0.20	-3.12-2.72	0.899	2	-0.38	-0.55--0.22	0.020	-0.31	-0.51--0.11	0.004
Television watching	0.48	-0.19-1.15	0.163	1	0.32	0.21-0.43	5.49E-08	0.32	0.19-0.44	5.17E-07
**Drug traget**										
β-blockers	-0.09	-1.12-0.95	0.878	0	-0.31	-0.6--0.02	9.23E-02	-0.31	-0.93-0	3.33E-03
Calcium-channel blockers	-0.26	-0.58-0.07	0.137	0	-0.17	-0.3--0.04	1.53E-02	-0.20	-0.32--0.07	4.54E-03

CI, confidence interval; SNPs, single nucleotide polymorphisms.

Supplementary Table 6. Sensitivity analysis of removing SNPs associated with liver fat at the loci-wide significance level

Exposure	SNPs	Beta	SE	95% CI	P
**Metabolic factor**					
Body mass index	310	0.319	0.032	0.26-0.38	1.20E-23
Waist-to-hip ratio	577	0.487	0.028	0.43-0.54	9.73E-70
Type 2 diabetes	488	0.134	0.012	0.11-0.16	1.79E-28
Fasting insulin	36	0.878	0.127	0.63-1.13	4.79E-12
Fasting glucose	68	-0.016	0.061	-0.14-0.1	7.97E-01
Systolic blood pressure	226	0.096	0.034	0.03-0.16	4.68E-03
HDL cholesterol	473	-0.148	0.021	-0.19--0.11	1.89E-12
LDL cholesterol	197	-0.040	0.031	-0.1-0.02	1.92E-01
Triglycerides	388	0.230	0.027	0.18-0.28	8.34E-18
**Lifestyle factor**					
Smoking initiation	314	0.125	0.029	0.07-0.18	2.19E-05
Alcohol drinking	79	0.271	0.112	0.05-0.49	1.53E-02
Coffee consumption	11	0.077	0.066	-0.05-0.21	2.38E-01
Caffeine consumption	23	0.080	0.052	-0.02-0.18	1.30E-01
Strenuous sports	5	-0.307	0.076	-0.46--0.16	5.56E-05
Television	111	0.319	0.055	0.21-0.43	5.37E-09

CI, confidence interval; SE, standard error; SNPs, single nucleotide polymorphisms.
